# Supplementary material for: The Efficacy of Sunitinib Treatment of Renal Cancer Cells Is Associated with the Protein PHAX In Vitro
Source: Biology (Basel). 2020 Apr 7;9(4):74. doi: 10.3390/biology9040074 (PMC7236799; doi:10.3390/biology9040074)
Supplement: Supplementary file 1 [file biology-09-00074-s001.pdf]

PROBE	GENE	RIF1	RIF2	Combined
ILMN_3260932	LOC100130441	-2.7407	2.9805	5.7212
ILMN_2190779	PHAX	-2.37641	3.18209	5.5585
ILMN_2141030	LOC641522	-2.16777	3.21311	5.38088
ILMN_2110751	CHRNA5	-3.79759	1.50554	5.30313
ILMN_1787314	ALS2CR14	-2.99772	2.28159	5.27931
ILMN_2169839	CNBP	-3.85154	1.34875	5.20029
ILMN_3179148	LOC100128096	-1.66996	3.50327	5.17323
ILMN_2053536	RHBDL2	-3.54844	1.58103	5.12947
ILMN_3279960	LOC642784	-3.04522	2.03643	5.08165
ILMN_1680774	LOC730994	-2.54861	2.52795	5.07656
ILMN_1721011	LCN1	-3.37776	1.61364	4.9914
ILMN_3235006	LOC728105	-3.55171	1.39407	4.94578
ILMN_2117809	DUXAP3	-3.57415	1.34058	4.91473
ILMN_2274775	SLC44A4	-3.35476	1.53996	4.89472
ILMN_2206188	SHROOM4	-3.68221	1.20153	4.88374
ILMN_1742324	C1orf9	-3.45455	1.41457	4.86912
ILMN_3299583	LOC729964	-3.33622	1.48069	4.81691
ILMN_1689710	C16orf50	-1.95595	2.853	4.80895
ILMN_2078724	C14orf153	-2.84422	1.94795	4.79217
ILMN_1651315	HMG20B	-2.95713	1.80742	4.76455
ILMN_1657728	LOC643831	-2.2931	2.41076	4.70386
ILMN_1788062	SH3GL1	-3.3515	1.3501	4.7016
ILMN_2334242	CREB1	-3.36049	1.31025	4.67074
ILMN_2214997	LRRFIP1	-2.63844	2.00322	4.64166
ILMN_3235449	LOC100131997	-2.09687	2.54244	4.63931
ILMN_1671661	HSD17B7	-3.147	1.47162	4.61862
ILMN_1724490	PSPC1	-3.67536	0.926933	4.602293
ILMN_3286204	LOC646547	-3.3059	1.29215	4.59805
ILMN_1806017	PSME1	-3.07474	1.49801	4.57275
ILMN_2110986	NOM1	-2.05573	2.51459	4.57032
ILMN_1725427	B2M	-3.38532	1.17635	4.56167
ILMN_1712678	RPS27L	-3.37656	1.17772	4.55428
ILMN_2329773	RAB27A	-3.64425	0.901932	4.546182
ILMN_2281089	STEAP3	-1.57129	2.97397	4.54526
ILMN_2046896	ESRRAP2	-3.47667	1.06271	4.53938
ILMN_1812726	MORF4L1	-2.94353	1.58668	4.53021
ILMN_2393254	CAPNS1	-3.33749	1.18853	4.52602
ILMN_1815300	LOC644948	-2.87326	1.64887	4.52213
ILMN_1651506	NCOA6IP	-2.68404	1.82787	4.51191
ILMN_3178553	LOC100128126	-3.32078	1.15113	4.47191
ILMN_2162972	LYZ	-1.38533	3.05065	4.43598
ILMN_1735958	METTL2B	-2.40394	2.03033	4.43427
ILMN_1655740	SNAI2	-3.00822	1.41471	4.42293
ILMN_2074773	KCNK6	-3.13084	1.29102	4.42186
ILMN_1762436	UBB	-3.0527	1.36908	4.42178
ILMN_2331197	C21orf66	-3.62229	0.798045	4.420335
ILMN_1751368	HNRNPD	-3.05461	1.36401	4.41862
ILMN_1681189	LOC642154	-2.45487	1.95688	4.41175
ILMN_3251356	NUDT4P1	-2.70401	1.69578	4.39979
ILMN_2209993	PLIN5	-2.74022	1.65889	4.39911
ILMN_1740764	CHD3	-1.70911	2.68729	4.3964

ILMN_1653466	HES4	-3.58107	0.810549	4.391619
ILMN_1675460	LOC283412	-3.42184	0.969066	4.390906
ILMN_1733374	LOC642197	-3.34418	1.02976	4.37394
ILMN_1654141	ZDHHC20	-3.38054	0.987232	4.367772
ILMN_1738326	EIF4E2	-2.4407	1.92423	4.36493
ILMN_1722492	DUSP19	-2.81175	1.55216	4.36391
ILMN_3236955	SNORA11	-3.44524	0.91369	4.35893
ILMN_2150294	FKBP14	-3.02923	1.32637	4.3556
ILMN_3251605	KLHL28	-2.92809	1.42527	4.35336
ILMN_1756378	LOC645580	-2.69335	1.65928	4.35263
ILMN_1785071	SEPP1	-2.54343	1.78241	4.32584
ILMN_2055634	TIAF1	-3.15742	1.16786	4.32528
ILMN_3280842	LOC149501	-3.11182	1.206	4.31782
ILMN_1802518	DKFZp667M241	-3.22729	1.09022	4.31751
ILMN_1795697	PTP4A2	-3.24654	1.04681	4.29335
ILMN_2115974	GSDM1	-1.08279	3.19909	4.28188
ILMN_1810172	SFRP4	-2.50118	1.77906	4.28024
ILMN_2206411	CTAG1A	-1.46156	2.79226	4.25382
ILMN_2410523	DDR2	-3.35102	0.896074	4.247094
ILMN_1789349	UBQLN4	-3.25179	0.989457	4.241247
ILMN_1674160	BIN1	-1.505	2.73583	4.24083
ILMN_1739601	MSI1	-2.12926	2.08871	4.21797
ILMN_3179371	HNRNPK	-2.93955	1.26131	4.20086
ILMN_1704619	VPS29	-3.46784	0.729634	4.197474
ILMN_3293049	LOC284167	-2.7578	1.41248	4.17028
ILMN_1712046	CPXM1	-3.05859	1.10512	4.16371
ILMN_3243175	LOC100132727	-3.27853	0.884322	4.162852
ILMN_3234884	KIF22	-3.09671	1.06115	4.15786
ILMN_3235637	LOC100128288	-2.6986	1.45641	4.15501
ILMN_2262275	TRIM13	-1.23509	2.91958	4.15467
ILMN_2091123	HCG2P7	-2.56158	1.59255	4.15413
ILMN_2058337	ROCK2	-2.93263	1.21152	4.14415
ILMN_2219437	PRRG4	-2.86674	1.26475	4.13149
ILMN_2042941	TMEM159	-1.00162	3.12741	4.12903
ILMN_2078547	HSPC268	-2.92666	1.19441	4.12107
ILMN_2119937	NDUFB3	-1.99541	2.11055	4.10596
ILMN_2347424	MBOAT2	-1.78701	2.31685	4.10386
ILMN_3253891	LOC100129144	-2.09241	2.01143	4.10384
ILMN_1776552	FUBP1	-2.18687	1.91591	4.10278
ILMN_1775944	ABI2	-2.48845	1.60944	4.09789
ILMN_1730611	RTN4	-1.06488	3.03265	4.09753
ILMN_2085922	WRB	-3.33097	0.766286	4.097256
ILMN_2279367	FCAR	-2.07284	2.02236	4.0952
ILMN_2401770	PHF14	-3.17071	0.917152	4.087862
ILMN_2191428	UBB	-2.79107	1.29486	4.08593
ILMN_2299661	TNFRSF25	-1.80484	2.26172	4.06656
ILMN_1693225	LOC646897	-3.05031	1.01418	4.06449
ILMN_3289128	LOC653695	-3.02565	1.03274	4.05839
ILMN_2279339	WTAP	-2.19124	1.85858	4.04982
ILMN_1656662	LOC731365	-2.35325	1.67958	4.03283
ILMN_3243549	LOC100133465	-1.07825	2.94616	4.02441
ILMN_3265742	LOC100130835	-2.62516	1.39784	4.023

ILMN_2406468	NFU1	-3.14071	0.880825	4.021535
ILMN_2307740	CD46	-2.30288	1.71204	4.01492
ILMN_2210386	TMEM17	-2.64282	1.36024	4.00306
ILMN_1695005	C1orf62	-1.17446	2.82051	3.99497
ILMN_2091707	LOC123688	-2.46792	1.52309	3.99101
ILMN_1749345	STX5	-3.19044	0.797673	3.988113
ILMN_1716816	TMEM87A	-2.93438	1.04864	3.98302
ILMN_1754558	LRRC10	-1.18241	2.79443	3.97684
ILMN_1718932	MTRR	-2.97162	1.00457	3.97619
ILMN_2411511	KLK12	-3.20461	0.767038	3.971648
ILMN_1810424	HRH4	-1.03926	2.92425	3.96351
ILMN_1680727	GLRX2	-1.94305	2.01966	3.96271
ILMN_1768392	LOC648059	-1.71929	2.22805	3.94734
ILMN_2176882	ZNF69	-2.90509	1.04207	3.94716
ILMN_3241626	QRFPR	-0.81479	3.12804	3.942826
ILMN_3206827	LOC100131737	-2.61982	1.32209	3.94191
ILMN_2115949	ZNF394	-2.99883	0.939348	3.938178
ILMN_3240236	SMCR5	-1.17406	2.76209	3.93615
ILMN_1685538	LOC158345	-1.00894	2.92511	3.93405
ILMN_1805161	LZTR1	-2.61139	1.31803	3.92942
ILMN_3310151	MIR1909	-1.36082	2.56416	3.92498
ILMN_1677534	SCAP	-1.35437	2.56022	3.91459
ILMN_1730940	KLHDC3	-3.1466	0.762961	3.909561
ILMN_3251460	ZNF765	-1.12998	2.77652	3.9065
ILMN_1698463	ILF3	-3.0788	0.795252	3.874052
ILMN_2359742	CTSB	-3.22904	0.644215	3.873255
ILMN_1769319	CNBP	-2.83085	1.03637	3.86722
ILMN_3304850	LOC730098	-1.6328	2.22757	3.86037
ILMN_3247821	TMEM206	-2.8934	0.966249	3.859649
ILMN_2154322	SEMA3E	-2.11764	1.74065	3.85829
ILMN_1802096	ABTB1	-2.48105	1.36718	3.84823
ILMN_2117569	DEM1	-2.69987	1.14747	3.84734
ILMN_1689798	LOC643817	-2.42463	1.41704	3.84167
ILMN_1716093	KRT10	-2.58227	1.25341	3.83568
ILMN_2160388	RPL24	-3.19506	0.640469	3.835529
ILMN_2184184	ANXA1	-3.06297	0.77244	3.83541
ILMN_1655564	KIAA1245	-2.42487	1.39797	3.82284
ILMN_1815205	LYZ	-3.12519	0.686714	3.811904
ILMN_3239946	LOC729375	-2.26915	1.52554	3.79469
ILMN_1666503	DENND2A	-2.3663	1.42454	3.79084
ILMN_1749011	NECAP2	-3.09527	0.694519	3.789789
ILMN_1883024	LOC732424	-2.44206	1.34629	3.78835
ILMN_3239811	LOC100133767	-2.70409	1.08183	3.78592
ILMN_1709571	LOC654126	-2.78478	0.98609	3.77087
ILMN_2178201	ZNF43	-1.00147	2.76378	3.76525
ILMN_2151168	SLC30A6	-1.06894	2.69402	3.76296
ILMN_3246145	EXOG	-1.04157	2.71902	3.76059
ILMN_2147306	PNRC2	-3.03448	0.723734	3.758214
ILMN_3246409	HNRNPH1	-3.21328	0.542009	3.755289
ILMN_1805623	SNIP	-2.09631	1.65753	3.75384
ILMN_3180420	LOC100129269	-2.25689	1.4949	3.75179
ILMN_1763080	QARS	-3.1201	0.628427	3.748527

ILMN_1662772	LOC653238	-3.08716	0.660648	3.747808
ILMN_3240003	LOC100133012	-3.07236	0.666601	3.738961
ILMN_1794912	ATP5H	-3.15822	0.580005	3.738225
ILMN_3248941	C6orf225	-1.51336	2.22292	3.73628
ILMN_3251217	PDXDC2	-2.40426	1.32892	3.73318
ILMN_1701052	TUBG2	-2.45566	1.2766	3.73226
ILMN_1671893	CHMP2A	-2.95276	0.774944	3.727704
ILMN_1756784	FREQ	-1.09656	2.62812	3.72468
ILMN_2148459	B2M	-2.95101	0.768801	3.719811
ILMN_1695719	EIF2C2	-2.47858	1.22033	3.69891
ILMN_1661142	TMF1	-2.79918	0.899346	3.698526
ILMN_3285162	LOC100131541	-2.47428	1.22228	3.69656
ILMN_1741640	ZNF675	-1.79163	1.8956	3.68723
ILMN_2168217	EBI2	-2.7399	0.94378	3.68368
ILMN_1693090	CROT	-2.49696	1.18619	3.68315
ILMN_2196078	SLAMF6	-2.54765	1.13259	3.68024
ILMN_1725791	PTPLA	-1.27265	2.40605	3.6787
ILMN_1675239	NDUFA7	-3.2553	0.407239	3.662539
ILMN_1660579	CRLF3	-1.9951	1.66663	3.66173
ILMN_2321931	DCLRE1C	-1.97687	1.68466	3.66153
ILMN_3239766	FKBP1P1	-2.66168	0.997398	3.659078
ILMN_2361186	PHF20L1	-2.36877	1.28628	3.65505
ILMN_1734476	KIF2A	-2.53595	1.11061	3.64656
ILMN_2370135	HNRNPU	-2.1217	1.52475	3.64645
ILMN_1690963	ASAP1	-3.17015	0.46826	3.63841
ILMN_3248164	LOC100132096	-2.85568	0.781115	3.636795
ILMN_1688971	NOL11	-1.04619	2.58773	3.63392
ILMN_1689327	LOC730534	-2.29031	1.34305	3.63336
ILMN_2132982	IGFBP5	-1.84383	1.78026	3.62409
ILMN_1679045	SBDS	-2.19291	1.42172	3.61463
ILMN_2297205	ATRN	-1.69383	1.91983	3.61366
ILMN_2189842	SNORA10	-1.75837	1.85353	3.6119
ILMN_2345824	PCDHGC3	-2.93223	0.672578	3.604808
ILMN_1768732	SPAG16	-3.05063	0.553631	3.604261
ILMN_1803597	FGL1	-2.85464	0.744421	3.599061
ILMN_3295075	LOC100131531	-2.29063	1.30767	3.5983
ILMN_2235707	ITIH5	-1.45973	2.13585	3.59558
ILMN_1691449	LOC646630	-3.10976	0.483107	3.592867
ILMN_1693340	RAC3	-0.90041	2.68987	3.590277
ILMN_3249449	LOC729737	-2.4388	1.1433	3.5821
ILMN_1666635	RPS14	-0.99828	2.58098	3.579262
ILMN_1683096	ASB1	-3.20755	0.37029	3.57784
ILMN_3249331	LOC100133482	-2.30448	1.26434	3.56882
ILMN_1665550	ZNF528	-1.36297	2.19998	3.56295
ILMN_1738482	CEP27	-1.62444	1.93776	3.5622
ILMN_3243846	FOLR4	-3.05795	0.502528	3.560478
ILMN_1671314	UXT	-2.30957	1.24677	3.55634
ILMN_2402558	ZHX1	-2.03337	1.52073	3.5541
ILMN_1812578	LOC645636	-1.22172	2.33201	3.55373
ILMN_3236102	RNU5D	-0.27719	3.27416	3.55135
ILMN_1756669	POGK	-2.38158	1.16432	3.5459
ILMN_2288483	C3orf34	-0.22793	3.31302	3.540954

ILMN_1804601	LOC649923	-2.79202	0.74635	3.53837
ILMN_2076415	TBCCD1	-0.82935	2.70632	3.535668
ILMN_1810462	C10orf115	-0.95842	2.57502	3.533436
ILMN_2130441	HLA-H	-2.89913	0.631445	3.530575
ILMN_2285713	TDP1	-1.32787	2.19617	3.52404
ILMN_1671054	HLA-A	-3.02963	0.492716	3.522346
ILMN_1690520	LOC651213	-2.80038	0.721805	3.522185
ILMN_1776154	COG3	-2.53715	0.982812	3.519962
ILMN_3182020	LOC100129441	-2.08788	1.43156	3.51944
ILMN_1668634	FBXW7	-3.1867	0.330448	3.517148
ILMN_1736234	C1orf77	-2.18424	1.33037	3.51461
ILMN_2273224	SLC4A5	-2.15034	1.3507	3.50104
ILMN_1689111	CXCL12	-1.46243	2.02876	3.49119
ILMN_1665423	ZFP91	-2.25713	1.23318	3.49031
ILMN_1708934	ADM	-2.30623	1.18116	3.48739
ILMN_3210217	LOC728054	-2.46036	1.02629	3.48665
ILMN_1726597	FAM65B	-1.84001	1.64561	3.48562
ILMN_3308335	RNU6-1	-3.15257	0.330892	3.483462
ILMN_1675448	ZFP36L1	-2.33845	1.13797	3.47642
ILMN_1658689	LOC652736	-2.71316	0.762606	3.475766
ILMN_1783276	NEXN	-1.5175	1.95572	3.47322
ILMN_1680850	RECQL	-2.72136	0.750723	3.472083
ILMN_1676588	CEPT1	-2.95801	0.511398	3.469408
ILMN_3310351	RNU6-15	-3.09774	0.371213	3.468953
ILMN_3235514	GPR183	-2.2857	1.18036	3.46606
ILMN_2293322	MTHFD2	-1.30536	2.15433	3.45969
ILMN_1686165	KRT126P	-2.43816	1.01372	3.45188
ILMN_2213297	C11orf54	-2.60073	0.8459	3.44663
ILMN_1810759	C2orf25	-2.27782	1.16813	3.44595
ILMN_1671971	LOC644743	-2.13443	1.30421	3.43864
ILMN_1740976	NONO	-2.63645	0.795128	3.431578
ILMN_1664750	TMBIM4	-1.85974	1.56844	3.42818
ILMN_2376520	PPP2R3A	-0.74222	2.67702	3.419236
ILMN_3267800	LOC100130276	-0.77134	2.64736	3.4187
ILMN_2343048	ABCB9	-1.00584	2.41181	3.41765
ILMN_1651316	CD69	-1.36289	2.05457	3.41746
ILMN_1704222	C1orf27	-2.59062	0.819573	3.410193
ILMN_1786759	C11orf10	-1.70664	1.68475	3.39139
ILMN_1680579	ATP2B4	-2.48312	0.90527	3.38839
ILMN_1749417	LOC649495	-1.40506	1.9808	3.38586
ILMN_1731838	COX6BP1	-2.73385	0.649014	3.382864
ILMN_2320853	UBE2D3	-1.27941	2.10269	3.3821
ILMN_3238618	C3orf50	-2.03074	1.34578	3.37652
ILMN_1692731	TTYH3	-3.17246	0.197631	3.370091
ILMN_1720249	LOC648057	-2.39583	0.969338	3.365168
ILMN_3185204	LOC100127894	-1.43912	1.92346	3.36258
ILMN_1795228	ZFAND5	-0.89364	2.46885	3.362493
ILMN_1760992	DPY19L2P2	-1.83324	1.52771	3.36095
ILMN_2154053	FVT1	-2.10292	1.25068	3.3536
ILMN_2106265	GDPD1	-1.88441	1.46534	3.34975
ILMN_2336109	L3MBTL2	-2.79178	0.557909	3.349689
ILMN_1665291	NUB1	-3.10649	0.238905	3.345395

ILMN_3244405	LOC100134172	-0.68986	2.65519	3.345047
ILMN_1704286	FXYD5	-3.05	0.293985	3.343985
ILMN_1798581	MCM8	-1.72152	1.6145	3.33602
ILMN_1666082	HEATR3	-1.14565	2.18972	3.33537
ILMN_1734826	NUP88	-3.06217	0.264486	3.326656
ILMN_1766309	ANKRD54	-3.1762	0.150143	3.326343
ILMN_2389273	FXR1	-3.02334	0.300737	3.324077
ILMN_1801456	CROP	-2.57216	0.743362	3.315522
ILMN_1783304	ATP1B3	-0.58832	2.72666	3.314981
ILMN_1760667	POLR3GL	0.414141	2.89877	3.312911
ILMN_2138019	HEPACAM	-2.30026	1.00861	3.30887
ILMN_3227321	LOC731542	0.514085	2.79057	3.304655
ILMN_3200106	LOC100131301	-0.92118	2.38072	3.301896
ILMN_2394498	SYF2	-2.50635	0.794181	3.300531
ILMN_1684321	CYB5B	-1.53827	1.7615	3.29977
ILMN_1694878	LOC644979	-1.72797	1.56993	3.2979
ILMN_2411723	RPSA	-2.04562	1.25206	3.29768
ILMN_1793598	APIP	-1.85075	1.4428	3.29355
ILMN_2361104	ZMAT3	-0.99033	2.30007	3.290395
ILMN_2352023	RIPK5	-2.56778	0.720923	3.288703
ILMN_1730084	COMT	-2.97997	0.292943	3.272913
ILMN_2095660	TMEM156	-1.05223	2.21934	3.27157
ILMN_1801905	ATG4D	-1.64052	1.62278	3.2633
ILMN_1692486	ZNRD1	-2.59293	0.669595	3.262525
ILMN_1698650	C17orf41	-1.22244	2.03926	3.2617
ILMN_2202637	CRY1	-2.98888	0.272055	3.260935
ILMN_3239084	LOC100131968	-2.25928	1.0002	3.25948
ILMN_1738333	CCDC50	-2.63502	0.623516	3.258536
ILMN_1703006	MTCH1	-0.82473	2.43235	3.257083
ILMN_1652394	RAB2A	-0.32812	2.92522	3.253337
ILMN_3247069	RAET1K	-0.7894	2.45374	3.243143
ILMN_1803162	RTEL1	-2.54518	0.694448	3.239628
ILMN_3241354	LOC390660	-2.0617	1.17783	3.23953
ILMN_2067370	SNRPF	-1.00234	2.2298	3.23214
ILMN_2377025	TCOF1	-2.55808	0.673714	3.231794
ILMN_1682864	SPSB3	-2.7177	0.512824	3.230524
ILMN_3273268	FAM75C1	-2.34052	0.889103	3.229623
ILMN_1687865	TMOD3	-0.69121	2.53594	3.227149
ILMN_3260910	CSAG3	-1.28069	1.94552	3.22621
ILMN_1756982	CLIC1	-2.43163	0.790134	3.221764
ILMN_1716678	NPC2	-2.39376	0.827461	3.221221
ILMN_1780591	FAT3	-2.73576	0.484053	3.219813
ILMN_1685239	GABPAP	-0.94306	2.27275	3.215808
ILMN_1712320	DDX50	-2.97774	0.237207	3.214947
ILMN_1710075	FAM89A	-2.45073	0.762931	3.213661
ILMN_1792473	AIF1	-2.84327	0.370308	3.213578
ILMN_2393765	IGLL1	-3.02788	0.183062	3.210942
ILMN_1794863	CAMK2N1	-1.40315	1.80639	3.20954
ILMN_1719072	LOC643011	-1.08152	2.12161	3.20313
ILMN_1653127	HERC2P2	-2.98109	0.217123	3.198213
ILMN_1737833	ATN1	-2.68555	0.5118	3.19735
ILMN_1705534	LOC651112	-0.37483	2.8156	3.190426

ILMN_1771898	NBPF3	-2.7765	0.406274	3.182774
ILMN_3249624	LOC641298	-2.54129	0.638168	3.179458
ILMN_1707286	FLJ22662	-2.20835	0.97016	3.17851
ILMN_1701289	MGC40489	-2.7734	0.401209	3.174609
ILMN_3236443	ALX1	0.423292	2.74928	3.172572
ILMN_3176403	FTHL16	-2.49497	0.677501	3.172471
ILMN_1802459	LOC643138	-2.42845	0.741711	3.170161
ILMN_1797578	CASP10	-2.5171	0.649162	3.166262
ILMN_1784380	DTX3L	-2.87812	0.286683	3.164803
ILMN_1807994	PCNP	-1.82465	1.3344	3.15905
ILMN_2110532	RPL26L1	-0.91401	2.24255	3.156564
ILMN_1652209	LOC731432	-0.96566	2.1863	3.151957
ILMN_1773567	LAMA5	-2.60057	0.551045	3.151615
ILMN_1712435	LOC644294	-0.62063	2.52904	3.149668
ILMN_1811644	FAM106A	-2.23859	0.90785	3.14644
ILMN_2058141	HMGN2	-1.73441	1.41129	3.1457
ILMN_1693740	LOC647834	-0.85807	2.2859	3.143974
ILMN_1745076	CLINT1	-1.76895	1.36942	3.13837
ILMN_1661519	KIAA0408	-1.54105	1.59208	3.13313
ILMN_2261519	AIRE	-0.70426	2.42719	3.131449
ILMN_2157075	LRCH4	-0.9398	2.18964	3.129436
ILMN_1695562	FLJ11292	-2.61887	0.50712	3.12599
ILMN_3243428	SNORA77	-2.74197	0.383522	3.125492
ILMN_2361478	PDLIM5	-2.95968	0.165546	3.125226
ILMN_1752810	LARP6	-1.55346	1.56885	3.12231
ILMN_1771320	FLJ38717	-2.11461	1.00445	3.11906
ILMN_1728305	PUM2	-2.42145	0.692277	3.113727
ILMN_2042595	PCM1	-1.04809	2.06508	3.11317
ILMN_2175465	RSL24D1	-1.33034	1.77897	3.10931
ILMN_1752837	ARL8B	-2.55481	0.552968	3.107778
ILMN_3231944	LOC100130516	0.4443	2.66346	3.10776
ILMN_2061310	ZNF280C	-0.95818	2.14436	3.102537
ILMN_3237329	FNIP2	-0.7863	2.31606	3.102359
ILMN_3201290	LOC100132896	-0.41127	2.69096	3.102231
ILMN_2309848	FXYD5	-2.80244	-0.29965	3.102094
ILMN_2398474	RAP1B	0.444857	2.65337	3.098227
ILMN_1754303	RPL30	-1.83078	1.26597	3.09675
ILMN_3251695	TFAMP1	-0.86134	2.23534	3.096677
ILMN_2379326	MAP3K7	-1.65237	1.4432	3.09557
ILMN_1758457	TBC1D16	-1.34403	1.74951	3.09354
ILMN_2298958	MRRF	-0.35199	2.73979	3.091779
ILMN_1660375	XAGE2	-1.39209	1.69755	3.08964
ILMN_1813657	PHF20	-0.69057	2.39824	3.088813
ILMN_3209832	LOC100131801	-2.38588	0.699313	3.085193
ILMN_1730999	TPR	-1.58402	1.49829	3.08231
ILMN_1797055	LIN52	-0.4349	2.64727	3.082174
ILMN_1737195	CENPK	-1.32342	1.75296	3.07638
ILMN_1797384	UROS	-1.91091	1.16445	3.07536
ILMN_2064311	C1orf151	-0.68954	2.38414	3.073678
ILMN_1663631	BANP	-1.73203	1.33482	3.06685
ILMN_2151739	CAT	-1.13761	1.92726	3.06487
ILMN_2407851	IL17RD	-1.35779	1.70519	3.06298

ILMN_3249501	ZNF697	-2.99048	0.065707	3.0561871
ILMN_3299682	LOC729780	-2.32787	-0.72803	3.055901
ILMN_3247906	RNF114	-2.22538	0.81933	3.04471
ILMN_1785703	LMOD3	-1.03871	2.00524	3.04395
ILMN_1755909	C20orf11	-2.40935	-0.62542	3.034766
ILMN_2096442	LOC260339	-1.00696	2.02218	3.02914
ILMN_1695430	DDB1	-2.18154	0.844485	3.026025
ILMN_2052383	R3HDM1	-0.98929	2.03527	3.02456
ILMN_3239130	AGAP6	-2.24145	0.780027	3.021477
ILMN_1661903	CYLC2	-1.13547	1.88084	3.01631
ILMN_3252733	CBY3	-2.58489	0.430915	3.015805
ILMN_3277365	LOC100133233	-1.9429	1.0701	3.013
ILMN_2144116	CPSF2	-0.58418	2.42671	3.010893
ILMN_3188106	CYTH2	-1.9896	1.02119	3.01079
ILMN_3249965	LOC646278	-2.856	0.1547	3.0107
ILMN_2352090	GPRC5C	-1.66558	-1.34369	3.00927
ILMN_1670589	LOC643933	-1.02128	1.98448	3.00576
ILMN_1760718	ZMIZ2	-1.17589	1.82978	3.00567
ILMN_1805643	RILPL1	-1.3379	1.66758	3.00548
ILMN_1809750	TDRD1	-0.89142	2.11157	3.002985
ILMN_1789106	IPP	-0.77261	2.22939	3.002003
ILMN_2097421	MRPL51	-1.94671	1.05335	3.00006
ILMN_3227315	LOC729009	-1.87621	1.11787	2.99408
ILMN_2414786	DIO1	0.132452	2.86011	2.992562
ILMN_1676459	ZNF785	-1.56826	1.42185	2.99011
ILMN_1654396	ITGB2	-2.24066	0.745871	2.986531
ILMN_1662097	SLC25A43	-1.16761	1.81475	2.98236
ILMN_1722186	C2orf56	-2.44787	0.5236	2.97147
ILMN_1701314	LOC399900	-1.74404	1.22312	2.96716
ILMN_1737988	PRNP	-2.56914	0.397848	2.966988
ILMN_3221790	LOC729123	-2.4559	0.510191	2.966091
ILMN_1700182	LOC400721	-2.12154	0.840645	2.962185
ILMN_1714278	C9orf30	-1.01937	1.94264	2.96201
ILMN_1768491	TBC1D25	-1.83412	1.12131	2.95543
ILMN_1667169	LOC440349	-0.8942	2.05994	2.95414
ILMN_1761476	TTC3	-2.73696	0.211744	2.948704
ILMN_3239969	LOC100133795	-2.06066	0.886991	2.947651
ILMN_2175912	ITGB2	-2.29501	0.652612	2.947622
ILMN_1706345	TCTN2	-1.03306	1.9122	2.94526
ILMN_3287996	LOC400446	-0.73712	2.20741	2.944533
ILMN_2353490	PAX2	-2.80984	-0.13301	2.942853
ILMN_3301468	LOC729324	-0.97019	1.97261	2.942803
ILMN_2206272	D2HGDH	-2.86324	-0.07922	2.9424573
ILMN_3175797	LOC100130696	-0.74062	2.20003	2.940654
ILMN_1736008	YRDC	-1.59803	1.34057	2.9386
ILMN_2106656	BLZF1	-1.10419	1.83416	2.93835
ILMN_1775743	BTG1	-2.77054	0.167422	2.937962
ILMN_1691858	LOC648039	-0.8977	2.03589	2.933592
ILMN_3260286	LOC100128062	-0.86874	2.06413	2.932867
ILMN_3248975	PPP4C	-1.86444	1.06614	2.93058
ILMN_3206343	LOC644914	-2.13013	0.798602	2.928732
ILMN_3243749	C7orf64	-0.78296	2.14234	2.925297

ILMN_3253304	BRI3P1	-2.75518	-0.16644	2.921615
ILMN_1812640	AHR	-1.43458	1.48626	2.92084
ILMN_2304624	EIF4H	-2.82385	-0.09309	2.9169438
ILMN_3284845	LOC100132797	-0.75972	2.15709	2.916812
ILMN_2106380	TNFSF15	-1.21249	1.70189	2.91438
ILMN_2235745	GRIPAP1	-1.15393	1.75816	2.91209
ILMN_2157020	SNORD48	-2.78939	-0.12239	2.911775
ILMN_1657153	ACTR3	-2.25872	0.652322	2.911042
ILMN_1696911	FTHL8	-2.57423	0.334229	2.908459
ILMN_1724544	PPP4R1	0.787817	2.12055	2.908367
ILMN_1694432	CRIP2	-1.67139	-1.23661	2.908
ILMN_1713174	TCP11L1	-2.56993	0.333626	2.903556
ILMN_1813694	LOC642759	0.459259	2.44241	2.901669
ILMN_3236680	LOC100134393	0.491884	2.40976	2.901644
ILMN_2253065	H2AFJ	-1.93088	0.968006	2.898886
ILMN_1749809	ZNF813	-0.61609	2.28258	2.898672
ILMN_3297013	PACRGL	-0.69169	2.20677	2.898457
ILMN_1750093	SDHALP1	-1.01531	1.88286	2.89817
ILMN_2374036	CTSL1	-2.43935	0.458508	2.897858
ILMN_1666156	MORF4L2	-0.17597	2.71998	2.895953
ILMN_2061446	AADACL1	-2.56341	0.331886	2.895296
ILMN_3268329	LOC100130262	-1.84526	1.04828	2.89354
ILMN_3243223	FLJ41562	-0.61251	2.28079	2.893301
ILMN_1791226	NXN	-2.15679	-0.73511	2.891904
ILMN_2412281	PDLIM5	-2.65332	-0.23647	2.889788
ILMN_3201663	LOC645231	-2.06536	0.823248	2.888608
ILMN_1741003	ANXA5	-2.5977	0.289298	2.886998
ILMN_3231338	TIMM23B	-1.46426	1.42255	2.88681
ILMN_1738579	VHL	-2.61091	-0.2759	2.886806
ILMN_3208264	LOC100132113	-2.10491	0.781334	2.886244
ILMN_1683664	LOC650369	-1.41334	1.47218	2.88552
ILMN_1768798	SPAG9	-2.57017	0.314708	2.884878
ILMN_1811574	MAPK8IP3	-2.35246	0.530624	2.883084
ILMN_1782247	KAT2A	-2.14867	0.734178	2.882848
ILMN_2262288	EEF1G	-0.72328	2.15779	2.881067
ILMN_1760506	HSD17B7P2	-2.33392	0.544536	2.878456
ILMN_2366388	PRDX1	-2.39455	-0.48259	2.877142
ILMN_3294158	LOC100132562	-0.62134	2.25551	2.876854
ILMN_1664010	ELF1	-2.78422	0.088738	2.8729578
ILMN_1727080	MYO6	-2.41118	-0.4586	2.869777
ILMN_3298829	LOC729505	-0.79068	2.0791	2.869776
ILMN_2186806	HLA-F	-2.74149	0.127586	2.869076
ILMN_3246713	SNORA22	-2.25283	-0.61205	2.864879
ILMN_1801795	LOC347544	-1.82505	-1.03695	2.862
ILMN_2225974	GCLM	-1.30663	1.55476	2.86139
ILMN_1737585	VARS2	-2.28792	0.568596	2.856516
ILMN_1737580	PPPDE2	1.82251	-1.0326	2.85511
ILMN_1760635	RAD51C	-1.56651	1.28667	2.85318
ILMN_1664398	LOC651621	-2.33511	0.515741	2.850851
ILMN_1674380	TRPC1	-1.90589	-0.94461	2.8505
ILMN_1709817	SLC35A5	-2.66255	-0.1866	2.849151
ILMN_1655126	PI4KAP2	-1.97687	0.87086	2.84773

ILMN_1726901	KLC1	-1.47572	1.37183	2.84755
ILMN_1724230	LOC642236	-2.14254	-0.70495	2.847493
ILMN_1672536	FBLN1	-1.37657	1.46978	2.84635
ILMN_3248403	LOC100132717	-1.60439	1.24147	2.84586
ILMN_1674608	FAM169B	-1.34077	-1.50448	2.84525
ILMN_1698213	RBM3	-1.39535	-1.44404	2.83939
ILMN_3220802	LOC728991	-0.92165	1.91517	2.836823
ILMN_1753342	SAT1	0.572731	2.26404	2.836771
ILMN_1756793	POLS	-1.34482	1.48833	2.83315
ILMN_1792078	RNF114	-1.91584	0.9153	2.83114
ILMN_3250993	LOC649203	-2.58569	0.245444	2.831134
ILMN_1651606	LOC653210	-1.60805	1.2218	2.82985
ILMN_2399264	Sep-06	-2.41779	0.410878	2.828668
ILMN_1758474	PRKRA	0.892297	1.93521	2.827507
ILMN_2380101	PHACTR4	-1.03449	1.79166	2.82615
ILMN_1743427	SCYL3	-1.28467	1.54131	2.82598
ILMN_1739583	ROCK1	1.79123	-1.03429	2.82552
ILMN_2364357	RPS6KB2	-1.79759	1.02781	2.8254
ILMN_1778401	HLA-B	-2.70584	0.117124	2.822964
ILMN_1658904	ID2B	-2.23023	-0.59208	2.822313
ILMN_1673421	LOC440704	-0.9994	1.82058	2.819976
ILMN_3247341	LOC100134739	-0.87662	1.94222	2.818835
ILMN_1693452	GAL3ST4	-0.9047	1.91296	2.817664
ILMN_1735274	LOC644038	-0.58816	2.22889	2.817052
ILMN_1801914	SH3BP2	-1.38175	-1.43523	2.81698
ILMN_1719303	P4HB	1.57082	-1.24347	2.81429
ILMN_1755749	PGK1	-2.14541	0.664577	2.809987
ILMN_1679555	TIMM23	-1.31188	-1.49522	2.8071
ILMN_3309443	MIR365-1	-2.76719	0.039185	2.806375
ILMN_1672589	SEMA4B	-1.43084	1.37451	2.80535
ILMN_2091792	ENTPD6	-1.79752	1.00596	2.80348
ILMN_1656678	SUZ12P	-1.05335	1.7501	2.80345
ILMN_1736856	PDPR	-0.82712	1.97583	2.80295
ILMN_3235410	HIATL2	-0.77413	2.02637	2.800495
ILMN_1676289	NCAM1	-1.25236	1.54808	2.80044
ILMN_1789187	LOC730809	-2.36113	0.438779	2.799909
ILMN_1706734	ZNF451	0.53008	2.26605	2.79613
ILMN_1725750	LOC644695	-0.79044	2.00473	2.795167
ILMN_1740716	RBM26	-1.60885	1.18307	2.79192
ILMN_1806106	GNL3	-1.5693	-1.22201	2.79131
ILMN_1805187	LOC727759	-2.57606	0.211714	2.787774
ILMN_1795275	USP53	-1.67927	1.10192	2.78119
ILMN_3235896	LOC100132640	0.853385	1.92778	2.781165
ILMN_1704941	PPCDC	-2.34959	-0.43055	2.780144
ILMN_1754795	FAT1	-2.68301	-0.09674	2.7797548
ILMN_1812571	RAB35	-2.57113	0.203193	2.774323
ILMN_3236211	DGCR11	-2.08835	0.684312	2.772662
ILMN_1699265	TNFRSF10B	-2.75056	-0.02084	2.7714016
ILMN_1704154	TNFRSF19	-1.66853	1.09985	2.76838
ILMN_2099783	ATP6V1F	-2.43434	-0.3321	2.76644
ILMN_1783954	FOXN3	1.90693	-0.85831	2.765239
ILMN_1800634	NME4	1.89834	-0.86667	2.765011

ILMN_2234016	FTHL7	-2.22554	0.536174	2.761714
ILMN_1795865	FGFRL1	-0.56039	2.20064	2.761031
ILMN_1658460	LOC653884	-1.19061	1.57019	2.7608
ILMN_1744059	DCTN6	-1.38294	1.3765	2.75944
ILMN_2152095	RNASEN	-2.29461	-0.46443	2.759036
ILMN_1746012	MBD6	-2.53078	-0.22763	2.758413
ILMN_3239108	SNORA3	-1.40291	1.35472	2.75763
ILMN_1695020	NEK3	-2.50444	-0.25169	2.756133
ILMN_3237277	LOC100133130	-1.70585	1.04992	2.75577
ILMN_1664614	FAU	0.554668	2.1997	2.754368
ILMN_3225761	MGC87895	0.404991	2.34793	2.752921
ILMN_1665004	CASC3	1.36226	-1.39022	2.75248
ILMN_1775045	ADIPOQ	-1.33822	1.41151	2.74973
ILMN_3236982	LOC730476	-1.71364	-1.03607	2.74971
ILMN_1693136	VTI1B	1.6636	-1.08561	2.74921
ILMN_1704315	LOC389435	-1.35644	1.39087	2.74731
ILMN_1692849	ANKRD58	-1.22737	1.51805	2.74542
ILMN_1753524	HIST1H2AB	-1.43864	1.30467	2.74331
ILMN_1761722	ZNF579	-2.15671	0.585905	2.742615
ILMN_2226753	ZC3HAV1L	-0.78702	1.9544	2.741415
ILMN_1815024	PRDX5	-1.41151	1.32984	2.74135
ILMN_3243334	LOC645762	-2.31951	-0.42042	2.739934
ILMN_1695745	DISP1	-2.2927	-0.44566	2.738362
ILMN_3245130	LOC100134708	-0.50827	2.22979	2.738061
ILMN_1672427	LOC729882	-2.62993	-0.10791	2.737835
ILMN_3249230	LOC100133649	-1.1821	1.55519	2.73729
ILMN_2369785	SNRPD2	-2.60741	-0.12903	2.73644
ILMN_1784627	CDC42BPA	-2.46474	-0.27098	2.735718
ILMN_1692864	PDLIM5	-2.04395	-0.69166	2.735606
ILMN_1761277	PDE4C	-0.70386	2.03115	2.735011
ILMN_1793017	DGKQ	-0.82831	1.90669	2.735004
ILMN_3220265	LOC100132553	1.9207	-0.81282	2.733522
ILMN_2173909	ZNF14	-1.31172	1.42115	2.73287
ILMN_1810474	UBE2I	-2.47604	-0.25683	2.73287
ILMN_1800602	GCA	0.232555	2.4966	2.729155
ILMN_2366391	PRDX1	-1.73521	-0.993	2.728212
ILMN_1719433	CD1D	-1.82059	-0.90563	2.726221
ILMN_2252136	YWHAE	-1.52609	1.19917	2.72526
ILMN_3274596	LOC286512	-1.0874	1.63679	2.72419
ILMN_2221066	VDAC2	-1.37769	1.34624	2.72393
ILMN_3238213	KILLIN	-0.48543	2.23839	2.723823
ILMN_1801928	YWHAZ	-0.65683	2.06168	2.718514
ILMN_1682717	IER3	-2.45399	-0.26384	2.717832
ILMN_1652969	ZNF479	-1.70131	-1.01549	2.7168
ILMN_3188110	C19orf60	-2.10005	0.616622	2.716672
ILMN_1778121	CALN1	-1.88361	0.832639	2.716249
ILMN_1666078	HLA-H	-0.61227	2.10388	2.716153
ILMN_2400500	LASS2	1.67577	-1.03871	2.71448
ILMN_2404320	SNTN	-0.69352	2.02019	2.713707
ILMN_1693771	ASPH	1.68349	-1.03018	2.71367
ILMN_3264112	LOC100129362	-0.93009	1.78302	2.713108
ILMN_1801553	LEO1	-0.86929	-1.84367	2.712964

ILMN_1756261	GPR143	-1.26915	1.44325	2.7124
ILMN_1763447	PLXNB2	-2.16315	-0.54651	2.709657
ILMN_1669310	RHOT2	-1.98403	0.725213	2.709243
ILMN_3242753	NBPF16	-1.29795	1.40905	2.707
ILMN_1789136	SERF2	-2.24074	0.465792	2.706532
ILMN_1700810	HSPCAL3	1.81932	-0.88096	2.700281
ILMN_1664798	GRHPR	1.14025	-1.55983	2.70008
ILMN_1675830	LOC644950	-1.41381	-1.2853	2.69911
ILMN_2227495	ZNF549	-1.02279	1.67302	2.69581
ILMN_3235808	POM121C	-1.79927	-0.89652	2.695794
ILMN_1676986	NPIP	-1.66439	1.02558	2.68997
ILMN_1735779	KCNJ8	-2.26739	-0.42174	2.689133
ILMN_1749915	C1orf63	1.6788	-1.00749	2.68629
ILMN_1668228	LOC136143	-0.20892	2.47706	2.685976
ILMN_1732053	SNRNP70	-2.42384	0.260338	2.684178
ILMN_1666372	ATP5H	-2.58998	-0.09339	2.6833696
ILMN_1733863	FAM100A	-2.62547	-0.05758	2.6830472
ILMN_1653039	LOC642934	1.92929	-0.75178	2.681073
ILMN_1678353	FARP1	-2.44055	0.238645	2.679195
ILMN_1815154	MYH10	-2.29365	-0.38467	2.67832
ILMN_1778010	IL32	1.68795	-0.99021	2.678159
ILMN_1800632	C7orf20	1.85107	-0.82517	2.676242
ILMN_3253787	LOC100128274	-0.72113	1.955	2.676129
ILMN_2059452	SLC12A2	1.64363	-1.03051	2.67414
ILMN_3217522	LOC339970	-0.42217	2.25059	2.672762
ILMN_1707783	CCDC72	-2.30885	0.362065	2.670915
ILMN_1810431	LOC642299	1.66599	-0.99971	2.665703
ILMN_3281651	LOC654350	-0.90017	1.76509	2.665258
ILMN_1734231	DDOST	1.09393	-1.56972	2.66365
ILMN_3306950	CDK10	-2.43752	-0.22444	2.661958
ILMN_1708537	RBPJ	-2.31951	-0.34137	2.660876
ILMN_1806015	LOC391045	-1.4341	-1.22557	2.65967
ILMN_2331890	RPL41	-1.46784	1.18808	2.65592
ILMN_1718136	UQCRHL	-1.77229	0.883162	2.655452
ILMN_1744381	SERPINE1	-1.25021	-1.40465	2.65486
ILMN_3297562	LOC728611	-0.8237	1.831	2.654699
ILMN_3294213	LOC401098	-0.75383	1.89928	2.653113
ILMN_1665538	SKP2	-1.59349	1.05826	2.65175
ILMN_1749081	AUTS2	1.15759	-1.49303	2.65062
ILMN_1746670	C18orf26	-2.45781	-0.19197	2.649784
ILMN_1797342	FNBP1	1.5222	-1.12581	2.64801
ILMN_1684434	SLC17A5	-0.41851	2.22949	2.648002
ILMN_1712673	SASH1	-0.97385	-1.67388	2.647733
ILMN_3257335	LOC100130884	-2.48169	-0.16573	2.647421
ILMN_2066020	TCEB1	-2.21305	-0.43312	2.646167
ILMN_2283325	GPR177	1.7088	-0.93649	2.645285
ILMN_1722858	PPP2CA	1.59485	-1.04939	2.64424
ILMN_1758250	TRAFD1	-1.88202	-0.76125	2.643271
ILMN_1696249	LOC647568	-0.44986	2.19106	2.640924
ILMN_3229128	LOC730978	0.919351	1.72129	2.640641
ILMN_3218721	LOC100131349	-0.42559	2.21484	2.640434
ILMN_1680208	LOC284821	-0.96343	1.67681	2.640239

ILMN_1656837	RBP1	1.62286	-1.01716	2.64002
ILMN_1723743	ROM1	-1.96621	-0.67291	2.63912
ILMN_3241853	RRP7B	1.46737	-1.17051	2.63788
ILMN_3306725	LOC729298	-2.0387	-0.59836	2.637062
ILMN_1748651	PSMB3	1.41271	-1.22432	2.63703
ILMN_3300301	LOC730105	1.25857	-1.3782	2.63677
ILMN_1714108	TP53INP1	-2.25076	-0.38574	2.636496
ILMN_1713964	BTBD3	1.26518	-1.36977	2.63495
ILMN_1708936	EXOSC3	1.83277	-0.80136	2.63413
ILMN_1677691	LOC648852	-0.83277	1.80101	2.63378
ILMN_3235559	CTGLF6	-0.70816	1.92362	2.631778
ILMN_1776995	LOC651192	-0.4213	2.20988	2.631177
ILMN_2408001	RFWD2	1.39401	-1.23441	2.62842
ILMN_1693538	STK36	-1.88465	0.74325	2.6279
ILMN_1809141	ING4	-1.90478	0.721391	2.626171
ILMN_2212999	KIF5C	-2.20573	-0.41979	2.625515
ILMN_1726108	LASS2	1.77795	-0.84693	2.62488
ILMN_3208330	LOC100132797	-1.38358	-1.24026	2.62384
ILMN_1686664	MT2A	-2.19323	0.428761	2.621991
ILMN_2345837	CLTA	1.52745	-1.094	2.62145
ILMN_3245559	CDK2AP1	-2.04849	-0.57296	2.621448
ILMN_1750800	ACO1	-2.50341	0.117662	2.621072
ILMN_2362122	AP3M1	-0.9959	-1.62516	2.621055
ILMN_1798270	C11orf75	1.32207	-1.29847	2.62054
ILMN_1769702	GPAA1	-1.71548	-0.9042	2.619679
ILMN_2154671	COX6B1	-1.745	0.874345	2.619345
ILMN_3205656	LOC391075	1.71413	-0.90423	2.618359
ILMN_3222974	PRKCB	-1.20923	1.40802	2.61725
ILMN_1783394	ATF4	0.935744	1.67934	2.615084
ILMN_1763688	C17orf49	-2.04562	-0.56611	2.611725
ILMN_2219134	RPS15	-0.71866	1.89262	2.611282
ILMN_3195815	LOC100130561	-1.00425	-1.60692	2.61117
ILMN_1706075	SF3B1	-0.85951	1.75165	2.611157
ILMN_2098126	CCL5	-2.25968	-0.3509	2.610579
ILMN_2200562	LOC595101	0.786226	1.82419	2.610416
ILMN_2055165	MRFAP1	1.69527	-0.91142	2.606694
ILMN_1671067	MED13	-1.35628	1.25032	2.6066
ILMN_3246255	LOC100133516	-1.00425	1.60233	2.60658
ILMN_1774584	C2orf28	-2.24742	0.358704	2.606124
ILMN_1745933	LOC400713	-1.01531	1.59053	2.60584
ILMN_1769926	DBN1	-1.96549	0.640132	2.605622
ILMN_2182750	DDX1	-0.23382	2.3695	2.603323
ILMN_1704369	LIMA1	1.55045	-1.05214	2.60259
ILMN_1682636	CXCL2	-1.2226	-1.37955	2.60215
ILMN_2364072	CLCNKA	1.78415	-0.81766	2.601806
ILMN_1711272	SERPING1	-2.0508	0.550702	2.601502
ILMN_2373515	HSP90AA1	1.50111	-1.09742	2.59853
ILMN_1795089	RASAL3	-1.64473	-0.95327	2.597997
ILMN_3187852	KIAA1310	-1.59047	-1.00722	2.59769
ILMN_3275033	LOC646841	0.674903	1.92205	2.596953
ILMN_1732328	LOC646200	-1.9308	0.665376	2.596176
ILMN_1788108	TXNDC5	-2.3375	-0.25749	2.594992

ILMN_1748454	DKFZp434M131	-0.60694	1.98797	2.594911
ILMN_1790953	TBCB	-1.37323	-1.22166	2.59489
ILMN_1709451	TFPT	-0.44684	2.14741	2.59425
ILMN_1749676	KIAA1704	-0.73911	-1.85396	2.593072
ILMN_3279354	LOC402360	-0.55689	2.03513	2.59202
ILMN_2255142	TRIM34	-0.53358	2.05816	2.591735
ILMN_1763091	C14orf43	-1.47445	-1.1167	2.59115
ILMN_1686679	ZNF462	0.818294	-1.77231	2.590604
ILMN_3228529	TMEM191A	-0.82593	1.76434	2.590267
ILMN_2228710	PDCD5	-1.6577	-0.93247	2.590173
ILMN_2344216	STX2	-1.57352	-1.0134	2.58692
ILMN_3234547	LOC100133803	-2.39439	-0.19245	2.586838
ILMN_1679620	LOC728519	-2.06162	0.523481	2.585101
ILMN_1777854	BMS1	-1.94425	0.639381	2.583631
ILMN_1651800	GSTM4	0.745643	1.83751	2.583153
ILMN_1765446	EMP3	-2.44691	-0.13436	2.581272
ILMN_2134555	KCTD3	1.73219	-0.84812	2.58031
ILMN_1763540	PSME4	1.41915	-1.16045	2.5796
ILMN_1737015	RPL39	-0.75789	1.82028	2.578172
ILMN_1761479	ZC3HC1	1.87471	-0.70219	2.576899
ILMN_2402600	GLIS3	-2.34912	-0.22653	2.575646
ILMN_1691849	LIN7C	1.80619	-0.76941	2.575596
ILMN_3238670	SNORA47	-2.44086	-0.13443	2.575287
ILMN_1666564	LOC652489	-1.18058	1.39396	2.57454
ILMN_2112580	FCGR3A	-1.95332	0.620403	2.573723
ILMN_1691959	AKAP2	1.74055	-0.83316	2.57371
ILMN_2175075	SFRS4	-1.7555	0.81778	2.57328
ILMN_1656011	RGS1	-1.22164	1.35159	2.57323
ILMN_1811539	LOC644316	-0.43069	2.1423	2.572987
ILMN_1748438	POLR2G	1.71039	-0.8605	2.570889
ILMN_1675427	NBPF15	1.43554	-1.1346	2.57014
ILMN_3276990	LOC389141	1.39408	-1.17585	2.56993
ILMN_1755733	RPLP2	-2.54136	-0.02797	2.5693262
ILMN_1683859	SLC7A1	-2.01515	0.552252	2.567402
ILMN_1683888	SRP72	1.42719	-1.13985	2.56704
ILMN_2087941	ENTPD3	0.756226	1.81053	2.566756
ILMN_1757660	CAPS	-0.80381	1.76267	2.566475
ILMN_2155998	PSMD6	-2.15464	-0.4103	2.564939
ILMN_1813240	EIF1AX	1.67219	-0.89255	2.564744
ILMN_2306540	PDE9A	1.26725	1.29722	2.56447
ILMN_1746314	EVI5	-0.50533	2.05862	2.563946
ILMN_1684836	AKAP12	-2.10156	-0.46193	2.563489
ILMN_3246247	LOC399491	0.895718	1.66746	2.563178
ILMN_1727815	CFI	-1.89698	-0.66548	2.562457
ILMN_1658768	LOC402643	1.64203	-0.91889	2.560922
ILMN_1758128	CYGB	-1.53779	-1.02291	2.5607
ILMN_2050112	LOC388524	1.57098	-0.98906	2.560041
ILMN_2055310	MBD4	-0.60201	1.95775	2.559758
ILMN_2214144	TWSG1	1.78336	-0.77497	2.558328
ILMN_2352036	RTN4	-0.32279	2.23483	2.557616
ILMN_2311761	AP3S1	1.50461	-1.05146	2.55607
ILMN_2403906	ARFIP1	1.63249	-0.92235	2.554838

ILMN_1783120	SLMAP	1.52084	-1.03273	2.55357
ILMN_3195198	KRT17P3	-0.56556	1.9874	2.552963
ILMN_2049228	NUDT4P1	-0.99239	1.55953	2.551924
ILMN_2229242	LSM3	1.45711	-1.09392	2.55103
ILMN_2119486	DMTF1	-2.3153	-0.23521	2.550513
ILMN_2371590	DDX17	-1.16499	-1.38547	2.55046
ILMN_1685567	TGDS	-1.21353	1.33687	2.5504
ILMN_1764230	GNPTG	1.4875	-1.0618	2.5493
ILMN_1700067	BTN3A2	1.34785	-1.20086	2.54871
ILMN_3232828	LOC728620	0.237409	2.31082	2.548229
ILMN_1720850	BAZ2B	-1.56819	-0.97894	2.547127
ILMN_2206716	JTB	-1.53874	1.0074	2.54614
ILMN_2183938	LEMD3	0.81702	-1.72894	2.54596
ILMN_1751378	RARS	-1.90247	0.642008	2.544478
ILMN_2129234	TMEM47	-1.61092	-0.92979	2.540712
ILMN_3238283	LOC100130123	-1.45623	-1.08283	2.53906
ILMN_1810844	RARRES2	-1.98507	-0.55319	2.538258
ILMN_1748251	LOC653254	-0.43506	2.10257	2.537633
ILMN_1724658	BNIP3	1.87439	-0.66285	2.53724
ILMN_1720858	C6orf115	1.45687	-1.07913	2.536
ILMN_2142554	NENF	1.58196	-0.95402	2.535978
ILMN_1682783	TUG1	-1.28419	-1.2517	2.53589
ILMN_1700695	SLC44A1	0.355735	2.17971	2.535445
ILMN_1774513	DDX17	-1.16809	-1.3642	2.53229
ILMN_2353202	PTK7	-1.85887	-0.67338	2.532247
ILMN_2164242	UBE2F	-1.06823	1.4625	2.53073
ILMN_1747223	FRYL	-0.64713	-1.88223	2.529356
ILMN_2336982	NPTN	1.76307	-0.76604	2.529114
ILMN_1798189	COX7C	-0.99677	1.53227	2.52904
ILMN_3248113	SNRNP70	-2.27607	0.252338	2.528408
ILMN_3233930	LOC390557	1.41167	-1.11616	2.52783
ILMN_3238018	LOC100134372	-1.90215	-0.62274	2.524891
ILMN_3279282	LOC100132364	-0.25388	2.27079	2.524665
ILMN_3208973	LOC653162	1.48965	-1.03494	2.52459
ILMN_1788254	PGAM5	-1.3239	1.20066	2.52456
ILMN_2292696	COX15	-1.61076	-0.91353	2.524285
ILMN_2102580	UTP20	-0.47047	2.0535	2.523973
ILMN_1656940	ABLIM3	1.20979	1.31403	2.52382
ILMN_1804854	CTNNA1	0.547426	1.97622	2.523646
ILMN_1697597	KIAA0494	1.69949	-0.82355	2.52304
ILMN_1657204	SAE1	0.496977	2.02549	2.522467
ILMN_2086952	TOP1P1	-1.70839	0.810756	2.519146
ILMN_1671494	USP5	0.862616	-1.65612	2.518736
ILMN_1685722	EIF4A2	-0.96088	1.55719	2.518073
ILMN_1714170	SPSB1	1.24831	-1.26953	2.51784
ILMN_2352245	RASSF6	-0.63288	1.88441	2.517292
ILMN_1782331	TDG	-1.03871	1.47822	2.51693
ILMN_2125562	MOBK1B	-1.50309	-1.01339	2.51648
ILMN_1665428	GSDMD	1.64116	-0.87446	2.515618
ILMN_3246783	LOC100131330	0.780735	1.73373	2.514465
ILMN_1735199	CIAPIN1	-1.94592	0.568442	2.514362
ILMN_2410145	NR4A1	-1.3609	-1.15276	2.51366

ILMN_2175601	VDAC1	-1.65444	-0.85864	2.513081
ILMN_1795927	PSPC1	-2.50317	0.009397	2.51256656
ILMN_2136010	ADH4	-0.63416	1.87795	2.512105
ILMN_1756874	FBXO42	-1.99573	0.515771	2.511501
ILMN_1795429	VCL	-1.52593	-0.98458	2.510512
ILMN_1771292	LOC163233	1.23693	-1.27155	2.50848
ILMN_3258914	LOC100129652	-1.03958	-1.46851	2.50809
ILMN_1690545	TAF11	-0.50835	1.9997	2.50805
ILMN_1738656	GLOD4	-1.0773	1.42992	2.50722
ILMN_1721349	MAGT1	-0.53596	1.97088	2.506842
ILMN_1708160	KPNA2	-1.2152	1.29125	2.50645
ILMN_1659462	DUSP23	1.85648	-0.64797	2.504453
ILMN_1770035	NCOA5	-2.24106	0.263326	2.504386
ILMN_1700109	PTOV1	-1.14565	-1.35836	2.50401
ILMN_1659952	MTMR2	-2.12114	0.381652	2.502792
ILMN_1733983	LOC653316	-1.99446	0.508066	2.502526
ILMN_1710207	C10orf6	-1.75757	-0.74496	2.502525
ILMN_2059886	TTC38	1.50127	-1.0011	2.50237
ILMN_3260715	CRTC3	1.20422	1.29796	2.50218
ILMN_3257361	FLJ20518	-0.50604	1.99582	2.501863
ILMN_1676893	ADCY3	-0.91855	1.58271	2.50126
ILMN_2374244	DYRK2	-2.24002	-0.26005	2.500069
ILMN_3297644	TMEM214	1.58084	-0.91521	2.496052
ILMN_1737314	BCL6	1.95348	-0.54191	2.495389
ILMN_1691506	NGRN	1.72949	-0.76581	2.495298
ILMN_1785852	OBFC2A	1.43777	1.05721	2.49498
ILMN_1785427	SYNE1	-2.34092	-0.15349	2.494413
ILMN_1755364	RALA	1.37913	-1.11504	2.49417
ILMN_1782086	AOC3	-2.21909	-0.275	2.494092
ILMN_3243390	ZNF506	-0.46761	2.0258	2.493409
ILMN_1712357	HNRPK	-1.74317	0.749439	2.492609
ILMN_1885728	KIAA1147	-1.3523	-1.14027	2.49257
ILMN_1802411	ITGA1	-1.81988	-0.67157	2.491446
ILMN_3268246	LOC100130701	0.344674	2.14476	2.489434
ILMN_1707481	BTBD15	-2.29127	0.197578	2.488848
ILMN_1768743	FIP1L1	0.435228	2.05224	2.487468
ILMN_3239730	LOC100133002	-2.12265	0.364509	2.487159
ILMN_2181540	YY1	1.12091	-1.36617	2.48708
ILMN_1802248	RNF152	1.20136	-1.28496	2.48632
ILMN_1708393	C1orf185	1.57535	-0.91095	2.486295
ILMN_1678004	TMEM41B	1.40117	-1.0847	2.48587
ILMN_1651828	CCT3	-1.44095	-1.04479	2.48574
ILMN_1786388	RNF113A	0.798639	1.68572	2.484359
ILMN_2104877	CMPK1	1.52642	-0.95788	2.484302
ILMN_2189222	KLHL8	-0.79275	-1.69133	2.484075
ILMN_2329309	POGZ	-0.75558	-1.72838	2.483964
ILMN_3184502	LOC100129015	-0.38644	2.09683	2.483274
ILMN_2395204	SLTM	1.58904	-0.89302	2.482055
ILMN_1706305	C14orf112	1.5779	-0.90402	2.481916
ILMN_1773763	MTA2	-1.10746	-1.37417	2.48163
ILMN_1740052	APEG1	-0.52641	1.95514	2.481553
ILMN_1698067	NBPF14	1.32056	-1.16073	2.48129

ILMN_1654118	BCL2L1	-2.21384	-0.26632	2.480161
ILMN_1666409	PSMB6	-2.18742	-0.29131	2.478725
ILMN_3245507	LOC100133627	1.49037	-0.98834	2.47871
ILMN_1700453	LOC642419	1.83007	-0.64822	2.478292
ILMN_1737396	PSMD14	1.07293	-1.40345	2.47638
ILMN_1707434	LOC653778	-1.685	0.790477	2.475477
ILMN_3243578	LOC727899	0.527056	1.94757	2.474626
ILMN_2059606	CCDC117	-1.51606	-0.95814	2.474197
ILMN_1710989	LOC653061	1.39353	-1.07899	2.47252
ILMN_2150654	ZSWIM4	-1.20382	-1.2686	2.47242
ILMN_1686871	PARP1	1.02988	-1.44245	2.47233
ILMN_1699214	LOC647450	-1.35907	-1.11161	2.47068
ILMN_1658926	NOTCH3	-2.21225	-0.2581	2.470352
ILMN_1741688	CPXM2	-0.71604	1.75426	2.470296
ILMN_2400874	SCYL1	-1.07427	1.39589	2.47016
ILMN_1698072	PITRM1	-2.14979	0.320104	2.469894
ILMN_1759991	MGC3731	-1.06083	1.40893	2.46976
ILMN_1651279	LOC645349	-1.53413	0.934165	2.468295
ILMN_1681542	HIST1H4E	1.68731	-0.7809	2.468213
ILMN_1665148	LOC648249	1.37546	-1.09234	2.4678
ILMN_1679700	LOC389672	1.42329	-1.04393	2.46722
ILMN_2188521	PVRL3	0.90097	1.56593	2.4669
ILMN_2116661	PRPSAP2	-1.35819	1.10806	2.46625
ILMN_1681871	FTHL7	-2.31028	-0.15438	2.464661
ILMN_1797974	AIG1	1.10539	-1.35804	2.46343
ILMN_1803988	MCL1	0.429658	2.03323	2.462888
ILMN_1739199	FAM39DP	-1.62659	0.835822	2.462412
ILMN_1680344	MYOM1	-2.33583	-0.12641	2.462239
ILMN_1696407	SFRS2	-2.20453	-0.25662	2.461153
ILMN_1748626	LOC642214	-2.03862	-0.42238	2.460996
ILMN_1747197	SLC41A2	1.105	-1.35578	2.46078
ILMN_1682929	SYTL2	1.19253	-1.26719	2.45972
ILMN_1796926	PABPC5	-1.35183	1.10708	2.45891
ILMN_3283772	LOC644237	1.58729	-0.87148	2.458772
ILMN_1781039	VPS26	1.01261	-1.44608	2.45869
ILMN_1659874	SFRS15	-1.00393	-1.4543	2.45823
ILMN_1712430	ATP5G1	-1.82123	-0.63496	2.456185
ILMN_1653001	CABLES1	1.36504	-1.09042	2.45546
ILMN_2403534	ALOX15	-0.80023	1.65489	2.455115
ILMN_1773236	LOC163233	-0.39766	2.05617	2.453834
ILMN_1696670	LOC92497	0.042535	2.41027	2.4528048
ILMN_3255792	LOC100128505	-0.9242	1.52848	2.45268
ILMN_3269655	FLJ35390	-0.28499	2.16682	2.451808
ILMN_2235785	KCNH6	0.617371	1.83381	2.451181
ILMN_1742277	LOC648064	0.236057	2.21439	2.450447
ILMN_1730906	FILIP1L	-1.11008	-1.33899	2.44907
ILMN_1807206	DHRS1	1.7384	-0.71063	2.449027
ILMN_1803429	CD44	-1.34888	-1.09985	2.44873
ILMN_1680196	LAPTM4B	1.05447	1.39342	2.44789
ILMN_2086417	NDUFV2	-1.99366	0.454218	2.447878
ILMN_3249197	LOC100133588	-2.1513	0.296547	2.447847
ILMN_1742332	KCTD12	-1.79242	-0.65497	2.447394

ILMN_1763147	NDUFB6	1.47406	-0.97228	2.44634
ILMN_2352303	RASSF2	-2.00035	0.44588	2.44623
ILMN_1673950	STBD1	-1.51885	-0.92657	2.445423
ILMN_2203858	OR10J1	0.469684	1.97542	2.445104
ILMN_3286655	LOC646127	-2.36057	0.083264	2.4438341
ILMN_1797236	TGM2	-1.85855	-0.5842	2.442745
ILMN_3244907	LOC100134201	-1.31665	1.12491	2.44156
ILMN_1658885	DAGLB	1.35358	-1.08797	2.44155
ILMN_2227573	GSTO1	-2.08597	-0.355	2.44097
ILMN_1782417	LOC651064	-2.39885	0.041765	2.440615
ILMN_1783675	ASB8	1.23558	-1.20498	2.44056
ILMN_3298582	LOC728873	1.29581	-1.14366	2.43947
ILMN_2113490	NTN4	-1.17271	-1.26675	2.43946
ILMN_1672961	LOC652624	-2.14437	-0.29418	2.438545
ILMN_1694539	MAP3K6	1.50517	-0.93288	2.438051
ILMN_1805512	WDR13	1.31555	-1.12211	2.43766
ILMN_3266775	LOC100130513	-1.88855	0.548944	2.437494
ILMN_1772690	OGFOD1	-1.28634	-1.15052	2.43686
ILMN_2397880	CSTF3	0.524748	-1.91155	2.436298
ILMN_1713178	FAM116A	-0.40578	-2.03001	2.43579
ILMN_2351902	SMN1	0.020413	2.41488	2.4352934
ILMN_1695420	CLTA	-0.49785	1.93634	2.434187
ILMN_1651787	MSH5	-1.59094	-0.84321	2.434148
ILMN_1687707	LOC646795	-1.70704	0.726793	2.433833
ILMN_1698369	LOC728127	1.62763	-0.8062	2.43383
ILMN_1718898	HOXC9	0.501035	-1.93136	2.432395
ILMN_2232121	GPR65	0.50398	-1.92818	2.43216
ILMN_1713995	SCNN1A	1.2156	-1.21611	2.43171
ILMN_1682699	PBX2	-1.67672	0.754392	2.431112
ILMN_3306632	LOC729209	1.18759	-1.24344	2.43103
ILMN_3218820	LOC645387	-0.59962	1.831	2.430621
ILMN_1662618	SQSTM1	-2.10387	-0.32674	2.430609
ILMN_3203724	LOC100131225	-1.69638	0.734155	2.430535
ILMN_1797522	DUSP3	1.21958	-1.21095	2.43053
ILMN_1747102	ZP3	-0.73092	1.69917	2.430086
ILMN_1701169	HP1BP3	1.69376	-0.73627	2.430034
ILMN_1747227	ADORA1	-1.27838	1.15144	2.42982
ILMN_1726839	DCUN1D5	-0.52522	1.90431	2.42953
ILMN_3264100	LOC100130564	-2.31776	-0.11133	2.429091
ILMN_1658800	BRPF3	0.985238	1.44378	2.429018
ILMN_1737484	RTN4R	-2.04491	0.383758	2.428668
ILMN_1726667	LOC648638	-0.39209	2.03611	2.428204
ILMN_2067656	CCND2	-0.91234	1.5156	2.427943
ILMN_1814859	DDX47	-2.29922	0.128515	2.427735
ILMN_1741440	SLC35A1	1.4801	-0.94761	2.42771
ILMN_2194852	FLJ46309	-0.82911	1.59853	2.42764
ILMN_2398403	TCEAL1	1.22141	-1.2059	2.42731
ILMN_1698144	CFH	1.88585	-0.54068	2.426534
ILMN_1670379	ANTXR1	-1.09098	-1.3345	2.42548
ILMN_1659343	BOLA2	1.22642	-1.19724	2.42366
ILMN_1689976	EDIL3	1.07134	-1.35102	2.42236
ILMN_1787324	C16orf48	-1.93406	0.488059	2.422119

ILMN_1652754	ZNF428	0.794183	-1.62745	2.421633
ILMN_1786612	PSME2	-1.15854	-1.26279	2.42133
ILMN_1694983	DDX20	-2.37267	-0.04743	2.4201047
ILMN_1746561	BCL2L2	1.52713	-0.89267	2.419802
ILMN_2361603	NDRG2	1.71317	-0.70653	2.419697
ILMN_2115862	ESPNL	-1.85099	0.568395	2.419385
ILMN_1778374	BSG	0.790682	1.62836	2.419042
ILMN_1670535	NDRG2	1.38661	-1.03241	2.41902
ILMN_1687440	HIPK2	1.77707	-0.64177	2.418836
ILMN_2349600	BRD8	-2.17748	0.241319	2.418799
ILMN_3251550	PHLDA1	1.39202	-1.02661	2.41863
ILMN_2413251	EWSR1	1.26645	-1.15112	2.41757
ILMN_1738217	LOC388237	-0.83587	1.58142	2.417293
ILMN_2278908	GGA1	1.51352	-0.90372	2.41724
ILMN_1742163	LOC441087	-0.97871	1.43821	2.416917
ILMN_3296519	LOC728002	-1.94831	-0.46791	2.416215
ILMN_1728512	YWHAH	1.48257	-0.93307	2.415641
ILMN_2415748	WSB1	1.42122	-0.99433	2.415548
ILMN_1794399	SNRK	0.700207	-1.71436	2.414567
ILMN_2151817	PFN1	-2.12973	0.284416	2.414146
ILMN_1780189	PSMC5	0.723522	-1.69039	2.413912
ILMN_3275590	LOC647302	1.54718	-0.8662	2.413377
ILMN_1693448	LOC643811	1.10834	-1.30441	2.41275
ILMN_3291344	LOC202227	-1.47978	-0.9328	2.412578
ILMN_2379788	HIF1A	0.924126	-1.48748	2.411606
ILMN_3305949	LOC730246	0.758614	1.6526	2.411214
ILMN_2071826	RNF152	1.51766	-0.8931	2.410758
ILMN_3225824	LOC729046	0.513449	-1.89698	2.410429
ILMN_2211780	SLC25A4	1.48679	-0.92341	2.410197
ILMN_1784216	KRTAP19-6	1.54217	-0.8664	2.408574
ILMN_2177090	LOC200030	1.20701	-1.20099	2.408
ILMN_3224126	LOC729222	-1.23278	-1.17491	2.40769
ILMN_1667653	LOC646278	-2.3702	-0.03706	2.4072555
ILMN_1770084	TACC1	0.422178	-1.98453	2.406708
ILMN_2208158	GTF2IP1	-1.25817	-1.14755	2.40572
ILMN_3200830	LOC649553	-1.38732	-1.01815	2.40547
ILMN_3248904	LOC100132062	0.537878	1.86541	2.403288
ILMN_1789283	PPP2R5C	1.53071	-0.87249	2.403198
ILMN_1741617	SCRT1	-1.70314	0.699827	2.402967
ILMN_2073235	FTHL12	-2.05605	-0.34475	2.400801
ILMN_1781764	HNRNPH2	1.31841	-1.08225	2.40066
ILMN_1713990	TRIP6	-0.7898	-1.61048	2.400281
ILMN_3270248	LOC100128172	1.403	-0.99719	2.400192
ILMN_1779648	HIST3H2A	-0.99359	-1.40544	2.399027
ILMN_2128128	SHFM1	-2.24472	-0.15375	2.398468
ILMN_1776777	ADAR	1.63654	-0.76161	2.398152
ILMN_1737561	LOC88523	-1.90558	0.492344	2.397924
ILMN_3282983	LOC100132992	-1.49943	-0.89813	2.397564
ILMN_1712446	LOC653884	1.04898	1.34858	2.39756
ILMN_1698231	RRM2B	-2.21671	-0.18076	2.397471
ILMN_2386732	RCHY1	-0.66511	1.73196	2.397069
ILMN_1665871	ANGEL1	-0.97441	-1.42249	2.3969

ILMN_1663165	LOC652808	1.48711	-0.90895	2.396055
ILMN_1769931	SFPQ	1.45464	-0.94055	2.39519
ILMN_1658847	MGC61598	-1.28976	-1.1051	2.39486
ILMN_2174612	CNOT8	-0.32199	2.07284	2.39483
ILMN_2197659	SLC14A1	-0.69789	1.6966	2.394493
ILMN_1709611	PSMA1	-2.11199	-0.28248	2.394466
ILMN_1762531	FGF9	1.24154	-1.15235	2.39389
ILMN_3289247	LOC400750	1.04054	-1.35316	2.3937
ILMN_3245093	LOC100131986	-0.54885	1.84457	2.393423
ILMN_1770412	AHCYL1	1.15497	-1.23832	2.39329
ILMN_1753111	NAMPT	-1.56269	-0.83043	2.393122
ILMN_1699049	PMS2L3	1.71007	-0.68242	2.392489
ILMN_1673305	RHOC	1.52761	-0.86484	2.392452
ILMN_1791642	LOC91664	1.57464	-0.81746	2.392095
ILMN_2245180	BUB3	0.290485	2.10155	2.392035
ILMN_1651405	BRD9	1.43133	-0.95866	2.389993
ILMN_1731203	RNF213	-0.80134	1.58861	2.389949
ILMN_2410516	PPM1A	1.35589	-1.03342	2.38931
ILMN_1730777	KRT19	-1.07173	1.3172	2.38893
ILMN_1656501	DUSP5	-1.83929	-0.54957	2.388862
ILMN_1797813	SUZ12	1.42806	-0.96	2.388055
ILMN_1778561	WEE1	1.48798	-0.89938	2.387362
ILMN_3240997	ARAP3	0.530716	-1.85608	2.386796
ILMN_2379931	PIGO	1.06569	-1.32076	2.38645
ILMN_3254973	LOC100129112	-1.54002	-0.84626	2.386276
ILMN_1663975	CNNM2	-2.35429	0.030546	2.3848355
ILMN_1706094	HECTD1	1.64625	-0.73842	2.384666
ILMN_1764970	JMJD1C	-1.44898	-0.93563	2.384607
ILMN_1804357	GNG4	-0.86762	-1.51635	2.383973
ILMN_3241234	LOC730278	-1.73935	-0.64431	2.383655
ILMN_3241725	GOLGA9P	-0.34244	2.04044	2.38288
ILMN_2101928	HNRPH1	-1.0368	1.34546	2.38226
ILMN_1753892	LOC654121	-1.48869	-0.89294	2.381628
ILMN_1734149	LOC388122	1.29342	-1.08774	2.38116
ILMN_1761797	CSTB	1.68381	-0.69704	2.380845
ILMN_1720241	TRIP12	1.44151	-0.93856	2.380072
ILMN_1742432	COBRA1	1.53421	0.844296	2.378506
ILMN_1682775	EDN1	-1.13109	-1.24736	2.37845
ILMN_1812995	CTSL1	-2.12973	0.248657	2.378387
ILMN_2096970	MYO3B	-0.37737	2.00067	2.378043
ILMN_1687848	C7	1.31746	-1.06003	2.37749
ILMN_2161832	VPS37A	1.40682	-0.96983	2.37665
ILMN_1681679	TSPO	1.39098	-0.98562	2.376597
ILMN_3275499	LOC644589	1.5888	-0.7876	2.376396
ILMN_3293676	LOC100131387	-0.61148	1.76459	2.376067
ILMN_1808584	FAM36A	1.16117	-1.21394	2.37511
ILMN_2145116	TMEM173	-0.79752	1.57753	2.375049
ILMN_1651776	FHOD1	-1.59166	-0.78321	2.374865
ILMN_1678730	NOMO1	0.814872	-1.5599	2.374772
ILMN_1712936	C21orf66	-0.91425	1.46029	2.374543
ILMN_1651657	ADAM33	-1.60861	-0.76478	2.373394
ILMN_2365595	GMPR2	1.58777	-0.78504	2.372809

ILMN_3228294	LOC729279	-1.17732	1.19494	2.37226
ILMN_1765829	NUFIP2	1.44438	-0.92762	2.371995
ILMN_1771048	LOC728153	1.32716	-1.04462	2.37178
ILMN_1680770	UBQLN2	-0.85712	1.5146	2.371719
ILMN_2206307	TLL2	-2.07515	0.296435	2.371585
ILMN_3230442	LOC728210	-2.06448	0.306873	2.371353
ILMN_2262044	PARP10	1.60241	-0.76813	2.370537
ILMN_2383693	UPF2	0.848213	-1.52232	2.370533
ILMN_1683747	CD276	-2.02677	-0.34348	2.370249
ILMN_1713892	C4orf34	1.2382	-1.13129	2.36949
ILMN_1805646	SS18	1.32693	-1.04157	2.3685
ILMN_3237946	LOC100134134	-0.8868	-1.48167	2.36847
ILMN_1681590	LARP1	0.189109	2.17922	2.368329
ILMN_2198376	PSMA4	-1.00218	-1.36496	2.36714
ILMN_1752579	ATP6V0A1	1.01858	-1.34814	2.36672
ILMN_1719985	FEM1A	1.39894	-0.96719	2.366125
ILMN_3243069	LOC100133692	1.209	-1.15677	2.36577
ILMN_3219764	LOC441506	1.31881	-1.04677	2.36558
ILMN_1812392	TMSB10	-1.55991	0.80488	2.36479
ILMN_1768784	PSMC2	-0.65938	-1.70508	2.36446
ILMN_3283680	LOC345041	-2.05255	-0.31175	2.3643
ILMN_1701731	AKR1B1	1.17781	-1.18547	2.36328
ILMN_3244700	COX16	-1.19865	1.1643	2.36295
ILMN_3239014	LOC100134598	-0.86619	1.49651	2.362701
ILMN_2134381	C14orf85	-1.15767	1.20502	2.36269
ILMN_1761113	GNL2	1.14574	-1.21652	2.36226
ILMN_1705985	PIGA	-1.00855	1.35346	2.36201
ILMN_3310785	MIR1299	1.89587	-0.46505	2.360923
ILMN_1853824	MGAT3	1.39751	-0.96275	2.360262
ILMN_2406532	F11R	-0.44167	1.91781	2.359478
ILMN_1808777	EHD2	0.912588	-1.44652	2.359108
ILMN_2412192	CFH	1.61816	-0.74087	2.359026
ILMN_2383305	GPATCH4	-0.80484	1.55415	2.35899
ILMN_1666894	CSPG4	-1.11422	-1.24391	2.35813
ILMN_1705384	PPFIBP1	-0.73354	-1.6226	2.356142
ILMN_2364828	OGT	1.04253	-1.31304	2.35557
ILMN_2373266	SFRS12	1.54026	-0.81481	2.35507
ILMN_1790249	F8A1	1.02829	-1.32646	2.35475
ILMN_1747205	JDP2	1.53827	-0.81636	2.354631
ILMN_1776005	OSTC	-1.91369	0.440755	2.354445
ILMN_1682288	LOC728565	1.3574	-0.99699	2.354385
ILMN_3251545	CHMP5	-1.38986	0.964403	2.354263
ILMN_1712944	AES	-1.06473	1.28953	2.35426
ILMN_1752451	CTSH	0.741744	-1.61232	2.354064
ILMN_1805415	PDE3A	1.47843	-0.87499	2.353421
ILMN_1700955	TCTEX1D2	1.62676	-0.72646	2.353217
ILMN_1719949	LOC644019	-0.36416	1.9885	2.352664
ILMN_1669113	ATF5	-0.83826	-1.51417	2.352431
ILMN_1677919	GMPR2	1.84908	-0.50271	2.351792
ILMN_1675542	LOC729148	1.63885	-0.71251	2.351359
ILMN_1710815	FBLN1	1.26812	-1.08286	2.35098
ILMN_1694491	CCNG1	-2.15536	0.195583	2.350943

ILMN_3225244	TSEN15	0.373002	-1.97763	2.350632
ILMN_1761946	PROM2	1.46952	-0.88054	2.350061
ILMN_3236021	LOC100133923	0.815668	-1.53388	2.349548
ILMN_2246894	EDF1	1.00442	1.34478	2.3492
ILMN_2195462	C1QTNF4	1.62819	0.720734	2.348924
ILMN_3243253	LPAR3	-1.7886	0.56011	2.34871
ILMN_1795876	GPS1	-1.31069	1.03758	2.34827
ILMN_1674522	HIGD1A	1.63694	-0.7111	2.348035
ILMN_1666924	PINK1	1.089	-1.25744	2.34644
ILMN_1796629	EDNRA	-2.13522	-0.21089	2.346107
ILMN_1726169	EDF1	0.393691	1.95193	2.345621
ILMN_1760714	RPS3	0.10683	2.23864	2.34547
ILMN_1747630	DEK	1.02232	-1.32205	2.34437
ILMN_1717219	C7orf70	1.45647	-0.8877	2.344171
ILMN_2079285	ATP5L	1.35143	-0.99134	2.34277
ILMN_1670028	LPIN2	-1.28809	1.05443	2.34252
ILMN_2410929	PAPSS2	0.993832	-1.34867	2.342502
ILMN_2228809	YWHAG	0.639015	-1.70342	2.342435
ILMN_1760441	MRPS5	-0.39416	1.9477	2.341863
ILMN_1761804	ALDH9A1	-0.72988	-1.61081	2.340692
ILMN_1683017	LOC646853	1.42456	-0.91601	2.340565
ILMN_3245066	DENND4B	1.67816	-0.66229	2.340454
ILMN_1712950	PFN1	-1.99836	-0.34199	2.340354
ILMN_2298511	BOLA2	1.28579	-1.0545	2.34029
ILMN_2234515	IMAA	0.962241	-1.37721	2.339451
ILMN_1768110	ZAK	1.641	-0.69821	2.339207
ILMN_1792997	NPTN	1.30154	-1.03693	2.33847
ILMN_1812787	LOC653717	1.33775	-1.00025	2.338
ILMN_2071937	ATP6V0E1	-1.80404	-0.53349	2.337528
ILMN_2356991	CD47	0.670765	-1.66615	2.336915
ILMN_1685156	ADCY6	-0.57137	1.76536	2.336732
ILMN_1700584	IER2	0.99049	-1.34494	2.33543
ILMN_1783735	LOC649639	1.32024	-1.0148	2.33504
ILMN_2213558	TMED10P	1.00823	-1.32564	2.33387
ILMN_1776464	PARP4	1.53445	-0.79898	2.333431
ILMN_1752520	SLFN11	-1.32533	-1.00793	2.33326
ILMN_2311989	CUTA	1.41111	-0.92103	2.332144
ILMN_2359287	ITGA6	0.972586	-1.35864	2.331226
ILMN_1710209	MFSD6	1.03911	-1.29183	2.33094
ILMN_2102515	PGAM4	1.27114	-1.05942	2.33056
ILMN_3181480	FLJ36131	-0.38414	1.94639	2.330527
ILMN_1708328	RAB11FIP3	1.62644	-0.70382	2.330262
ILMN_1667551	GUSBL1	1.15306	-1.17701	2.33007
ILMN_1706521	CSNK1G2	1.66479	-0.66343	2.32822
ILMN_1671048	ZNF644	1.13571	1.19213	2.32784
ILMN_1703531	S1PR3	-1.43848	-0.88924	2.32772
ILMN_1776838	LOC728895	1.76132	-0.5655	2.326822
ILMN_2237428	SCD5	-1.48439	-0.84211	2.326503
ILMN_1741133	NME1	0.520053	-1.80508	2.325133
ILMN_1663416	LOC642250	-0.63209	1.6926	2.324686
ILMN_1716153	LOC342892	-0.3843	1.93929	2.323586
ILMN_2156982	IMP4	1.57074	-0.75137	2.322109

ILMN_1703100	LOC284701	-0.67466	-1.64706	2.321718
ILMN_1752947	C17orf79	1.0438	-1.27708	2.32088
ILMN_1699489	TUBB6	-0.9585	-1.3621	2.320596
ILMN_1746883	SAT2	-0.40061	1.91998	2.320588
ILMN_1802157	THOC1	-0.25913	-2.06056	2.319687
ILMN_1711311	PODXL	-1.17414	-1.14538	2.31952
ILMN_2180677	ARMCX1	0.920625	-1.39873	2.319355
ILMN_2093674	GMFB	1.32247	-0.99684	2.319307
ILMN_1737862	PSMB4	1.24632	1.07291	2.31923
ILMN_1665455	DCUN1D3	0.36592	-1.95314	2.31906
ILMN_1784410	UBP1	1.35915	-0.95949	2.318642
ILMN_3273491	LOC100130332	1.20176	-1.11675	2.31851
ILMN_1800261	TUBA1B	0.546074	-1.77167	2.317744
ILMN_3237826	FLJ37512	1.62007	-0.69745	2.317519
ILMN_1740441	CYB5R3	0.952533	-1.36473	2.317263
ILMN_1703288	C19orf6	0.742381	-1.57413	2.316511
ILMN_1748904	WTAP	-1.17478	-1.14155	2.31633
ILMN_2358626	ADK	0.620077	1.69589	2.315967
ILMN_1784286	NDUFA1	-1.43434	0.880925	2.315265
ILMN_1751120	HIST1H4H	1.0773	-1.23792	2.31522
ILMN_2335072	TAF1C	-2.07674	0.23742	2.31416
ILMN_1653718	ZFAND2B	1.17669	-1.13643	2.31312
ILMN_1700681	CD99L2	1.16189	-1.15012	2.31201
ILMN_1733615	MTF2	1.16659	-1.14541	2.312
ILMN_2337655	WARS	-0.80285	-1.50889	2.311741
ILMN_1713752	SERINC3	1.13014	-1.18144	2.31158
ILMN_1764851	TP53RK	-1.55633	0.754801	2.311131
ILMN_1773885	DMAP1	0.176616	2.13427	2.310886
ILMN_1764721	RPL8	-0.99239	1.31814	2.310534
ILMN_1788384	C9orf5	1.22977	-1.08035	2.31012
ILMN_1721989	ATP5F1	1.30934	-1.00068	2.31002
ILMN_1705310	VEZF1	1.45886	-0.85046	2.309323
ILMN_1663618	STAT3	1.13181	-1.17638	2.30819
ILMN_2367300	ANKRD5	-0.59039	1.71777	2.30816
ILMN_1794392	DDX3X	-1.02828	1.27982	2.3081
ILMN_3245390	LOC392635	0.311333	1.99543	2.306763
ILMN_1654324	HEYL	-1.16053	-1.14607	2.3066
ILMN_3298107	LOC728927	-2.24957	0.056695	2.3062648
ILMN_1676938	LOC649214	-1.32199	-0.98417	2.306164
ILMN_1677273	TH	-1.76998	-0.53578	2.305759
ILMN_1811592	ARHGAP21	1.65906	-0.64663	2.30569
ILMN_2157510	BLOC1S1	-2.05684	-0.24869	2.305533
ILMN_2285112	FBXO11	1.21202	-1.09227	2.30429
ILMN_1806778	UBE2E1	0.784157	-1.52001	2.304167
ILMN_2149782	RAMP2	-0.94362	-1.36017	2.303785
ILMN_1751956	MGST3	1.08208	-1.22126	2.30334
ILMN_1707156	LRRFIP2	1.76633	-0.53663	2.302961
ILMN_2280441	PACRG	-0.51392	1.78875	2.30267
ILMN_3198247	LOC388707	0.937653	-1.36497	2.302623
ILMN_2157544	GBF1	-0.23144	2.07104	2.302476
ILMN_1772703	OTUD6B	0.525544	1.77638	2.301924
ILMN_1678165	LSM7	-0.44907	1.85156	2.300628

ILMN_2086077	JUNB	0.749542	-1.551	2.300542
ILMN_2389876	TGFB1I1	-1.42018	-0.87995	2.30013
ILMN_2389582	HNRNPL	-1.42193	-0.87759	2.299524
ILMN_2353633	EMR2	-1.0613	1.23753	2.29883
ILMN_1742618	XAF1	1.41493	-0.8838	2.298732
ILMN_1692164	LOC646864	-1.14223	1.15606	2.29829
ILMN_1810910	CFH	1.7797	-0.51783	2.297531
ILMN_1812618	ARAP3	-0.84622	-1.45038	2.296598
ILMN_2056606	PPP1R1A	0.656521	-1.63892	2.295441
ILMN_2317348	APTX	1.02884	-1.26656	2.2954
ILMN_3178302	FNDC3B	0.902323	-1.39299	2.295313
ILMN_1754842	DLGAP4	0.956114	-1.33876	2.294874
ILMN_1806818	MCM3	0.6418	1.65293	2.29473
ILMN_1754811	FBXO38	-2.12337	0.170286	2.293656
ILMN_3245074	LOC646916	-0.38812	1.9054	2.293515
ILMN_1808196	GSTO1	-0.6301	-1.6632	2.293297
ILMN_2081162	SH3BGRL	-0.43172	-1.86104	2.292761
ILMN_2105983	XRCC5	1.27154	-1.02088	2.29242
ILMN_1651354	SPP1	-1.20804	-1.08432	2.29236
ILMN_3231596	PLXNA4	-0.51814	1.77411	2.292248
ILMN_3243084	LOC100134793	0.787737	-1.50351	2.291247
ILMN_2388070	TMEM44	-1.29843	-0.99243	2.290864
ILMN_3190972	LOC100130291	1.0103	-1.28045	2.29075
ILMN_3209220	LOC100131210	0.573129	-1.71733	2.290459
ILMN_1781345	RAD52	-1.22363	1.06672	2.29035
ILMN_1744649	PSMB5	-2.0531	-0.23713	2.290225
ILMN_1758798	LOC728734	1.60034	-0.68964	2.289984
ILMN_2292592	PCSK6	-1.34403	-0.94562	2.289646
ILMN_2241775	TROVE2	0.997174	-1.29186	2.289034
ILMN_1691181	TMX1	0.516632	1.77225	2.288882
ILMN_1674559	FLJ44379	-0.69662	-1.59207	2.28869
ILMN_1651574	AQP3	0.708801	-1.57987	2.288671
ILMN_2097793	ZBTB4	-0.9772	1.31029	2.287485
ILMN_2320964	ADAR	1.53063	-0.75662	2.287254
ILMN_3249618	LOC100133477	1.27409	-1.01302	2.28711
ILMN_1815107	MATR3	0.962958	1.32353	2.286488
ILMN_1726327	AMY1B	1.24536	-1.04103	2.28639
ILMN_1655690	CDR1	-1.13706	1.14846	2.28552
ILMN_1778505	LOC642771	-1.9378	0.347603	2.285403
ILMN_1655195	SMA4	0.966857	-1.31801	2.284867
ILMN_1653601	LOC646144	-0.7154	-1.56926	2.28466
ILMN_2061979	LOC440093	-2.23238	0.05099	2.2833703
ILMN_3237839	ZNF674	-1.62309	0.659962	2.283052
ILMN_1678754	PFDN2	-1.0165	1.26529	2.28179
ILMN_1785765	TM9SF2	-1.17247	-1.10922	2.28169
ILMN_1660858	RIN1	1.12998	-1.15158	2.28156
ILMN_1745447	HIVEP2	1.29669	-0.98401	2.280698
ILMN_1742250	CCNH	-1.21011	-1.06998	2.28009
ILMN_3261345	LOC100130053	-0.70872	-1.57129	2.280005
ILMN_3236704	LOC100133420	-1.03711	1.24271	2.27982
ILMN_3269835	C17orf86	1.0668	-1.21295	2.27975
ILMN_1743049	PWP1	-1.68715	0.592591	2.279741

ILMN_1666178	TP53I13	1.30878	-0.97068	2.279456
ILMN_1743535	PIP5K3	-1.79855	-0.48043	2.278983
ILMN_1721869	LOC645600	1.24401	-1.03497	2.27898
ILMN_1747525	LOC650780	-2.12981	0.148688	2.278498
ILMN_2185264	ZNF461	-0.72455	1.55351	2.278061
ILMN_2231242	HMGB1	1.27337	-1.00461	2.27798
ILMN_3236122	LOC100134363	-0.33074	1.94713	2.277873
ILMN_2041222	FLJ40504	0.93089	-1.34693	2.27782
ILMN_1803376	AEBP2	-2.21082	0.066914	2.2777342
ILMN_1779674	PTPRG	1.06433	-1.21245	2.27678
ILMN_1743829	ATXN2	-1.67131	-0.60472	2.276033
ILMN_3242551	LOC100130707	-1.10459	1.17112	2.27571
ILMN_1702124	LNX2	-1.31443	0.961178	2.275608
ILMN_1741459	CDK10	0.880122	-1.3952	2.275322
ILMN_3217172	LOC286444	0.686043	1.58906	2.275103
ILMN_1728934	PRC1	0.70705	1.56737	2.27442
ILMN_1699476	RPE	0.781929	-1.49243	2.274359
ILMN_1657683	C1orf198	-2.05374	0.219295	2.273035
ILMN_2088234	GTF2IRD2P	-0.75224	1.51957	2.271812
ILMN_1733757	LOC374395	1.44796	-0.82383	2.271794
ILMN_2101885	TUBB	-1.66558	-0.60599	2.271569
ILMN_1753182	COL20A1	-0.83945	1.43153	2.270984
ILMN_2259633	MLL5	0.855215	-1.41567	2.270885
ILMN_1774659	LOC652388	1.06195	-1.20876	2.27071
ILMN_1671320	LOC642156	1.35462	-0.91607	2.27069
ILMN_1770338	TM4SF1	-0.78527	-1.48422	2.269485
ILMN_1688515	ZNF195	0.581006	1.68724	2.268246
ILMN_1654445	LOC347292	0.727501	1.54066	2.268161
ILMN_3307930	RAN	-1.35978	-0.90821	2.267991
ILMN_3251587	LOC100008589	-1.37148	-0.8965	2.267975
ILMN_2110281	UFC1	0.813599	-1.45369	2.267289
ILMN_1693060	VEGFA	-1.43745	-0.8298	2.267249
ILMN_1671221	GAPVD1	0.475413	-1.7914	2.266813
ILMN_1756152	MFSD11	-0.6593	1.60742	2.266721
ILMN_1716679	FAIM	1.30711	-0.95848	2.26559
ILMN_1662358	MX1	1.39528	-0.87017	2.265448
ILMN_1676002	QRICH1	1.59047	-0.67488	2.26535
ILMN_2284744	HNMT	0.430295	-1.83496	2.265255
ILMN_3255765	LOC100128559	1.36743	-0.89761	2.265037
ILMN_1750893	LOC441081	1.20351	-1.06148	2.26499
ILMN_1676005	KPNA1	1.00235	-1.26222	2.26457
ILMN_1724017	LOC732450	-1.73322	-0.53124	2.26446
ILMN_1669484	WDR6	-1.83945	-0.42423	2.263679
ILMN_3245600	LRRC37B2	-0.33146	1.93184	2.263299
ILMN_3242111	LOC641784	0.375946	-1.88634	2.262286
ILMN_1651395	LOC644897	-2.10148	0.160747	2.262227
ILMN_1669599	DENND4C	1.11789	-1.14429	2.26218
ILMN_1673529	AGXT2L2	1.26215	-0.99913	2.261277
ILMN_2373830	ATN1	-0.81503	-1.44608	2.261105
ILMN_1698680	ARL17P1	0.679359	1.58118	2.260539
ILMN_1771618	FLJ37396	-0.30735	1.95319	2.260538
ILMN_2212823	ZNF577	-0.71747	1.54283	2.260298

ILMN_1686416	GRIP2	-0.55888	1.70123	2.260109
ILMN_1658802	KRTCAP2	1.69614	-0.56368	2.259819
ILMN_1796206	LOC642678	0.496579	1.76305	2.259629
ILMN_1715133	IVNS1ABP	0.570025	-1.68909	2.259115
ILMN_1687978	PHLDA1	0.741903	-1.51712	2.259023
ILMN_1659923	GNAQ	1.89365	-0.36513	2.258775
ILMN_1675462	LSM6	1.11717	-1.14107	2.25824
ILMN_1660426	CPSF4	0.739516	-1.51869	2.258206
ILMN_1692342	CDC42BPG	1.19086	-1.06734	2.2582
ILMN_1682098	PSMA4	0.779701	-1.47838	2.258081
ILMN_2316918	PANK1	1.50215	-0.75578	2.257934
ILMN_1658464	GTF3A	1.45018	-0.80671	2.256889
ILMN_3260070	LOC100128266	-0.56286	-1.69365	2.256508
ILMN_3242485	LOC100134651	-0.63495	-1.62086	2.255811
ILMN_2202967	ARL15	0.997492	-1.25823	2.255722
ILMN_1659405	LOC653773	0.265817	1.98929	2.255107
ILMN_1714527	VAMP3	-0.44159	-1.81333	2.254918
ILMN_1707858	H2AFZ	1.0251	-1.22937	2.25447
ILMN_1666819	PHLDB1	-1.64951	-0.60448	2.25399
ILMN_1807919	TNS1	0.860308	-1.39366	2.253968
ILMN_1654340	UGP2	1.36934	-0.88441	2.253751
ILMN_1769309	LOC654192	-1.1591	-1.09458	2.25368
ILMN_1656016	DHX9	-1.70967	0.543506	2.253176
ILMN_2190084	VAMP8	-2.19315	0.059168	2.2523183
ILMN_1738239	RBM6	1.15171	-1.1004	2.25211
ILMN_1702835	SH3BGRL	-0.89929	-1.35193	2.251223
ILMN_1775762	GNAI2	-0.9394	-1.31159	2.250988
ILMN_1738909	TROVE2	-1.84136	-0.40788	2.249239
ILMN_1764770	MGC15763	0.94768	-1.30127	2.24895
ILMN_2224143	MCM3	0.700923	-1.54798	2.248903
ILMN_1726603	ATP5I	-0.68516	-1.56374	2.248902
ILMN_1658405	LOC100101266	0.884419	-1.36444	2.248859
ILMN_3236481	LOC100129086	1.67681	-0.57033	2.247135
ILMN_2371700	UCHL5IP	1.41358	-0.83301	2.246586
ILMN_1764500	C3orf10	1.0041	-1.24222	2.24632
ILMN_1781472	CDC42BPA	-0.81065	-1.4356	2.246249
ILMN_1780582	CD83	1.55323	-0.69293	2.246164
ILMN_3215206	LOC100133836	1.31276	-0.93298	2.245736
ILMN_1668507	DDAH1	-0.7294	-1.51631	2.245714
ILMN_3297898	LOC729769	-1.21019	1.03486	2.24505
ILMN_1814823	FTL	-2.08016	0.164659	2.244819
ILMN_1790757	ADSL	1.22197	-1.02279	2.24476
ILMN_1781251	LOC653115	0.954921	-1.28964	2.244561
ILMN_1746525	FTHL2	-0.69304	1.5504	2.24344
ILMN_3237579	ZNF845	1.51901	-0.7244	2.243407
ILMN_1806486	LOC389137	-2.1762	-0.06696	2.2431564
ILMN_1733035	FAM128A	0.203511	2.03882	2.242331
ILMN_2190851	PPID	-0.33783	1.90448	2.242305
ILMN_2316974	LILRB1	-0.34928	1.89289	2.242173
ILMN_1768181	TOR3A	0.449233	1.7926	2.241833
ILMN_3248890	SNORA24	-1.24854	-0.99291	2.241454
ILMN_3273340	LOC100130776	-0.13412	2.10667	2.240788

ILMN_3288717	LOC392437	-2.13658	-0.10379	2.240373
ILMN_3244286	TANC2	-1.16228	1.07808	2.24036
ILMN_1735762	NPNT	0.567797	1.67181	2.239607
ILMN_1772540	ATMIN	1.29295	-0.94641	2.239364
ILMN_1669366	IFT88	0.741108	-1.49718	2.238288
ILMN_1748374	LOC400304	0.744688	-1.4936	2.238288
ILMN_2123415	BMP8B	-0.25284	1.98478	2.237621
ILMN_1716224	STARD4	-0.35111	-1.88624	2.237354
ILMN_3194508	ASAP2	1.01078	-1.22626	2.23704
ILMN_1715635	ATP6V0E1	-1.89674	-0.33999	2.236728
ILMN_2166093	PIK3C2A	1.55466	-0.68186	2.236523
ILMN_1718520	C10orf59	-0.4665	-1.76966	2.236155
ILMN_1692535	DPP4	0.75551	-1.48023	2.23574
ILMN_2204909	XRCC2	-0.417	1.81775	2.23475
ILMN_1697793	SYNJ2BP	-0.8241	1.41038	2.234477
ILMN_1756469	GAMT	0.876223	-1.35803	2.234253
ILMN_1774229	SLC7A4	-1.97353	-0.26022	2.233745
ILMN_3224934	SFRS18	-0.59477	-1.63896	2.233727
ILMN_1730612	DBNDD2	-0.61625	1.61709	2.233341
ILMN_3246315	LOC100133697	1.15282	-1.08028	2.2331
ILMN_1727789	RNF12	1.09473	-1.13768	2.23241
ILMN_1739263	LOC643284	0.830707	1.40125	2.231957
ILMN_1712577	FAM174A	1.32175	-0.90973	2.231476
ILMN_3236377	C2orf69	-0.70999	1.52123	2.231219
ILMN_3209676	LOC644315	0.835163	-1.39604	2.231203
ILMN_3301878	LOC729681	1.42576	-0.80467	2.230434
ILMN_2106994	RABIF	-1.77348	0.45691	2.23039
ILMN_3230435	LOC729086	0.552042	-1.67763	2.229672
ILMN_1683063	PDE9A	-0.10229	2.12708	2.229368
ILMN_3228639	LOC728324	-0.48671	-1.74255	2.229256
ILMN_1798659	CCDC28A	-0.31387	1.91444	2.228313
ILMN_2341815	TFG	0.718509	-1.50867	2.227179
ILMN_1799600	STARD8	1.05486	-1.17224	2.2271
ILMN_3296663	LOC727900	0.51512	-1.71193	2.22705
ILMN_3236468	TMEM14E	1.53175	-0.69459	2.226341
ILMN_2183510	ARMET	0.381676	-1.84457	2.226246
ILMN_2277252	PPFIBP1	-0.71031	1.51471	2.225017
ILMN_1718558	PARP12	1.40626	-0.81809	2.224354
ILMN_3205364	LOC400389	0.51878	-1.70504	2.22382
ILMN_1706118	HN1L	0.951419	-1.27225	2.223669
ILMN_3243782	LOC100133939	0.959615	-1.26394	2.223555
ILMN_1716465	RBP7	-0.40514	-1.81782	2.222964
ILMN_3182000	FLJ10088	0.925319	-1.29692	2.222239
ILMN_1768449	PRPSAP1	1.31706	-0.90512	2.222176
ILMN_2150284	RNPC3	1.31801	-0.90393	2.221943
ILMN_1691431	FAM18B	0.777632	1.44401	2.221642
ILMN_1674551	SMAD5	-1.53986	-0.68171	2.221569
ILMN_1663390	CDC20	-0.69964	1.52139	2.221034
ILMN_3263393	LOC100128202	-0.62365	1.59721	2.220862
ILMN_3243664	LOC440353	0.894206	-1.32628	2.220486
ILMN_3248957	CBWD7	-1.95809	0.262362	2.220452
ILMN_1781457	TIPRL	1.58745	-0.63253	2.219979

ILMN_1768396	AURKAIP1	1.72909	-0.49042	2.219511
ILMN_1814074	PHKA2	1.06998	-1.14948	2.21946
ILMN_2394305	PDGFB	-1.80603	-0.41255	2.218578
ILMN_1725175	FOSL2	0.838983	-1.379	2.217983
ILMN_2246083	C7orf28B	1.01261	-1.20507	2.21768
ILMN_3251733	C19orf43	-1.08549	1.13163	2.21712
ILMN_1679754	ADRA1B	0.795058	-1.42173	2.216788
ILMN_1758626	IDS	-0.43172	1.78388	2.215601
ILMN_1652790	CLK1	0.26152	-1.95383	2.21535
ILMN_1766010	YARS	0.953727	-1.26136	2.215087
ILMN_1802358	LOC642787	0.751532	1.46345	2.214982
ILMN_2362439	C19orf12	-0.61864	1.59487	2.213509
ILMN_1757644	UBE2H	0.893888	-1.31848	2.212368
ILMN_3259081	LOC100128477	-1.92046	0.29173	2.21219
ILMN_1665203	SUMO4	0.974973	-1.23646	2.211433
ILMN_1781514	PCDH17	-1.35986	-0.85149	2.211352
ILMN_1655990	CDK5RAP2	1.0703	-1.14068	2.21098
ILMN_1660669	LOC653073	0.875745	-1.33455	2.210295
ILMN_2087692	CYBRD1	-1.88831	0.321868	2.210178
ILMN_1671583	MKRN1	-1.73903	0.47084	2.20987
ILMN_1662427	PTP4A3	-2.0839	0.124964	2.208864
ILMN_2205882	NSUN7	1.62524	-0.58295	2.208187
ILMN_1653599	ATP5D	-0.46005	-1.7481	2.208149
ILMN_1742547	NRP1	-2.05987	-0.14609	2.20596
ILMN_3224860	LOC730004	-1.12401	1.08164	2.20565
ILMN_2059535	PPM1F	-0.85911	-1.34591	2.205019
ILMN_2184049	COX7B	1.01269	-1.19137	2.20406
ILMN_1810420	DYSF	0.245765	-1.95746	2.203225
ILMN_1715680	NEIL2	-1.69128	-0.51177	2.203051
ILMN_3278683	LOC100133176	-1.09226	1.11063	2.20289
ILMN_1734602	SRRM2	1.11749	-1.0853	2.20279
ILMN_1718988	DAZAP2	-1.23541	0.967367	2.202777
ILMN_1710885	LOC387867	1.11606	-1.08663	2.20269
ILMN_1739081	ABI1	0.858319	1.34405	2.202369
ILMN_2041327	MRPL37	1.34897	-0.85295	2.201918
ILMN_3219643	LOC401537	1.24719	-0.95461	2.2018
ILMN_2371379	ACLY	0.986272	-1.21548	2.201752
ILMN_1700419	HSPC171	0.686918	-1.51482	2.201738
ILMN_2410362	ACBD5	-0.44079	1.76015	2.200943
ILMN_1812067	RER1	-1.1178	1.08274	2.20054
ILMN_1703427	SON	-0.31228	-1.88822	2.200502
ILMN_1758356	OR2W5	1.49363	-0.70604	2.199671
ILMN_2152257	SSTR2	-0.3644	1.83512	2.199522
ILMN_1703852	EFNB2	0.469127	-1.73031	2.199437
ILMN_3265797	LOC100130561	-0.63471	-1.56442	2.199132
ILMN_1774375	LOC284422	1.26764	-0.93131	2.198953
ILMN_2154603	CCDC16	1.28515	-0.9138	2.198953
ILMN_3245707	RIMKLB	-1.61346	0.585319	2.198779
ILMN_1810289	FER1L3	1.05232	-1.14526	2.19758
ILMN_1690566	RASSF4	1.84908	-0.34836	2.197441
ILMN_3176532	LOC100130441	-0.4501	1.74732	2.197423
ILMN_1769810	ARL6IP5	-0.72479	-1.4724	2.197189

ILMN_2271336	Mar-02	1.53859	-0.65831	2.196901
ILMN_2055477	EXOSC7	0.675778	-1.52061	2.196388
ILMN_1806474	LOC727732	-0.91736	-1.27863	2.195986
ILMN_1765858	CAB39	-0.53389	1.66195	2.195843
ILMN_1725534	ACTN4	0.598433	-1.59712	2.195553
ILMN_1790114	LOC474170	0.738243	1.45682	2.195063
ILMN_3236765	UPLP	-0.80866	-1.38631	2.194969
ILMN_2151277	LPGAT1	-1.31753	-0.87665	2.194178
ILMN_2415144	SP110	1.51909	-0.67451	2.193597
ILMN_2340052	NCOR2	0.583951	-1.60844	2.192391
ILMN_1655613	GSTA2	0.723681	-1.46871	2.192391
ILMN_2301624	MACF1	0.955557	-1.23677	2.192327
ILMN_3235647	SIK1	1.63734	-0.55487	2.192208
ILMN_1662895	C14orf153	-1.24018	-0.95186	2.192044
ILMN_1703330	FEM1C	1.72797	-0.46395	2.191922
ILMN_3248536	LOC100134524	0.872881	1.31904	2.191921
ILMN_1750352	C14orf78	1.45345	-0.73842	2.191872
ILMN_3190570	LOC100128648	-1.22013	0.97164	2.19177
ILMN_1676759	DDX27	0.74429	-1.4466	2.19089
ILMN_1667500	LOC653086	1.24115	-0.94971	2.19086
ILMN_1766221	B4GALT1	0.369262	1.82134	2.190602
ILMN_1788387	UGCGL2	-1.67537	0.514522	2.189892
ILMN_3256004	LOC100130003	-1.59238	-0.59744	2.189824
ILMN_1769705	LOC440093	-0.3426	-1.8472	2.189799
ILMN_1764794	PSMB2	-0.83404	-1.3555	2.189543
ILMN_1813604	NDUFB7	-1.10276	-1.08667	2.18943
ILMN_1773238	XCR1	-0.88139	1.30793	2.189319
ILMN_3188005	LOC100127913	-1.18432	1.00499	2.18931
ILMN_1768533	CACNA2D1	0.640368	-1.54893	2.189298
ILMN_1773080	OAZ1	-2.17899	0.010113	2.1891026
ILMN_1716862	PPM1B	-0.37021	-1.81875	2.188961
ILMN_1810922	PCNT	-1.93422	-0.25421	2.188428
ILMN_1701058	TATDN1	-0.86985	-1.31852	2.188371
ILMN_1736178	AEBP1	1.10683	1.0815	2.18833
ILMN_2124951	RBMX	0.335364	-1.85246	2.187824
ILMN_2088172	POLR2B	1.1159	1.07166	2.18756
ILMN_1765076	APPL2	1.40172	-0.78531	2.187032
ILMN_1780334	KCNJ2	-1.66487	-0.52193	2.186802
ILMN_1673820	HLTF	0.242661	-1.94301	2.185671
ILMN_2219712	HMGB2	-1.91982	0.264853	2.184673
ILMN_1667707	SPCS3	-1.32533	0.859297	2.184627
ILMN_2188722	GLS	0.933913	-1.25071	2.184623
ILMN_2051232	SDHA	0.63806	-1.54619	2.18425
ILMN_1716922	DHX16	1.23884	-0.94538	2.184219
ILMN_3225784	LOC730029	1.25969	-0.92311	2.182796
ILMN_3178792	HNRNPA2B1	1.33385	-0.8486	2.182449
ILMN_2143148	TM2D1	-2.14231	-0.04013	2.1824425
ILMN_2129572	F3	-0.56612	-1.61628	2.1824
ILMN_3246424	LOC100133600	-1.70497	-0.47693	2.181899
ILMN_3294156	LOC388572	0.570264	1.60958	2.179844
ILMN_1723048	GJC2	-1.71237	-0.46721	2.179583
ILMN_2175094	TDRD1	-0.58299	1.59612	2.17911

ILMN_2409062	ISCU	-1.60089	0.577721	2.178611
ILMN_1751615	COQ10B	0.410561	-1.76741	2.177971
ILMN_1766405	GOLM1	1.06855	-1.10925	2.1778
ILMN_2144791	GOLGA6B	0.214015	-1.96378	2.177795
ILMN_1722066	ARMC1	-0.01905	2.15817	2.1772248
ILMN_1720857	GUSBL1	1.32279	-0.85406	2.176851
ILMN_1733851	DACT3	-1.71699	-0.45984	2.17683
ILMN_2229877	PCDH18	-1.84526	0.331164	2.176424
ILMN_2394576	FBXW11	0.879804	-1.29625	2.176054
ILMN_1676955	TYK2	1.19038	-0.98537	2.175749
ILMN_1753719	GTF2A2	1.43077	-0.74485	2.175618
ILMN_1680403	SSR4	-2.10148	-0.07411	2.1755906
ILMN_1771627	ZMIZ1	-0.9624	-1.213	2.175395
ILMN_1681360	LOC652271	-0.69105	1.48424	2.17529
ILMN_3201480	LOC643358	0.605117	1.56919	2.174307
ILMN_1810864	PMP22	-0.4018	-1.77212	2.173922
ILMN_2398432	BRMS1	-0.32119	-1.85208	2.173274
ILMN_1789505	ITPR1	1.60631	-0.56476	2.171066
ILMN_2375599	RNH1	0.704663	-1.46542	2.170083
ILMN_3237400	LOC100133421	-1.3983	0.771719	2.170019
ILMN_1792305	ZNF318	1.00959	-1.16033	2.16992
ILMN_2380839	SNRNP70	-1.54734	-0.62226	2.169602
ILMN_1676737	Sep-02	-0.87319	1.29604	2.169233
ILMN_3178307	KRT18P28	0.901845	-1.26661	2.168455
ILMN_2142185	CLEC14A	-1.32421	-0.84403	2.168241
ILMN_1729161	NOTCH1	-0.65134	-1.51656	2.167903
ILMN_1714383	TPD52L1	-1.20103	0.966675	2.167705
ILMN_3237665	COX7A2L	-0.6784	1.48894	2.167338
ILMN_1732151	COL6A1	1.42942	-0.73771	2.167132
ILMN_1719518	ARF4	0.942587	-1.22448	2.167067
ILMN_1678961	FRMD4A	-0.50326	-1.66336	2.166618
ILMN_3189585	FLJ31306	0.963674	-1.20239	2.166064
ILMN_1774161	ARL15	1.66615	-0.4999	2.166051
ILMN_1664922	FLNB	1.05184	-1.11418	2.16602
ILMN_1756920	ADAM15	1.26446	-0.90145	2.165908
ILMN_3251501	SERF2	1.1299	-1.03563	2.16553
ILMN_1730658	YTHDF2	-1.38453	-0.78095	2.165481
ILMN_1731287	ARFGAP3	-2.06878	-0.09536	2.1641401
ILMN_2325338	APOL2	0.866037	-1.29809	2.164127
ILMN_1811367	MAT2B	-0.9063	1.25781	2.164106
ILMN_1691499	TJP1	1.35414	-0.80989	2.164027
ILMN_1783673	LRRC37A	1.43467	0.72919	2.16386
ILMN_3281039	LOC642909	-1.99143	0.172061	2.163491
ILMN_1729563	UGDH	1.06226	-1.10116	2.16342
ILMN_2339825	YME1L1	-1.01459	1.14881	2.1634
ILMN_1811702	GRN	0.95309	-1.20903	2.16212
ILMN_1732575	SEC14L1	-0.84924	-1.31237	2.161612
ILMN_1661717	TFDP1	1.28602	-0.87498	2.160999
ILMN_1657286	LOC728734	-0.08757	2.07329	2.1608573
ILMN_1804328	WWP1	-1.16451	0.995653	2.160163
ILMN_1655296	UTRN	-1.6094	-0.5504	2.159801
ILMN_3294222	LOC100132673	0.877973	1.28126	2.159233

ILMN_1776077	SF1	-0.99844	-1.16058	2.159021
ILMN_1809478	SSBP1	-0.6593	-1.49965	2.158951
ILMN_1681888	PRKAR2A	1.06362	-1.09526	2.15888
ILMN_2404063	APP	-0.70458	-1.45375	2.158328
ILMN_1725707	ATG16L1	0.835879	-1.32217	2.158049
ILMN_1660547	ERC1	-1.67736	0.480521	2.157881
ILMN_2342695	PDGFA	-1.25705	-0.90081	2.157864
ILMN_3299804	ZNF638	0.856648	-1.3008	2.157448
ILMN_2144426	HIST2H2AA3	1.17908	-0.97836	2.157443
ILMN_1738424	CDC42	0.90853	-1.2488	2.15733
ILMN_1713156	MSL3L1	-2.08119	0.076134	2.1573236
ILMN_1779633	PSMD6	0.381119	-1.77605	2.157169
ILMN_1656868	LOC23117	1.61625	0.540819	2.157069
ILMN_3241729	EMX2OS	-0.70712	1.44974	2.156864
ILMN_1758100	GALR3	0.844553	-1.31201	2.156563
ILMN_3200717	LOC643387	-0.52856	1.62767	2.156232
ILMN_2393046	NHLRC3	-0.7571	-1.39903	2.156126
ILMN_1799069	LOC440280	-0.88354	1.27258	2.156118
ILMN_1695965	LOC653971	0.855534	-1.29948	2.155014
ILMN_1774585	CASP4	1.09696	-1.05804	2.155
ILMN_1774083	TRIAP1	1.14399	-1.01086	2.15485
ILMN_2183409	SCARB1	-0.70211	-1.45218	2.154291
ILMN_2276758	POFUT1	0.036726	2.11738	2.1541059
ILMN_1731714	CREB5	0.422576	-1.72984	2.152416
ILMN_1660568	LOC401622	0.929139	-1.22276	2.151899
ILMN_1662896	BRWD2	-0.16229	1.98936	2.151647
ILMN_1752307	FLT1	0.575038	-1.57658	2.151618
ILMN_1799128	SLC30A9	1.46132	-0.68957	2.150893
ILMN_1697377	LOC649841	0.84694	-1.30394	2.15088
ILMN_1778444	FKBP5	1.2834	-0.86736	2.150763
ILMN_1745669	NY-REN-7	0.684133	1.46644	2.150573
ILMN_3238633	SDHAF2	-1.87414	0.275209	2.149349
ILMN_3242008	DEM1	0.694796	-1.45335	2.148146
ILMN_2408039	EEF1D	0.576471	1.57098	2.147451
ILMN_2279834	ZNF483	0.219585	1.92776	2.147345
ILMN_1775708	SLC2A3	-1.17796	-0.96861	2.146565
ILMN_1656066	TNPO2	1.0516	-1.09492	2.14652
ILMN_3307887	C22orf30	0.921341	-1.22447	2.145811
ILMN_2061950	RABGAP1	0.615382	-1.53038	2.145762
ILMN_1710738	RC3H2	-2.02963	-0.11561	2.14524
ILMN_1724718	NCK2	-0.93327	-1.21187	2.145141
ILMN_1745110	LAPTM4A	-0.47517	1.66957	2.144738
ILMN_3208715	LOC440063	-0.77078	-1.37354	2.144323
ILMN_2182531	C18orf55	1.51711	-0.62697	2.144082
ILMN_1713529	SEMA6A	1.39528	-0.74813	2.143406
ILMN_3255668	LOC100128853	0.305604	1.83681	2.142414
ILMN_1672834	SSH2	0.768322	-1.37406	2.142382
ILMN_2167922	TRMT5	-1.88226	0.25864	2.1409
ILMN_2326282	C19orf6	0.77039	-1.37009	2.14048
ILMN_2059844	ANKRD44	-0.44318	1.6972	2.14038
ILMN_1785175	SWAP70	-0.36249	-1.77758	2.140073
ILMN_3266128	LOC100129553	-0.40101	1.73767	2.138676

ILMN_3279306	LOC100132742	-0.21656	1.92197	2.138525
ILMN_1754211	DCLRE1C	-0.31889	1.81894	2.137827
ILMN_1715273	PICALM	0.88243	-1.25521	2.13764
ILMN_1785158	HERPUD2	-1.6429	0.494717	2.137617
ILMN_3283449	LOC440991	0.609016	1.52693	2.135946
ILMN_2387599	C20orf24	0.202954	-1.93295	2.135904
ILMN_1814305	SAMD9	-0.95054	1.18518	2.135718
ILMN_1660533	RPN1	0.708085	-1.4275	2.135585
ILMN_2334296	IL18BP	-1.30631	-0.82925	2.135559
ILMN_1655913	NUCB2	-0.73832	1.39692	2.135237
ILMN_1676448	WDFY1	0.685804	1.44895	2.134754
ILMN_1815777	NBPF12	1.6706	-0.46344	2.134038
ILMN_1667295	VASN	0.912349	-1.22126	2.133609
ILMN_1688480	CCND1	1.67339	0.460159	2.133549
ILMN_1671565	RNASET2	1.46467	-0.6688	2.133473
ILMN_2186597	RPP21	1.12672	-1.00671	2.13343
ILMN_1773066	CDKN2AIP	1.23311	-0.89996	2.133072
ILMN_3279297	LOC643503	0.921739	-1.21122	2.132959
ILMN_1739854	C15orf29	-1.14947	-0.98348	2.132951
ILMN_1727087	GJA1	1.27759	-0.85523	2.132822
ILMN_2353033	FUBP3	1.0699	-1.06127	2.13117
ILMN_1806502	ZNF165	-1.89444	0.236414	2.130854
ILMN_1722447	LOC642167	1.00816	-1.12241	2.13057
ILMN_1756992	MUC1	1.46085	-0.6687	2.129546
ILMN_1746171	H2AFY	0.24664	-1.88221	2.12885
ILMN_1811117	LOC400986	1.21886	-0.90941	2.128273
ILMN_1664802	WSB1	1.34411	-0.78376	2.127871
ILMN_1676626	ARID4A	-0.38573	-1.74134	2.127068
ILMN_3237941	LOC645159	0.470957	1.6558	2.126757
ILMN_1740429	FTL	-2.03385	0.092803	2.1266531
ILMN_1686626	BAT1	0.262634	-1.86401	2.126644
ILMN_1809957	AP2S1	1.02025	-1.10617	2.12642
ILMN_3246206	SNORA79	0.325577	-1.80072	2.126297
ILMN_2127328	PURA	0.603605	-1.52256	2.126165
ILMN_2285091	ZNF3	-0.14017	1.98576	2.125925
ILMN_2074477	GPR4	0.423292	-1.70147	2.124762
ILMN_1660869	LOC643438	1.06624	-1.05816	2.1244
ILMN_2403823	ZMYM2	-1.20597	-0.9181	2.124069
ILMN_1665095	NELF	0.943701	-1.18002	2.123721
ILMN_2153280	KIAA0090	0.863014	-1.26065	2.123664
ILMN_1697499	HLA-DRB5	-1.74396	-0.37956	2.123518
ILMN_3308961	MIR1974	1.00068	-1.12274	2.12342
ILMN_1784554	LOC647389	-0.16268	1.96068	2.123364
ILMN_3238072	CHADL	0.303057	1.82008	2.123137
ILMN_1654594	SLC34A2	0.656999	-1.46596	2.122959
ILMN_1662353	LOC643547	-0.38923	-1.73362	2.122849
ILMN_2057573	FAM62B	0.532228	-1.59058	2.122808
ILMN_1658883	ARAF	0.565012	-1.55777	2.122782
ILMN_3290298	LOC439953	0.920784	-1.20194	2.122724
ILMN_2103014	CCDC104	1.54488	-0.57767	2.122548
ILMN_2059173	SLC35E1	-0.15799	1.96455	2.12254
ILMN_1737653	KRT78	0.770072	-1.35211	2.122182

ILMN_2415949	MRRF	-1.76783	-0.3543	2.122126
ILMN_2185984	SASH1	-0.31562	-1.80631	2.121934
ILMN_1690064	RUNDC2C	0.586179	-1.53571	2.121889
ILMN_1691843	RNPS1	0.518144	-1.60352	2.121664
ILMN_1667716	TMEM101	1.53859	-0.58282	2.121406
ILMN_2054297	PTGS2	1.0119	-1.10872	2.12062
ILMN_1808707	FSCN1	-0.61052	-1.50997	2.120492
ILMN_1671516	CIR1	1.39703	-0.72221	2.119238
ILMN_1673991	ATIC	1.172	-0.94704	2.119036
ILMN_1703335	LACTB	-0.36337	-1.75377	2.117138
ILMN_2148679	SDHAP3	0.69965	-1.41664	2.11629
ILMN_1667813	LOC648000	-1.12655	0.989718	2.116268
ILMN_1676036	LOC649679	1.62095	0.495291	2.116241
ILMN_1724376	C2orf30	1.15958	-0.95655	2.116125
ILMN_1789999	SLC30A7	1.2986	-0.8175	2.116103
ILMN_1780496	MGC12760	0.822511	-1.29341	2.115921
ILMN_1722985	ZNF658B	0.594773	-1.52053	2.115303
ILMN_1760089	LOC645018	1.43865	-0.67517	2.11382
ILMN_1723480	BST2	1.48567	0.627433	2.113103
ILMN_2323385	TRIM4	0.432125	-1.68069	2.112815
ILMN_1686360	ZNF124	-0.49594	-1.61687	2.112807
ILMN_2100834	ZNF528	-0.31562	1.79707	2.112694
ILMN_1683026	PSMB10	-1.21942	-0.89288	2.112299
ILMN_1667966	C1orf24	0.971551	-1.14067	2.112221
ILMN_1758812	LDHAL6A	-0.297	1.81432	2.111324
ILMN_3268475	LOC100128533	0.637105	-1.47419	2.111295
ILMN_1681760	LOC728518	0.511141	-1.60013	2.111271
ILMN_1670218	EXOSC6	0.12553	-1.98537	2.1109
ILMN_1741406	HOOK1	1.01993	-1.09029	2.11022
ILMN_2414826	VPS13A	0.234227	-1.87594	2.110167
ILMN_2202790	MCFD2	0.11797	1.99208	2.11005
ILMN_3237656	LOC730313	-0.51122	1.59868	2.109895
ILMN_1800461	CSNK2B	-0.15918	1.95052	2.109703
ILMN_2041368	RN7SL1	0.680234	-1.42934	2.109574
ILMN_1715994	HGS	1.07253	-1.03648	2.10901
ILMN_1780058	DEGS1	-1.27949	-0.82946	2.108946
ILMN_1691409	SEC31A	-1.54264	-0.56585	2.108485
ILMN_1733696	IMP3	-0.89317	1.21515	2.108316
ILMN_1693983	MUC5B	0.602332	-1.5059	2.108232
ILMN_1803788	LGALS3	0.292554	-1.81507	2.107624
ILMN_1723874	MRPS6	0.146378	-1.96111	2.107488
ILMN_1807211	NICN1	0.632331	-1.4748	2.107131
ILMN_1702114	NOMO1	0.87304	-1.2327	2.10574
ILMN_1703692	LOC647000	-1.37594	-0.72936	2.105296
ILMN_3242900	HIST2H2AA4	0.84129	-1.2637	2.10499
ILMN_1756676	PHF19	0.01739	2.08756	2.1049497
ILMN_1764090	AK3L1	1.60551	-0.49929	2.104796
ILMN_2405684	BIRC3	-1.70457	0.40012	2.10469
ILMN_1809437	RHBDD2	-0.19284	1.91105	2.103893
ILMN_3203186	LOC100133045	0.896116	-1.2073	2.103416
ILMN_3279414	LOC440595	-0.31507	1.78702	2.102087
ILMN_1694213	PLEKHO1	0.275286	-1.82546	2.100746

ILMN_2410038	FAM111A	0.633127	-1.46678	2.099907
ILMN_1757467	H1F0	1.23733	-0.86139	2.098716
ILMN_2038778	GAPDH	0.825694	-1.27221	2.097904
ILMN_2055930	FARSB	0.590555	-1.50721	2.097765
ILMN_1705570	H2AFY2	1.16157	-0.93504	2.096611
ILMN_1749177	LOC401087	-1.06584	-1.03006	2.0959
ILMN_1770339	RPAIN	-1.16515	-0.93065	2.0958
ILMN_1778202	FLJ40722	-0.13658	1.95913	2.095714
ILMN_2049364	FAM119A	-0.66312	1.43234	2.09546
ILMN_1765547	IRF2	0.019777	-2.075	2.0947769
ILMN_1709026	C6orf145	0.711108	-1.38273	2.093838
ILMN_1686531	GGT1	1.29732	-0.79637	2.093691
ILMN_3297311	LOC729629	1.77277	-0.32086	2.093632
ILMN_2138689	TFAMP1	0.57289	-1.52025	2.09314
ILMN_1705991	GUSBL1	1.23359	-0.85915	2.09274
ILMN_3211079	LOC148430	0.864207	1.22595	2.090157
ILMN_1654313	LOC730995	0.84869	-1.24146	2.09015
ILMN_1696419	STOM	0.750975	1.33898	2.089955
ILMN_1813275	DUSP22	0.768481	-1.32094	2.089421
ILMN_1712432	PSMD2	0.488144	-1.60045	2.088594
ILMN_3214117	LOC100132870	-1.25435	-0.83421	2.088564
ILMN_1664921	PPP6C	-1.16379	-0.92462	2.08841
ILMN_1743677	HNRNPU	1.4614	-0.62681	2.088207
ILMN_1767393	GRIA3	-0.22682	1.86093	2.08775
ILMN_1791388	ZNF787	1.34698	-0.74029	2.087266
ILMN_1792518	STX7	1.21552	-0.87128	2.086795
ILMN_3248263	CCDC93	-0.20971	-1.87672	2.086432
ILMN_1787514	CAPN12	1.08224	-1.0035	2.08574
ILMN_1679268	PELI1	0.260884	-1.82458	2.085464
ILMN_1732074	LOC648210	0.990251	-1.09507	2.085321
ILMN_3208233	LOC100131735	1.19953	-0.88574	2.085267
ILMN_1714622	TNRC6A	-1.66121	0.424015	2.085225
ILMN_1804327	NAP1L4	1.13905	-0.94617	2.085222
ILMN_1671589	LOC641995	0.96996	-1.11504	2.085
ILMN_3226663	MGC26356	-0.45575	1.62922	2.084972
ILMN_2390974	DNAJB2	-0.27759	-1.80646	2.084048
ILMN_1670272	LRP10	-1.69805	-0.38555	2.083596
ILMN_1694100	PRIM2	-0.21727	1.86629	2.083562
ILMN_1784300	TUBA4A	1.40634	-0.67712	2.083457
ILMN_1727194	CALU	1.18345	-0.89997	2.083418
ILMN_1664560	DYRK1A	-1.28514	0.798235	2.083375
ILMN_1665540	LOC728417	0.828638	1.2545	2.083138
ILMN_2412549	GAR1	1.1572	-0.92537	2.082566
ILMN_2090949	NAG18	1.20645	-0.87574	2.082186
ILMN_1660923	HCG4	-0.52999	-1.55092	2.080914
ILMN_1674250	NCKAP1L	-2.01547	-0.06535	2.0808168
ILMN_2092536	HSPE1	-1.88242	0.197938	2.080358
ILMN_3244395	LOC728877	-1.18019	0.899879	2.080069
ILMN_1811909	LOC402221	-1.76783	0.311713	2.079543
ILMN_2052208	GADD45A	0.525066	-1.55447	2.079536
ILMN_1718334	ITPA	0.186721	-1.89234	2.079061
ILMN_2128741	RDH11	1.55427	-0.52435	2.078622

ILMN_1710362	LOC644591	-0.41764	1.66051	2.078147
ILMN_1800575	LOC649260	0.307434	-1.77038	2.077814
ILMN_1658647	LOC642778	0.389474	-1.68797	2.077444
ILMN_3178050	CYTSB	-1.72972	-0.34759	2.077306
ILMN_3246604	IPW	1.01301	1.06382	2.07683
ILMN_3236156	OSTC	0.681348	-1.39528	2.076628
ILMN_1721102	LOC255326	-1.6437	0.43269	2.07639
ILMN_1745887	FBXO21	0.721055	-1.35527	2.076325
ILMN_1657884	NME2	-0.29231	-1.78282	2.075129
ILMN_2129715	COG3	-0.39687	-1.67781	2.074678
ILMN_1804248	FDPS	1.10189	-0.97277	2.074661
ILMN_3233132	LOC100130224	-0.10412	1.97047	2.074589
ILMN_1774547	MPRIP	-0.80062	-1.27369	2.074312
ILMN_2387090	CGGBP1	-1.20469	-0.86953	2.074219
ILMN_3226045	LOC728533	0.769515	-1.30458	2.074095
ILMN_3283155	LOC642357	0.515279	1.55828	2.073559
ILMN_2383754	GTPBP10	-0.23653	1.83658	2.073108
ILMN_3226495	LOC730173	-0.33353	1.73938	2.072908
ILMN_1802252	GAPDH	1.30457	-0.768	2.072573
ILMN_2330341	TCEAL4	1.18258	0.888718	2.071298
ILMN_1800220	KCTD3	1.4949	-0.57579	2.070692
ILMN_2218208	SPARCL1	1.71214	-0.35845	2.07059
ILMN_1784706	GABRE	1.28873	-0.78144	2.070166
ILMN_2281225	PALM2-AKAP2	1.12672	-0.9431	2.069815
ILMN_1758852	ENTPD7	0.612995	-1.45658	2.069575
ILMN_1752631	CGGBP1	1.39528	-0.67429	2.069574
ILMN_1808238	RBPMS2	1.52331	-0.54597	2.069278
ILMN_1756998	LOC647691	1.49976	-0.56877	2.068534
ILMN_3248707	KCNQ1OT1	-1.25817	0.809649	2.067819
ILMN_1730824	ZNF512	-1.0395	-1.02821	2.06771
ILMN_1692733	NRARP	-0.6749	-1.39276	2.067657
ILMN_1697024	LOC730432	1.3247	0.742605	2.067305
ILMN_1703617	AHSA1	0.931287	1.13578	2.067067
ILMN_3284768	LOC400013	0.736015	-1.32995	2.065965
ILMN_3221032	LOC728611	-1.25538	0.81046	2.06584
ILMN_2333670	RNASE1	-1.52195	-0.54368	2.065628
ILMN_1746941	HIPK3	0.864526	-1.20091	2.065436
ILMN_2125590	LOC401252	0.202556	-1.8627	2.065256
ILMN_1779014	TSPYL1	1.36027	-0.7042	2.064471
ILMN_1713163	SMARCA5	0.457191	-1.60703	2.064221
ILMN_1776052	LOC148915	-1.69574	-0.36788	2.063623
ILMN_3248781	SDHAP2	0.959854	-1.10372	2.063574
ILMN_2355042	CLUAP1	1.57392	-0.4893	2.063217
ILMN_1785988	CSNK1A1	1.35239	0.710721	2.063111
ILMN_1759396	NNT	1.54193	-0.52067	2.062601
ILMN_1748968	ATG10	1.30218	-0.76017	2.062354
ILMN_3298334	LOC729313	-0.52896	-1.53313	2.06209
ILMN_3259146	LOC100129681	1.69097	-0.37072	2.061687
ILMN_1738346	EFHA1	0.445971	-1.61571	2.061681
ILMN_3238297	LOC641787	-0.62564	1.43505	2.060691
ILMN_1685445	NOTCH2NL	-0.14987	-1.91055	2.060423
ILMN_2379393	ASL	-0.48695	1.57333	2.060275

ILMN_1704253	C6orf106	0.240433	1.81972	2.060153
ILMN_3251616	C2orf67	0.823386	-1.23591	2.059296
ILMN_1770085	BTG2	1.16038	-0.89871	2.059094
ILMN_1662719	GPBP1L1	0.055426	2.00356	2.0589856
ILMN_2338323	CDC25B	1.36202	-0.69688	2.058895
ILMN_2412101	PRKAG1	-0.00417	2.05386	2.05803467
ILMN_3251361	NBPF11	1.48631	-0.57141	2.057718
ILMN_3304435	LOC728640	-1.37888	-0.67875	2.05763
ILMN_1782488	RNASEH2B	0.403319	-1.65421	2.057529
ILMN_2151579	HMGN1	0.430136	1.62731	2.057446
ILMN_2043265	ZFP106	0.850123	-1.20723	2.057353
ILMN_1734276	PMEPA1	-1.73951	-0.31708	2.056591
ILMN_1814789	UBAP2L	-1.98889	-0.06681	2.0556984
ILMN_1805344	DDX5	1.34109	-0.71433	2.055422
ILMN_2056074	RGPD2	0.899538	-1.15583	2.055368
ILMN_1692511	TMEM106C	0.95134	-1.10371	2.05505
ILMN_1792497	HRB	-0.18115	1.87363	2.054775
ILMN_2057836	RNU2-1	0.908609	-1.14609	2.054699
ILMN_3232894	CNRIP1	0.408571	-1.64592	2.054491
ILMN_2233878	SERF1B	-1.40228	0.650251	2.052531
ILMN_3297410	RPS6P1	0.722328	-1.33019	2.052518
ILMN_1718833	UGT2B7	1.18632	-0.86619	2.052511
ILMN_1652128	LMBRD1	0.763149	-1.28866	2.051809
ILMN_1655316	PSMC6	-2.02231	-0.02929	2.0515958
ILMN_1797793	BLVRB	1.6663	-0.38476	2.051059
ILMN_1755405	FRAG1	-1.02979	1.0212	2.05099
ILMN_1684346	TNFAIP8L1	-0.54559	1.50524	2.05083
ILMN_1691104	PGAM4	1.08582	-0.96461	2.050431
ILMN_1729058	SCAMP1	0.684531	-1.36564	2.050171
ILMN_2213136	LEF1	1.57233	-0.4772	2.049526
ILMN_1752968	LAMB2	0.817498	-1.23158	2.049078
ILMN_1670532	GMCL1	1.27624	-0.77256	2.0488
ILMN_3284404	LOC285741	0.07317	-1.97533	2.0485004
ILMN_3305339	UBA5	1.46681	-0.58134	2.048153
ILMN_1673933	LOC440341	-1.61553	0.4324	2.04793
ILMN_1757387	UCHL1	0.708085	1.33978	2.047865
ILMN_2358277	RSRC2	-1.97146	0.076376	2.0478362
ILMN_1687510	HRNR	0.686043	-1.36157	2.047613
ILMN_3213185	LOC645452	0.254836	1.79266	2.047496
ILMN_1788778	Sep-11	-0.7481	-1.29911	2.047214
ILMN_2402499	SC4MOL	0.22046	1.82655	2.04701
ILMN_1657949	RHEB	-0.90542	-1.14129	2.04671
ILMN_1697670	SRRM1	1.38024	-0.66643	2.04667
ILMN_1672287	MYNN	0.861502	-1.18462	2.046122
ILMN_3243909	SNORD102	0.659306	-1.38672	2.046026
ILMN_3231638	FAM160B1	-1.56922	-0.47672	2.045942
ILMN_2257607	STARD13	-0.58633	-1.45942	2.045752
ILMN_1670420	METAP2	0.568354	-1.47726	2.045614
ILMN_1677092	GEM	0.324542	-1.7207	2.045242
ILMN_1671356	COQ6	0.576232	-1.46858	2.044812
ILMN_1761941	C4orf18	0.702276	-1.34226	2.044536
ILMN_1679929	KLF13	1.04189	-1.00228	2.04417

ILMN_1726222	FLOT2	0.842165	-1.20198	2.044145
ILMN_2342033	F11R	-1.18361	-0.86045	2.044062
ILMN_2105254	PTGR2	-1.43816	-0.60585	2.044007
ILMN_2097410	DAPP1	-0.16101	1.88238	2.043393
ILMN_1762808	LOC651919	0.969164	-1.07404	2.043204
ILMN_2260756	GSDMB	-0.1381	1.90486	2.042956
ILMN_1652533	DKFZp434K191	-0.66941	1.37342	2.042826
ILMN_1774735	DDB1	-1.93287	0.109845	2.042715
ILMN_1676600	SEC24C	-0.29008	-1.75239	2.042471
ILMN_2311798	TES	-0.7158	1.32589	2.041687
ILMN_1704961	ACTG1	0.517109	-1.52449	2.041599
ILMN_1808757	RPL37A	-1.35692	0.684312	2.041232
ILMN_1750278	FTHL12	-1.61243	0.428648	2.041078
ILMN_1670817	GRPEL1	0.335682	1.70387	2.039552
ILMN_1805827	PPA1	-0.61665	-1.42258	2.039229
ILMN_2073307	IL10	-0.98579	1.05236	2.038149
ILMN_1782419	GNG11	0.988262	-1.04938	2.037642
ILMN_2174127	DCBLD2	0.950305	-1.08732	2.037625
ILMN_1692956	LOC643949	-1.4836	0.553418	2.037018
ILMN_3267275	LOC100128979	0.31189	-1.72338	2.03527
ILMN_2243516	C11orf63	0.420746	1.61452	2.035266
ILMN_1689012	NBPF10	0.695353	-1.33981	2.035163
ILMN_2330552	CDC2L2	1.52403	-0.5109	2.034926
ILMN_1754235	SLC35C2	0.353905	-1.68082	2.034725
ILMN_1779735	C7orf59	-1.63558	-0.39865	2.034233
ILMN_2204876	FLVCR2	0.858001	1.17592	2.033921
ILMN_2222101	N4BP2	0.331226	1.70261	2.033836
ILMN_1756631	ZNF526	0.606549	-1.4265	2.033049
ILMN_1810785	RNF11	1.12346	-0.90953	2.032991
ILMN_1794782	ABCG1	-0.79641	1.23611	2.032515
ILMN_1777318	C9orf64	0.541936	-1.49055	2.032486
ILMN_2174574	HNRNPA3P1	0.165077	-1.86698	2.032057
ILMN_3204898	LOC644101	0.661853	-1.36991	2.031763
ILMN_1711156	LOC645895	-0.35215	1.67927	2.031418
ILMN_1683120	UNG	-1.96048	0.070595	2.0310749
ILMN_3243296	LOC730378	0.528647	1.50213	2.030777
ILMN_3274677	LOC644384	0.684929	-1.3454	2.030329
ILMN_1725247	GTF2A1	0.36218	-1.66795	2.03013
ILMN_3300663	LOC728661	-0.20367	-1.82629	2.029955
ILMN_3304872	LOC728693	-0.68763	1.34171	2.029339
ILMN_1676088	MSRB3	-1.27305	-0.75584	2.028887
ILMN_1697952	OSBPL1A	-0.7754	1.25276	2.028158
ILMN_1694398	EIF3F	1.07874	-0.94923	2.027971
ILMN_2117330	NDUFB2	-0.97194	-1.05567	2.027613
ILMN_3245983	NEURL1B	1.47079	-0.55629	2.027078
ILMN_1810678	LOC642819	-1.61824	0.408162	2.026402
ILMN_3248970	LOC100134266	-1.64473	-0.38143	2.026158
ILMN_3193186	LOC100128260	-0.44358	-1.5819	2.025478
ILMN_2221006	RAD21	1.27465	-0.75072	2.025374
ILMN_1762899	EGR1	-1.86261	0.162676	2.025286
ILMN_1761281	LOC441019	-2.00194	-0.02332	2.0252551
ILMN_1655635	METTL3	1.59914	0.42608	2.02522

ILMN_1755737	TRABD	1.30289	-0.72233	2.025216
ILMN_1760778	ENG	-0.19181	-1.83333	2.025138
ILMN_1719579	LOC441081	0.730445	-1.29295	2.023395
ILMN_1685289	C16orf58	-1.39678	-0.62627	2.023054
ILMN_2381037	LIMS1	-0.83325	-1.18963	2.022877
ILMN_2075440	PDIA3P	0.632808	-1.38992	2.022728
ILMN_1781045	FXYD2	0.741267	-1.28099	2.022257
ILMN_1812250	LOC644642	-0.87208	1.14916	2.021239
ILMN_2380566	SIAH1	1.2156	-0.80538	2.020978
ILMN_1737074	LOC440589	0.972347	1.04798	2.020327
ILMN_1790549	TSPAN3	0.870573	-1.14966	2.020233
ILMN_2180682	MTPN	0.517666	-1.50189	2.019556
ILMN_1719828	LOC653052	1.56206	-0.45709	2.019154
ILMN_1681300	LOC641806	-0.69535	-1.32304	2.018387
ILMN_2271014	ALG8	-0.17231	-1.84561	2.017923
ILMN_1809582	VPS13C	1.11041	-0.90746	2.01787
ILMN_3200018	LOC442609	-0.91012	-1.10767	2.017785
ILMN_3283563	LOC344423	-1.87208	0.145516	2.017596
ILMN_1683781	ADAMTS15	0.747155	-1.26976	2.016915
ILMN_1678457	ZNF600	0.558089	-1.45839	2.016479
ILMN_1770800	PODN	0.464591	-1.55174	2.016331
ILMN_2070300	LSM2	1.1116	0.9045	2.0161
ILMN_1719203	DISC1	-0.86277	1.15254	2.015309
ILMN_1774207	ANGPT2	-0.50708	-1.50717	2.014247
ILMN_1805216	GPC6	0.976724	-1.03744	2.014164
ILMN_1670895	ZNF207	-0.70386	-1.3102	2.014061
ILMN_1775036	SEC22A	-0.80174	1.21214	2.013876
ILMN_1782057	ATP8B2	-0.73736	-1.27633	2.013692
ILMN_2365196	PDCD10	1.30464	-0.70878	2.013421
ILMN_3274344	LOC100131076	1.0368	0.976575	2.013375
ILMN_3188449	LOC100129751	0.198657	-1.81418	2.012837
ILMN_3242405	RMRP	-0.71898	-1.29316	2.01214
ILMN_1744725	BTBD6	-1.14008	-0.87189	2.01197
ILMN_1658494	C13orf15	0.491964	-1.51919	2.011154
ILMN_1653667	LOC643423	-1.03425	0.976865	2.011115
ILMN_2081988	LANCL1	1.30751	-0.70341	2.010924
ILMN_1659006	CBWD6	0.917601	1.09321	2.010811
ILMN_1809550	LOC401620	0.743654	-1.26698	2.010634
ILMN_2173740	ASB8	-0.97465	1.03584	2.010489
ILMN_2052891	PKD2	0.233749	-1.77673	2.010479
ILMN_3308038	MIR1322	1.34992	-0.66047	2.010391
ILMN_1651966	FGFR3	1.20526	-0.80511	2.010371
ILMN_1657746	BPHL	0.688112	-1.3214	2.009512
ILMN_1736700	ALDOA	0.875029	-1.13386	2.008889
ILMN_1747924	LOC644254	1.03426	-0.97455	2.008812
ILMN_1810852	LAMC1	-0.83699	-1.17079	2.007777
ILMN_3245274	SNORD94	-0.64808	-1.35961	2.007691
ILMN_1665945	ACBD3	1.47223	-0.53532	2.007547
ILMN_1795826	ATP6V0D1	0.579256	-1.42825	2.007506
ILMN_1660063	POLE4	-1.46371	-0.54274	2.006447
ILMN_1770281	ERC1	0.557453	-1.44891	2.006363
ILMN_2186216	GOLPH4	1.01587	-0.98979	2.005659

ILMN_1779404	PUF60	1.3804	-0.62517	2.005574
ILMN_2259119	PRMT2	0.821476	-1.18377	2.005246
ILMN_1780842	RANBP6	1.04786	-0.95739	2.005245
ILMN_1705390	KLHL22	-0.45766	-1.54735	2.005012
ILMN_3242362	LOC92973	0.355178	-1.64973	2.004908
ILMN_1788283	COTL1	0.34921	-1.65555	2.00476
ILMN_1665212	EDC4	0.055664	1.94893	2.0045944
ILMN_2340439	ZFP64	-0.38732	1.61699	2.004309
ILMN_1712386	C21orf45	1.12242	-0.88186	2.004275
ILMN_3211187	LOC100133479	0.238444	-1.76548	2.003924
ILMN_2162465	RKHD1	-1.66558	0.338295	2.003875
ILMN_1807492	LOC643668	0.153141	-1.85059	2.003731
ILMN_3251699	APBB2	-0.41573	1.58788	2.003607
ILMN_2409395	CCNC	1.07189	-0.93134	2.003227
ILMN_1788160	KIF5B	-0.94807	-1.05477	2.002842
ILMN_1710264	LOC728226	1.37236	-0.63045	2.002806
ILMN_1666471	UQCRQ	-1.54479	0.457892	2.002682
ILMN_2096012	UHMK1	1.21298	-0.78951	2.002487
ILMN_3193231	LOC100128693	-0.51002	1.49159	2.001611
ILMN_1707464	MST1	1.33528	-0.66625	2.001527
ILMN_3243890	NDUFA2	-0.5288	1.47254	2.001341
ILMN_1656998	LOC653555	0.131259	-1.87006	2.001319
ILMN_1700549	ERLIN2	0.39568	-1.60487	2.00055
ILMN_1783448	DYNC1LI2	1.05749	-0.94286	2.000348
ILMN_1769191	GNAS	-1.61028	0.389143	1.999423
ILMN_3256693	LOC100128476	0.916487	1.08287	1.999357
ILMN_3221499	LOC730291	-0.36822	-1.63104	1.999262
ILMN_2194448	STT3B	0.504537	-1.49466	1.999197
ILMN_1792344	LOC728498	1.299	-0.69998	1.998976
ILMN_1733176	LIMS1	-1.48622	-0.51254	1.998755
ILMN_1666670	RBX1	0.33982	-1.6588	1.99862
ILMN_2070477	TAF8	-0.60495	1.39284	1.997792
ILMN_3304111	LOC729978	1.75463	-0.24276	1.997388
ILMN_2364768	MYLK	-0.80094	-1.19625	1.997191
ILMN_1674719	NID1	-0.45321	-1.54394	1.997146
ILMN_1674620	SGCE	0.473344	-1.52296	1.996304
ILMN_1672006	SSB	-1.0138	-0.98249	1.996293
ILMN_1724628	LOC144383	0.716122	-1.27899	1.995112
ILMN_1887174	KIAA0146	1.09998	-0.89501	1.994989
ILMN_2065299	EDEM3	-1.69837	-0.29596	1.994326
ILMN_1682206	GCC1	-0.19682	-1.7975	1.994321
ILMN_1731395	LOC644380	-1.87176	0.122059	1.993819
ILMN_3243744	EAPP	1.33624	-0.65728	1.993516
ILMN_1695311	HLA-DMA	1.20271	0.790483	1.993193
ILMN_2396956	AKAP13	1.09393	-0.89926	1.993188
ILMN_2334760	ARMCX3	0.536923	1.45618	1.993103
ILMN_1756849	HIST1H2AE	0.404115	-1.58889	1.993005
ILMN_2352159	BBS9	-0.41294	1.57999	1.992932
ILMN_1709032	FYCO1	0.448597	1.54391	1.992507
ILMN_1704537	PHGDH	0.484405	1.5079	1.992305
ILMN_3198090	LOC100132947	1.41215	-0.58	1.99215
ILMN_3288706	LOC440311	0.742858	-1.2491	1.991958

ILMN_2318568	HCFC1R1	0.70331	-1.28852	1.99183
ILMN_3246097	LOC649839	0.782088	-1.20939	1.991478
ILMN_1771728	PXMP4	1.37165	-0.61923	1.990882
ILMN_1668092	ESAM	0.275684	-1.71478	1.990464
ILMN_1655163	STK24	0.245128	-1.74471	1.989838
ILMN_2340935	WBP5	1.34499	0.644795	1.989785
ILMN_1676842	BTAF1	-0.558	-1.43168	1.989684
ILMN_1659550	LOC388654	1.0002	-0.98857	1.98877
ILMN_3245517	LOC100134273	0.930412	1.05805	1.988462
ILMN_1698732	PALLD	-0.55411	-1.43402	1.988125
ILMN_1786105	PCBD1	1.45273	-0.53405	1.986781
ILMN_2298818	RPS29	0.44231	-1.5444	1.98671
ILMN_1660000	SNURF	0.745325	-1.24044	1.985765
ILMN_1666420	ZNF679	-0.41398	-1.57137	1.985346
ILMN_3202432	LOC644464	1.13523	-0.84981	1.985042
ILMN_1675488	SLC13A3	0.623021	1.36196	1.984981
ILMN_3231554	LOC728093	1.2803	-0.70452	1.984815
ILMN_1685275	MCAM	-1.14907	-0.83554	1.984609
ILMN_1726981	VEGFB	1.67243	-0.31136	1.983789
ILMN_3286067	LOC391578	0.649917	-1.33363	1.983547
ILMN_1749821	MED28	0.42377	-1.55966	1.98343
ILMN_2089329	SPRY2	0.81145	-1.17172	1.98317
ILMN_1689445	POLR2H	1.57877	-0.40399	1.982755
ILMN_1666399	RING1	0.188392	-1.79416	1.982552
ILMN_2414165	PRDM1	1.72622	0.25632	1.98254
ILMN_2408450	UBE1DC1	0.983647	-0.99865	1.982301
ILMN_1686516	CUGBP1	-1.5498	-0.43228	1.982082
ILMN_1813117	ITGA9	-1.84788	-0.1339	1.981781
ILMN_1666485	HERC2P2	0.728217	1.25315	1.981367
ILMN_1797828	DDRGK1	-1.31077	-0.67047	1.981236
ILMN_1680867	C6orf61	-0.55442	1.4258	1.980223
ILMN_1699015	H3F3A	0.805244	1.17495	1.980194
ILMN_2070210	RNF219	0.662171	-1.31794	1.980111
ILMN_1744912	CTTN	0.819805	-1.16024	1.980045
ILMN_1797861	IL6ST	1.21592	0.763718	1.979638
ILMN_1762769	POLR2J4	-0.48726	-1.49173	1.978993
ILMN_1720088	SFRS12	-1.22857	0.750167	1.978737
ILMN_1692191	GNA12	0.219426	-1.75926	1.978686
ILMN_1691648	TRO	-0.10889	-1.86978	1.978673
ILMN_2272967	TRAPPC6B	1.50111	-0.47689	1.978004
ILMN_3305475	LOC729708	1.37411	-0.60389	1.977998
ILMN_1768558	LOC727848	-0.31029	-1.66759	1.977883
ILMN_1675130	NFIC	1.19603	0.781234	1.977264
ILMN_1771593	RRM1	-1.50779	-0.46914	1.976932
ILMN_1759670	AMACR	0.975371	-1.0002	1.975571
ILMN_3245151	URB1	-0.18775	1.78749	1.97524
ILMN_3278137	LOC642585	1.59334	-0.38129	1.974626
ILMN_1755926	DBI	0.156722	-1.81768	1.974402
ILMN_3282517	LOC100131871	0.799912	1.17418	1.974092
ILMN_3214332	LOC727963	0.105318	1.86855	1.973868
ILMN_1806320	PPFIBP1	-0.80739	-1.16647	1.973856
ILMN_1798533	ZNF22	0.48719	-1.48615	1.97334

ILMN_2126239	SMG5	-0.9398	-1.03348	1.973276
ILMN_1695341	KCNK10	1.06083	-0.91216	1.972988
ILMN_1730957	NBPF10	0.538037	-1.43475	1.972787
ILMN_1802753	TSSC4	0.708642	-1.26376	1.972402
ILMN_2107655	SNORD116-2	-0.67386	-1.29854	1.972402
ILMN_1737497	LOC649009	0.01914	1.95311	1.9722503
ILMN_3226955	RELL1	1.19364	-0.7786	1.972241
ILMN_1757877	HCFC1R1	1.02351	-0.94844	1.971948
ILMN_1715496	HTR2A	1.20287	-0.76872	1.971589
ILMN_1668629	LOC401115	-0.98714	0.984179	1.971321
ILMN_1675844	WDR1	1.09521	-0.87609	1.971295
ILMN_1756573	NDUFA4L2	0.856966	-1.114	1.970966
ILMN_1735467	LOC643943	-1.08677	-0.88372	1.970489
ILMN_3268414	LOC100128083	1.01046	0.959669	1.970129
ILMN_3208216	LOC100131940	-0.22053	1.74933	1.969864
ILMN_3247400	LOC572558	0.555304	-1.41449	1.969794
ILMN_1661799	HIGD1A	1.43117	-0.53801	1.969179
ILMN_2243687	LAMP2	0.655407	-1.31365	1.969057
ILMN_1657790	SFRS11	1.53994	-0.42864	1.968583
ILMN_3239785	LOC100134304	-1.715	0.252752	1.967752
ILMN_1692517	LOC653381	-0.95579	1.01195	1.96774
ILMN_3187612	LOC100128084	0.143991	1.82316	1.967151
ILMN_1722502	CCT6A	0.669412	-1.29768	1.967092
ILMN_2398159	DKK3	0.526976	-1.43988	1.966856
ILMN_2092664	ADSS	0.642596	-1.32383	1.966426
ILMN_1809086	XRN1	-0.43618	-1.52957	1.965747
ILMN_3259283	LOC100128747	0.468888	-1.4968	1.965688
ILMN_2134538	FTHL11	-0.92181	1.04299	1.964802
ILMN_1721391	ATP6V0B	0.72567	-1.23851	1.96418
ILMN_1748437	LOC646568	0.810734	-1.15337	1.964104
ILMN_2380698	DSTN	0.741346	-1.22259	1.963936
ILMN_1778709	PICALM	-0.65445	-1.30915	1.963597
ILMN_1767556	C10orf10	-0.66121	1.30231	1.96352
ILMN_1739573	TNRC6A	0.457668	1.50523	1.962898
ILMN_1729408	LOC647357	0.090915	1.87174	1.9626553
ILMN_1803423	ARHGEF6	-0.33257	1.63006	1.962633
ILMN_1698706	NID2	0.560715	-1.40188	1.962595
ILMN_1785424	ABLIM1	0.490134	-1.47183	1.961964
ILMN_2124187	TSC22D2	1.01747	-0.94443	1.961902
ILMN_2375879	VEGFA	0.425282	-1.53545	1.960732
ILMN_2307883	ATP5J2	0.388837	-1.5714	1.960237
ILMN_1786718	NDUFV1	0.339104	-1.62074	1.959844
ILMN_1688818	LOC652864	0.561034	-1.39812	1.959154
ILMN_1673966	POLR3F	1.06577	-0.89338	1.959152
ILMN_1656399	TCEAL8	-0.72487	-1.23372	1.958589
ILMN_1652123	HMGN1	1.32462	-0.63395	1.958569
ILMN_3265237	FLJ44342	-0.3208	1.63766	1.958456
ILMN_1726245	TGFBR2	1.10134	-0.85662	1.957963
ILMN_2368773	FAM3C	1.13277	-0.82519	1.957959
ILMN_3251572	FAM134A	0.665831	-1.29207	1.957901
ILMN_1796268	LOC441114	1.163	-0.79487	1.957874
ILMN_1705151	SF3A3	1.61005	-0.3477	1.957754

ILMN_1713086	RPL27A	-0.85545	1.10173	1.957178
ILMN_2161286	FAM40B	0.469922	1.48723	1.957152
ILMN_3241965	LOC100134774	0.685566	-1.27116	1.956726
ILMN_1721128	TOMM34	-0.95953	0.996895	1.956425
ILMN_1758412	COPS7A	0.498807	-1.4576	1.956407
ILMN_1663685	DGCR6	-0.83866	1.11731	1.955968
ILMN_2347349	CCNB1IP1	0.896196	-1.0594	1.955596
ILMN_2410909	AKT1	0.671083	-1.28451	1.955593
ILMN_3278004	LOC100132652	-0.11844	1.83695	1.955392
ILMN_1733937	MMD	0.514483	-1.44084	1.955323
ILMN_2208903	CD52	0.755669	1.19921	1.954879
ILMN_1740587	RPS11	-0.40825	1.54643	1.954677
ILMN_3263375	LOC100130604	0.115742	1.8389	1.954642
ILMN_1653910	LOC650496	1.10141	-0.85282	1.954234
ILMN_1781691	TRAK2	1.02502	-0.9292	1.95422
ILMN_1658697	LOC730953	1.03298	0.920341	1.953321
ILMN_1812105	PIP5K2B	0.36974	1.58306	1.9528
ILMN_2326071	MYL6	1.4918	0.459768	1.951568
ILMN_2414436	RBM3	-0.43538	-1.51605	1.951432
ILMN_1740308	C2orf21	-0.32486	-1.62616	1.951015
ILMN_3297945	UBA1	0.524589	-1.42622	1.950809
ILMN_3269402	LOC100130420	1.38684	-0.56348	1.950324
ILMN_3248635	LOC100133744	-1.45551	0.494705	1.950215
ILMN_1770515	UBE2V2	0.945611	1.00444	1.950051
ILMN_2308950	AKAP12	-0.42042	-1.52931	1.949732
ILMN_2150465	C5orf28	1.06394	0.884612	1.948552
ILMN_1723978	LGALS1	-0.23056	-1.71794	1.9485
ILMN_1694671	ZFAND2A	1.04794	-0.90041	1.948346
ILMN_3187571	LOC100130844	0.436581	-1.5114	1.947981
ILMN_1708064	MAP4	-0.05176	-1.89617	1.9479294
ILMN_1667460	SULF2	1.71286	0.235023	1.947883
ILMN_1653738	INTS4	0.503661	-1.44412	1.947781
ILMN_1671703	ACTA2	-0.64697	-1.30058	1.947547
ILMN_1809099	IL33	0.395362	-1.55134	1.946702
ILMN_3302896	LOC728820	-0.36958	1.57673	1.946305
ILMN_1772036	STEAP4	0.32112	-1.62505	1.94617
ILMN_1732799	CD34	0.461249	-1.4845	1.945749
ILMN_1732705	HCFC1	0.573686	-1.37187	1.945556
ILMN_1667893	TNS3	0.397192	-1.54785	1.945042
ILMN_1703228	AGFG2	1.216	-0.72898	1.944977
ILMN_1720048	CCL2	-1.76704	0.177482	1.944522
ILMN_3235800	LOC100134528	0.935823	1.00862	1.944443
ILMN_1740724	LOC392221	1.00402	0.940082	1.944102
ILMN_2181432	SPC24	-0.73657	1.20637	1.942936
ILMN_1812557	CDK5RAP3	-0.34952	-1.59338	1.942902
ILMN_1689774	MRFAP1L1	1.20223	-0.73998	1.942208
ILMN_2395932	UNC45A	-1.52299	0.41921	1.9422
ILMN_2058841	LILRA6	-0.33162	1.61025	1.941868
ILMN_2338997	PTP4A2	-0.33743	-1.60373	1.941157
ILMN_3234922	LOC728790	0.921977	1.01851	1.940487
ILMN_1692177	TSC22D1	0.486553	-1.45313	1.939683
ILMN_1721729	PPARBP	0.985158	-0.95427	1.939425

ILMN_1659975	C1orf216	-0.17152	1.76789	1.939407
ILMN_1798181	IRF7	0.715644	-1.22359	1.939234
ILMN_1669502	E2F3	0.768162	-1.17073	1.938892
ILMN_1717639	SIK1	1.37507	-0.56357	1.938637
ILMN_1729650	PEX7	0.979031	0.959006	1.938037
ILMN_1652207	COX4I1	-1.63781	0.299807	1.937617
ILMN_3274351	LOC644037	0.530239	-1.40734	1.937579
ILMN_3273065	LOC100130367	0.665195	-1.27221	1.937405
ILMN_1706553	SMG7	0.629069	-1.30721	1.936279
ILMN_1699058	LOC644979	-0.73147	1.20463	1.936103
ILMN_1704424	PLA2R1	0.323189	-1.61291	1.936099
ILMN_1815666	ATP2A2	0.962401	-0.97369	1.936089
ILMN_1796094	CD36	0.558805	-1.37681	1.935615
ILMN_3219197	LOC100132528	1.00473	-0.93088	1.935605
ILMN_3243813	LOC727914	-1.63678	0.298707	1.935487
ILMN_2278152	TPM1	0.861343	-1.07386	1.935203
ILMN_2105253	PTGR2	0.251096	1.68363	1.934726
ILMN_1786720	PROM1	-1.46227	0.472337	1.934607
ILMN_3235027	TAF1D	1.18855	-0.74583	1.93438
ILMN_1662130	LOC730316	-1.29175	-0.64243	1.934179
ILMN_1672503	DPYSL2	-1.54057	0.392741	1.933311
ILMN_3300226	LOC731275	1.04858	0.884718	1.933298
ILMN_1673880	EFEMP1	0.730126	-1.20263	1.932756
ILMN_1734190	TCEAL3	1.46069	-0.47204	1.932732
ILMN_1722872	MYH9	-1.42312	-0.50961	1.932732
ILMN_1697665	LOC653972	0.551564	-1.38089	1.932454
ILMN_1807397	NDUFB5	-1.89284	-0.03878	1.9316234
ILMN_3237617	RNU5A	0.748667	1.18285	1.931517
ILMN_2141650	MALAT1	-0.16133	1.77015	1.931482
ILMN_3203625	LOC100131323	0.16269	-1.76845	1.93114
ILMN_1814039	LOC653086	0.382949	1.5478	1.930749
ILMN_1815447	LOC401623	1.00362	-0.92706	1.930678
ILMN_1729497	LOC648145	0.82633	-1.10426	1.93059
ILMN_1745954	CORO1C	1.06974	0.860806	1.930546
ILMN_1801040	SPN	0.340218	1.58986	1.930078
ILMN_1697448	TXNIP	0.48337	1.44663	1.93
ILMN_2363392	TNFSF14	-0.52267	1.40685	1.929523
ILMN_1798957	C12orf47	1.78511	-0.14439	1.929496
ILMN_1680437	LOC731096	0.11614	1.8125	1.92864
ILMN_3190442	LOC100128591	-0.66941	1.25873	1.928136
ILMN_3256445	LOC100130289	-1.01483	-0.91308	1.927905
ILMN_1806733	COL18A1	-1.59508	-0.33252	1.9276
ILMN_1742467	C6orf138	-0.71397	1.21228	1.926247
ILMN_1678729	SIL1	1.74055	-0.1856	1.926151
ILMN_3254413	LOC100129426	0.564853	1.36051	1.925363
ILMN_1654722	MPV17L	0.171284	1.75347	1.924754
ILMN_3284177	LOC100132425	-1.3149	-0.60958	1.924475
ILMN_1726752	APTX	0.050413	-1.87339	1.9238025
ILMN_3214256	LOC128192	-0.4349	-1.48865	1.923554
ILMN_2378555	ACSL6	-0.33687	1.58665	1.92352
ILMN_3284521	LOC646572	0.300829	-1.62242	1.923249
ILMN_2161357	C6orf111	-0.10468	-1.81816	1.922836

ILMN_2341363	ATP5A1	0.948396	-0.97389	1.922285
ILMN_2121816	GPR137B	-0.30003	1.62223	1.922258
ILMN_2192683	DHX37	0.940597	-0.98156	1.922161
ILMN_1659553	ANAPC1	-0.02829	1.8936	1.9218853
ILMN_3265895	HNRNPR	-0.58363	-1.33811	1.921736
ILMN_3200384	LOC441013	1.14088	0.780843	1.921723
ILMN_3232270	CROCCL1	1.16452	-0.75665	1.921168
ILMN_1783023	C5orf51	1.53716	-0.38382	1.920978
ILMN_1663575	MGC87042	0.8619	-1.05788	1.91978
ILMN_1760727	ANG	0.49268	-1.42678	1.91946
ILMN_1696702	NEO1	-1.25506	-0.66347	1.918525
ILMN_2222992	ETF1	1.42098	-0.49679	1.917768
ILMN_1714412	ZNF292	1.61824	-0.2995	1.91774
ILMN_1797776	PRSS23	-0.67999	-1.23717	1.91716
ILMN_1815673	DKK3	0.328441	-1.58858	1.917021
ILMN_3287583	LOC648390	-0.81845	1.09765	1.916097
ILMN_2215824	ANKRD20A1	0.964947	-0.9511	1.916042
ILMN_1768197	ROD1	1.17796	-0.73803	1.915991
ILMN_3275531	LOC284685	0.799753	-1.11588	1.915633
ILMN_2049293	CD84	-1.43816	0.476627	1.914787
ILMN_1718924	ETFA	-0.63463	1.27979	1.914423
ILMN_2085339	FLJ35848	-1.78478	-0.12918	1.913964
ILMN_1753279	HNRNPA0	-0.74031	-1.1736	1.913906
ILMN_1673185	CPSF2	-0.31085	1.60285	1.9137
ILMN_1797933	MRPL17	0.88044	-1.0328	1.91324
ILMN_2410924	PLOD2	0.547506	-1.36553	1.913036
ILMN_1701331	UBE2M	0.552042	-1.36057	1.912612
ILMN_1809439	HMGB1L1	-0.74619	-1.16632	1.912514
ILMN_1747968	RBM33	0.997094	-0.91538	1.912471
ILMN_1787879	ARL2	-1.23461	-0.6774	1.912011
ILMN_1698334	LOC728863	1.24552	0.665636	1.911156
ILMN_1677043	AKR7A2	1.38366	-0.52673	1.910391
ILMN_1745820	RASSF6	-0.10993	-1.80034	1.910267
ILMN_1690237	PCDHB19P	-0.32692	1.58331	1.910233
ILMN_2320641	NBPF7	0.751134	-1.15902	1.910154
ILMN_2331087	MS4A7	1.38581	-0.52386	1.909671
ILMN_1812616	MYO1C	-1.72677	0.182239	1.909009
ILMN_2379469	EIF3B	1.63463	-0.27437	1.908999
ILMN_1709326	LOC23117	-0.80651	-1.10227	1.908781
ILMN_1784238	SEC22B	-0.24695	1.66182	1.908772
ILMN_3235853	S1PR1	0.658829	-1.24968	1.908509
ILMN_2192693	EIF3M	0.994628	-0.91383	1.908461
ILMN_1708204	COPG	0.925001	-0.98163	1.90663
ILMN_1661559	LOC129870	0.460214	-1.44508	1.905294
ILMN_1673409	MGC16121	-0.28045	1.62451	1.904963
ILMN_1783846	RAPH1	-1.00624	-0.89835	1.904587
ILMN_2359907	CD68	-0.56373	1.34081	1.904543
ILMN_1795442	LAMA4	-0.39766	-1.50685	1.904514
ILMN_3238854	RGPD8	0.667821	-1.23633	1.904151
ILMN_3301193	LOC728942	-0.06911	-1.83494	1.9040464
ILMN_3187648	LOC100128908	0.934391	0.969208	1.903599
ILMN_1688090	PTER	0.965583	-0.93778	1.903358

ILMN_1679727	CLK1	1.09752	-0.80536	1.90288
ILMN_3235880	LOC728725	0.612358	1.29038	1.902738
ILMN_1701857	LOC644373	0.266931	-1.63509	1.902021
ILMN_3251404	NUCKS1	0.65302	-1.24889	1.90191
ILMN_3287239	LOC642956	-1.00481	0.896944	1.901754
ILMN_3236713	SNHG1	-0.91879	-0.98245	1.901235
ILMN_3178680	LOC100128031	0.192053	-1.70867	1.900723
ILMN_2387078	MPZL2	-0.35103	-1.54953	1.900564
ILMN_1757106	Mar-06	-0.91115	-0.98933	1.900484
ILMN_1693766	CEP135	0.925399	-0.97476	1.900158
ILMN_3195372	LOC100128056	0.469127	-1.43103	1.900157
ILMN_2196588	C18orf32	0.964629	-0.93508	1.899706
ILMN_2164152	ENPEP	1.32589	-0.57308	1.898972
ILMN_2399489	CBFB	-1.45105	0.447874	1.898924
ILMN_2184869	ZMYM6	-0.71031	1.18856	1.898867
ILMN_3235657	SRGAP2L	-0.88402	-1.01478	1.898795
ILMN_2411963	RBM39	1.08367	-0.81511	1.898782
ILMN_3224555	LOC729397	0.715565	-1.18306	1.898625
ILMN_1762281	DCTN3	1.22125	-0.67735	1.898598
ILMN_2414325	TNFAIP8	0.577664	-1.32074	1.898404
ILMN_1666206	GSDMB	1.13356	-0.76412	1.897675
ILMN_3185144	LOC100128975	1.28467	-0.61298	1.897654
ILMN_1718853	UQCRC2	0.831741	-1.06587	1.897611
ILMN_2366703	SGK3	-0.55824	1.33936	1.897603
ILMN_1778238	UTP6	-0.45758	-1.43989	1.897473
ILMN_1728360	MED29	1.34952	0.547607	1.897127
ILMN_1772631	EPX	0.284676	-1.61216	1.896836
ILMN_1749410	PKP4	0.990251	-0.90633	1.896581
ILMN_1718769	ITSN1	0.335205	-1.56122	1.896425
ILMN_1688534	EIF2B5	-0.73569	1.1606	1.896291
ILMN_2168449	DHX15	-1.76632	0.129911	1.896231
ILMN_1776157	Sep-04	-1.38222	0.51351	1.89573
ILMN_3243291	LOC100133372	1.12187	-0.77312	1.894992
ILMN_1659782	STK19	0.54671	-1.34811	1.89482
ILMN_2391141	UBE3A	0.648325	-1.24645	1.894775
ILMN_3281684	LOC441253	0.83564	-1.05851	1.89415
ILMN_2339748	RNF13	1.04293	-0.85066	1.893594
ILMN_1698100	ANXA2P1	1.03951	-0.85397	1.893482
ILMN_2323087	RBPMS	0.313402	-1.58002	1.893422
ILMN_1661599	DDIT4	0.654055	1.23878	1.892835
ILMN_3248759	LOC732272	-1.75988	0.132628	1.892508
ILMN_1662103	LOC648751	0.575198	1.3173	1.892498
ILMN_1742798	SFRS10	-0.32661	1.56572	1.892325
ILMN_3223500	LOC728903	0.107864	1.78437	1.892234
ILMN_3221156	NBPF8	1.09075	-0.80143	1.892181
ILMN_2362902	RASSF5	-1.18886	-0.70305	1.891913
ILMN_2319344	APEX1	-0.34706	-1.54372	1.890775
ILMN_2286014	CATSPER2	-0.13969	1.75055	1.890238
ILMN_2281529	STAP2	-0.4291	1.46076	1.889855
ILMN_1717877	IVNS1ABP	-0.29215	-1.59766	1.88981
ILMN_1749213	SDF2L1	0.571537	-1.31721	1.888747
ILMN_1778377	ERGIC1	1.21337	-0.67485	1.888221

ILMN_1746435	HIST1H1E	-0.2261	-1.66199	1.888094
ILMN_3201221	LOC341315	1.54623	-0.3418	1.888034
ILMN_2352097	GPR56	0.529125	-1.35888	1.888005
ILMN_1698766	PYCARD	-0.4774	-1.41056	1.887956
ILMN_3294965	LOC100133211	0.469684	-1.41827	1.887954
ILMN_1776173	PSMD7	1.59135	-0.2964	1.88775
ILMN_1805726	LOC654069	-0.77436	1.11317	1.887533
ILMN_2221507	F2R	0.441594	-1.44565	1.887244
ILMN_3199454	LOC642305	-0.03179	1.85528	1.8870666
ILMN_1765725	SBDSP	0.839937	-1.04703	1.886967
ILMN_1691188	UIMC1	0.392179	-1.49451	1.886689
ILMN_1790136	C20orf20	-0.56771	-1.31877	1.886482
ILMN_1767448	LHFP	-0.5405	-1.34548	1.885978
ILMN_1682781	TEAD2	0.552758	-1.3329	1.885658
ILMN_2157435	DYNLRB1	1.09409	-0.7906	1.884686
ILMN_1752592	HLA-DRB4	-1.60582	-0.27875	1.884568
ILMN_2219556	ISCA1	0.181867	-1.70219	1.884057
ILMN_3195497	LOC100128309	-0.78073	-1.10324	1.883969
ILMN_3239610	LOC100133565	-0.92078	0.962953	1.883731
ILMN_2269564	ARID4B	1.26016	-0.62349	1.883647
ILMN_1810423	RPP40	0.256587	1.62671	1.883297
ILMN_1785330	SH3BP4	0.936619	-0.94637	1.882986
ILMN_1660806	CSRP2	-1.75351	0.12903	1.88254
ILMN_2218450	LSM1	1.72925	0.152924	1.882174
ILMN_2359453	ERGIC3	0.808586	-1.07304	1.881626
ILMN_1651958	MGP	0.286108	-1.59548	1.881588
ILMN_3248583	LOC100189589	0.128792	1.75202	1.880812
ILMN_2406043	VPS24	-0.9016	-0.97882	1.880425
ILMN_1656981	LOC388160	1.18107	0.699212	1.880282
ILMN_2099594	SRP9	0.631535	1.24859	1.880125
ILMN_1716014	LOC653232	-1.22117	-0.65838	1.879546
ILMN_1782377	LOC440354	-0.48018	-1.39923	1.879411
ILMN_1791280	HSPB8	1.09823	-0.7809	1.879127
ILMN_1812262	DDR1	0.777552	1.10142	1.878972
ILMN_3229038	LOC728310	0.445652	1.43298	1.878632
ILMN_1772946	STRN3	0.149402	-1.72915	1.878552
ILMN_1717990	CALD1	-1.04746	-0.82993	1.877389
ILMN_1737738	NDUFA12	0.685088	-1.19156	1.876648
ILMN_1662328	CNNM3	-1.68373	0.192571	1.876301
ILMN_2115669	SEMA4C	-0.27719	-1.59888	1.87607
ILMN_2041788	PLS3	-1.59619	-0.27923	1.875423
ILMN_1707627	TPI1	-0.80007	-1.07521	1.875275
ILMN_1730888	ZNF680	0.413027	-1.46156	1.874587
ILMN_3276697	LOC646956	-1.09942	0.775062	1.874482
ILMN_1651735	TGOLN2	-0.18473	1.68949	1.874216
ILMN_2389211	LAIR1	1.07134	0.802838	1.874178
ILMN_1699071	C21orf7	0.588725	-1.28459	1.873315
ILMN_3241396	LOC729277	1.08717	-0.78605	1.873215
ILMN_1789233	VPS37C	0.219426	-1.65375	1.873176
ILMN_1687375	ATP2A2	-1.36185	-0.51093	1.872775
ILMN_3287068	LOC728031	1.16006	-0.71229	1.87235
ILMN_3245635	LOC100133690	0.698854	-1.17321	1.872064

ILMN_1749244	LYRM1	1.22627	-0.64579	1.87206
ILMN_1687824	SEPHS2	1.48217	-0.38985	1.872018
ILMN_1778242	CALM1	0.383824	-1.48787	1.871694
ILMN_1815057	PDGFRB	-0.62874	-1.24283	1.871574
ILMN_1657632	ZMYM6	0.964867	-0.90632	1.871185
ILMN_3258795	FAM13B	-0.68262	-1.18839	1.871005
ILMN_1784540	KBTBD2	-1.27528	-0.59531	1.870588
ILMN_1720578	PRAF2	-1.01666	0.853894	1.870554
ILMN_2123312	LRAP	-0.22905	1.64142	1.870468
ILMN_1720819	LOC653566	-1.68922	0.180944	1.870164
ILMN_1801869	WDR75	0.528727	-1.34126	1.869987
ILMN_1706958	PCNA	-0.25125	1.61846	1.869709
ILMN_1744822	BECN1	-0.28157	-1.58797	1.869537
ILMN_1733164	FBXO11	-0.20589	1.66354	1.869433
ILMN_1779373	HIST1H2BF	0.23542	-1.63399	1.86941
ILMN_3248828	LOC100133758	1.63344	-0.23596	1.869399
ILMN_2150019	SUCLA2	0.788135	-1.08124	1.869375
ILMN_2073543	C15orf63	-0.00378	1.86518	1.8689568
ILMN_3307950	CDK5RAP3	-0.94537	-0.92357	1.868939
ILMN_1765371	LUC7L	-1.61975	-0.24891	1.868656
ILMN_1670172	WDR33	0.560954	-1.30748	1.868434
ILMN_3279092	LOC645691	1.11996	-0.74813	1.868092
ILMN_2200636	KIAA1267	-1.45257	0.415293	1.867863
ILMN_1679920	LOC651894	0.492441	-1.3753	1.867741
ILMN_1710413	MGC3032	-0.33655	1.53068	1.867232
ILMN_1691846	G0S2	0.052879	-1.81352	1.8663993
ILMN_1678651	LOC651022	1.38183	0.48455	1.86638
ILMN_2145997	SP4	0.8171	-1.04915	1.86625
ILMN_1767422	POLR1D	-0.55968	1.30618	1.865855
ILMN_1733110	RASSF7	-1.56596	0.299884	1.865844
ILMN_2383934	ITGB1	0.505173	-1.36065	1.865823
ILMN_1705750	TGM2	-1.31299	0.552554	1.865544
ILMN_2374293	DYRK1A	-1.625	0.240148	1.865148
ILMN_3310035	MIR2116	1.53732	-0.32752	1.86484
ILMN_1693334	P4HA1	0.657954	-1.20675	1.864704
ILMN_1723962	LXN	-0.0664	-1.79731	1.8637109
ILMN_1689318	NUAK1	-1.00624	-0.85709	1.86333
ILMN_1726617	ZNF182	-0.0411	-1.82167	1.8627666
ILMN_1735052	ULK1	-0.17558	1.68606	1.861635
ILMN_3274443	LOC728100	0.726546	1.13476	1.861306
ILMN_1688702	PJA2	0.949669	0.911495	1.861164
ILMN_1802053	ZNF91	0.504616	-1.35643	1.861046
ILMN_2100815	TMEM9B	1.14852	-0.71136	1.859875
ILMN_3283573	LOC647276	-0.24488	1.61415	1.859033
ILMN_1730678	ANKRD30B	0.006011	1.85295	1.8589607
ILMN_1717261	HLA-DRB3	-1.84804	-0.0108	1.8588397
ILMN_2162367	DMC1	0.171205	1.68711	1.858315
ILMN_1666615	PREPL	0.995026	-0.86256	1.85759
ILMN_2192351	GOLGA8E	0.918476	-0.93846	1.856937
ILMN_2363439	RYK	0.585781	-1.27096	1.856741
ILMN_2103397	ZNF234	-0.46276	-1.39356	1.856315
ILMN_2373495	H2AFY	-0.44278	-1.41302	1.855802

ILMN_2080158	FAM10A7	0.514324	-1.34136	1.855684
ILMN_2413084	HSPA8	-0.12258	-1.73284	1.85542
ILMN_3230508	NCRNA00152	1.10269	0.752303	1.854993
ILMN_1662640	C20orf127	-0.54567	-1.30892	1.85459
ILMN_3304678	LOC728671	-0.43522	1.41892	1.854142
ILMN_2053921	CAPZB	0.795615	-1.05846	1.854075
ILMN_3265761	LOC100128337	1.04937	-0.80462	1.85399
ILMN_1739161	PPAP2A	0.431648	-1.42123	1.852878
ILMN_2038777	ACTB	1.35509	-0.49767	1.85276
ILMN_1741134	WDR89	0.584587	1.26745	1.852037
ILMN_3296855	LOC389857	-0.80659	1.04542	1.85201
ILMN_1805606	PLEKHG1	0.908609	-0.94319	1.851798
ILMN_1794011	CHST2	0.765298	-1.08618	1.851478
ILMN_2203807	MRP63	-0.46307	1.38825	1.851323
ILMN_1658468	LOC652840	0.380402	-1.4708	1.851202
ILMN_3248712	SNORD67	0.860069	-0.99106	1.851125
ILMN_2371055	EFNA1	1.19125	-0.65986	1.851112
ILMN_3309514	MIR574	0.488861	-1.36217	1.851031
ILMN_2375141	C10orf4	-0.12346	1.7274	1.850855
ILMN_1790354	RABGGTB	0.992877	0.857971	1.850848
ILMN_3269369	LOC100128562	-0.82728	1.02347	1.850749
ILMN_1798212	LLGL1	0.937096	-0.9128	1.849899
ILMN_1655154	PTBP1	1.19818	-0.65168	1.849864
ILMN_3305899	LOC728572	0.884817	-0.96496	1.849777
ILMN_2408440	TRAPPC2	1.62604	0.223656	1.849696
ILMN_2230892	IL10RB	1.11757	0.7319	1.84947
ILMN_1702837	PSMD1	0.766571	-1.08276	1.849331
ILMN_1784141	JAKMIP1	1.48289	-0.36626	1.849151
ILMN_1685855	UBN2	1.14144	0.707579	1.849019
ILMN_1710758	RNF20	0.311333	-1.53734	1.848673
ILMN_1736130	C18orf22	0.92707	0.921217	1.848287
ILMN_3298410	LOC729120	-0.85903	0.989209	1.848238
ILMN_1660021	M6PRBP1	0.868425	-0.97972	1.848149
ILMN_3237907	EIF2B3	-0.31332	1.53452	1.847836
ILMN_2104106	XPR1	-0.98014	-0.86756	1.847698
ILMN_1740430	SLC2A4RG	-0.56962	-1.27807	1.847692
ILMN_1810467	PPP2R1A	1.23939	-0.60731	1.846699
ILMN_2131467	GNAI1	-0.17104	-1.67502	1.84606
ILMN_3215374	LOC257396	-1.5923	-0.25365	1.845952
ILMN_1795338	YPEL1	0.103965	-1.74154	1.845505
ILMN_2059294	RTCD1	0.718509	-1.12696	1.845469
ILMN_3281383	LOC100131722	0.627557	-1.21711	1.844667
ILMN_1685988	LOC441958	-0.88776	-0.95688	1.844631
ILMN_2332368	VPS13B	1.20955	-0.63497	1.844523
ILMN_1652996	SMG1	0.231521	1.61255	1.844071
ILMN_1812236	LOC650739	1.18242	0.661648	1.844068
ILMN_2363621	RBBP8	0.847258	-0.9967	1.843959
ILMN_3207694	LOC388907	0.719702	-1.12424	1.843942
ILMN_1801421	EMD	-0.40029	1.44362	1.84391
ILMN_1701229	RBM12	0.885135	-0.95869	1.843828
ILMN_2224907	C4orf34	-0.76768	1.07607	1.843749
ILMN_3187429	LOC100130071	1.42528	0.418026	1.843306

ILMN_1658176	ABCG1	-1.7181	0.125118	1.843218
ILMN_1689400	CLK1	0.922057	-0.92085	1.842908
ILMN_3296943	LOC100131096	-0.18664	1.65618	1.842816
ILMN_2047885	PCDHB9	0.307752	1.53486	1.842612
ILMN_1755808	LOC654194	0.204864	1.6374	1.842264
ILMN_2324574	RALGAPA1	0.123461	-1.7187	1.842161
ILMN_1711009	ISLR	1.253	-0.58914	1.842142
ILMN_2167426	ASPHD2	-0.46562	1.37594	1.841559
ILMN_1708728	H2AFJ	-0.91815	-0.92306	1.84121
ILMN_2072973	TOP3A	-0.12815	1.71286	1.84101
ILMN_1666049	NUP214	1.03155	-0.80935	1.840904
ILMN_3295109	LOC653557	1.34379	-0.49657	1.840359
ILMN_2398865	VPS13C	0.274252	-1.56593	1.840182
ILMN_1744487	C1QTNF5	-0.88219	-0.95744	1.839629
ILMN_3240773	LOC100129445	1.50835	-0.33124	1.839586
ILMN_1780699	THAP11	0.79848	1.04025	1.83873
ILMN_1708382	C3orf75	1.15576	0.682939	1.838699
ILMN_1762330	C1D	1.03187	-0.80662	1.838485
ILMN_2414007	NME2	0.220381	-1.61794	1.838321
ILMN_1734153	GDI1	0.940438	-0.89784	1.838276
ILMN_1729453	TSPAN9	-1.82799	-0.00943	1.83742278
ILMN_2269136	AGAP3	0.67347	1.16363	1.8371
ILMN_2407389	GPNMB	0.129747	-1.70713	1.836877
ILMN_1724504	SETD3	0.730683	-1.10614	1.836823
ILMN_3249578	LOC100132394	-1.21687	-0.61987	1.836736
ILMN_1663866	TGFBI	0.898503	-0.93817	1.836668
ILMN_1727165	LOC644852	-0.46188	1.37447	1.836349
ILMN_1664216	NKIRAS1	0.910121	-0.92586	1.835978
ILMN_2363658	PXDN	0.23725	-1.59845	1.8357
ILMN_3302937	LOC729793	-0.09751	-1.73796	1.835474
ILMN_3234615	LOC728650	-1.21305	-0.62165	1.834697
ILMN_3236812	LOC100134199	0.651826	1.18273	1.834556
ILMN_1725090	CTHRC1	0.512653	-1.32189	1.834543
ILMN_1814156	PSMB7	0.098077	1.73636	1.8344368
ILMN_2112673	FKSG30	-0.38087	1.45297	1.833844
ILMN_3217262	LOC642812	0.717633	1.11522	1.832853
ILMN_2266595	LILRA5	0.541777	-1.29086	1.832637
ILMN_2101651	MBTD1	0.098395	1.73422	1.8326151
ILMN_2054607	CYP4V2	0.316823	-1.51514	1.831963
ILMN_1670410	LOC641710	0.8619	0.969717	1.831617
ILMN_1746664	WSB2	0.020175	-1.81121	1.8313847
ILMN_2273103	ELK4	0.571458	-1.25935	1.830808
ILMN_2355225	LSP1	-1.35191	-0.47876	1.830668
ILMN_1696029	FLJ36031	-0.3492	1.48135	1.830554
ILMN_3278906	LOC644464	1.05097	-0.77944	1.830412
ILMN_2091084	C8orf37	0.434114	1.39623	1.830344
ILMN_1763556	LOC654000	-0.79442	-1.03575	1.830166
ILMN_2326997	DHPS	0.65111	-1.17899	1.8301
ILMN_3239361	LOC100133298	0.04532	1.78391	1.8292298
ILMN_1689446	EIF3G	0.929378	-0.89915	1.82853
ILMN_3274470	LOC100133607	-1.3126	-0.51593	1.828525
ILMN_1754234	ZMYND11	-0.75734	-1.07091	1.828245

ILMN_2129102	FLJ40453	0.167385	-1.65998	1.827365
ILMN_1667081	CCND2	0.997015	-0.83017	1.827181
ILMN_1752351	LAMP2	0.859751	-0.9672	1.826953
ILMN_1802550	PCSK6	-0.05844	1.7685	1.8269436
ILMN_1651899	LOC653314	-0.00354	1.8234	1.82693808
ILMN_1783142	RPS4Y1	0.678165	-1.14863	1.826795
ILMN_2113470	GSTA1	0.563978	-1.26269	1.826668
ILMN_1781386	WIPI1	0.793546	-1.03266	1.826206
ILMN_3251560	TMEM167A	0.625169	-1.20018	1.825349
ILMN_1814573	FTSJD1	1.01508	0.8102	1.82528
ILMN_2390586	SP100	1.39997	-0.42522	1.825187
ILMN_3290562	LOC131691	-0.77134	-1.05374	1.82508
ILMN_1782273	N4BP2	-0.9398	0.885115	1.824911
ILMN_1687403	MRPL40	1.47254	0.35236	1.8249
ILMN_1666512	SKIV2L	0.946088	-0.87865	1.824736
ILMN_2399503	UBN1	0.627159	-1.19662	1.823779
ILMN_1713978	SDF2	-0.31928	1.50439	1.823674
ILMN_1750395	MBD2	-0.55785	-1.26579	1.823635
ILMN_3304584	LOC729739	0.325417	-1.49805	1.823467
ILMN_1791702	SMARCA2	0.526658	-1.29638	1.823038
ILMN_1727332	ATPIF1	1.60838	0.21449	1.82287
ILMN_2383150	MCHR2	0.010467	1.81233	1.8227968
ILMN_1770732	COPS3	0.623419	-1.19929	1.822709
ILMN_1652161	PNKD	1.33966	0.482651	1.822311
ILMN_3238797	FAM72A	0.651508	1.17027	1.821778
ILMN_2081465	APLP2	0.346345	-1.47543	1.821775
ILMN_3271724	LOC100129159	1.4057	-0.41593	1.821632
ILMN_1751644	LIMS1	1.03696	-0.78455	1.821514
ILMN_2181363	UBE3C	0.584746	-1.23452	1.819266
ILMN_1656656	COX19	-0.30138	1.5175	1.81888
ILMN_1808984	CXorf48	-1.38517	-0.43226	1.817429
ILMN_1710192	LOC440345	0.338786	-1.47841	1.817196
ILMN_1713884	C16orf42	1.00855	-0.80864	1.817194
ILMN_1782050	CEBPD	1.05884	-0.75792	1.81676
ILMN_1721921	BLMH	1.51639	0.300109	1.816499
ILMN_2322552	NCKAP1	-1.24122	-0.57415	1.815367
ILMN_1765043	RPL38	0.542175	1.27312	1.815295
ILMN_2374164	HERPUD1	-0.97425	0.840799	1.81505
ILMN_1706275	C8orf33	-1.2787	0.536204	1.814904
ILMN_1791057	IFNAR2	0.49276	-1.32205	1.81481
ILMN_3175662	LOC100130107	1.43753	-0.37722	1.81475
ILMN_1655821	CAPG	0.195236	-1.61885	1.814086
ILMN_1691702	ZNF775	0.816623	0.997103	1.813726
ILMN_1667381	CAMKV	-1.22379	0.58813	1.81192
ILMN_2291619	RAB3IP	1.21369	-0.59807	1.811756
ILMN_3301440	LOC730004	-0.48416	1.32745	1.81161
ILMN_1690063	LOC651143	1.61204	-0.19919	1.811228
ILMN_3247732	ZRSR2	0.477482	-1.33365	1.811132
ILMN_1651499	ERGIC1	-0.01929	1.79142	1.8107136
ILMN_2330994	NKTR	0.118209	-1.69243	1.810639
ILMN_1762204	C1orf150	1.54814	0.261853	1.809993
ILMN_1739885	SLC41A3	-0.394	1.41593	1.809934

ILMN_1671291	EIF3I	0.61952	1.19013	1.80965
ILMN_1697548	LPHN2	0.980066	0.828899	1.808965
ILMN_1760741	NDUFA9	0.529045	1.27961	1.808655
ILMN_2048793	CIAO1	0.323428	1.4852	1.808628
ILMN_1653028	COL4A1	-0.75805	-1.05021	1.808261
ILMN_1693826	HAVCR2	-1.5202	0.287706	1.807906
ILMN_3230260	LOC729348	-0.78359	-1.02383	1.807424
ILMN_1768913	TSN	-1.48662	-0.32074	1.807364
ILMN_1691376	JAG1	-0.16515	-1.64212	1.807271
ILMN_1787762	HEATR1	1.20207	-0.60508	1.807148
ILMN_1801077	PLIN2	-0.00895	1.79795	1.80689906
ILMN_1679460	PPFIBP1	-1.61108	-0.19522	1.806303
ILMN_1718960	SERPINB8	-0.2795	-1.52652	1.806018
ILMN_1747058	TRAPPC2L	0.927309	-0.87834	1.805649
ILMN_1767665	LOC493869	0.736651	-1.06874	1.805391
ILMN_1717262	PROCR	1.22372	-0.58166	1.805377
ILMN_2279961	LAMP2	0.493476	-1.31188	1.805356
ILMN_3268924	GIGYF2	-1.60192	-0.20332	1.805244
ILMN_1707175	NSD1	-1.75057	-0.05435	1.8049163
ILMN_2053527	PARP9	0.608857	-1.19573	1.804587
ILMN_1736940	HPRT1	0.402126	1.40225	1.804376
ILMN_3185429	LOC100129842	0.409049	-1.39519	1.804239
ILMN_1743620	RARRES1	-0.65349	1.15021	1.803702
ILMN_1727813	BRP44	1.34451	-0.45865	1.80316
ILMN_1674069	TOMM7	-1.35071	-0.45206	1.802768
ILMN_2383489	CDK5RAP3	-0.2935	-1.50918	1.802683
ILMN_1657470	YTHDF3	-1.23676	-0.5659	1.802658
ILMN_3176090	LOC100130919	0.972665	-0.82971	1.802375
ILMN_2162989	TMEM189	-0.68779	1.11421	1.801998
ILMN_1739428	IFIT2	0.574959	-1.22698	1.801939
ILMN_1652487	RIOK1	-0.39257	-1.40924	1.801811
ILMN_1795251	SPARCL1	1.61212	-0.18968	1.801804
ILMN_2343278	PPAP2A	0.457191	-1.34416	1.801351
ILMN_3227563	LOC729255	-0.98324	0.817904	1.801147
ILMN_1761808	MCFD2	-0.2577	1.54327	1.800965
ILMN_3188976	LOC100130211	0.36592	-1.43486	1.80078
ILMN_1776483	LOC728417	0.840256	0.96042	1.800676
ILMN_1791466	HMGB1	-0.52904	-1.27156	1.800599
ILMN_1783220	LOC648668	0.950146	-0.85001	1.800153
ILMN_3273854	HNRNPA2B1	0.016117	-1.7839	1.8000165
ILMN_1707336	ARPC4	0.639413	-1.16041	1.799823
ILMN_1772663	KIAA0692	-0.41907	1.38073	1.799799
ILMN_1744713	PARK7	-1.38135	-0.41752	1.798874
ILMN_3268914	LOC100128410	-0.49061	1.30796	1.798565
ILMN_1778173	AK3	0.506526	-1.29158	1.798106
ILMN_1723300	FAM70B	0.591351	-1.20662	1.797971
ILMN_1651285	BCL6B	-0.58936	-1.20805	1.797406
ILMN_1800055	LOC647295	0.888318	-0.90882	1.797133
ILMN_2328813	DMAP1	1.18043	-0.61652	1.796952
ILMN_2170625	LOC389286	0.955796	-0.84052	1.796318
ILMN_1759628	ATP1B3	0.054391	1.7416	1.7959912
ILMN_3220792	LOC728658	-0.31722	1.47827	1.795485

ILMN_3187470	LOC100129094	0.331545	1.46382	1.795365
ILMN_1731931	LOC727773	-0.31682	1.47844	1.795258
ILMN_2160209	TACSTD1	1.26987	0.524795	1.794665
ILMN_1800787	RFTN1	0.708721	-1.08559	1.794311
ILMN_1657680	CCDC69	0.738959	-1.05481	1.793769
ILMN_1688490	PTP4A2	-0.28801	1.50572	1.793732
ILMN_1768470	EIF4G1	0.280459	-1.51299	1.793449
ILMN_1666111	YTHDC1	0.389553	-1.40376	1.793313
ILMN_1662741	EDG4	0.55045	-1.24174	1.79219
ILMN_2323302	SON	0.611483	-1.18034	1.791823
ILMN_3243419	LOC100130715	0.668378	-1.12336	1.791738
ILMN_2359800	MS4A6A	1.18314	0.608255	1.791395
ILMN_2297096	ARHGAP8	-0.17136	1.61961	1.790968
ILMN_3217285	LOC389322	-0.50755	1.28313	1.790684
ILMN_2130078	CDKN2AIPNL	-0.28658	1.50363	1.79021
ILMN_2149952	FLJ45256	-0.48583	1.30424	1.790071
ILMN_1710406	PSMC1	-0.54535	1.24438	1.789732
ILMN_1662312	LOC653720	-0.23836	1.55104	1.789398
ILMN_1769264	MCCC2	-0.15425	-1.63496	1.78921
ILMN_2229649	KCTD12	0.729967	-1.05916	1.789127
ILMN_2178186	PIGW	-0.51281	1.27557	1.788376
ILMN_3243209	LOC100133463	0.572174	-1.2162	1.788374
ILMN_1738976	OR2A20P	0.650633	-1.13755	1.788183
ILMN_2228196	C6orf170	0.01166	1.77616	1.7878204
ILMN_1729216	CRYAB	1.12553	-0.66203	1.787557
ILMN_3236746	LOC100132184	0.290485	-1.49683	1.787315
ILMN_1685365	ZNF773	0.223405	1.56249	1.785895
ILMN_2207539	RPS17	-1.47174	0.313571	1.785311
ILMN_3187206	FLJ11827	0.542016	-1.2432	1.785216
ILMN_1686367	HSPA8	-0.80874	-0.97597	1.784711
ILMN_1664855	PPP1R14C	0.418041	-1.3659	1.783941
ILMN_3216365	LOC100131096	0.287302	1.49652	1.783822
ILMN_1746243	TES	0.444857	-1.33872	1.783577
ILMN_1678814	UBL5	-0.30027	1.4831	1.783366
ILMN_1735996	NOX4	0.303773	-1.47946	1.783233
ILMN_1803810	RRBP1	1.73872	-0.04439	1.7831072
ILMN_3235264	SNORA48	-1.47214	0.310335	1.782475
ILMN_1739441	GANAB	0.709278	-1.07302	1.782298
ILMN_1813028	CBX5	-0.52546	-1.25683	1.782288
ILMN_1716907	FLJ20254	1.39599	0.386196	1.782186
ILMN_3211677	LOC100132526	-0.12505	1.65714	1.782186
ILMN_2051900	EID2B	-0.55029	1.23176	1.782045
ILMN_2217955	TTC21B	-0.26947	1.51247	1.781942
ILMN_2381197	RNF19A	0.501035	-1.28077	1.781805
ILMN_3300891	LOC729652	-0.26414	-1.51731	1.78145
ILMN_2155322	ZNF652	0.545994	-1.23448	1.780474
ILMN_3240981	LOC100134587	0.584348	-1.19589	1.780238
ILMN_1733559	LOC100008589	0.295339	-1.48473	1.780069
ILMN_2212354	WDR46	0.853385	0.926561	1.779946
ILMN_2402392	COL8A1	0.661057	-1.11887	1.779927
ILMN_1756022	HIST1H2AM	1.51114	-0.26863	1.779769
ILMN_3284631	LOC100132496	0.293588	-1.48591	1.779498

ILMN_3201975	LOC392285	-0.73482	-1.04453	1.779345
ILMN_1766499	HSPA2	1.11088	-0.66801	1.77889
ILMN_3186390	LOC100128510	0.924126	-0.85466	1.778784
ILMN_1675674	UBE4B	0.313879	-1.46475	1.778629
ILMN_1685012	EAF1	0.270671	-1.50782	1.778491
ILMN_1793990	ID2	0.35287	-1.42546	1.77833
ILMN_2371397	SLC25A45	-0.15306	1.62525	1.778306
ILMN_2148290	PDCD7	-0.51464	1.26322	1.777857
ILMN_1753782	ZNF266	-1.17684	0.600935	1.777775
ILMN_1673899	FAM57A	0.179719	1.59771	1.777429
ILMN_1670305	SERPING1	-1.64028	0.136941	1.777221
ILMN_1677138	POLR2J3	-0.35549	-1.42161	1.7771
ILMN_2151281	GABARAPL1	1.22189	-0.55513	1.777019
ILMN_1737163	SH3BGRL3	-0.88688	-0.88995	1.77683
ILMN_2196479	XRN2	-0.53318	1.24329	1.776467
ILMN_1711766	SKP1A	-1.04547	0.730859	1.776329
ILMN_2215862	SLFN13	0.482495	-1.29329	1.775785
ILMN_2345872	SUMF2	0.727341	-1.04823	1.775571
ILMN_2201668	SLC19A2	0.715724	1.05974	1.775464
ILMN_1805668	ZNF486	-0.58944	1.18599	1.775425
ILMN_1773764	CECR7	0.89532	-0.87962	1.774944
ILMN_3292224	LOC100131609	1.13078	-0.64407	1.774848
ILMN_1681304	PAN3	0.932242	-0.84214	1.774379
ILMN_1751871	STK11	0.723283	-1.0505	1.773783
ILMN_1752832	LOC729602	1.43976	0.333827	1.773587
ILMN_3238640	LOC100133812	0.40507	1.36846	1.77353
ILMN_1758705	IRX6	0.171682	-1.60175	1.773432
ILMN_1776493	MTUS1	0.694955	-1.07827	1.773225
ILMN_1661432	NUP43	0.862059	-0.91077	1.772827
ILMN_1768855	C15orf37	-1.21902	-0.55276	1.771776
ILMN_1660775	LOC650152	0.464591	1.30716	1.771751
ILMN_1792265	TRIM4	0.067362	1.7043	1.7716616
ILMN_2372403	ALDH5A1	0.525305	-1.24602	1.771325
ILMN_1653797	C6orf62	-0.1373	1.63354	1.770841
ILMN_1652003	GNG10	0.455917	-1.31423	1.770147
ILMN_1729188	HAMP	0.88227	-0.8877	1.769971
ILMN_1729051	MSH6	0.474856	-1.29471	1.769566
ILMN_1671039	GALNT3	0.060041	1.70908	1.7691209
ILMN_3239820	LOC100134766	1.06521	0.703425	1.768635
ILMN_2147440	MAN2A1	1.41286	-0.35556	1.768422
ILMN_3244319	CCDC125	-0.52514	1.24319	1.76833
ILMN_1697922	LOC645671	0.468569	-1.29943	1.767999
ILMN_1726210	GPIHBP1	0.505491	-1.26239	1.767881
ILMN_1785711	NEDD8	1.12513	-0.64238	1.767511
ILMN_1804798	BEXL1	-0.03927	-1.72812	1.7673864
ILMN_1812769	UBXN1	0.694955	-1.07238	1.767335
ILMN_3188076	LOC100128060	-0.04253	-1.72415	1.7666789
ILMN_1771738	ARL5A	1.05232	0.713751	1.766071
ILMN_3300744	LOC728666	1.57448	0.190767	1.765247
ILMN_1695246	KLHDC8B	0.347459	-1.41736	1.764819
ILMN_1806003	SPEF2	-1.34037	-0.42411	1.76448
ILMN_1705114	NUMB	0.631535	-1.13274	1.764275

ILMN_2355033	KIAA1147	0.180594	-1.58368	1.764274
ILMN_1652768	LOC440589	1.04126	0.722604	1.763864
ILMN_1765557	OLFML2B	-0.82171	-0.94201	1.763721
ILMN_1759252	ADD1	0.004499	-1.75906	1.76355881
ILMN_1805345	MOSPD3	0.22985	-1.53342	1.76327
ILMN_3192934	LOC100130420	0.414062	1.34904	1.763102
ILMN_1675124	DDX17	-0.37594	-1.38676	1.7627
ILMN_1801121	SENP2	0.670447	1.09208	1.762527
ILMN_2067520	SNORD116-4	-0.49482	-1.26742	1.762243
ILMN_2276000	CPNE1	-0.46236	1.29928	1.761637
ILMN_3249244	TMEM106A	-0.16507	1.59642	1.761492
ILMN_3283869	LOC100134091	1.24043	-0.52089	1.76132
ILMN_1736939	UGCG	-1.55132	0.209921	1.761241
ILMN_1667257	SDHB	1.50215	0.258953	1.761103
ILMN_1691428	PSMD12	-0.57113	1.18973	1.760863
ILMN_1676563	HTRA1	0.45361	-1.30696	1.76057
ILMN_3199647	LOC645251	-1.32286	-0.4374	1.760261
ILMN_1709650	LOC652815	-1.37856	0.381504	1.760064
ILMN_2063114	TAF1D	1.06648	0.693271	1.759751
ILMN_2223922	AGTPBP1	1.33815	-0.42154	1.759686
ILMN_3246209	SCARNA10	0.566683	-1.19228	1.758963
ILMN_1671865	LOC147710	-0.63543	1.12346	1.758889
ILMN_1704754	LOC651017	-0.83062	0.928229	1.758851
ILMN_2070052	LOC613037	1.55212	-0.2066	1.758722
ILMN_2082273	RGS5	-0.29669	-1.46201	1.758696
ILMN_2173835	FTHL3	-1.65333	0.105289	1.758619
ILMN_3239734	LOC100134822	0.858478	0.90008	1.758558
ILMN_3304396	LOC730202	-0.60742	1.15106	1.758479
ILMN_2054233	SENP6	0.614029	1.14425	1.758279
ILMN_1784661	TMEM2	-1.09425	-0.66395	1.758195
ILMN_1666276	LOC643451	0.711506	1.04606	1.757566
ILMN_1760757	BRIP1	-0.25077	1.50668	1.757452
ILMN_1738678	C9orf130	0.420666	1.33627	1.756936
ILMN_3293146	LOC642975	0.532626	-1.22424	1.756866
ILMN_1768534	BHLHB2	-0.63034	-1.12637	1.756706
ILMN_2312897	DGUOK	0.35478	-1.40191	1.75669
ILMN_1716435	ATF7IP	-1.39416	-0.36224	1.756403
ILMN_3244963	WDR42A	-1.69144	-0.06471	1.7561477
ILMN_2138530	LOC651921	0.292315	1.46369	1.756005
ILMN_1751072	SRPRB	0.876223	-0.87975	1.755977
ILMN_2133360	LOC91561	1.43339	-0.32251	1.755898
ILMN_1694980	NAGLU	-1.09783	0.657299	1.755129
ILMN_1815010	RNF141	1.04444	-0.71017	1.754612
ILMN_1807533	C17orf71	-1.19626	0.558276	1.754536
ILMN_2333319	PTBP1	1.12744	-0.62691	1.754353
ILMN_1664175	VAMP4	-0.15719	-1.59706	1.754254
ILMN_3307729	CXXC5	0.554668	-1.19954	1.754208
ILMN_2362982	ARID4B	1.17828	-0.57546	1.753741
ILMN_1692145	ZNF14	-0.11009	1.64364	1.753727
ILMN_2395728	HNRPUL1	-0.25579	1.49783	1.753615
ILMN_2396272	PDCD4	0.364249	1.38936	1.753609
ILMN_1758633	CCDC130	0.569468	-1.18398	1.753448

ILMN_3236259	PPIAL4A	-0.79808	-0.95506	1.753136
ILMN_1679025	LOC641848	1.32756	-0.42505	1.752605
ILMN_1651385	MFN2	-0.25897	1.49331	1.752278
ILMN_1655622	PRKRIR	-1.13674	-0.6152	1.751943
ILMN_2116242	ZNF808	-0.56103	1.19059	1.751618
ILMN_3250970	DAZ2	0.126803	-1.62416	1.750963
ILMN_2223130	SMARCA5	1.31435	0.436394	1.750744
ILMN_1688749	LOC400963	1.0633	0.687276	1.750576
ILMN_2360784	RRBP1	1.57137	0.179044	1.750414
ILMN_2120210	RCAN2	0.602969	-1.14744	1.750409
ILMN_1803939	YIPF6	1.22706	-0.52324	1.750299
ILMN_1688318	MGC72104	-0.72678	-1.02343	1.750209
ILMN_2122953	CISD1	0.215766	-1.53385	1.749616
ILMN_1694882	AMY2B	1.29032	-0.45925	1.749568
ILMN_3247882	ERI3	1.11359	-0.63588	1.749468
ILMN_1653527	LOC648358	-0.4774	1.27177	1.749166
ILMN_1755910	LOC648366	0.35852	-1.38968	1.7482
ILMN_1705213	TMBIM1	-0.31642	1.43165	1.74807
ILMN_1661161	SIAH1	0.146378	1.60169	1.748068
ILMN_2404688	NUPR1	-1.00019	-0.74771	1.747902
ILMN_1769118	Sep-09	0.757261	-0.9903	1.747559
ILMN_1769783	ZDHHC2	-0.98754	-0.75997	1.747513
ILMN_1676611	PHPT1	0.521804	-1.22538	1.747184
ILMN_1654998	SMEK1	0.849725	-0.89726	1.746983
ILMN_2061435	MEG3	-0.22356	1.52341	1.746968
ILMN_3247283	LOC100134703	0.878053	0.868842	1.746895
ILMN_2179083	LOXL4	0.760523	-0.98593	1.746448
ILMN_3246885	LOC100132518	-0.37212	1.37432	1.746441
ILMN_2264011	GRAP	0.193326	-1.55283	1.746156
ILMN_1679195	C20orf24	0.039272	-1.70681	1.7460823
ILMN_1728785	GPR116	0.716758	-1.02932	1.746078
ILMN_1798354	PAPOLA	-0.50612	-1.23988	1.746002
ILMN_1718424	MRPS28	0.542254	-1.20326	1.745514
ILMN_2280568	ANKRD36	-0.04062	-1.70468	1.7452992
ILMN_2379762	NPM1	0.585542	-1.15869	1.744232
ILMN_1721659	LOC643668	0.759171	-0.98483	1.743996
ILMN_2343010	BOLA3	-0.15727	-1.58655	1.743823
ILMN_1666192	DCTN5	0.64562	-1.09801	1.74363
ILMN_1709722	FKSG83	1.16054	-0.58304	1.743575
ILMN_1659047	HIST2H2AA3	0.231362	-1.51202	1.743382
ILMN_2291407	PDE4DIP	-0.81264	0.930395	1.743033
ILMN_2159384	SBDSP	1.07404	-0.66808	1.742121
ILMN_1796743	LOC652335	0.068874	-1.67315	1.7420235
ILMN_2369924	NDUFB6	0.847179	0.894133	1.741312
ILMN_1696974	ANG	1.08287	-0.65775	1.740619
ILMN_1745620	KRCC1	-1.006	0.734492	1.740492
ILMN_3208014	LOC100131866	1.27743	-0.46302	1.740448
ILMN_3191922	KRT8P9	0.758375	-0.98204	1.740412
ILMN_2222163	HIST1H3B	1.53509	-0.20525	1.740343
ILMN_1749478	TCEAL3	1.12354	-0.61653	1.740068
ILMN_1671621	PCMT1	1.28149	-0.45819	1.739679
ILMN_1758846	ANKRD40	0.613472	1.12615	1.739622

ILMN_1793433	RAB10	-1.69582	0.043801	1.7396206
ILMN_1796339	PLEKHA2	-0.36774	1.37149	1.739234
ILMN_1659894	SLC25A26	0.357565	-1.38157	1.739135
ILMN_1685260	DNM1L	0.518621	1.22046	1.739081
ILMN_1675100	EDNRB	0.471355	-1.26759	1.738945
ILMN_3263968	LOC100129667	0.63432	-1.10428	1.7386
ILMN_1779677	ZCCHC6	0.32494	-1.41309	1.73803
ILMN_1666746	LOC153561	0.774767	-0.96311	1.73788
ILMN_2165369	HIST1H4B	0.516234	-1.22135	1.737584
ILMN_2298936	THOC3	0.759807	0.977498	1.737305
ILMN_1690443	C14orf82	0.17025	1.56689	1.73714
ILMN_1724497	ABI2	-0.87463	0.86225	1.736875
ILMN_2196232	C1orf210	-0.39225	1.34442	1.736673
ILMN_1695797	NCOA6	0.665991	1.0706	1.736591
ILMN_2366634	PKM2	1.25443	-0.48201	1.736437
ILMN_1673478	C5orf5	1.33337	-0.40263	1.736
ILMN_2371685	UBE2E1	0.305683	1.43013	1.735813
ILMN_1658289	WDR54	-0.28674	-1.44884	1.735579
ILMN_1740170	CHCHD10	0.644108	1.09056	1.734668
ILMN_3254666	LOC100129842	0.229691	-1.50486	1.734551
ILMN_3225432	MAPKSP1	0.798798	0.935502	1.7343
ILMN_1661266	HLA-DQB1	0.796252	-0.93761	1.733861
ILMN_1686725	DICER1	0.457827	1.27573	1.733557
ILMN_1691364	STAT1	-0.44795	-1.28541	1.733364
ILMN_2099586	CCDC28B	0.493237	1.23993	1.733167
ILMN_1807372	ADORA2A	0.848372	0.884127	1.732499
ILMN_2329735	ECM1	-0.30512	1.42725	1.73237
ILMN_1713143	MRPL3	0.891899	-0.84003	1.731929
ILMN_1701832	RPL19	0.378652	1.35297	1.731622
ILMN_3201365	LOC442232	-0.15489	1.57654	1.731426
ILMN_3179324	LOC100128461	1.41247	0.318714	1.731184
ILMN_1691466	YES1	-0.11773	1.61339	1.731116
ILMN_3225941	LOC728368	-1.09321	-0.63781	1.731023
ILMN_1685625	UCP2	0.483211	-1.24779	1.731001
ILMN_1807667	C14orf105	0.980225	0.750546	1.730771
ILMN_1775937	DDB1	1.07006	-0.66057	1.730626
ILMN_1814204	C21orf55	-0.7076	1.02302	1.730621
ILMN_2197101	DDX19A	0.579176	1.15122	1.730396
ILMN_2048326	RPS27A	0.787737	0.942632	1.730369
ILMN_1798874	TMEM85	1.03211	-0.69815	1.730258
ILMN_1701293	COX7A2	0.943621	-0.78661	1.730235
ILMN_1683609	UBE1	0.259372	-1.47026	1.729632
ILMN_3234605	LOC100131360	-0.70402	-1.0255	1.729521
ILMN_1666096	ACSL3	1.64562	-0.08315	1.7287665
ILMN_2370573	XAF1	0.344197	1.38445	1.728647
ILMN_1771966	BCCIP	-0.6714	1.05699	1.728386
ILMN_3187619	LOC100127984	-1.27034	-0.4576	1.727943
ILMN_1729019	Sep-07	-1.27281	-0.45504	1.727845
ILMN_1794085	SAPS1	0.798082	-0.92943	1.727507
ILMN_1736819	LOC202134	-0.70012	-1.02725	1.727372
ILMN_3225712	LOC730861	-0.26653	1.46083	1.727357
ILMN_1812403	BCAP31	-0.11247	-1.61411	1.726584

ILMN_2234956	LEPR	0.456554	-1.26999	1.726544
ILMN_1745785	NR2F2	-0.58156	-1.14444	1.725998
ILMN_1655868	ANP32C	1.10293	0.622492	1.725422
ILMN_1694399	ICA1	0.265101	1.46019	1.725291
ILMN_3246962	CYTSA	1.17574	-0.54947	1.725212
ILMN_1680239	NUDT9	0.815429	0.909589	1.725018
ILMN_1803143	SYVN1	0.313402	1.41105	1.724452
ILMN_1813650	INPP5F	0.633286	1.09112	1.724406
ILMN_3289685	LOC645452	-0.31523	1.40844	1.723666
ILMN_1726967	TWSG1	0.73315	0.990463	1.723613
ILMN_1779813	FAM96B	0.492282	-1.23121	1.723492
ILMN_1666609	USP22	-0.94194	0.781299	1.723243
ILMN_1783717	DUXAP3	1.30918	0.413754	1.722934
ILMN_3305628	LOC728678	0.982135	-0.74064	1.72277
ILMN_1717594	DKFZp761E198	-0.0999	-1.62286	1.7227612
ILMN_3199737	LOC727865	-0.86213	0.860486	1.722619
ILMN_3229770	SKP1	0.599945	-1.12141	1.721355
ILMN_1685339	TPM1	0.564296	-1.15695	1.721246
ILMN_1788955	PDLIM1	-0.40793	-1.31317	1.721099
ILMN_1724994	COL4A2	0.432284	-1.28816	1.720444
ILMN_1787026	SEC61G	0.718191	-1.0018	1.719991
ILMN_1815578	ZNF223	-0.34682	1.3731	1.719917
ILMN_1792138	UQCRH	-1.14239	0.577466	1.719856
ILMN_1674376	ANGPTL4	0.083038	1.63635	1.7193875
ILMN_2359789	RAC1	0.15537	-1.56375	1.71912
ILMN_2415979	KIAA1751	-0.43546	1.28352	1.718981
ILMN_2399896	SEC31A	0.457588	-1.26127	1.718858
ILMN_1811181	FLJ20444	0.557612	-1.16121	1.718822
ILMN_1789005	ATP6V0C	-1.40291	-0.31576	1.718672
ILMN_1798308	AHSA2	1.22985	-0.48876	1.718608
ILMN_3262691	LOC100130108	-0.19841	1.52018	1.718593
ILMN_1740010	PCNX	1.54917	0.168635	1.717805
ILMN_2107004	GPR1	0.178366	1.53942	1.717786
ILMN_1684032	ZNF613	-0.09513	1.62129	1.7164168
ILMN_3194217	LOC100129424	0.183936	1.53245	1.716386
ILMN_1782439	CNN3	1.2452	-0.47118	1.716378
ILMN_1667791	PPFIA4	1.33679	-0.37916	1.715945
ILMN_1799604	OCIAD1	0.362817	-1.35301	1.715827
ILMN_1679319	PSIP1	0.788613	-0.92706	1.715671
ILMN_1741175	RAB11FIP2	-1.56007	0.155534	1.715604
ILMN_1778796	ADSS	0.694	-1.0216	1.7156
ILMN_1739946	VKORC1	1.06107	-0.65395	1.71502
ILMN_2131880	DPY30	0.756545	-0.95719	1.713735
ILMN_1750507	RPL9	-0.78853	0.925063	1.71359
ILMN_2092933	ADAMTS4	-0.15497	1.55859	1.713556
ILMN_3244935	LOC100130308	0.207251	-1.50612	1.713371
ILMN_1664912	IL11RA	-1.07778	-0.63522	1.713001
ILMN_1711792	GPBP1	1.08924	-0.62362	1.712863
ILMN_1791114	CDR1	-0.87343	0.838893	1.712325
ILMN_1803367	EVI1	1.61116	-0.10095	1.712112
ILMN_1673642	FLJ35848	-1.63272	-0.07926	1.7119787
ILMN_1792955	IRGQ	0.247117	-1.46453	1.711647

ILMN_1762747	RPL15	1.09401	-0.61719	1.711195
ILMN_2075334	HIST1H4C	0.808824	-0.90228	1.711106
ILMN_2116714	SLC39A1	1.39655	0.313784	1.710334
ILMN_1706498	DSE	0.718509	-0.99141	1.709914
ILMN_1757604	TPM2	-0.66073	-1.04901	1.709743
ILMN_1694742	RPS29	0.838266	0.870351	1.708617
ILMN_1681203	TWF1	0.803652	-0.90473	1.708384
ILMN_2173611	MT1E	-0.87049	-0.83766	1.708145
ILMN_1739222	ETV5	0.621032	-1.08691	1.707942
ILMN_1703727	LOC339809	0.658033	-1.04982	1.707853
ILMN_1744006	GFOD2	0.963276	-0.74454	1.707816
ILMN_1668996	C1QBP	0.614666	-1.09274	1.707406
ILMN_3296087	LOC100131364	0.912508	-0.79476	1.707264
ILMN_2325610	AKT3	0.762115	-0.94446	1.706571
ILMN_2209578	CTGLF3	0.786066	-0.92048	1.70655
ILMN_3262849	LOC100128510	-0.34005	1.36648	1.706533
ILMN_1726786	TNRC6B	-0.24902	-1.45732	1.706341
ILMN_1766657	STOM	0.537719	1.16829	1.706009
ILMN_1791119	NDUFA10	1.0325	0.673394	1.705894
ILMN_1670926	CHST15	0.507879	-1.19772	1.705599
ILMN_1814971	TCF25	0.28014	-1.42528	1.70542
ILMN_1689655	HLA-DRA	-1.00377	0.701455	1.705225
ILMN_1759973	NDUFA5	1.20772	0.497486	1.705206
ILMN_1674780	SF3B1	0.145662	1.5593	1.704962
ILMN_2061318	TAF13	0.52061	-1.18399	1.7046
ILMN_3296884	LOC100133232	-1.70227	-0.00202	1.70428821
ILMN_3209317	LOC644949	0.030599	1.67356	1.7041588
ILMN_1761016	SPZ1	-1.42615	-0.27801	1.704158
ILMN_1683678	SPATS2L	1.05311	0.650464	1.703574
ILMN_2089073	ATP9A	1.26923	-0.43425	1.703483
ILMN_1680578	LOC650739	-1.07046	-0.63302	1.70348
ILMN_1651832	EHD1	-0.38557	-1.31779	1.703359
ILMN_1674038	CTSD	0.743972	-0.95931	1.703286
ILMN_1699570	TPD52L2	1.12807	0.574815	1.702885
ILMN_1800078	LMO2	0.463636	-1.23921	1.702846
ILMN_1737076	HHLA2	1.10571	-0.59704	1.702752
ILMN_2160819	RPS27	0.53923	1.16328	1.70251
ILMN_1793033	RBM28	-0.4797	1.22255	1.702254
ILMN_2395451	ASS1	0.609573	-1.09257	1.702143
ILMN_3236760	LOC100134816	0.857444	-0.84452	1.701966
ILMN_1692678	FMNL3	0.805562	-0.89628	1.701844
ILMN_2307861	COL6A3	0.203432	-1.49823	1.701662
ILMN_1680018	RIPK4	0.543925	-1.15769	1.701615
ILMN_1771066	PCDHB16	-0.86929	0.832159	1.701453
ILMN_3245282	LOC100192426	-0.71564	-0.98555	1.70119
ILMN_2082865	PLLP	1.2877	-0.4131	1.700798
ILMN_3209883	LOC728047	0.267011	-1.43364	1.700651
ILMN_3291986	LOC642892	0.819885	0.88012	1.700005
ILMN_1757995	PARP2	-1.00902	-0.69097	1.69999
ILMN_1663893	C21orf128	-0.61792	1.08204	1.699962
ILMN_1800952	PSMD11	-0.38239	1.31749	1.699876
ILMN_1791006	AHI1	-0.18616	-1.51366	1.699818

ILMN_1719256	CKS1B	-0.6745	1.02529	1.699789
ILMN_1756541	MXD4	-0.43689	-1.26277	1.699664
ILMN_2128770	CDR2L	0.721294	-0.97836	1.699651
ILMN_1810327	HNRPUL2	0.504377	-1.1952	1.699577
ILMN_1660498	RPS27	0.275207	1.42427	1.699477
ILMN_1775566	ATP1A1	1.17661	0.522629	1.699239
ILMN_1769546	RIN2	0.119482	-1.57959	1.699072
ILMN_1795963	OKL38	0.96805	-0.73086	1.698909
ILMN_2135798	NR2C2AP	-0.79816	-0.90057	1.698728
ILMN_1705594	NAT10	-0.62453	1.07392	1.698447
ILMN_1763460	NHP2L1	0.953011	-0.74522	1.698226
ILMN_3236599	FLJ44054	-0.08478	1.61343	1.6982123
ILMN_1663313	AMY1C	0.721135	0.977007	1.698142
ILMN_3243471	CNPY2	-0.34825	-1.34974	1.697989
ILMN_1703142	Mar-02	0.74994	-0.94798	1.697917
ILMN_1654016	MRLC2	0.94203	0.755812	1.697842
ILMN_2267535	NET1	1.03227	-0.66548	1.697747
ILMN_1658044	LOC648814	0.627159	-1.07028	1.697439
ILMN_2147920	BP75	1.32334	-0.37373	1.697069
ILMN_1689725	RPLP1	-1.00282	-0.69386	1.696683
ILMN_2218401	ZNF563	0.22794	-1.46851	1.69645
ILMN_1717165	IGBP1	1.19467	-0.50135	1.696015
ILMN_3188151	LOC100130824	0.484564	-1.21129	1.695854
ILMN_1723735	KIAA0889	-0.1778	1.51776	1.695563
ILMN_3187425	LOC100127975	-0.31745	1.378	1.695454
ILMN_1657529	CYP2B6	-0.45536	-1.23974	1.695095
ILMN_3249840	GEN1	-0.98404	0.710934	1.694973
ILMN_1760062	IFI44	1.2534	-0.44091	1.69431
ILMN_1660086	MYH11	-1.37307	-0.32097	1.694039
ILMN_1694274	NDUFC2	0.212583	1.48123	1.693813
ILMN_2103547	GOLGA8B	0.529204	-1.16424	1.693444
ILMN_1678775	CLEC2D	0.51687	1.17608	1.69295
ILMN_2361185	PHF20L1	0.704981	0.987771	1.692752
ILMN_3198464	LOC728210	-1.35421	-0.33831	1.692523
ILMN_1725612	NUP50	-0.47708	-1.2151	1.692178
ILMN_3245194	LOC100132323	0.571855	1.12029	1.692145
ILMN_3243859	NDUFB9	0.136909	-1.55506	1.691969
ILMN_2128428	DAB2	1.32199	-0.36994	1.691926
ILMN_2151818	PSMA6	-0.32676	1.36507	1.691834
ILMN_1774228	LOC650280	-1.32835	-0.36317	1.691516
ILMN_2387553	PSMA3	0.344117	-1.34733	1.691447
ILMN_1728957	ANKRD5	-0.20931	1.48194	1.691254
ILMN_3236344	CTGLF7	0.202159	1.48862	1.690779
ILMN_2292387	TANK	-0.40212	1.28842	1.69054
ILMN_1661647	LOC646144	0.868743	0.821786	1.690529
ILMN_1678671	KLHL24	0.76307	-0.92697	1.69004
ILMN_1679262	DPYSL3	0.00243	-1.68759	1.6900199
ILMN_1712929	DNAJB12	-0.86229	0.827638	1.68993
ILMN_2135232	SFRS3	0.875507	-0.81436	1.689867
ILMN_2387696	CCR6	-0.0368	-1.653	1.6897997
ILMN_1713636	S100A6	-1.25546	-0.43431	1.689766
ILMN_2123559	FAM73A	-0.51472	1.1749	1.689616

ILMN_1659990	C7orf68	0.668457	-1.02111	1.689567
ILMN_2071809	MGP	0.096247	-1.59298	1.6892267
ILMN_3239629	LOC729679	1.15123	-0.53773	1.688955
ILMN_2342455	PPA2	-0.58092	1.10794	1.688861
ILMN_1694799	PIAS2	0.590157	1.09867	1.688827
ILMN_2141118	C15orf59	1.08677	-0.60153	1.688303
ILMN_1706825	PKN2	0.586736	-1.10153	1.688266
ILMN_2376458	CSF2RA	-0.65588	1.03232	1.688199
ILMN_2246548	GSTTP2	-0.22236	1.46571	1.688074
ILMN_3293025	LOC728170	-1.21711	0.470532	1.687642
ILMN_1744302	LOC645489	1.19388	-0.49374	1.687615
ILMN_1754220	SF3A2	0.903357	-0.78377	1.687124
ILMN_1711823	C17orf70	-0.97346	0.713639	1.687094
ILMN_1673112	ZNF32	-0.52434	1.16272	1.687064
ILMN_1722781	EGR3	0.271546	-1.41538	1.686926
ILMN_1777906	RPRC1	0.636389	-1.04992	1.686309
ILMN_1718063	LIPA	1.52419	-0.1617	1.685885
ILMN_1664153	SLC30A5	-0.43371	1.25174	1.685451
ILMN_2311779	TMUB2	0.498091	-1.18733	1.685421
ILMN_1758281	CALCRL	0.065929	-1.61928	1.6852093
ILMN_1764261	TMEM128	1.11303	-0.57208	1.685106
ILMN_3243871	LOC642076	1.40101	0.283996	1.685006
ILMN_2104784	FKBP3	1.63774	0.047209	1.6849491
ILMN_3289262	LOC100131261	-0.85688	0.827994	1.684875
ILMN_3200330	LOC399988	0.06394	-1.62072	1.68466
ILMN_1695868	PRICKLE4	-0.56938	1.1151	1.684483
ILMN_2230566	RAB40B	1.3691	-0.31524	1.684341
ILMN_3247657	LOC100132119	0.601616	1.08267	1.684286
ILMN_1754421	NDUFAF1	0.95874	0.725409	1.684149
ILMN_3200157	LOC100132210	1.14781	-0.53618	1.683985
ILMN_1780188	B3GALNT2	-0.22412	1.45942	1.683535
ILMN_2155323	ZNF652	0.445334	-1.23777	1.683104
ILMN_3238116	LOC200030	1.01834	-0.66438	1.682723
ILMN_1683562	SNRPG	-0.94362	-0.73874	1.68235
ILMN_1688848	TMEM44	-0.6102	-1.07182	1.682024
ILMN_2288915	DNAJB14	1.038	-0.64389	1.68189
ILMN_1713936	GOLGA6B	-0.3948	-1.28669	1.681489
ILMN_3243700	RPS8	0.568195	1.11276	1.680955
ILMN_2403006	TJP1	1.05311	-0.62739	1.680503
ILMN_1773390	LOC641996	1.20948	0.470828	1.680308
ILMN_1760954	DENR	1.05295	-0.62729	1.680236
ILMN_1754865	LOC400455	0.754396	-0.92583	1.680224
ILMN_1688865	PPP1R9B	0.584348	-1.09549	1.679838
ILMN_2045419	BNIP3L	-0.26884	1.41064	1.679475
ILMN_2294751	ASCC3	-0.89277	0.786317	1.679085
ILMN_3238751	PMS2L4	-0.52442	-1.15421	1.678634
ILMN_2137789	KLF4	0.820044	-0.85842	1.67846
ILMN_2139100	SHISA5	0.403717	-1.2747	1.678417
ILMN_1752249	FAM38A	-1.49856	0.179837	1.678397
ILMN_1768117	RBM25	-1.22204	-0.45626	1.6783
ILMN_1720266	LOC91431	0.216084	1.46208	1.678164
ILMN_2066858	TNFSF13B	-0.76895	0.909193	1.678145

ILMN_2330213	CUX1	0.678961	-0.99918	1.678141
ILMN_2116366	RPL12	0.838107	-0.8395	1.677611
ILMN_3228090	ZNF716	-0.37515	-1.3022	1.677345
ILMN_1695706	H3F3B	0.926195	-0.75107	1.677262
ILMN_1652306	MEGF10	0.23725	-1.43953	1.67678
ILMN_3241979	TMEM179B	0.816543	-0.86017	1.676716
ILMN_3239254	UCA1	1.23645	-0.43963	1.676082
ILMN_2139970	ALDH1A3	1.07563	-0.60031	1.675938
ILMN_1666594	IRF8	0.645381	-1.03009	1.675471
ILMN_1682343	LOC652246	-0.82505	0.8499	1.674951
ILMN_2316806	RWDD1	1.25085	-0.42372	1.67457
ILMN_1685005	TNFRSF1A	0.990251	0.684016	1.674267
ILMN_2242463	CTSC	-1.18966	-0.48447	1.674128
ILMN_3238491	LOC100129055	0.098475	1.57555	1.6740247
ILMN_1711414	MRPS27	-0.24393	1.42944	1.673369
ILMN_2398627	TENC1	0.424009	-1.24923	1.673239
ILMN_1730698	ODF2	-1.16085	-0.51238	1.673231
ILMN_2138765	PLIN2	-0.54217	1.13102	1.673189
ILMN_2203950	HLA-A	-1.25355	-0.41961	1.673163
ILMN_2091846	FTHL2	1.15274	0.519694	1.672434
ILMN_1677404	RAP2A	-0.37936	-1.29301	1.672372
ILMN_1789196	TPM2	-0.51201	-1.15964	1.671651
ILMN_1735453	FAM98A	1.00211	-0.6694	1.671505
ILMN_3251269	SRP14	0.859194	-0.81031	1.669501
ILMN_2277077	PRKAR1A	1.02001	0.648239	1.668249
ILMN_2316778	ETNK1	0.587213	1.0807	1.667913
ILMN_2079098	C9orf80	0.413664	1.25385	1.667514
ILMN_1693664	POMGNT1	1.22929	-0.4379	1.667188
ILMN_1798104	GRIN2A	0.669492	-0.99738	1.666867
ILMN_2218648	RAG1AP1	0.845826	-0.82098	1.666802
ILMN_3301478	ZNF716	-1.01921	-0.64744	1.666651
ILMN_1697971	TRIM38	0.224757	-1.44184	1.666597
ILMN_1659463	APAF1	0.242184	-1.42338	1.665564
ILMN_1653458	LOC648840	-0.19499	-1.46969	1.664681
ILMN_1688622	UBQLN1	1.35971	-0.30482	1.66453
ILMN_1773868	U2AF1L2	0.59334	-1.07101	1.66435
ILMN_2189614	ANKRD36B	0.172	1.49183	1.66383
ILMN_1787461	RUNX3	1.05502	-0.60878	1.663796
ILMN_2215382	DDX51	-0.36997	1.29381	1.663782
ILMN_1706687	KLHL5	0.24282	-1.42081	1.66363
ILMN_1689251	SPG3A	-1.57654	-0.08664	1.6631778
ILMN_1729269	LOC285074	-1.36185	0.301299	1.663149
ILMN_1673711	HSP90AB1	-0.7894	0.873528	1.662931
ILMN_1683194	DCN	1.34777	-0.31434	1.662106
ILMN_1728605	TTC3	-0.10539	-1.55651	1.661902
ILMN_1681260	LOC643272	0.724238	-0.93744	1.661682
ILMN_3199628	LOC100131271	-0.0746	-1.587	1.6615969
ILMN_1750658	HAX1	-0.1642	1.49717	1.661366
ILMN_1773121	LOC644424	-0.62079	1.04045	1.661237
ILMN_1805826	BIVM	0.49077	-1.17043	1.6612
ILMN_3247152	RIMBP3B	-0.52713	-1.13405	1.661179
ILMN_1726578	ZNF93	1.02717	0.63383	1.661

ILMN_3242459	DCTPP1	0.669412	-0.99152	1.660929
ILMN_3189463	LOC100129054	-0.47087	1.18938	1.660251
ILMN_1697088	ARMC5	0.621111	-1.03899	1.660101
ILMN_1684205	CIB1	-0.73211	0.927981	1.660091
ILMN_1786429	P2RY5	0.490054	-1.16993	1.659984
ILMN_3183620	LOC100128326	1.26168	0.397552	1.659232
ILMN_1662578	C1GALT1	1.27823	-0.38094	1.659166
ILMN_1661194	CLDN14	0.127439	1.53158	1.659019
ILMN_1765258	HLA-E	-0.18377	1.47512	1.658891
ILMN_3218538	LOC345645	0.539947	-1.1181	1.658047
ILMN_1793349	MOBKL1A	0.083117	1.57455	1.6576671
ILMN_1724959	SEC31A	-0.26764	-1.38982	1.657461
ILMN_1803882	VEGFA	0.622703	-1.0346	1.657303
ILMN_1655595	SERPINE2	0.946486	0.70994	1.656426
ILMN_1761474	LOC654189	0.517348	-1.13847	1.655818
ILMN_1737947	LSM5	0.858637	-0.79663	1.655269
ILMN_1669657	LOC440345	0.119641	1.53542	1.655061
ILMN_1768260	GAS6	0.946088	0.70823	1.654318
ILMN_1694731	CLCN7	0.776199	-0.87753	1.653728
ILMN_1711606	PRDX5	-0.93184	0.721497	1.653336
ILMN_1706505	COL5A1	-1.5658	0.087164	1.6529637
ILMN_2388975	CERK	-0.49737	1.15557	1.652939
ILMN_3201658	LOC642585	-0.18457	-1.46837	1.652937
ILMN_3245380	ZNF860	0.357247	1.29551	1.652757
ILMN_1708611	RDX	-0.09171	-1.561	1.6527051
ILMN_2393144	ARL6IP4	0.370854	-1.28182	1.652674
ILMN_2172269	TMEM183B	0.515597	-1.13653	1.652127
ILMN_1795495	ERCC5	0.358918	-1.29279	1.651708
ILMN_3276019	LOC728060	1.02105	-0.63033	1.651384
ILMN_1668432	IMPACT	-0.23215	1.41896	1.651112
ILMN_2211724	C21orf24	-0.06839	1.58251	1.6509002
ILMN_1720838	DECR1	0.371968	1.27887	1.650838
ILMN_1810953	LOC653496	1.33289	0.317424	1.650314
ILMN_3240793	OR2A4	0.493237	-1.15692	1.650157
ILMN_1656913	MDH1	0.429022	-1.22091	1.649932
ILMN_1791576	CHSY1	-0.37332	-1.27656	1.649875
ILMN_1762002	CSTF3	0.655805	-0.99403	1.649831
ILMN_3240316	TMSL3	-0.15282	1.49684	1.649657
ILMN_2123567	SENP2	1.59182	-0.05753	1.6493499
ILMN_1732080	SUMO1P3	0.359395	-1.28966	1.649055
ILMN_2367020	SEC61G	-1.33138	-0.31718	1.648556
ILMN_1782977	UBA52	0.244014	1.40445	1.648464
ILMN_1801504	RUNX1	-0.12998	1.518	1.64798
ILMN_1744517	GNS	1.36735	-0.28052	1.647867
ILMN_1698745	CLRN1	-1.59357	0.054221	1.6477913
ILMN_1710752	NAPRT1	-0.56174	-1.08598	1.647724
ILMN_1771026	GARS	-0.15465	-1.49271	1.647358
ILMN_1696883	THOC2	-0.05765	1.58955	1.6471978
ILMN_1771720	LOC645162	-1.22443	-0.42262	1.647049
ILMN_3267085	LOC100128564	-1.09743	0.549175	1.646605
ILMN_3238662	SNORD89	0.517905	-1.12864	1.646545
ILMN_2206141	BAGE5	0.191018	1.45466	1.645678

ILMN_1678404	FBXO11	0.018106	1.62755	1.6456558
ILMN_1654195	GCC2	0.274809	-1.37077	1.645579
ILMN_1714167	CYB5A	-0.55617	-1.08884	1.645014
ILMN_1673566	ADAMTS1	0.463795	-1.18121	1.645005
ILMN_1789358	ZNF628	0.949589	0.695401	1.64499
ILMN_1727389	CDC16	-0.10006	1.5449	1.64496
ILMN_3290261	LOC644877	0.474537	-1.16873	1.643267
ILMN_1731175	XKR6	1.19523	0.447738	1.642968
ILMN_2242491	CLUAP1	1.31897	0.323998	1.642968
ILMN_1797903	ZNF544	0.627795	-1.01516	1.642955
ILMN_1749882	STRN	0.296134	1.34645	1.642584
ILMN_2347068	MKNK2	0.26335	-1.37921	1.64256
ILMN_1815682	C3orf37	0.218869	-1.42362	1.642489
ILMN_1769508	PIGX	0.184095	1.45824	1.642335
ILMN_2339284	CHD2	0.706016	-0.93628	1.642294
ILMN_2136089	MTE	-1.40777	-0.23379	1.641563
ILMN_1682938	ARF3	0.934072	-0.7074	1.641469
ILMN_3239445	ZBTB42	0.963594	-0.67709	1.640687
ILMN_1773174	LOC653650	-1.51376	-0.12675	1.640506
ILMN_1679184	ANKRD20A1	0.679598	-0.96061	1.640208
ILMN_1785286	SOCS5	-0.48448	-1.15553	1.640008
ILMN_1795227	DNCL1	-1.08923	0.550506	1.639736
ILMN_1796245	DNASE2	1.17239	-0.46661	1.638999
ILMN_1712806	AP1S1	0.966618	-0.67216	1.638782
ILMN_3290497	LOC643863	-0.26104	1.37761	1.638647
ILMN_1718173	PCDH24	-0.79386	-0.84476	1.638623
ILMN_1815571	LOC646146	-1.34641	-0.29189	1.638295
ILMN_3199896	LOC442153	0.895798	-0.74242	1.638214
ILMN_1667260	MAPK3	0.094735	1.54312	1.6378548
ILMN_1684034	STAT5B	0.157279	-1.48038	1.637659
ILMN_1682350	LOC390933	-0.05908	-1.57854	1.6376202
ILMN_1785179	UBE2G2	-0.03656	-1.60093	1.6374909
ILMN_1656145	GOT1	-0.04444	-1.59291	1.6373487
ILMN_1740160	PLCG1	0.671799	-0.9652	1.636995
ILMN_1657797	FIBP	-1.09345	-0.5435	1.636945
ILMN_2293131	ARHGEF1	0.461567	1.17495	1.636517
ILMN_3217815	LOC646294	0.761956	0.874469	1.636425
ILMN_1657011	LOC286208	0.810257	0.8261	1.636357
ILMN_2404065	APP	-0.28459	-1.35161	1.6362
ILMN_3192813	LOC100128081	-0.29589	1.34002	1.63591
ILMN_2043809	PFKM	0.658431	0.977238	1.635669
ILMN_1727045	RASGRP3	0.383506	-1.25194	1.635446
ILMN_3187535	FAM30A	-0.23868	1.39621	1.634887
ILMN_1709814	NMRAL1	1.2974	-0.33531	1.632713
ILMN_3244338	LOC100134210	-0.40713	1.22549	1.632623
ILMN_1726666	GPX3	1.08502	-0.54746	1.63248
ILMN_1735689	SNORD64	-1.56628	-0.0659	1.6321831
ILMN_1811221	SLC5A8	-0.35684	1.2753	1.632143
ILMN_1767514	LOC441155	-0.81328	-0.8186	1.631872
ILMN_2150352	CBWD5	0.421144	1.21062	1.631764
ILMN_1765019	SACM1L	0.18322	1.4485	1.63172
ILMN_1719032	LSM3	0.839062	0.792578	1.63164

ILMN_2105033	PLDN	-0.18966	1.44155	1.63121
ILMN_1776523	LOC645307	-1.01372	0.617445	1.631165
ILMN_2183610	SERAC1	-0.3765	1.25441	1.630907
ILMN_1690894	TRA1P2	0.600184	-1.03046	1.630644
ILMN_1672446	RPL11	0.495545	1.13489	1.630435
ILMN_1660277	LOC731999	-0.56628	1.0639	1.630179
ILMN_2170643	FLJ45032	0.963674	0.666465	1.630139
ILMN_2206746	BGN	-0.37133	-1.25879	1.630115
ILMN_1730007	MPZL2	-0.76108	-0.86847	1.629545
ILMN_1677814	ABCC3	1.11065	-0.51819	1.628842
ILMN_1789001	SLC35B2	-0.37865	-1.24955	1.628196
ILMN_1773389	PLTP	0.454803	-1.17263	1.627433
ILMN_1700316	LOC440055	0.729808	-0.89742	1.627232
ILMN_1661864	LOC286239	-0.16101	-1.46614	1.627153
ILMN_1794612	UBA7	-0.21186	-1.41522	1.627081
ILMN_1805064	SCARNA9	0.626283	-0.99945	1.62573
ILMN_2407669	PEAR1	-0.05096	-1.57465	1.6256137
ILMN_1654893	SCAMP2	0.451302	-1.17386	1.625162
ILMN_2166865	ENY2	-1.15122	-0.47384	1.625055
ILMN_2263236	HFE	-0.27464	1.35002	1.624664
ILMN_1736002	COPS5	1.52785	0.096786	1.6246355
ILMN_3305597	LOC730235	0.760842	0.863557	1.624399
ILMN_1666179	HIST2H3C	1.20319	-0.42118	1.624365
ILMN_1745152	UQCC	-1.52235	-0.10173	1.624077
ILMN_1713394	LOC441876	-0.34873	1.27528	1.624006
ILMN_1687041	LOC647481	-1.32358	-0.30022	1.623796
ILMN_1727479	TPRG1L	0.638379	0.985037	1.623416
ILMN_1704056	RPPH1	0.921659	-0.70156	1.623221
ILMN_3235931	LOC100134768	1.15067	0.472213	1.622883
ILMN_2156172	HK2	0.639015	-0.98384	1.622851
ILMN_3246634	LOC100134108	0.439684	-1.18311	1.622794
ILMN_1683927	ITGAE	0.664081	-0.95866	1.622739
ILMN_1709164	RUNDC2C	0.300909	1.32167	1.622579
ILMN_1660436	HSPA1B	1.20486	-0.41731	1.622165
ILMN_1712515	FOXO3	0.852271	-0.76961	1.621878
ILMN_1716687	TPM1	-0.94752	-0.67434	1.621851
ILMN_1749210	BUD13	0.196986	-1.42455	1.621536
ILMN_1778177	ZNF207	0.80954	-0.81196	1.621504
ILMN_1761922	LOC647037	-1.38923	0.23226	1.62149
ILMN_1745697	MGRN1	-1.30512	-0.31618	1.621302
ILMN_1722276	PAFAH1B1	0.748508	-0.87276	1.621268
ILMN_1698533	IDH3A	0.525942	1.09524	1.621182
ILMN_1666785	SYNE1	-0.71285	-0.90802	1.620869
ILMN_3246401	AIF1L	-0.23995	-1.38063	1.62058
ILMN_2347145	DCN	-0.58554	1.03465	1.620186
ILMN_1659692	LOC23117	-0.95046	0.669625	1.620084
ILMN_1739792	RHOG	0.445891	-1.17416	1.620051
ILMN_2119945	NDUFB3	0.506844	-1.11317	1.620014
ILMN_1713650	FLJ40113	0.469127	-1.15076	1.619887
ILMN_1721733	LOC441155	0.218948	-1.40074	1.619688
ILMN_2361570	SNX14	-0.63336	-0.98631	1.61967
ILMN_2259292	C7orf28A	0.903755	0.715858	1.619613

ILMN_1778668	TAGLN	-0.41843	-1.20081	1.619243
ILMN_1691789	SMNDC1	0.703708	0.915317	1.619025
ILMN_1668374	ITGB5	0.292633	-1.32586	1.618493
ILMN_1838885	KIAA1632	1.12568	-0.49266	1.618344
ILMN_1807945	ANP32A	1.02367	-0.59465	1.618315
ILMN_3238369	JHDM1D	0.612279	-1.00583	1.618109
ILMN_2049343	LOC642947	0.394009	-1.22396	1.617969
ILMN_3248703	SNORD99	-0.04094	-1.57698	1.6179175
ILMN_2392717	LARP7	0.014684	1.60314	1.6178242
ILMN_2188533	CICK0721Q.1	0.658272	-0.95913	1.617403
ILMN_3239907	LOC100131875	0.835163	-0.78215	1.617309
ILMN_2388605	ACTR2	0.817259	-0.80002	1.617276
ILMN_2128623	LOC285359	0.307513	1.30967	1.617183
ILMN_1725700	MOV10	0.896673	-0.72043	1.617106
ILMN_3212013	LOC100133169	0.72941	0.887352	1.616762
ILMN_1750092	SEPSECS	0.217039	1.3996	1.616639
ILMN_3296979	LOC730187	0.061553	1.55507	1.6166228
ILMN_2378670	SNX15	-0.41549	1.20108	1.616568
ILMN_2082244	FOXK1	1.14526	-0.47128	1.616538
ILMN_1769520	UBE2L6	-1.54137	-0.07468	1.6160546
ILMN_1718607	TSPAN4	1.17383	-0.44165	1.61548
ILMN_3240150	SNORA75	-0.89054	0.724911	1.615451
ILMN_2147993	ZNF23	0.016753	1.5986	1.6153531
ILMN_3214052	LOC644877	1.56238	0.052689	1.6150687
ILMN_1727605	FRK	-1.53906	0.075708	1.6147676
ILMN_1802251	PTTG1IP	-0.49045	-1.12426	1.614706
ILMN_1776076	POFUT1	0.748349	0.866208	1.614557
ILMN_1761242	COMMD1	0.892854	0.721574	1.614428
ILMN_1745841	KIAA0492	0.052641	1.56157	1.6142106
ILMN_3308148	MIR612	-0.51511	-1.09878	1.613894
ILMN_1749868	FAM171A1	0.471036	1.1428	1.613836
ILMN_1663453	HIST1H2AI	0.16826	-1.44556	1.61382
ILMN_1798826	MRPS25	0.75742	0.8558	1.61322
ILMN_1678235	KIAA1267	1.49315	-0.12006	1.61321
ILMN_1755158	TLL2	-0.42798	1.18509	1.613071
ILMN_1735004	C4orf43	-0.51782	-1.09467	1.612489
ILMN_2211800	HMGB1L1	0.006409	-1.60604	1.61244856
ILMN_3188984	C20orf199	-1.14056	-0.47173	1.612288
ILMN_1741782	SS18	1.07762	-0.53447	1.612085
ILMN_1755114	EIF2AK4	-1.0107	0.600994	1.611694
ILMN_3233239	LOC731789	-0.17605	1.43543	1.611483
ILMN_1760338	LOC643357	0.387007	-1.22417	1.611177
ILMN_1780806	ANKRD36B	0.529125	-1.08201	1.611135
ILMN_1739283	UPF2	0.852351	-0.75854	1.610886
ILMN_1774334	HIGD2A	1.39289	-0.21776	1.610647
ILMN_2383077	SLC39A7	-0.79195	-0.81835	1.610298
ILMN_1653283	APP	-0.35796	-1.25228	1.610237
ILMN_1804663	THBS3	0.520769	1.0894	1.610169
ILMN_2147251	MGC10997	0.969244	0.640712	1.609956
ILMN_2402363	STK19	0.114867	-1.49502	1.609887
ILMN_1788416	FAM108C1	1.00434	-0.60552	1.609856
ILMN_1792508	TMEM59	0.231123	1.3781	1.609223

ILMN_2300970	ETFB	0.968926	-0.6402	1.609124
ILMN_1738598	LOC651483	0.237489	-1.37049	1.607979
ILMN_1734194	EXOSC3	0.990012	-0.61762	1.607635
ILMN_1754990	LOC388720	0.585065	1.0225	1.607565
ILMN_3274601	LOC343184	1.25913	-0.34758	1.60671
ILMN_1692121	USO1	1.36854	-0.2374	1.605943
ILMN_2188909	FANCL	0.560795	-1.04498	1.605775
ILMN_1792268	LOC731950	-0.89293	0.712319	1.605246
ILMN_3249059	SNHG12	0.05837	-1.54638	1.6047498
ILMN_3246448	LOC100132540	0.653179	0.951526	1.604705
ILMN_3306869	LOC728975	-0.43713	1.16723	1.604362
ILMN_1809477	CARHSP1	1.1903	-0.41368	1.603984
ILMN_2213680	ZCCHC10	-0.60161	-1.00218	1.60379
ILMN_2062381	LCOR	0.211946	-1.3916	1.603546
ILMN_1756779	CLTC	1.25913	-0.34418	1.603307
ILMN_1741259	LOC649209	0.518144	-1.0851	1.603244
ILMN_1697694	ATP6AP1	0.303694	-1.29947	1.603164
ILMN_1814719	RBP4	0.020016	-1.58311	1.6031256
ILMN_3246019	LOC645534	1.11701	-0.48527	1.602277
ILMN_1682912	LOC441124	0.903835	0.698342	1.602177
ILMN_1666269	CTSZ	-0.72686	-0.87513	1.601985
ILMN_1750100	TUBB4Q	0.678404	-0.9235	1.601906
ILMN_3213792	LOC439953	-0.55777	-1.04411	1.601875
ILMN_1678678	SLC37A4	1.37666	-0.22521	1.601867
ILMN_3267017	LOC100129028	-1.49991	-0.10192	1.601833
ILMN_1676503	LOC643446	0.66798	0.933738	1.601718
ILMN_3206312	LOC643873	0.696944	-0.90459	1.601534
ILMN_2360730	CAV2	1.01062	-0.59083	1.601449
ILMN_2141452	RPL18A	0.856648	0.744664	1.601312
ILMN_1661337	SRM	0.323189	1.27807	1.601259
ILMN_1751425	ERMP1	0.885055	-0.71593	1.600985
ILMN_1711073	LOC653489	0.002748	1.59813	1.6008782
ILMN_3236942	SNORA5A	1.11558	0.484627	1.600207
ILMN_1809173	LOC729021	1.06943	0.530316	1.599746
ILMN_3275771	LOC642567	-1.52243	-0.07726	1.5996886
ILMN_3273996	LOC100129148	0.086937	-1.51269	1.5996266
ILMN_1696187	PYGL	0.366318	-1.23317	1.599488
ILMN_1656082	H3F3A	0.904073	-0.6953	1.599374
ILMN_1728180	CROP	-1.37108	0.228094	1.599174
ILMN_2130411	KDELR1	-0.15552	1.44294	1.598463
ILMN_1753393	OSGEP	0.256666	-1.34177	1.598436
ILMN_1719627	SLC27A3	0.195872	-1.40251	1.598382
ILMN_1752988	C11orf17	1.12362	0.474526	1.598146
ILMN_1701753	LOC644063	0.273217	1.3248	1.598017
ILMN_3246766	LOC100132247	1.41207	-0.18577	1.597837
ILMN_1664176	FBLN5	-1.24337	-0.35436	1.597731
ILMN_1772177	ASCC3	1.50859	0.088809	1.5973988
ILMN_1672947	CAST	0.49077	1.10645	1.59722
ILMN_1771261	SYNC1	0.982294	-0.61475	1.597041
ILMN_1690321	BCLAF1	-0.133	1.46371	1.596714
ILMN_1805300	LOC650132	0.948714	-0.64786	1.596575
ILMN_1807710	HINT1	1.4322	-0.16416	1.596362

ILMN_2297069	FAM115A	0.410242	1.18595	1.596192
ILMN_1798980	CAPZB	1.39194	-0.20401	1.595945
ILMN_1722537	FLJ40504	-0.76768	0.828201	1.59588
ILMN_1693421	RPN2	0.569468	-1.02628	1.595748
ILMN_3309990	MIR1256	1.32891	-0.2668	1.595711
ILMN_1661366	PGAM1	-0.48249	1.1132	1.595689
ILMN_1733305	EIF2A	0.944974	-0.65038	1.595356
ILMN_1749034	CALN1	-0.6714	0.923726	1.595122
ILMN_1677402	LOC387763	0.318654	-1.27605	1.594704
ILMN_1711748	PLTP	0.128553	-1.46614	1.594693
ILMN_2391765	C6orf48	1.25221	0.342318	1.594528
ILMN_1761911	SCYL1BP1	-0.54686	1.04762	1.594484
ILMN_1756727	ANKRD30A	-1.46689	-0.12759	1.594477
ILMN_2100085	WDR47	-0.60487	-0.98955	1.594425
ILMN_1731851	OXA1L	1.26963	-0.32461	1.594238
ILMN_1763694	RSPRY1	0.696069	-0.89789	1.593954
ILMN_1696311	IMPAD1	0.63074	-0.96268	1.593421
ILMN_3188124	LOC100130511	-0.10706	-1.48581	1.592873
ILMN_1659490	LOC653158	0.517587	-1.07506	1.592647
ILMN_3238392	LOC729010	0.595807	0.996611	1.592418
ILMN_1767612	BBS2	0.36974	1.2223	1.59204
ILMN_1698803	ZAK	0.432205	1.15979	1.591995
ILMN_1711566	TIMP1	0.307673	-1.28376	1.591433
ILMN_3240490	LOC100131970	0.518701	1.07253	1.591231
ILMN_1764850	HPCAL1	-0.69869	-0.8924	1.591089
ILMN_2179652	PRELID1	0.518382	-1.0726	1.590982
ILMN_3304203	LOC729342	-0.56039	1.03039	1.590781
ILMN_1667825	MLKL	0.531353	-1.0591	1.590453
ILMN_1668771	LOC642946	0.715883	-0.87457	1.590448
ILMN_1791186	ARHGAP18	1.48766	0.102662	1.590322
ILMN_1720965	TULP4	0.558567	-1.03174	1.590307
ILMN_3236045	SPINT3	0.639174	0.950893	1.590067
ILMN_1723971	SLC29A1	-0.38302	-1.20691	1.589932
ILMN_1730709	BSG	0.634798	0.955071	1.589869
ILMN_1666932	FCGR2A	0.714132	0.875398	1.58953
ILMN_1794781	VAV2	0.435547	1.15378	1.589327
ILMN_1745778	SLC45A4	0.873676	-0.71524	1.588919
ILMN_1749405	KIAA1191	0.793546	0.795229	1.588775
ILMN_2115218	ANKRD10	0.525783	1.06263	1.588413
ILMN_3266944	LOC100129599	-1.31856	-0.26978	1.588337
ILMN_1809013	MYL6	1.11327	0.474846	1.588116
ILMN_1691578	GTF3C6	1.26382	-0.32395	1.587771
ILMN_3235775	LOC553137	0.728217	-0.85875	1.586964
ILMN_3242673	LOC100132453	-0.53246	1.0545	1.586961
ILMN_2371825	AGL	0.989296	-0.59749	1.586788
ILMN_1790534	MAP2K3	-0.67116	0.915128	1.586285
ILMN_2258004	METRNL	0.434273	1.15159	1.585863
ILMN_3245116	GOLIM4	-0.23748	-1.34727	1.584753
ILMN_1712347	LOC644422	0.483609	-1.1006	1.584209
ILMN_2056002	LOC723972	0.682701	0.901417	1.584118
ILMN_1800276	RCN1	-0.50262	-1.08128	1.583901
ILMN_1785402	LTBP1	1.34459	0.238917	1.583507

ILMN_1810729	UBL3	1.06322	0.520233	1.583453
ILMN_2082209	C20orf100	0.568513	1.01478	1.583293
ILMN_1722102	ANAPC11	0.495784	-1.08748	1.583264
ILMN_1730685	MRPL16	0.746121	0.836497	1.582618
ILMN_1740426	RASD1	1.48217	-0.10037	1.582536
ILMN_1652160	LRBA	0.209241	1.37329	1.582531
ILMN_1663002	STOML2	0.968528	-0.61384	1.582364
ILMN_1785266	OFD1	0.257701	-1.32433	1.582031
ILMN_1651296	LOC143666	-1.40084	0.18102	1.58186
ILMN_1670542	AK2	1.32295	-0.25879	1.581744
ILMN_1689616	SNORA66	-0.03624	1.54514	1.5813827
ILMN_3205162	LOC646688	-0.33361	1.24754	1.581148
ILMN_2358801	PLEKHG5	1.14112	-0.43973	1.580852
ILMN_2415235	CSNK1E	-0.16316	-1.41749	1.580652
ILMN_1745034	SLC11A2	0.960491	-0.62012	1.580611
ILMN_3274543	LOC286002	0.506128	1.07436	1.580488
ILMN_2160929	FEN1	0.659863	-0.92052	1.580383
ILMN_1698668	NR1D2	0.922455	-0.65779	1.580246
ILMN_2075794	NLRP8	-0.5518	1.02808	1.579877
ILMN_1704730	CD93	0.61769	-0.96209	1.57978
ILMN_1679838	WBP5	1.21497	-0.36463	1.579604
ILMN_1759915	ARPC1A	-0.06362	-1.5154	1.5790158
ILMN_1671925	SDHAP3	-0.4544	-1.12451	1.57891
ILMN_1755419	EIF1AX	0.683497	0.895346	1.578843
ILMN_1772626	FLJ38773	-1.34092	-0.23776	1.578684
ILMN_3208995	LOC653075	0.520849	-1.05775	1.578599
ILMN_2112460	MAD2L1	-0.95651	0.62209	1.578596
ILMN_1743034	KIF1B	0.07699	-1.50138	1.57837
ILMN_1714401	SNX27	0.990172	0.588195	1.578367
ILMN_3275193	ZNF728	0.121153	-1.45716	1.578313
ILMN_1675249	SGTB	-0.60058	0.977042	1.577618
ILMN_1805696	DFFA	0.569787	-1.00772	1.577507
ILMN_1721967	OR2A7	0.551564	-1.02592	1.577484
ILMN_1813836	DARS	0.549893	1.02752	1.577413
ILMN_1799104	SPAG9	-0.04364	-1.53375	1.577393
ILMN_1811786	CCDC102B	-0.43984	-1.13735	1.577188
ILMN_1724854	LOC728005	-1.25172	0.325383	1.577103
ILMN_1802973	ANAPC4	0.676494	-0.9005	1.576995
ILMN_1659273	LOC441408	0.439366	-1.13745	1.576816
ILMN_1711617	GMFG	0.514324	1.06245	1.576774
ILMN_1744046	DIAPH2	0.846065	-0.73063	1.576693
ILMN_1807651	LOC653596	-0.69089	0.884683	1.575574
ILMN_3251555	MTPAP	-0.246	1.32943	1.575428
ILMN_1802611	SPEN	-0.15178	-1.42301	1.574793
ILMN_1756355	NDUFS3	-0.71039	0.864214	1.5746
ILMN_1778478	CCDC28B	0.093462	1.48107	1.5745316
ILMN_3249711	FLJ45340	1.37212	0.202264	1.574384
ILMN_1685520	LOC641999	0.567956	1.0064	1.574356
ILMN_1750144	C3orf19	0.113275	1.46105	1.574325
ILMN_2360229	MAPKAP1	0.985477	0.587668	1.573145
ILMN_1788268	NAPG	1.46077	-0.1123	1.573072
ILMN_2249288	TIPRL	0.257064	-1.3156	1.572664

ILMN_1689389	SF3B5	1.21242	-0.36024	1.572663
ILMN_2066060	HLA-DRB6	-0.86707	-0.70558	1.572646
ILMN_1803819	IQGAP1	0.868265	0.703739	1.572004
ILMN_1721204	CSF2RA	0.494033	1.07676	1.570793
ILMN_1684385	COPB2	-0.29104	-1.27966	1.570696
ILMN_1705331	ZNHIT3	0.187199	-1.38336	1.570559
ILMN_1703791	ANXA7	0.358281	-1.21227	1.570551
ILMN_1769634	STRA13	0.222848	1.34763	1.570478
ILMN_1724783	MGC25181	-0.14828	-1.42155	1.569832
ILMN_1783156	LOC650832	-0.73283	0.836769	1.569595
ILMN_3246214	B3GNT1	0.54488	-1.02418	1.56906
ILMN_2194467	SGCB	0.287541	-1.28056	1.568101
ILMN_2129545	GNB4	-0.3539	1.21393	1.567829
ILMN_1756404	LOC648309	0.121631	-1.44619	1.567821
ILMN_1701134	PTEN	0.494829	-1.07279	1.567619
ILMN_3222402	LOC730284	1.34141	-0.22616	1.56757
ILMN_1728445	IGFBP1	0.276559	-1.29055	1.567109
ILMN_1750324	IGFBP5	0.366636	1.20025	1.566886
ILMN_1789176	PSMB1	-1.31546	-0.25136	1.566822
ILMN_1730907	ZFAND1	1.44071	0.125817	1.566527
ILMN_1703102	LOC731777	1.11661	-0.44957	1.566177
ILMN_1736834	SKIL	-0.56708	-0.99902	1.566096
ILMN_1789044	LOC646190	0.140171	-1.42574	1.565911
ILMN_1654268	HMGB2	-0.03266	-1.53277	1.5654319
ILMN_3183750	LOC100130353	-0.50095	1.06434	1.56529
ILMN_3238521	LOC100133952	0.52252	-1.04252	1.56504
ILMN_1756862	APOL3	-1.55147	-0.01355	1.5650154
ILMN_1669598	SUV420H1	0.418757	-1.14582	1.564577
ILMN_2182482	SHCBP1	-0.50835	1.05556	1.56391
ILMN_1752510	FAM13A	-0.60814	0.955698	1.563833
ILMN_1663160	ZNF337	1.21727	-0.34644	1.563714
ILMN_1727073	MEA1	-0.05805	-1.50533	1.5633757
ILMN_3225211	LOC729742	0.849725	-0.71363	1.563359
ILMN_2230016	HIGD1A	1.13786	0.425151	1.563011
ILMN_3291413	LOC646672	0.260884	-1.30207	1.562954
ILMN_2354391	EGLN2	1.16157	0.401221	1.562791
ILMN_3234513	LOC728416	1.46833	0.094052	1.5623817
ILMN_2320250	NOL6	1.05478	-0.50754	1.562315
ILMN_1656073	LOC442204	-0.89341	-0.66841	1.561811
ILMN_3228585	LOC728661	-1.44485	-0.11666	1.561513
ILMN_3252771	LOC100127999	-0.62795	0.933372	1.561321
ILMN_1715175	MET	-0.15958	-1.40173	1.561311
ILMN_1719205	FBL	1.43244	0.128728	1.561168
ILMN_1743137	BAT2D1	0.93821	0.62293	1.56114
ILMN_2134453	FCGR3B	-0.17844	1.38263	1.56107
ILMN_1692705	RECQL	-0.25475	-1.30628	1.561031
ILMN_3243876	LOC100134461	1.36974	-0.19118	1.560921
ILMN_2344130	PSMD4	1.47167	0.089211	1.5608812
ILMN_1719343	WDR26	0.796888	0.763712	1.5606
ILMN_3285616	LOC100133478	0.23542	1.32451	1.55993
ILMN_1788462	AMD1	-0.29947	-1.25991	1.559381
ILMN_2415179	CLSTN1	-0.2912	-1.26812	1.559315

ILMN_2038773	UBC	0.647927	0.911027	1.558954
ILMN_2129309	SEC23A	0.688032	-0.87084	1.558869
ILMN_1695317	RCBTB1	0.233033	-1.32569	1.558723
ILMN_3253456	FNDC3B	0.67546	-0.88321	1.55867
ILMN_1756942	SP3	0.715326	-0.84314	1.558463
ILMN_1805371	HNRPM	0.700446	-0.85726	1.557708
ILMN_1813114	LOC654113	-0.15258	1.4051	1.557679
ILMN_1782094	PAIP2	0.688748	-0.86773	1.556478
ILMN_1804861	LOC643854	1.55068	-0.00564	1.55631969
ILMN_1775919	C6orf79	-0.36353	1.19259	1.556117
ILMN_3234920	LOC728741	1.3048	-0.2512	1.555996
ILMN_1719695	NFKBIZ	0.306399	-1.24955	1.555949
ILMN_3248247	LOC100134634	1.10866	0.446738	1.555398
ILMN_1698243	C1orf85	0.912986	-0.64238	1.555362
ILMN_3227912	LOC728576	-0.66789	-0.88739	1.555288
ILMN_3256742	LOC100129902	0.804368	0.750457	1.554825
ILMN_1790537	C16orf75	1.16014	0.394232	1.554372
ILMN_3278995	LOC643167	1.09425	-0.45999	1.554244
ILMN_1769911	SLC38A1	0.439844	-1.11409	1.553934
ILMN_3242016	POTEE	0.258178	-1.29522	1.553398
ILMN_1712312	RAB11A	-1.30456	-0.24866	1.553224
ILMN_1703622	PPIB	0.997094	-0.55604	1.553134
ILMN_3249807	SKA2	0.533262	1.01941	1.552672
ILMN_2358540	RBMS1	0.417802	-1.13448	1.552282
ILMN_1757723	C6orf106	1.22929	-0.32297	1.552259
ILMN_3278753	LOC647515	1.22467	-0.32694	1.55161
ILMN_1712184	HIST1H3C	1.47597	-0.07534	1.5513055
ILMN_1814011	LOC731985	0.718986	-0.83203	1.551016
ILMN_1814726	SCARB2	0.457509	-1.0935	1.551009
ILMN_1659843	DNAJC3	0.928423	-0.62239	1.550815
ILMN_2182198	ICT1	0.203909	-1.34685	1.550759
ILMN_1780036	WDR1	0.853544	-0.69658	1.550123
ILMN_1651369	LOC652300	-1.45535	0.094709	1.5500585
ILMN_1785795	METAP1	1.25388	0.295186	1.549066
ILMN_2336280	QKI	0.908132	0.640789	1.548921
ILMN_1726107	UBE2V1	0.307593	-1.24098	1.548573
ILMN_2357272	BCLAF1	0.12736	-1.42121	1.54857
ILMN_1666122	HEG1	0.459816	-1.08872	1.548536
ILMN_2107184	SNRK	0.285949	-1.26218	1.548129
ILMN_1745623	EFCAB4A	0.183379	-1.36465	1.548029
ILMN_3241970	POLR2J2	-0.2382	-1.30953	1.547729
ILMN_1755883	RPS27A	0.170011	1.3777	1.547711
ILMN_1699226	UBR4	1.21465	-0.3328	1.547448
ILMN_2411282	QSOX1	1.24083	-0.3065	1.547325
ILMN_1716195	HIST1H2BG	0.414062	-1.13325	1.547312
ILMN_1784553	SDC2	0.924524	0.622557	1.547081
ILMN_1682139	RAI14	0.953966	-0.59272	1.546688
ILMN_2402936	LOC440926	0.480346	-1.0662	1.546546
ILMN_1654331	HOXB4	0.809859	0.736681	1.54654
ILMN_3249172	NCRNA00092	0.15163	1.39487	1.5465
ILMN_2193443	TRIM16L	1.08852	0.457821	1.546341
ILMN_1739274	PDHB	-0.78678	-0.75949	1.546265

ILMN_3288161	LOC100132488	0.386211	1.15958	1.545791
ILMN_1801460	LOC651647	0.810655	-0.73508	1.545733
ILMN_1717173	ECT2	1.31722	0.22836	1.54558
ILMN_1737586	LOC653994	-0.08112	-1.46444	1.5455619
ILMN_3299558	SFRS18	0.212264	-1.33305	1.545314
ILMN_2384857	DHRS2	0.705857	-0.83907	1.544923
ILMN_1654653	KLC1	1.19841	0.34607	1.54448
ILMN_1796126	MXRA7	0.518939	1.02551	1.544449
ILMN_2058070	NEDD8	1.0033	-0.54106	1.544363
ILMN_1690844	LOC387820	1.42178	-0.12171	1.543491
ILMN_3240177	LLPH	-0.6936	0.849462	1.543059
ILMN_1700604	RBM14	-0.58044	-0.96247	1.542912
ILMN_3252446	LOC100129543	-0.01786	1.52467	1.5425313
ILMN_2356284	PFDN5	0.145502	1.3965	1.542002
ILMN_1797576	PLEKHA3	0.964549	-0.57735	1.541898
ILMN_1718977	GADD45B	1.09728	-0.44447	1.541746
ILMN_1691119	RNF122	-0.64792	-0.89378	1.541701
ILMN_1753534	RPS18	0.176377	1.36487	1.541247
ILMN_3280020	LOC441506	0.356292	-1.18469	1.540982
ILMN_2113535	PCYOX1	0.015082	1.52537	1.540452
ILMN_2172174	NP	0.524748	-1.01564	1.540388
ILMN_1661051	WBP11	0.63257	0.907725	1.540295
ILMN_1748742	LOC647592	-0.18337	1.35682	1.540193
ILMN_3263864	LOC100129905	-0.14351	1.39634	1.539847
ILMN_1760020	LOC648370	-1.13714	0.402493	1.539633
ILMN_1710482	APLP2	-0.06982	-1.46948	1.5393025
ILMN_3244333	LOC100132176	-0.84622	0.692703	1.538921
ILMN_1777555	ESF1	1.45058	-0.08818	1.5387623
ILMN_3271412	LOC100127922	0.254677	1.28396	1.538637
ILMN_3262025	LOC100127993	-0.48082	-1.05754	1.538358
ILMN_2389844	SP3	0.532467	-1.00569	1.538157
ILMN_3283664	LOC100132037	0.700366	0.837734	1.5381
ILMN_1726636	HECTD2	0.731081	0.806904	1.537985
ILMN_1661346	LOC648210	-0.24687	1.29106	1.537933
ILMN_1685608	NPTX2	0.561829	-0.9761	1.537931
ILMN_1654151	COX6C	0.085027	-1.45279	1.5378168
ILMN_1757742	ZRANB1	-1.21202	-0.3256	1.537622
ILMN_3244515	LOC100134009	-0.06354	-1.47334	1.5368762
ILMN_3240717	C2orf14	-0.33735	1.19928	1.536627
ILMN_1751904	EDNRB	0.367273	-1.16925	1.536523
ILMN_1665510	ERRFI1	0.687316	0.849178	1.536494
ILMN_1653622	LOC648716	0.097918	1.43809	1.5360077
ILMN_1779147	ENC1	0.729251	0.806282	1.535533
ILMN_1673682	GATAD2A	-0.44215	1.09333	1.535475
ILMN_2246256	RGPD5	0.187915	-1.34751	1.535425
ILMN_2360415	PRNP	1.0524	0.482811	1.535211
ILMN_1808435	OGT	-1.39273	-0.14095	1.533684
ILMN_1787628	NOP56	0.750895	-0.78218	1.53307
ILMN_1779616	SUCLG1	-0.46562	-1.06694	1.532559
ILMN_1654516	TMEM120A	-0.75726	0.774926	1.532181
ILMN_1728355	PSMD4	1.45321	-0.07853	1.531735
ILMN_1705753	C3orf26	0.380402	1.15061	1.531012

ILMN_2311548	PTRH2	0.495784	-1.03481	1.530594
ILMN_1801999	LOC644474	0.709119	-0.82126	1.530379
ILMN_3200414	LOC441131	-0.89317	0.636191	1.529357
ILMN_3310201	MIR181C	0.67355	-0.85509	1.52864
ILMN_1707925	ABHD12B	-0.01993	1.5087	1.5286302
ILMN_1708906	C2orf29	-0.96415	0.564365	1.52851
ILMN_2191681	CHCHD2	-0.64259	-0.88552	1.528114
ILMN_1776038	UBXN2A	-0.62763	0.900234	1.527864
ILMN_3226613	LOC729686	-0.45591	1.07169	1.527602
ILMN_3242315	SNORD3D	-0.45217	-1.07536	1.527532
ILMN_3207738	LOC646527	1.20868	-0.31856	1.527235
ILMN_3203801	LOC442041	1.13046	0.396511	1.526971
ILMN_1757845	SPIRE1	0.506367	1.02016	1.526527
ILMN_1710697	BUD31	0.121949	-1.40394	1.525889
ILMN_1706817	SERP1	1.03847	-0.48711	1.525577
ILMN_2120273	AP1S2	-1.2491	0.276209	1.525309
ILMN_3217610	LOC645181	0.920306	-0.60483	1.525135
ILMN_3278548	LOC643308	-0.55705	0.967965	1.525014
ILMN_1686235	GNPNAT1	1.4918	-0.03316	1.5249559
ILMN_3209194	LOC653557	0.413664	1.11127	1.524934
ILMN_3248595	LOC642441	0.06577	-1.45916	1.5249301
ILMN_1675365	LARP4	-0.94728	0.576886	1.524162
ILMN_2053679	ACADM	1.12767	-0.39546	1.523134
ILMN_1738342	METT5D1	-0.28125	1.24166	1.522908
ILMN_1772612	ANGPTL2	0.573049	-0.94957	1.522617
ILMN_1671404	SVIL	1.24886	0.273528	1.522388
ILMN_3226807	NHP2	0.782326	-0.73986	1.522186
ILMN_2385410	GPX5	0.499285	-1.02277	1.522055
ILMN_2363489	BRE	0.54297	-0.97904	1.522007
ILMN_1778236	PTPN11	-0.02829	-1.49346	1.5217453
ILMN_2386008	MPZL1	-0.55426	-0.96712	1.521384
ILMN_1794740	CD151	0.300511	-1.22085	1.521361
ILMN_2363591	SDCBP	-1.09687	-0.42397	1.520838
ILMN_2321451	HNRNPD	-0.94346	0.57691	1.520366
ILMN_1804361	SUFU	1.08669	0.433583	1.520273
ILMN_1694136	LOC649144	0.028132	1.49204	1.520172
ILMN_2410421	NBPF1	0.982373	-0.53777	1.52014
ILMN_1728779	NGLY1	1.40642	-0.11274	1.51916
ILMN_1671933	CLCC1	0.368069	1.15086	1.518929
ILMN_3205603	LOC388572	1.32971	0.189092	1.518802
ILMN_2396020	DUSP6	-1.50532	0.013403	1.5187227
ILMN_2336609	SYTL2	0.649837	-0.86885	1.518691
ILMN_1690921	STAT2	1.47143	-0.04712	1.5185511
ILMN_1772218	HLA-DPA1	0.533422	0.985078	1.5185
ILMN_1733511	GOLGA3	0.563819	-0.95446	1.518275
ILMN_1755536	PFDN5	0.242184	1.27607	1.518254
ILMN_1766115	PLEKHF2	0.0863	-1.43184	1.51814
ILMN_2222984	RDH14	-0.32947	1.18776	1.51723
ILMN_1712231	SH3YL1	0.11606	-1.40112	1.51718
ILMN_1652955	LOC648622	0.552599	0.964444	1.517043
ILMN_1670215	FLJ46836	0.393771	1.12287	1.516641
ILMN_2412624	TP53I11	0.102215	-1.41419	1.516405

ILMN_1671217	LOC440733	0.313641	1.20255	1.516191
ILMN_3310840	MIR21	-0.51997	-0.9955	1.515468
ILMN_3250642	LOC728003	1.29939	-0.21555	1.51494
ILMN_2198823	H6PD	-0.156	1.35885	1.51485
ILMN_1736353	PSMC1	-0.52896	0.985445	1.514405
ILMN_3294335	LOC646294	0.731718	0.782672	1.51439
ILMN_3214893	LOC100132761	1.36496	-0.14941	1.51437
ILMN_1748473	GIMAP4	1.06083	-0.45305	1.513883
ILMN_3249608	ANKLE2	-0.0368	-1.47693	1.5137297
ILMN_1784523	ATP6V1G1	0.037442	1.47594	1.5133821
ILMN_1678314	POLR2J4	-0.15847	1.35487	1.513337
ILMN_1694305	SMS	-0.40689	-1.10643	1.513324
ILMN_1679039	CASKIN1	1.22523	-0.2879	1.513126
ILMN_1752340	ARF5	0.681428	0.831627	1.513055
ILMN_1714599	CAMLG	0.623101	0.889085	1.512186
ILMN_2066249	RPP30	-0.26136	1.25	1.511355
ILMN_1782444	YIPF4	0.464273	-1.0468	1.511073
ILMN_1728132	LDHB	0.415096	-1.0957	1.510796
ILMN_1777528	NCBP1	0.922455	0.5881	1.510555
ILMN_1694504	RNF220	1.42186	0.088685	1.5105445
ILMN_1763852	ACACB	0.472707	-1.03782	1.510527
ILMN_1794949	DSCR1L1	0.700605	-0.80966	1.510261
ILMN_1773906	NCOA4	0.307513	1.20231	1.509823
ILMN_2368292	TSEN34	1.19483	-0.31486	1.509686
ILMN_3242508	C19orf71	0.373161	-1.13559	1.508751
ILMN_1710652	MTMR8	0.458305	1.05042	1.508725
ILMN_1673380	GNG12	0.811132	-0.69747	1.508605
ILMN_1760610	LOC654116	1.01754	-0.49092	1.508458
ILMN_1761083	HNRNPA3	-1.10833	0.400102	1.508432
ILMN_1704236	MAX	1.14995	0.358041	1.507991
ILMN_1753575	GP1BA	-0.16985	1.33779	1.507636
ILMN_3300051	LOC731542	0.780019	-0.72761	1.507624
ILMN_1771957	MAN1B1	0.508754	-0.99886	1.507615
ILMN_3226392	LOC729608	0.954921	0.552347	1.507268
ILMN_3287106	LOC100133077	0.354143	-1.15298	1.507123
ILMN_1653205	C8orf59	1.05415	-0.45283	1.506984
ILMN_3219808	LOC391670	0.588805	-0.91754	1.506348
ILMN_1807611	PPIG	0.298124	-1.20753	1.505654
ILMN_1728684	PELP1	0.222927	-1.28205	1.504977
ILMN_1654583	CHD1	0.785589	-0.71926	1.50485
ILMN_1657983	TERF2IP	0.649519	-0.85529	1.504805
ILMN_1651617	CTNNB1	-0.15608	-1.34869	1.50477
ILMN_3269405	HNRNPM	0.403319	-1.10124	1.504559
ILMN_1715896	PMVK	0.544801	-0.95969	1.504488
ILMN_3286809	LOC100131139	1.02574	0.47839	1.50413
ILMN_1772019	RPS6KA3	-1.09138	-0.41249	1.503874
ILMN_1769299	MTMR11	0.752327	-0.75128	1.503607
ILMN_2189936	RPL36AL	-0.336	1.16635	1.502345
ILMN_3237554	LOC729061	0.905028	-0.59723	1.502259
ILMN_2093027	MYO1B	-0.70092	-0.80103	1.501945
ILMN_3310563	MIR606	0.473821	1.02765	1.501471
ILMN_1753312	PLXDC2	0.303853	-1.19757	1.501423

ILMN_3212395	LOC642131	0.391543	-1.10987	1.501413
ILMN_1698519	LOC642267	0.176775	1.32415	1.500925
ILMN_1677305	PVR	-1.35429	-0.14652	1.500812
ILMN_1795419	C2orf24	0.300352	1.20044	1.500792
ILMN_1658411	CHD4	-0.34236	-1.15837	1.500731
ILMN_2206953	PLCL1	-1.45448	-0.04621	1.5006898
ILMN_1773797	LOC652615	-0.47573	1.02451	1.500235
ILMN_1769091	PRCP	0.57663	0.922833	1.499463
ILMN_1789074	HSPA1A	1.37268	-0.12665	1.499332
ILMN_1720476	PHF2	0.417245	-1.08184	1.499085
ILMN_1664577	DLD	-0.06481	1.43415	1.4989594
ILMN_1662619	TFPI	0.90089	-0.598	1.498891
ILMN_2391150	FILIP1L	-0.1299	-1.36891	1.49881
ILMN_1655930	ELL2	-0.50692	0.991783	1.498701
ILMN_1687315	RXRA	-0.95834	-0.54021	1.498547
ILMN_1783681	MRPL34	0.397749	1.10062	1.498369
ILMN_1768115	TSPAN12	1.17033	-0.32763	1.497962
ILMN_1697493	WDFY3	-1.18082	-0.31711	1.497925
ILMN_2325612	AKT3	0.77604	-0.72178	1.497822
ILMN_1785528	FAM39DP	1.49689	-0.00064	1.49752945
ILMN_1680624	CREG1	1.31077	0.186719	1.497489
ILMN_1759030	MAP4K5	1.41461	-0.08232	1.496934
ILMN_1740572	TCN2	-0.31738	-1.17858	1.495955
ILMN_1806937	C19orf62	-1.19037	-0.30473	1.495096
ILMN_1683660	EIF3H	0.535013	0.959491	1.494504
ILMN_1666652	BRCA1	-1.32573	-0.16871	1.494437
ILMN_2318643	TGIF1	0.396237	-1.0982	1.494437
ILMN_1675325	ENPEP	1.05001	-0.44427	1.494281
ILMN_3297317	LOC728658	-0.16046	1.33378	1.494236
ILMN_3293114	LOC727821	0.640209	-0.85386	1.494068
ILMN_1771203	SMAD2	0.942348	0.551719	1.494067
ILMN_1716876	MED27	-1.28809	0.205424	1.493514
ILMN_3234837	PKDCC	0.756624	0.736622	1.493246
ILMN_1707901	DHRS7B	0.584189	-0.90866	1.49285
ILMN_3237177	FAM175A	-0.3196	1.17315	1.492753
ILMN_2222750	RPL23AP7	1.03624	-0.45646	1.492695
ILMN_2231569	ATAD3C	0.109933	1.38266	1.492593
ILMN_1810082	LOC642548	0.468569	-1.02377	1.492339
ILMN_2339796	CDC16	-0.1003	1.39185	1.492149
ILMN_1789599	NBL1	0.681269	-0.8108	1.492067
ILMN_1811515	DLG3	0.991843	0.499865	1.491708
ILMN_1692597	LOC652489	0.319211	-1.17227	1.491481
ILMN_1656111	MYLIP	-0.62389	0.867226	1.491116
ILMN_3274790	LOC648921	0.708323	0.782376	1.490699
ILMN_1662198	RANGAP1	-0.77651	0.714077	1.490589
ILMN_2116594	GPR89A	-1.29533	-0.19419	1.489518
ILMN_1653496	GLUL	0.816941	-0.67156	1.488501
ILMN_2050617	CCT6P1	0.084072	-1.40431	1.488382
ILMN_1774062	SLC25A5	0.714769	-0.77336	1.488127
ILMN_2114876	RPL11	0.205182	1.28263	1.487812
ILMN_1690965	DHX9	0.585701	-0.90203	1.487734
ILMN_1706013	FTHL11	-0.94481	0.542488	1.487297

ILMN_2058975	KIAA0776	-0.93717	-0.55012	1.487287
ILMN_1740265	ACOT7	0.742062	0.745143	1.487205
ILMN_3248389	LOC727781	0.314595	1.17253	1.487125
ILMN_1738383	EEF2	0.201681	1.28533	1.487011
ILMN_3240943	LOC100133772	-0.11796	1.36862	1.486584
ILMN_1789751	MFSD1	-1.25721	-0.2293	1.486506
ILMN_2249473	SPTLC1	-0.0407	1.44508	1.4857787
ILMN_3282937	LOC646049	-0.18337	-1.30223	1.485603
ILMN_3242038	GPX8	0.359713	1.12555	1.485263
ILMN_1676449	SLIT2	1.15409	-0.3311	1.48519
ILMN_1748591	ODC1	1.02876	-0.45637	1.485126
ILMN_2076250	GPBP1L1	1.04786	0.437169	1.485029
ILMN_3181695	LOC100130178	1.33568	0.149303	1.484983
ILMN_2388746	ARHGAP6	1.09775	-0.38694	1.484687
ILMN_1709377	ZNF28	1.44644	-0.03779	1.4842292
ILMN_3225309	LOC730176	1.1108	0.373208	1.484008
ILMN_1794132	NDUFS8	0.314595	-1.1692	1.483795
ILMN_2341229	CD34	-0.18759	-1.29592	1.483511
ILMN_3240301	LOC652776	0.10118	1.38207	1.48325
ILMN_1710078	TMEM181	0.691454	-0.79167	1.483121
ILMN_3222462	LOC730281	1.18361	0.299239	1.482849
ILMN_1753584	KRT8	-1.19825	0.284487	1.482737
ILMN_1728467	GHITM	-0.9059	0.576821	1.482719
ILMN_3220952	LOC730658	-0.08621	-1.39609	1.4823046
ILMN_3294134	LOC389765	-0.17375	1.30853	1.482275
ILMN_2070043	PPM1K	-0.14462	1.33685	1.481471
ILMN_1680154	MAP1B	0.41446	-1.06686	1.48132
ILMN_3202483	LOC100133876	0.664876	0.815892	1.480768
ILMN_2347807	EXOC1	0.538435	-0.94192	1.480352
ILMN_1660691	RAB31	0.01548	-1.46486	1.4803399
ILMN_3282773	LOC389322	0.947918	-0.53221	1.480128
ILMN_1892403	SNORD13	0.723681	-0.7561	1.479784
ILMN_1810826	MAK10	0.098952	-1.38079	1.4797422
ILMN_1662419	COX7A1	-0.24441	-1.23523	1.479636
ILMN_3246805	LOC100134364	-1.06783	-0.41178	1.479608
ILMN_1766859	MSL2	-0.21067	-1.26857	1.479237
ILMN_1748984	INADL	-0.53214	0.946343	1.478486
ILMN_1680388	FLJ45337	-0.16396	1.31445	1.478408
ILMN_2358457	ATF4	-0.09043	-1.38773	1.478162
ILMN_1682675	TWF1	0.772698	-0.70529	1.477988
ILMN_3243644	LOC100132564	0.937653	-0.53961	1.477266
ILMN_1689552	FAM63A	-0.12887	1.3483	1.477166
ILMN_2330307	SLC43A3	-0.27743	-1.19924	1.476669
ILMN_1691106	LOC729692	0.711825	0.764387	1.476212
ILMN_1677481	NKIRAS2	0.018902	1.45698	1.4758815
ILMN_3229210	LOC728440	1.08216	0.39296	1.47512
ILMN_3248223	SCARNA12	0.65119	0.823905	1.475095
ILMN_1766851	TMEM126B	0.759409	-0.71566	1.475072
ILMN_3235984	LOC100134688	1.00855	0.466189	1.474739
ILMN_2085933	OR1J1	-0.03752	1.4371	1.4746158
ILMN_1711078	CDC2L2	-1.11056	-0.36405	1.474614
ILMN_1773018	CUEDC2	-0.62556	0.84858	1.474141

ILMN_1756326	CKS2	0.206455	1.26745	1.473905
ILMN_1811560	SF4	0.35104	1.12243	1.47347
ILMN_3268938	LOC100128096	0.650315	-0.82256	1.472871
ILMN_3235357	LOC100133329	0.390508	1.08223	1.472738
ILMN_2326713	CD151	-1.38962	0.082962	1.4725824
ILMN_1687478	C10orf21	1.26239	-0.20977	1.472164
ILMN_1753265	C12orf76	0.921022	-0.55112	1.472144
ILMN_1662340	ZNF358	0.446846	-1.02521	1.472056
ILMN_1651347	SERTAD2	1.28722	0.184476	1.471696
ILMN_2126802	RPS27L	-0.4914	0.980161	1.471562
ILMN_2412873	ZNF37A	-0.02272	-1.44878	1.4714952
ILMN_1708619	SEH1L	-0.85457	0.616859	1.471432
ILMN_2122022	ZNF639	-0.57074	0.900305	1.471041
ILMN_1720829	ZFP36	0.369262	1.1014	1.470662
ILMN_2042651	EVI2B	0.115344	1.35523	1.470574
ILMN_2391419	SYNE2	-0.75129	-0.71927	1.47056
ILMN_1668378	SFT2D2	-0.65524	-0.81527	1.470508
ILMN_1759952	PSMA5	0.164998	-1.30484	1.469838
ILMN_2274180	MKKS	0.596603	0.873138	1.469741
ILMN_1693287	POMP	-0.18592	-1.28337	1.46929
ILMN_1784665	LOC387820	0.935266	0.533127	1.468393
ILMN_1791912	SIDT2	-0.03911	-1.42896	1.4680673
ILMN_2220187	GFPT1	0.329714	-1.13832	1.468034
ILMN_2155025	ZNF681	0.230487	-1.23736	1.467847
ILMN_1679800	BRIX1	0.672197	0.795542	1.467739
ILMN_1844692	FOXO3	0.248868	-1.21875	1.467618
ILMN_2289887	SEPN1	0.543846	-0.92366	1.467502
ILMN_2184602	HIST1H2AG	-1.13905	-0.32823	1.46728
ILMN_1758941	REEP5	0.554747	-0.91243	1.467177
ILMN_1797728	HMGCS1	0.75734	-0.70944	1.466784
ILMN_1769282	FRMD6	-0.34164	-1.1249	1.466544
ILMN_2379375	PPP1R12B	0.808983	0.657145	1.466128
ILMN_1725130	FAM50A	0.277673	-1.18833	1.466003
ILMN_1773200	CP110	-0.99359	0.472396	1.465983
ILMN_2094106	HSD17B12	0.666945	-0.79894	1.465885
ILMN_1691485	GTF2H2	-0.59556	0.870132	1.465694
ILMN_1754178	GDI2	0.17765	1.2879	1.46555
ILMN_1796734	SPARC	-0.07929	-1.38563	1.4649217
ILMN_1685079	TELO2	-0.41668	1.04793	1.464612
ILMN_2187830	CCNH	0.750497	-0.71382	1.46432
ILMN_1660655	SUGT1P	-1.24297	0.221271	1.464241
ILMN_2406892	C19orf2	1.36862	-0.09554	1.4641577
ILMN_3252597	LOC100130552	0.586815	0.876824	1.463639
ILMN_1657421	LOC440917	0.374991	1.08859	1.463581
ILMN_1702168	HSD17B12	-0.09107	-1.37234	1.4634086
ILMN_1743476	LOC653829	0.29518	1.1678	1.46298
ILMN_1812638	ATP5L	0.241547	-1.22071	1.462257
ILMN_1713993	UBAC2	-0.74795	-0.71384	1.461786
ILMN_1782543	EEF1D	1.04683	0.414896	1.461726
ILMN_3279219	LOC100132457	-0.47063	-0.99061	1.461245
ILMN_1683146	FTH1	-1.44397	0.017172	1.4611421
ILMN_3285785	LOC647307	-0.04205	1.41847	1.4605215

ILMN_2094776	CCNL1	-0.04062	-1.41987	1.4604892
ILMN_1791605	LOC729786	-0.45575	1.00381	1.459562
ILMN_3249629	LOC100133142	1.35104	0.108496	1.459536
ILMN_3241034	SNORD3C	-0.12942	-1.32956	1.458983
ILMN_1811921	CSRP1	-0.35334	-1.10552	1.458862
ILMN_1697153	ZDHHC17	0.085266	-1.37342	1.4586856
ILMN_1704385	LOC347376	0.545437	-0.91318	1.458619
ILMN_2383097	RPL17	-0.16245	1.29593	1.458376
ILMN_3301065	LOC728590	0.630103	0.828153	1.458256
ILMN_1665877	RNF149	-0.81757	0.640682	1.458254
ILMN_1774596	BSCL2	0.339104	-1.11891	1.458014
ILMN_1789955	PNRC1	-0.3173	1.14029	1.457585
ILMN_3284570	LOC648294	0.933197	-0.52426	1.457454
ILMN_3220934	NCRNA00152	-0.77771	0.679134	1.456839
ILMN_3238012	LOC100133033	1.05606	0.400487	1.456547
ILMN_1665515	MGC4677	0.746678	0.709544	1.456222
ILMN_2119555	TMTC3	0.280777	-1.17524	1.456017
ILMN_1656977	HIBCH	0.749224	-0.70674	1.455964
ILMN_3247256	LOC646996	-0.33273	1.1232	1.455932
ILMN_1794492	HOXC6	-0.91863	-0.53683	1.455455
ILMN_1693293	LOC648176	0.194042	-1.26016	1.454202
ILMN_1679044	LOC644584	0.959615	0.494433	1.454048
ILMN_2093748	ZNF669	-0.25213	1.20188	1.454005
ILMN_1671046	HSPB2	-0.78853	0.665275	1.453802
ILMN_1782305	NR4A2	0.19993	-1.25378	1.45371
ILMN_2128923	HCCS	0.059166	-1.3937	1.4528656
ILMN_1687947	HIST1H2BE	0.87479	-0.57774	1.452529
ILMN_2369826	C16orf13	0.897867	-0.55445	1.452315
ILMN_1796461	PRSS8	-1.42543	-0.02668	1.4521122
ILMN_1810514	SLC25A44	1.09927	0.352757	1.452027
ILMN_1734553	LOC653234	1.15839	-0.29301	1.451399
ILMN_1675687	LOC644701	0.244969	1.20615	1.451119
ILMN_3236458	AK2P2	-0.11606	-1.33476	1.450815
ILMN_1743104	RBM4B	1.31889	0.131917	1.450807
ILMN_1790819	LOC728556	0.439207	1.01159	1.450797
ILMN_2141453	RPL18A	0.754715	0.695981	1.450696
ILMN_2309156	PMEPA1	-0.69559	0.754954	1.45054
ILMN_1727252	WNT8A	0.916089	0.533334	1.449423
ILMN_1784328	SNORD25	0.341412	1.10785	1.449262
ILMN_3302905	LOC729798	1.06123	-0.3871	1.448332
ILMN_1699878	RAB27A	-0.31586	1.13229	1.448153
ILMN_3251341	TUBA1C	-0.09799	-1.34994	1.4479314
ILMN_3278735	LOC389465	1.07181	-0.37574	1.447551
ILMN_3200661	LOC389404	-0.56079	0.886736	1.447525
ILMN_1689474	PMS2L1	0.31754	-1.12992	1.44746
ILMN_1675519	LOC644611	-1.29358	0.153812	1.447392
ILMN_2061327	TAF13	0.624215	-0.82289	1.447103
ILMN_3208881	LOC647597	0.631615	0.814916	1.446531
ILMN_3243677	SNORA73B	0.585224	-0.8613	1.446528
ILMN_2365176	ALDH8A1	1.21544	0.230887	1.446327
ILMN_1690114	PTPLAD2	-0.21353	1.23269	1.446222
ILMN_1688996	LOC643060	-0.03537	-1.41017	1.4455373

ILMN_2411190	SMC2	1.42695	-0.01788	1.444827
ILMN_2141444	RPL18A	0.721373	0.723207	1.44458
ILMN_2380237	C1QTNF1	1.05152	-0.39252	1.444037
ILMN_2121068	ADAM17	-0.31952	1.12443	1.443953
ILMN_2287157	DST	1.13277	0.311163	1.443933
ILMN_1721895	LOC644338	0.436104	-1.00773	1.443834
ILMN_2148819	TUBA1A	0.721055	-0.72275	1.443807
ILMN_1759872	LOC643509	0.051606	1.39219	1.4437961
ILMN_2334587	HNRNPC	1.10412	0.339591	1.443711
ILMN_1780141	TMEM66	-0.45989	0.983818	1.443708
ILMN_2122952	CISD1	0.285631	-1.15804	1.443671
ILMN_3275489	LOC100131905	0.170011	1.27357	1.443581
ILMN_1697200	MON2	-1.10332	-0.33957	1.442893
ILMN_1713450	MYL6B	0.150436	1.29221	1.442646
ILMN_2343775	RPL38	0.290166	1.15236	1.442526
ILMN_1704557	RPS6KB1	-1.06878	0.373716	1.442496
ILMN_3224856	LOC729660	1.3913	0.050464	1.4417637
ILMN_1704753	EPAS1	-0.20605	-1.2357	1.441752
ILMN_1672496	DNAJA1	-0.53421	0.907347	1.441559
ILMN_1694233	ACYP1	1.11757	-0.32385	1.441415
ILMN_3207933	LOC647150	0.747633	0.69378	1.441413
ILMN_2165975	CES3	0.669571	0.771636	1.441207
ILMN_1706376	OSBP	0.058688	-1.38248	1.4411681
ILMN_1717337	Mar-07	0.28213	-1.15863	1.44076
ILMN_1652237	CBR3	1.15409	-0.28654	1.440631
ILMN_1666385	CALM3	0.883146	0.557436	1.440582
ILMN_1768227	DCN	0.21306	1.2274	1.44046
ILMN_1740486	POLR2J4	0.08256	-1.35763	1.4401901
ILMN_3231667	LOC100130133	1.30584	-0.13432	1.440155
ILMN_3255124	ATL1	-0.62588	0.814259	1.440139
ILMN_1759460	TAF7	0.186801	-1.2533	1.440101
ILMN_3249748	LDHA	-0.85752	0.582419	1.439936
ILMN_2101832	LAPTM4B	1.29788	-0.142	1.439876
ILMN_2087656	SLCO2B1	-1.03369	-0.40584	1.439533
ILMN_1727248	INF2	-1.38055	0.058713	1.4392626
ILMN_3239574	SNORD3A	-0.09131	-1.34772	1.4390273
ILMN_1760320	GNB1	-0.06736	1.37133	1.4386858
ILMN_2362245	HNRNPH2	1.22213	0.215916	1.438046
ILMN_3275345	LOC100132291	0.318017	1.12001	1.438027
ILMN_1689342	NUBP1	0.151152	-1.28664	1.437792
ILMN_1766797	CCS	0.282289	-1.15486	1.437149
ILMN_3245403	LOC100133931	-0.26732	1.16971	1.437033
ILMN_1748625	TCEAL4	1.16746	0.269552	1.437012
ILMN_1651980	LOC283767	1.25372	-0.18308	1.4368
ILMN_1684982	PDK4	0.21688	-1.21991	1.43679
ILMN_3295847	LOC645387	0.12545	1.31105	1.4365
ILMN_1772207	LOC653377	0.839062	0.597213	1.436275
ILMN_1738229	NDRG3	-0.28865	1.14757	1.436219
ILMN_1754894	C1orf162	-0.77802	0.658151	1.436175
ILMN_1770811	PELO	0.772619	0.663364	1.435983
ILMN_1685779	FOXR1	-0.58116	0.854729	1.435889
ILMN_1692249	LOC641750	0.553713	-0.88202	1.435728

ILMN_1676625	SS18L1	0.845985	0.588887	1.434872
ILMN_1739847	EIF3D	-0.31801	1.11669	1.434701
ILMN_1757552	PTRF	0.181708	-1.25299	1.434698
ILMN_2193233	MGC29506	0.253085	1.18153	1.434615
ILMN_3209477	LOC100131277	-0.58355	-0.85085	1.434394
ILMN_2082585	SNAI2	-0.78009	-0.65421	1.434303
ILMN_1685774	LOC647340	0.810416	0.623788	1.434204
ILMN_2208491	RPLP0P2	-0.18958	1.24425	1.43383
ILMN_2141941	TOR1AIP1	0.40881	-1.02468	1.43349
ILMN_2205470	FAM153B	0.233749	1.1997	1.433449
ILMN_1712400	SERPINB6	0.189825	1.24272	1.432545
ILMN_1803398	SRF	0.906301	-0.52553	1.43183
ILMN_2184007	LOC440354	-0.51472	0.916951	1.431667
ILMN_1671911	MTA1	1.33536	-0.09614	1.4315012
ILMN_1760503	ZBBX	0.137306	1.29371	1.431016
ILMN_1732291	LOC653125	0.500478	-0.92969	1.43017
ILMN_2149400	SPC25	-0.40817	1.02196	1.430128
ILMN_1777061	ZSWIM6	1.20574	0.224076	1.429816
ILMN_1657993	ADNP	0.023756	1.40602	1.4297755
ILMN_1706784	H2AFV	1.22786	-0.20163	1.429492
ILMN_1798467	FRYL	-1.07157	-0.35764	1.429209
ILMN_2207504	LEP	0.734185	-0.69441	1.428593
ILMN_1695290	FERMT2	-0.39058	1.03794	1.428522
ILMN_3243297	LOC641814	0.038238	1.39023	1.4284678
ILMN_2112599	C16orf80	-0.43562	-0.99233	1.427954
ILMN_1728965	C11orf57	0.217596	-1.21008	1.427676
ILMN_3220769	LOC729964	-0.48336	0.944289	1.427653
ILMN_1789510	STIP1	-0.35151	1.0755	1.427012
ILMN_1796113	BCDIN3D	0.715962	0.71068	1.426642
ILMN_1666403	GCSH	1.01651	-0.41004	1.426549
ILMN_2055271	A1BG	-0.24727	1.17924	1.426511
ILMN_1656274	PRPF38A	-1.01571	0.410689	1.426399
ILMN_3238910	LOC653545	0.141285	-1.28498	1.426265
ILMN_1792495	AHNAK	0.345947	-1.08011	1.426057
ILMN_3283244	LOC391334	0.56724	-0.85855	1.425792
ILMN_1704261	RANGRF	0.499683	-0.92606	1.425747
ILMN_1676629	INSIG2	0.536684	-0.88903	1.425717
ILMN_1790761	POSTN	0.512176	-0.91348	1.42566
ILMN_3241021	RNY4	1.12712	0.29808	1.4252
ILMN_3251303	NR2C2	1.33265	-0.09221	1.4248561
ILMN_1783818	AKAP11	-1.15791	0.266776	1.424686
ILMN_3249193	LOC100132329	0.424009	1.00007	1.424079
ILMN_3307742	CAPS2	0.959854	-0.46423	1.424079
ILMN_1669390	PPP1R13L	-0.05478	-1.36929	1.4240732
ILMN_3241481	LOC100134031	-0.63694	-0.78692	1.423861
ILMN_1774211	FLJ45032	1.31897	0.104668	1.423638
ILMN_3218248	LOC440595	0.196986	1.22652	1.423506
ILMN_2132809	ARHGEF10	0.663922	-0.75914	1.423061
ILMN_1789123	PLK4	0.22619	1.19677	1.42296
ILMN_2410540	CASP2	0.525066	-0.89728	1.422348
ILMN_1655952	FAM39E	1.25125	0.171002	1.422252
ILMN_3287493	LOC650157	-0.2834	-1.13664	1.420037

ILMN_2414027	CKLF	-0.39456	-1.02542	1.419981
ILMN_2372379	MGA	-0.97489	0.444957	1.419845
ILMN_1706326	MRPL33	0.821874	-0.59795	1.419821
ILMN_1695645	CETN2	1.36162	0.058192	1.4198119
ILMN_2061419	CYCSL1	-0.39981	1.01961	1.419422
ILMN_1769575	JAM3	-0.3122	-1.10705	1.419252
ILMN_2128750	PTTG1IP	-0.15361	-1.26521	1.418823
ILMN_1788410	PAMR1	-0.36759	1.05084	1.418425
ILMN_1668582	CRBN	0.984522	-0.43379	1.418307
ILMN_1743078	LOC643031	-0.30003	-1.11826	1.418288
ILMN_2338452	SERPINA1	1.31769	0.100306	1.417996
ILMN_1756162	EXOSC8	-1.28658	0.131349	1.417929
ILMN_1658071	ATP1B1	0.808983	-0.60883	1.417813
ILMN_3245020	WASH1	0.238842	1.17895	1.417792
ILMN_1810836	PDE5A	-1.20971	-0.20804	1.41775
ILMN_3276016	LOC339843	-1.25793	0.159434	1.417364
ILMN_2223836	CHORDC1	0.637583	-0.77968	1.417261
ILMN_1724139	TMEM123	-0.62429	0.792495	1.416783
ILMN_2352009	ACADVL	1.39599	-0.02075	1.4167429
ILMN_1678966	SNRPF	0.442072	-0.97436	1.416435
ILMN_2382431	ANKRD36	0.480108	-0.93622	1.416327
ILMN_2087702	MYH9	-0.36607	-1.04991	1.415983
ILMN_2361695	BAG5	1.12115	-0.29464	1.415792
ILMN_3282503	LOC644101	0.153141	-1.26219	1.415331
ILMN_1735014	KLF6	-0.54042	0.874901	1.415319
ILMN_2342084	BCAP29	0.490611	0.924649	1.41526
ILMN_1684293	ANP32B	0.673789	0.741403	1.415192
ILMN_3299478	LOC728428	0.464193	0.950609	1.414802
ILMN_1652073	LOC653658	0.445175	0.969492	1.414667
ILMN_1799837	LOC727848	1.28284	-0.13176	1.414598
ILMN_1668713	GABPB2	0.032588	1.38155	1.4141381
ILMN_2383107	VPS41	0.763229	0.650902	1.414131
ILMN_1737604	FLJ10986	0.733787	-0.6801	1.413886
ILMN_1787648	GPR125	0.552519	0.860948	1.413467
ILMN_2043816	ARPC5L	1.38684	-0.02656	1.4134038
ILMN_3200299	LOC646942	0.366318	1.0466	1.412918
ILMN_1761112	PORCN	0.191337	-1.22158	1.412917
ILMN_1654771	LOC647958	-0.69861	0.713923	1.412533
ILMN_3225505	LOC728553	0.49085	0.921507	1.412357
ILMN_1813763	SLC20A2	0.157041	1.25528	1.412321
ILMN_1728471	ARFGEF1	-1.06067	-0.35122	1.411889
ILMN_3230962	LOC728802	0.366398	-1.04518	1.411578
ILMN_2072598	LOC401357	1.09672	0.314607	1.411327
ILMN_2349610	DPH3	1.04794	-0.36321	1.411154
ILMN_1721138	GRPEL2	0.039909	-1.37113	1.4110389
ILMN_1732300	POLR2C	0.130224	1.2808	1.411024
ILMN_1674064	ZNF225	-1.16817	-0.24197	1.410135
ILMN_2150000	C15orf24	-0.76235	-0.64724	1.409588
ILMN_1690638	LOC402677	1.08311	-0.32624	1.409346
ILMN_3183139	LOC100130914	1.12998	0.279363	1.409343
ILMN_1805999	MYO1D	1.32159	-0.08764	1.4092279
ILMN_1789965	LOC643313	0.250937	1.15803	1.408967

ILMN_3191647	FLJ32790	-1.06648	-0.34237	1.408847
ILMN_3310643	MIR886	-0.67386	0.733255	1.407117
ILMN_1679797	ADARB1	-0.76824	-0.63877	1.407008
ILMN_1729421	LOC649754	0.314357	-1.09197	1.406327
ILMN_1711627	SIAH1	0.512812	-0.89339	1.4062
ILMN_1780937	MUS81	-0.76068	0.645286	1.405963
ILMN_1744623	MGC57359	-0.2912	1.1145	1.405695
ILMN_1791375	STAG3L2	0.130065	1.27506	1.405125
ILMN_3238560	IFI27L2	0.23176	-1.1733	1.40506
ILMN_3239709	LOC441666	-0.96677	0.438193	1.404964
ILMN_1718303	PVRL2	-1.13053	0.274321	1.404851
ILMN_1782329	HIST1H4L	1.32462	0.079554	1.4041739
ILMN_1712389	CKLF	-0.46689	-0.93697	1.403863
ILMN_2163306	FAM120A	0.727182	-0.6765	1.403684
ILMN_1696749	LMNA	0.756783	-0.64655	1.403336
ILMN_2118472	C10orf58	-0.02558	1.37746	1.4030398
ILMN_3199916	LOC100130980	1.37244	0.030522	1.4029619
ILMN_1685369	SLU7	-1.24122	-0.16146	1.402684
ILMN_2396786	ZNF266	-1.22284	-0.17854	1.401382
ILMN_2173524	FOXD4	0.336398	-1.06492	1.401318
ILMN_1720344	NIPA2	1.18855	-0.21259	1.401141
ILMN_2336781	SOD2	-1.30543	-0.09557	1.4010032
ILMN_1720124	RCC2	0.613393	-0.78758	1.400977
ILMN_2134224	ATP13A1	-0.61219	-0.78873	1.400925
ILMN_2232177	ACTN1	0.955876	0.444554	1.40043
ILMN_2082893	LOC440157	0.553951	0.846326	1.400277
ILMN_1808590	GUCY1A3	0.45353	-0.94667	1.400199
ILMN_2085525	SNORA32	0.169215	-1.23079	1.400005
ILMN_3281309	LOC100131850	-0.19229	-1.2073	1.399586
ILMN_2052871	TMEM116	1.34133	-0.05786	1.3991853
ILMN_3232048	LOC100128405	0.038715	-1.3601	1.3988153
ILMN_2404795	SULT1A1	0.096883	1.30185	1.3987332
ILMN_2133784	PATE2	0.309503	-1.08916	1.398663
ILMN_3310491	MIR1978	-0.28634	1.11219	1.398531
ILMN_1651554	RGS5	-0.3753	-1.02319	1.398494
ILMN_1787410	EIF6	1.18345	0.215028	1.398478
ILMN_2135456	FLJ32784	-0.66177	-0.73608	1.397851
ILMN_1721623	APOO	0.072534	1.325	1.3975339
ILMN_1792014	FAM70B	0.126962	-1.27042	1.397382
ILMN_1662426	AP2S1	1.28992	0.107384	1.397304
ILMN_1671992	LOC650128	-0.70665	0.690578	1.397225
ILMN_1792689	HIST1H2AC	-1.3492	0.04802	1.3972198
ILMN_3246821	MYO1H	0.070067	1.32712	1.3971871
ILMN_1713031	PGCP	1.02566	0.370675	1.396335
ILMN_1749776	LOC642816	0.040466	1.35585	1.3963159
ILMN_1765696	FARSLB	0.245685	1.15051	1.396195
ILMN_2042771	PTTG1	0.507322	0.888298	1.39562
ILMN_2083334	PMS2L5	0.119402	-1.27609	1.395492
ILMN_3240062	LOC729369	0.051129	1.34426	1.3953887
ILMN_2394561	IRF2BP2	-0.21441	1.18088	1.395287
ILMN_1711516	ATP6V1A	-0.81861	-0.57657	1.395174
ILMN_1741521	LOC650346	0.060518	1.33386	1.3943783

ILMN_1798298	CLU	-0.33615	1.05787	1.394024
ILMN_2315569	N6AMT1	0.570105	-0.82382	1.393927
ILMN_3236220	LPAR2	0.544482	-0.84942	1.393903
ILMN_2338963	SLC29A1	0.697422	-0.69644	1.393865
ILMN_1699383	POLR2J4	0.467296	0.926004	1.3933
ILMN_1760556	C1orf63	0.514881	-0.8783	1.393185
ILMN_3236904	LOC648740	1.08677	0.306263	1.393033
ILMN_1768311	LOC728888	1.2051	0.187766	1.392866
ILMN_1735552	KIF1B	0.828877	-0.56366	1.392539
ILMN_2394571	FBXW11	0.298522	-1.09388	1.392402
ILMN_2394438	FAM177A1	-0.53175	0.86054	1.392285
ILMN_1802627	PSMG3	0.159985	1.23211	1.392095
ILMN_1748823	LOC374920	-0.78001	0.61187	1.391883
ILMN_3191393	LOC100128892	0.450029	-0.94101	1.391041
ILMN_1777344	RPS13	-0.47596	0.914353	1.390317
ILMN_3248672	FMN1	-1.20263	0.187618	1.390248
ILMN_1699521	KIAA1641	1.02097	-0.36908	1.390048
ILMN_3238818	LOC649076	0.498409	-0.89155	1.389957
ILMN_1702396	PACSIN2	-0.56572	0.824212	1.389934
ILMN_3248928	UBN2	0.271785	1.11794	1.389725
ILMN_2160210	TACSTD1	1.21473	0.174967	1.389697
ILMN_2139761	LIMCH1	1.11868	-0.27077	1.389445
ILMN_3287722	LOC100131649	-0.27655	1.11267	1.389224
ILMN_3213692	LOC441073	0.518541	-0.87056	1.389105
ILMN_1806294	RPS6KA3	1.36234	0.026569	1.388909
ILMN_2087060	TOMM7	-0.13006	1.25879	1.388849
ILMN_1695583	LOC644094	-0.30162	-1.0872	1.388819
ILMN_1675472	LOC644799	1.04189	-0.34672	1.388612
ILMN_3290417	LOC388401	0.356451	-1.03211	1.388561
ILMN_2147133	NBPF15	1.34467	0.04386	1.3885298
ILMN_1763875	ABCF1	-0.93956	0.44888	1.388437
ILMN_3192419	LOC100128485	1.052	-0.33631	1.388307
ILMN_1773493	TIMM23	0.815588	-0.57241	1.388001
ILMN_1668721	CCND3	0.890069	-0.49793	1.387999
ILMN_1754489	FBXL20	0.711506	0.676175	1.387681
ILMN_2096719	GRK5	-0.3796	-1.0076	1.387201
ILMN_3243356	LOC100131963	1.24735	-0.13956	1.386914
ILMN_3247390	LOC100132347	0.421223	-0.96558	1.386804
ILMN_1806651	PARP8	0.503343	0.883411	1.386754
ILMN_1745343	ZMAT2	0.201442	1.18485	1.386292
ILMN_3306444	LOC729828	0.520133	-0.8651	1.38523
ILMN_3302499	LOC730990	0.291201	1.09246	1.383661
ILMN_1684549	RNPC2	1.13746	0.245722	1.383182
ILMN_2359096	SS18	-0.36703	1.01562	1.382648
ILMN_2073520	C2orf21	-0.18711	1.19541	1.382523
ILMN_2378952	GPX4	0.074921	-1.30736	1.3822811
ILMN_1804142	LOC643444	0.110649	1.27084	1.381489
ILMN_1761566	C5orf32	0.494351	-0.88684	1.381188
ILMN_2106331	H3F3A	0.648882	-0.73226	1.381138
ILMN_1651569	XRCC2	0.385256	0.995505	1.380761
ILMN_3243529	FDXACB1	-0.17239	1.20677	1.379162
ILMN_1696757	TTC14	-0.99852	-0.3802	1.378718

ILMN_1686388	LOC644330	-0.81877	-0.5599	1.378663
ILMN_3278170	LOC441455	0.27274	1.10584	1.37858
ILMN_1672830	LOC642082	0.372923	1.00565	1.378573
ILMN_2318725	EEF1B2	0.286665	1.09176	1.378425
ILMN_2367883	GEM	0.662967	-0.71446	1.377429
ILMN_1754195	RPL31	0.653259	0.724113	1.377372
ILMN_1691892	TAGLN2	0.499444	-0.87778	1.377222
ILMN_2318011	PSMA3	0.630501	-0.74637	1.37687
ILMN_1782069	TRAK1	0.865799	-0.51094	1.376742
ILMN_1762573	LOC401630	-0.16173	1.21431	1.37604
ILMN_1653180	TPM4	0.23725	-1.13836	1.37561
ILMN_1680279	USP49	-0.16714	1.20831	1.375451
ILMN_1784602	CDKN1A	-0.04237	1.33294	1.3753098
ILMN_3266666	LOC100128196	0.589759	-0.78534	1.3751
ILMN_1782292	LAMP1	0.767287	-0.60701	1.3743
ILMN_1654262	ZMAT3	-1.26079	0.113491	1.374281
ILMN_2389851	MDM2	-0.77381	-0.60024	1.374049
ILMN_2201533	C17orf61	0.44231	0.931656	1.373966
ILMN_3250984	CNBP	0.304569	-1.06923	1.373799
ILMN_1762624	LOC641852	-1.10961	0.263652	1.373262
ILMN_3242535	LOC100133568	0.141763	-1.23148	1.373243
ILMN_1753243	DNAJB11	0.521486	-0.8512	1.372689
ILMN_3232753	LOC728054	1.31491	0.05744	1.3723504
ILMN_3250257	ACVRL1	0.344356	-1.0279	1.372256
ILMN_2405233	FAM133B	0.572174	-0.80001	1.372185
ILMN_1714700	TRIB2	-0.4205	-0.95157	1.372069
ILMN_1803277	MVP	0.74063	0.631179	1.371809
ILMN_1771734	LOC728564	0.044365	1.32734	1.371705
ILMN_1705144	ULK1	-0.15672	1.21492	1.371636
ILMN_3226291	LOC728820	-0.4548	-0.91645	1.371245
ILMN_2415722	RPS29	0.796888	0.574259	1.371147
ILMN_3251399	SLC17A5	0.128713	1.24232	1.371033
ILMN_1792528	LOC401206	-0.29732	1.07363	1.370952
ILMN_1657436	FGFR1OP2	0.965186	-0.40568	1.370869
ILMN_1691611	LOC645436	1.12067	-0.25013	1.370801
ILMN_1724293	KDELR2	0.632968	-0.73766	1.370632
ILMN_3278132	LOC440487	0.417006	-0.95349	1.370492
ILMN_2145670	TNC	-0.18298	-1.18728	1.370256
ILMN_1756696	USF2	0.536923	-0.83276	1.369681
ILMN_1754772	LOC650698	-0.04691	1.32268	1.3695855
ILMN_1706511	TEF	0.927945	-0.44156	1.369506
ILMN_1674236	HSPB1	0.429977	-0.93872	1.368693
ILMN_3275936	LOC100133277	0.509709	0.858806	1.368515
ILMN_1778237	FN1	0.269239	-1.09901	1.368249
ILMN_3249327	LOC100134530	0.973541	0.39422	1.367761
ILMN_1774111	LOC651643	-0.26525	-1.10243	1.367684
ILMN_1715508	NNMT	-0.8007	0.566655	1.367357
ILMN_2332713	SLC25A3	0.246799	-1.11975	1.366549
ILMN_3304887	LOC729423	0.716599	0.649831	1.36643
ILMN_1779751	C7orf55	-0.37292	0.993167	1.366084
ILMN_1762861	HLA-F	-0.05709	-1.30892	1.3660108
ILMN_2103480	ZNF320	0.130702	1.23523	1.365932

ILMN_1667050	PRPS1	0.365602	-1.00021	1.365812
ILMN_1727153	LOC390414	-0.59015	0.775636	1.365787
ILMN_1660847	PFKFB3	1.16149	0.204199	1.365689
ILMN_1777668	LOC647949	0.729092	-0.63652	1.365609
ILMN_3292056	LOC727984	-0.35716	1.00843	1.365591
ILMN_1696485	HNRNPAB	0.65111	-0.71443	1.365542
ILMN_2307025	CPNE1	0.388201	-0.97694	1.365144
ILMN_3194755	LOC100128737	0.821079	-0.54399	1.365071
ILMN_2340065	UBL5	-1.10754	0.25745	1.36499
ILMN_3256024	LOC100128035	-0.0146	1.35035	1.3649488
ILMN_2377977	MTRR	0.245208	-1.11971	1.364918
ILMN_1794068	RFX4	-0.4131	0.951804	1.364905
ILMN_1719759	TNC	0.605276	-0.75941	1.364687
ILMN_1775111	SND1	1.04563	-0.31852	1.364149
ILMN_2110829	LOC441743	0.864128	-0.49995	1.364076
ILMN_2395981	PYHIN1	0.770629	-0.59266	1.363286
ILMN_1752668	DAAM2	-0.93136	-0.43159	1.362951
ILMN_1795344	GOLPH4	0.378811	-0.98375	1.362565
ILMN_3239694	LOC100134412	0.968528	-0.39372	1.362246
ILMN_2185563	ANKRA2	-0.37674	0.985161	1.361897
ILMN_3238915	LOC100133060	1.16595	0.195678	1.361628
ILMN_1794072	B3GAT1	0.583314	0.778281	1.361595
ILMN_1691402	LOC644162	0.621509	-0.73885	1.360363
ILMN_1705871	DDHD2	0.316346	1.04388	1.360226
ILMN_1683044	PPP1R2	1.26358	-0.09622	1.3597982
ILMN_2327276	STAU1	-0.76354	-0.59607	1.359612
ILMN_1713682	FBXO11	0.203193	1.15555	1.358743
ILMN_2339835	PTGS1	0.389872	-0.96862	1.358489
ILMN_1910645	LOC730841	0.318972	1.03939	1.358362
ILMN_1702231	C1orf54	0.665911	-0.69237	1.358283
ILMN_2136133	PABPC1	0.857364	0.500575	1.357939
ILMN_1744210	SDHA	0.973302	-0.38419	1.357487
ILMN_2230624	RPL18	0.928343	0.428701	1.357044
ILMN_1698258	DNAJC8	-0.5962	-0.75938	1.35558
ILMN_1652379	SUCLG2	0.686282	0.669246	1.355528
ILMN_1683271	TMSB4X	-0.79577	-0.55894	1.354707
ILMN_1752798	LOC642780	0.065452	1.28908	1.3545318
ILMN_3242833	LOC730375	0.617928	-0.7365	1.354432
ILMN_1688698	ZEB2	0.728694	-0.62573	1.354424
ILMN_3246514	LOC100134182	0.291042	1.06337	1.354412
ILMN_1798705	CCNC	0.792591	-0.56126	1.35385
ILMN_3251723	PNPT1	0.447721	0.905216	1.352937
ILMN_1773262	ESM1	0.31197	-1.0409	1.35287
ILMN_2133534	SMA4	-0.05439	-1.29811	1.3524953
ILMN_3241335	LOC100132550	0.129827	-1.22266	1.352487
ILMN_3223181	SEC13	1.21043	0.141942	1.352372
ILMN_1747589	HIST2H2AB	0.309184	-1.04272	1.351904
ILMN_2162564	DYNLT3	0.912349	0.43911	1.351459
ILMN_1741204	KLHDC2	0.550609	-0.79976	1.350371
ILMN_1693789	ALPP	0.02861	1.32152	1.3501295
ILMN_3244666	LOC729387	1.23844	0.111496	1.349936
ILMN_1665865	IGFBP4	0.957865	0.391859	1.349724

ILMN_1804148	TMED4	0.458384	-0.89071	1.349091
ILMN_1737878	FLJ34047	-0.36066	0.987753	1.348415
ILMN_2227368	SELT	0.086459	-1.2615	1.3479592
ILMN_1705861	AP1M2	0.440401	-0.90739	1.34779
ILMN_1655046	NUTF2	-0.01492	1.33249	1.347407
ILMN_3181565	FLJ40672	0.382392	0.964852	1.347244
ILMN_3222998	LOC729513	0.995742	-0.3507	1.34644
ILMN_3294282	LOC388907	0.093939	-1.25242	1.346359
ILMN_2280911	VPS41	-0.25674	1.08954	1.34628
ILMN_1660999	WBP11P1	0.77795	0.568022	1.345972
ILMN_1680353	NSF	0.684531	-0.66129	1.345825
ILMN_1731353	CHPF	-1.04245	0.303311	1.345761
ILMN_1810488	NFYC	1.08733	-0.25785	1.345183
ILMN_2076640	KHDRBS1	-1.09138	0.253681	1.345061
ILMN_1677098	YPEL2	0.407457	-0.93726	1.344717
ILMN_1673601	LRRC37B	0.348653	-0.99564	1.344295
ILMN_1773850	FXC1	0.562784	0.781157	1.343941
ILMN_3307611	LOC400464	-0.41525	0.928691	1.343941
ILMN_1656791	RPS6	-0.21934	1.12387	1.34321
ILMN_1661439	FLOT1	-1.23891	0.103585	1.342495
ILMN_3267232	LOC100129808	-0.65333	-0.68885	1.342184
ILMN_1799614	PNPLA6	0.156245	-1.18575	1.341995
ILMN_1815169	MCM5	0.238046	1.10379	1.341836
ILMN_2325347	B3GALNT1	0.46093	0.880836	1.341766
ILMN_2382083	CUGBP1	-0.44231	0.89934	1.341645
ILMN_3279277	LOC387930	-0.22491	1.11661	1.341521
ILMN_1750368	LOC642946	-0.27902	1.06248	1.3415
ILMN_1815039	C6orf153	-0.85274	0.487941	1.340684
ILMN_1726783	RNASEH1	0.847576	0.493054	1.34063
ILMN_2168952	DENR	-0.12274	1.21766	1.340399
ILMN_2378868	SFRS5	0.893968	0.446371	1.340339
ILMN_1734939	LOC654254	-0.02685	1.31335	1.340203
ILMN_1763989	SEC63	0.899219	-0.4406	1.339815
ILMN_1785265	PLS3	1.02017	-0.31961	1.339778
ILMN_1779279	C14orf32	1.1681	-0.17157	1.339665
ILMN_2048811	NUBPL	0.432364	0.907246	1.33961
ILMN_1750075	DMTF1	-0.04086	-1.29874	1.3395979
ILMN_2046730	S100A10	-0.83444	-0.50485	1.339295
ILMN_1778788	AMOTL2	-0.96789	0.371397	1.339282
ILMN_3278745	LOC253482	0.56533	-0.77336	1.338688
ILMN_3239397	LOC100134253	0.374594	0.963876	1.33847
ILMN_1773742	DNAJB9	0.546392	-0.79206	1.33845
ILMN_1707775	ZNF257	-0.53556	-0.80246	1.338019
ILMN_3231550	LOC100131718	-0.2799	1.05778	1.337676
ILMN_3243492	LOC729501	-0.95706	-0.38044	1.337502
ILMN_2262203	PMS2CL	0.116856	1.2206	1.337456
ILMN_1813139	ANKDD1A	-0.01691	-1.31949	1.3363964
ILMN_2409167	ANXA2	0.928582	-0.4077	1.336283
ILMN_2247664	SON	-0.03688	-1.29885	1.3357292
ILMN_2129015	AFF1	-0.16905	-1.1666	1.33565
ILMN_1757019	NTN4	-0.65349	-0.68201	1.335497
ILMN_3179762	LOC100130483	-0.30631	-1.0291	1.335414

ILMN_1728019	PML	-0.48838	-0.84698	1.335354
ILMN_3281988	LOC728157	0.651429	-0.68372	1.33515
ILMN_1760676	MORF4L1	0.519098	-0.81595	1.335044
ILMN_1739253	STAMBP	0.111206	-1.22368	1.334886
ILMN_1656110	LOC440261	0.338308	-0.99645	1.33476
ILMN_1696394	IL6R	0.365841	-0.9689	1.334736
ILMN_2379226	CCNL2	0.76124	0.57304	1.33428
ILMN_3192289	LOC100129195	0.792591	0.541535	1.334126
ILMN_1772487	SFRS14	0.113753	-1.22031	1.334063
ILMN_1769637	RNMT	-1.21902	-0.11487	1.33389
ILMN_1705302	FCGRT	0.605356	0.728397	1.333753
ILMN_3244192	LOC729439	-0.3134	1.02012	1.333516
ILMN_2319414	BTF3	-0.41493	0.918359	1.33329
ILMN_3244821	LOC100128276	0.008318	1.32497	1.33328832
ILMN_2275803	LRRC45	0.788613	-0.5445	1.333108
ILMN_1778381	LOC650029	-0.99637	-0.3367	1.33307
ILMN_1689747	NOL8	0.300511	1.03254	1.333051
ILMN_1719064	KCTD10	1.03998	0.292854	1.332834
ILMN_1783060	SLC25A20	0.598353	0.734143	1.332496
ILMN_1779486	FAM126B	0.49085	0.841468	1.332318
ILMN_1704477	COX5A	-0.0029	-1.3294	1.3323015
ILMN_3244579	LOC649330	0.319529	-1.01247	1.331999
ILMN_1775830	TM4SF20	0.124734	-1.20708	1.331814
ILMN_1771800	PRKCA	-0.06529	1.26647	1.3317569
ILMN_1798975	EGFR	0.352631	0.978551	1.331182
ILMN_1773849	ATP6V0C	0.472071	0.859096	1.331167
ILMN_2125675	LOC728643	0.683178	0.64773	1.330908
ILMN_1751627	TRAPPC3	0.2429	-1.08762	1.33052
ILMN_2216582	LYL1	0.057972	1.27251	1.330482
ILMN_3178252	LOC100129379	0.641402	0.688969	1.330371
ILMN_1742224	SLTM	0.419871	0.910477	1.330348
ILMN_2360710	TPM1	-0.65604	-0.6743	1.330338
ILMN_2347798	IFI6	0.996856	-0.33319	1.33005
ILMN_1715947	LOC648210	0.021687	1.30806	1.3297466
ILMN_1651235	AFAP1	0.457827	0.871901	1.329728
ILMN_1740900	BMP4	0.944337	-0.38519	1.329522
ILMN_3284607	LOC728207	0.958104	-0.37096	1.329064
ILMN_2401906	CDAN1	-0.22618	1.1028	1.328984
ILMN_2395856	TMEM218	0.597478	-0.73145	1.328923
ILMN_3205837	LOC727833	0.071897	1.25605	1.3279473
ILMN_1671250	CLIC4	0.832537	-0.49537	1.327911
ILMN_3239606	LOC100134357	0.377617	0.950059	1.327676
ILMN_1678546	PEX11B	0.653179	0.674418	1.327597
ILMN_1779780	CHTF8	-0.40483	0.922022	1.326848
ILMN_1715693	LOC440160	0.562227	-0.76428	1.326502
ILMN_2298365	PPP2R2B	-0.01404	1.31215	1.3261917
ILMN_1716555	MGC42630	-0.86022	0.465425	1.325648
ILMN_2330495	OCIAD1	-0.0255	-1.29996	1.3254603
ILMN_2166506	XRCC6	0.315471	-1.00975	1.325221
ILMN_1786125	CCNA2	0.226985	1.09774	1.324725
ILMN_1746428	RGPD1	0.799196	-0.52523	1.324424
ILMN_3302350	LOC728728	1.24528	-0.07865	1.3239292

ILMN_2313821	AIFM1	0.234147	-1.08978	1.323927
ILMN_3287784	LOC100132153	1.27711	0.046771	1.3238812
ILMN_1714567	AHNAK	0.082321	1.24139	1.3237114
ILMN_1802907	CBWD1	0.8619	-0.46169	1.323586
ILMN_3271179	LOC100128775	0.991763	0.33172	1.323483
ILMN_2233401	FTHL8	0.745882	-0.5775	1.323384
ILMN_3307700	SPCS3	-0.7407	-0.58248	1.323183
ILMN_3270833	LOC100129547	1.23741	-0.08573	1.3231424
ILMN_3241798	SNORA41	-0.0524	1.27069	1.323086
ILMN_1663664	MRPS10	0.797843	-0.52491	1.322751
ILMN_2108357	RPL39L	0.257223	1.06501	1.322233
ILMN_1707815	SDHALP1	1.07921	0.242929	1.322139
ILMN_1739257	EIF3E	0.997094	0.324418	1.321512
ILMN_1777118	INTS9	-1.12918	-0.19221	1.321385
ILMN_3201986	LOC100132795	0.825535	-0.49558	1.32111
ILMN_1726460	RPL14	-1.30488	0.016048	1.3209278
ILMN_3225300	LOC728532	0.257541	-1.06332	1.320861
ILMN_1723141	OTUD1	0.235341	-1.08508	1.320421
ILMN_1770473	LOC647326	0.752805	-0.56758	1.320384
ILMN_1776577	DSCC1	0.073728	1.24646	1.3201875
ILMN_1677237	CHCHD9	0.578699	-0.74145	1.32015
ILMN_1784717	RPS19	1.20367	-0.11645	1.32012
ILMN_1807873	SNX6	1.0477	-0.27146	1.319164
ILMN_1745798	GTF2F2	0.022642	-1.29594	1.3185815
ILMN_3279996	LOC649395	0.881872	-0.43659	1.318462
ILMN_1724907	NUDT3	0.085504	-1.23293	1.3184343
ILMN_1750693	SSR1	0.392418	-0.92596	1.318382
ILMN_3182735	LOC100129518	0.323826	0.993854	1.31768
ILMN_3199172	LOC100132430	1.14478	-0.1722	1.316984
ILMN_1658302	PIAS2	0.223643	1.09297	1.316613
ILMN_1734696	FRG1	0.61769	-0.6987	1.316388
ILMN_2129161	LRRC32	0.26518	-1.0512	1.31638
ILMN_3248443	SNHG6	-0.42122	-0.89496	1.31618
ILMN_1772261	GLG1	-0.14263	-1.17354	1.316172
ILMN_3239388	LOC100133950	-0.89754	0.418488	1.316031
ILMN_3234384	LOC728953	-0.37308	0.942479	1.315555
ILMN_1802553	MRPS24	-1.11239	-0.20264	1.315028
ILMN_2294274	S100PBP	-0.43156	0.883268	1.31483
ILMN_1737124	PRPF4B	-1.17525	0.139433	1.314683
ILMN_1689119	ZC3H5	-0.91171	0.402765	1.314472
ILMN_1756071	MFGE8	-0.461	-0.8534	1.314402
ILMN_1704750	LOC647000	-0.62962	-0.68421	1.313832
ILMN_1785926	ZNF621	-0.24759	1.06624	1.313829
ILMN_3263363	LOC100127984	-0.52705	-0.78642	1.313474
ILMN_1730487	CALD1	0.151868	-1.16145	1.313318
ILMN_3237623	RNY1	0.10309	-1.21017	1.31326
ILMN_3206429	LOC646936	-1.07969	-0.23356	1.313246
ILMN_3271244	LOC100130775	0.536923	0.776322	1.313245
ILMN_1675523	PPAP2C	0.069351	-1.24353	1.3128809
ILMN_3203515	LOC401717	0.037044	1.27557	1.3126142
ILMN_2368713	TMEM189-UBE2	0.215925	-1.09663	1.312555
ILMN_3244110	FAM156B	0.196191	1.116	1.312191

ILMN_2366463	FN1	0.242661	-1.06948	1.312141
ILMN_1761996	SFRS5	1.05638	-0.25493	1.31131
ILMN_3229203	LOC728711	-0.14017	1.17108	1.311245
ILMN_1735877	EFEMP1	0.632172	-0.67886	1.311029
ILMN_2090105	TAGLN2	0.971711	0.33917	1.310881
ILMN_1718128	PABPC3	0.863809	-0.44654	1.310352
ILMN_1724424	PRRT2	-0.28197	1.02818	1.310145
ILMN_3238053	LOC100129211	0.079457	1.23057	1.3100267
ILMN_1659437	TXNDC17	0.389235	-0.92076	1.309991
ILMN_1773966	LOC645232	1.01341	-0.29646	1.309869
ILMN_1745607	A2M	-0.20374	-1.10599	1.309734
ILMN_2222786	ZNF192	0.619918	-0.68925	1.309172
ILMN_1686954	RPS10	0.738561	0.570543	1.309104
ILMN_1660227	LOC646434	0.520451	0.788631	1.309082
ILMN_1796458	GABARAPL2	1.23589	0.07295	1.30884
ILMN_1797596	SPRY4	0.06211	-1.24625	1.3083598
ILMN_2140059	LAMA4	0.08996	-1.21839	1.3083504
ILMN_1714820	ITGB1	0.663603	-0.6438	1.307405
ILMN_1779852	LOC387934	0.764582	-0.54278	1.307361
ILMN_2278653	ZNF493	0.371331	0.93552	1.306851
ILMN_1775058	CSNK1A1	-0.54822	0.758534	1.30675
ILMN_1667030	HSBP1	0.325815	-0.98035	1.306166
ILMN_1676980	MTSS1	1.1408	-0.16439	1.305187
ILMN_1661335	SPTBN1	0.544164	-0.76019	1.304356
ILMN_3249496	LOC653340	1.02089	0.282552	1.303442
ILMN_1664706	LOC653604	0.923251	-0.37945	1.302696
ILMN_2260833	CCNB1IP1	0.533103	-0.76952	1.302627
ILMN_1744023	MGC18216	0.367193	0.935265	1.302458
ILMN_1675501	SENP5	0.423849	0.87854	1.302389
ILMN_3283470	LOC644937	0.450109	0.852018	1.302127
ILMN_3207722	LOC442181	0.982533	-0.31925	1.301786
ILMN_2230035	BBS2	0.332261	0.969486	1.301747
ILMN_1714093	RMND5A	-0.72368	0.57791	1.301585
ILMN_1731907	LOC643313	0.933356	0.368207	1.301563
ILMN_3245829	AQP7P1	1.05932	-0.24206	1.30138
ILMN_3251742	ZNF322A	-1.14167	-0.15968	1.301347
ILMN_1814173	SMARCA4	0.424088	-0.87724	1.301327
ILMN_1805028	THOC7	0.428942	-0.87236	1.301305
ILMN_3237516	SNRNP48	-0.21433	1.08695	1.301277
ILMN_1744109	OR2A42	0.585781	-0.71509	1.30087
ILMN_1687508	ALDH7A1	1.04476	-0.25597	1.300732
ILMN_2350634	EFEMP1	0.556498	-0.74293	1.299429
ILMN_1788468	ARGLU1	-0.1194	-1.17985	1.299247
ILMN_1793950	P704P	1.10929	-0.18974	1.299034
ILMN_2294878	NSUN5	0.494669	0.803158	1.297827
ILMN_1665655	CTDSPL2	1.09561	0.202104	1.297714
ILMN_2109708	ECGF1	0.647052	-0.65057	1.297623
ILMN_2095653	AFMID	-0.4326	0.864883	1.29748
ILMN_1757278	LOC643300	0.680075	0.617303	1.297378
ILMN_1669273	PPT1	0.942985	-0.35425	1.297234
ILMN_1805750	IFITM3	0.753203	0.543725	1.296928
ILMN_1689142	UBE1C	0.799594	0.497036	1.29663

ILMN_2333594	SUMO2	-0.61156	0.685016	1.296573
ILMN_1687738	LOC650276	0.805641	0.490616	1.296257
ILMN_1806408	ACADVL	1.20438	-0.09186	1.296237
ILMN_1679725	PCYOX1	0.775881	-0.52023	1.296108
ILMN_1801616	EMP1	0.725511	-0.57005	1.295563
ILMN_2205211	LOC134997	-0.25929	-1.03615	1.295436
ILMN_1761519	EIF4G2	0.694159	0.600473	1.294632
ILMN_1702809	LOC388237	0.174547	-1.12006	1.294607
ILMN_1696839	RPS27	0.540742	0.753741	1.294483
ILMN_3291511	LOC100131971	-0.22356	-1.07076	1.294318
ILMN_1662232	DCTN2	0.133009	-1.16122	1.294229
ILMN_1721651	LOC646463	0.327248	0.966811	1.294059
ILMN_1703123	AXUD1	0.512971	-0.78045	1.293424
ILMN_1736256	CALR	-0.58315	-0.71025	1.293398
ILMN_2175114	KCNS3	0.319609	-0.97349	1.293102
ILMN_1726769	CNDP2	1.08311	-0.20995	1.293062
ILMN_3197767	LOC645691	0.695592	-0.59742	1.293013
ILMN_2352590	ZNF33A	0.515756	-0.77636	1.29212
ILMN_2104141	FGD5	-0.36448	-0.92755	1.292032
ILMN_3206866	LOC100132593	0.27839	-1.01359	1.29198
ILMN_1724811	PARN	-0.45225	-0.83959	1.291838
ILMN_2196337	C12orf11	0.202079	-1.08932	1.291399
ILMN_1749634	PLRG1	-0.21751	1.0734	1.29091
ILMN_2318869	UBE2V1	0.974337	0.31498	1.289317
ILMN_1663751	CYCSL1	0.617212	-0.67207	1.289281
ILMN_1756311	UFSP2	-0.41207	0.876895	1.288962
ILMN_1654430	LOC731170	0.587054	-0.70172	1.28877
ILMN_1790625	CBX3	0.168022	-1.12045	1.288472
ILMN_3227994	LOC729992	0.768083	-0.52021	1.288293
ILMN_1770127	DNAJA2	-0.42711	0.860995	1.288101
ILMN_1669566	FAM169A	0.439764	0.846539	1.286303
ILMN_1800697	LDB2	0.351279	-0.93468	1.285959
ILMN_1654543	MED6	-1.23605	0.049884	1.2859338
ILMN_1776094	PPCS	-0.63439	-0.65113	1.285521
ILMN_1691476	MYLK	0.0341	-1.25125	1.28535
ILMN_3224290	LOC730052	0.283562	-1.00146	1.285022
ILMN_1755290	CEP170	-0.07316	-1.21175	1.2849146
ILMN_2261784	CCNY	-1.09377	-0.19107	1.284839
ILMN_2402930	LOC440926	0.344833	-0.93889	1.28372
ILMN_1790603	ATPAF1	0.32868	-0.9548	1.283479
ILMN_1683354	LOC644694	0.834208	-0.44811	1.282314
ILMN_1661264	SHMT2	1.00919	0.272954	1.282144
ILMN_2186715	CENPH	0.551485	-0.73063	1.282113
ILMN_2106002	ACBD7	-0.20629	1.0757	1.281991
ILMN_1709294	CDCA8	-0.25889	1.02284	1.281728
ILMN_1734312	GCN1L1	0.885374	0.395854	1.281228
ILMN_3245441	LOC100133042	0.311731	-0.96913	1.280862
ILMN_3225406	LOC728640	0.778189	-0.50243	1.280623
ILMN_1658978	LOC650155	0.741983	-0.53837	1.280353
ILMN_1749009	REXO2	-0.50875	0.771476	1.280224
ILMN_2180997	GTF2IRD2B	-0.30242	0.977723	1.280138
ILMN_3235168	MUL1	0.943542	0.336206	1.279748

ILMN_1805266	NBR1	-0.0173	-1.26223	1.2795342
ILMN_1794306	USP28	-0.67601	0.603195	1.279206
ILMN_1765326	DGKD	1.21107	-0.06789	1.2789554
ILMN_2351298	WIPF1	-1.24942	-0.02952	1.2789366
ILMN_1708486	CNN2	-0.7228	-0.556	1.278804
ILMN_1684054	ASAH1	-0.64896	0.629753	1.278709
ILMN_1776925	PRSS22	-0.43451	0.842645	1.277151
ILMN_1746359	RERG	0.185528	-1.09162	1.277148
ILMN_1748124	TSC22D3	0.358679	0.917957	1.276636
ILMN_1758214	RARS2	1.20518	-0.07127	1.2764502
ILMN_1792314	ACTR1A	-1.05669	0.219395	1.276085
ILMN_1749014	ACLY	0.021289	-1.25479	1.2760787
ILMN_1804834	C6orf130	-0.52602	0.749593	1.275608
ILMN_1751586	AQP7P2	0.766173	-0.50899	1.275163
ILMN_1756139	LOC643310	-0.43164	0.843213	1.274855
ILMN_1739025	LOC645367	-0.69821	0.576626	1.274838
ILMN_2348268	IFFO1	0.834447	0.440116	1.274563
ILMN_1815308	SDC1	-0.53485	0.739652	1.2745
ILMN_3227060	LOC728931	-0.85768	0.416807	1.274483
ILMN_2047206	TMX3	0.65302	0.621303	1.274323
ILMN_1746923	LOC646443	0.735776	0.538228	1.274004
ILMN_1741398	KIAA0406	0.495386	0.778577	1.273963
ILMN_1652230	LOC647361	0.308389	0.965563	1.273952
ILMN_1772702	SFRS2B	0.722249	-0.55153	1.273774
ILMN_3240022	SNORA73A	0.318415	-0.95519	1.273605
ILMN_3288726	LOC650901	0.256587	1.01675	1.273337
ILMN_3251728	MTMR10	-0.42361	0.849527	1.273132
ILMN_2367070	ACOT9	-0.09003	-1.18285	1.2728841
ILMN_2117508	CTHRC1	0.452257	-0.82026	1.272517
ILMN_1748105	DDB1	-0.11606	-1.15646	1.272515
ILMN_2379835	SUMO1	0.856568	0.415813	1.272381
ILMN_3241996	C6orf59	0.328043	0.944094	1.272137
ILMN_1653687	GALNT9	-1.22984	0.041848	1.2716879
ILMN_1702279	KIF3B	0.548222	-0.72325	1.271471
ILMN_2362858	PILRA	-0.45774	0.813673	1.271415
ILMN_2370091	NGFRAP1	1.02359	0.247811	1.271401
ILMN_2122511	CCBE1	-0.16563	1.10563	1.271259
ILMN_3279675	LOC388339	0.146378	1.1243	1.270678
ILMN_1718311	WBSCR19	0.99041	0.280073	1.270483
ILMN_1688413	SNW1	0.182584	1.08776	1.270344
ILMN_2135898	GPR42	0.945929	0.3244	1.270329
ILMN_1683969	FKBP1A	0.965186	0.304743	1.269929
ILMN_2053103	SLC40A1	0.611165	0.658293	1.269458
ILMN_3292244	LOC100132804	0.50581	-0.76342	1.269233
ILMN_2053415	LDLR	0.80031	-0.46883	1.269138
ILMN_1736567	CD74	0.316585	0.952367	1.268952
ILMN_2168866	C6orf62	0.142877	-1.12584	1.268717
ILMN_1652806	ATP5J	-0.43053	0.838183	1.268711
ILMN_3239060	KRBA1	-0.39942	0.869274	1.268689
ILMN_1781097	UBXN4	0.730286	0.538399	1.268685
ILMN_1668463	SON	-1.18981	-0.07861	1.2684178
ILMN_1781285	DUSP1	1.0477	0.220685	1.268385

ILMN_2377240	AKTIP	0.590476	-0.67758	1.26806
ILMN_3187479	LOC100129982	0.818612	-0.44942	1.268031
ILMN_1759154	PABPN1	0.730683	0.537299	1.267982
ILMN_1684461	CADPS2	0.841211	0.42608	1.267291
ILMN_1679587	LOC651202	0.405945	0.861202	1.267147
ILMN_1807074	MIF	0.857364	0.409695	1.267059
ILMN_3247533	LOC100133840	0.778268	-0.48847	1.266742
ILMN_3210917	LOC389168	0.639493	0.627226	1.266719
ILMN_1735156	SLC4A11	-0.01014	1.25644	1.2665827
ILMN_1715396	SNORD56B	0.184891	-1.08169	1.266581
ILMN_3308225	MIR1282	0.756386	0.510108	1.266494
ILMN_1807304	MBNL1	0.849805	-0.41657	1.266376
ILMN_1792860	MED22	0.305126	-0.96124	1.266364
ILMN_3243568	LOC100131835	0.129508	1.13654	1.266048
ILMN_2086105	SPRY4	-0.13682	-1.12912	1.265943
ILMN_1739821	EIF2S1	-1.22515	0.04015	1.2652996
ILMN_1686645	UTP14C	-0.96868	0.296399	1.26508
ILMN_1656208	LOC645304	0.985715	0.278948	1.264663
ILMN_2253017	LGMN	0.987387	0.277132	1.264519
ILMN_3278626	LOC100132811	0.553076	-0.71142	1.264496
ILMN_2113074	UFM1	0.198737	1.0654	1.264137
ILMN_1664103	LOC441081	-0.17963	1.08366	1.263293
ILMN_2413158	PODXL	0.362976	-0.90002	1.262992
ILMN_1657128	PTPRU	-0.47095	0.791601	1.262552
ILMN_2232712	MYO10	1.17399	0.088093	1.2620828
ILMN_1794534	CCDC81	0.383983	0.877481	1.261464
ILMN_3227732	LOC729789	0.615939	0.64505	1.260989
ILMN_1656962	LOC401019	0.233351	1.02632	1.259671
ILMN_1753913	GPR177	0.479869	0.779098	1.258967
ILMN_3279322	LOC645174	-0.22205	1.03616	1.258206
ILMN_2270845	WDR74	0.389235	0.86893	1.258165
ILMN_3256184	LOC100128498	-0.06927	1.18864	1.2579055
ILMN_1781560	ST3GAL6	0.141524	1.11493	1.256454
ILMN_3227811	LOC729423	0.744848	0.51109	1.255938
ILMN_1780894	ANTXR1	-0.88441	0.371474	1.255887
ILMN_3261938	LOC100130154	-0.69392	0.561844	1.255759
ILMN_1680618	MYC	1.03306	-0.22262	1.255681
ILMN_1768284	P2RY8	-0.08526	-1.17013	1.2553897
ILMN_1689097	NAT5	0.858876	0.396416	1.255292
ILMN_1739345	C11orf48	1.00036	0.254829	1.255189
ILMN_1689086	CTSC	1.2534	-0.00157	1.2549744
ILMN_1655497	EIF4B	-0.44461	0.81036	1.254972
ILMN_3211935	LOC100132715	-0.42949	0.825425	1.254918
ILMN_1722491	APRT	-0.59015	-0.66471	1.254865
ILMN_3201643	LOC100133019	0.189506	1.06516	1.254666
ILMN_3237483	LOC100132098	0.859353	0.394682	1.254035
ILMN_1654170	HERC2P2	0.404593	-0.84944	1.254032
ILMN_1737298	MAT2A	0.326372	0.927253	1.253625
ILMN_3251506	ZNF69	0.044285	-1.209	1.2532854
ILMN_2064898	CCDC56	0.158632	-1.09452	1.253152
ILMN_3305169	LOC728809	0.053436	1.19965	1.2530863
ILMN_3182451	LOC100129652	-0.03449	-1.21856	1.253052

ILMN_1787212	CDKN1A	-0.93407	-0.31897	1.253036
ILMN_3266606	FABP5L2	0.69217	-0.56083	1.252997
ILMN_2211065	TMEM91	1.17629	0.075826	1.2521159
ILMN_1742093	LOC644539	0.923648	0.327898	1.251546
ILMN_1731043	TRA2A	0.423452	-0.82779	1.251239
ILMN_1662852	IQCK	-0.1186	1.13262	1.251221
ILMN_3212045	LOC100133775	-0.29167	0.959331	1.251004
ILMN_3225983	LOC728927	0.01166	1.23916	1.2508204
ILMN_1698139	TCEA1	0.575357	0.675371	1.250728
ILMN_1764964	IFNGR2	0.780496	-0.47001	1.250502
ILMN_1796305	UPF2	1.1716	-0.07878	1.2503794
ILMN_3251521	PARG	0.887681	-0.3626	1.250285
ILMN_2081269	OR2A20P	0.64745	-0.60232	1.24977
ILMN_1671935	LOC646103	-0.44859	0.80104	1.249631
ILMN_1772686	FGD3	-0.13237	1.11716	1.249527
ILMN_3236594	SNORA29	-0.38366	0.865439	1.249098
ILMN_1713838	LOC642889	-0.16117	1.08783	1.249003
ILMN_1716524	RAB7A	0.387803	-0.86119	1.248988
ILMN_2131103	PPP1R3B	1.00155	0.24729	1.24884
ILMN_1684391	PLOD1	0.943144	-0.30403	1.247177
ILMN_1772876	ZNF395	0.768162	0.478805	1.246967
ILMN_1658053	DYNLRB1	-0.98722	-0.25939	1.246614
ILMN_3240117	AIDA	-0.23573	1.01063	1.246363
ILMN_3306482	LOC730107	0.457509	-0.78822	1.245726
ILMN_1777584	KARS	-0.15266	-1.09285	1.245508
ILMN_1804611	C12orf23	0.791875	-0.45302	1.244898
ILMN_1742167	TUBA1C	0.543766	-0.70095	1.244718
ILMN_1665280	SPCS1	0.048025	-1.19643	1.2444553
ILMN_1729208	NGFRAP1	0.471673	0.77263	1.244303
ILMN_3197896	LOC442319	-0.94378	-0.30041	1.244187
ILMN_2332105	WRNIP1	-0.08526	-1.15856	1.2438197
ILMN_1752028	PMS2L1	-0.07778	1.16521	1.2429898
ILMN_2329914	SPRY1	0.669094	-0.57341	1.242502
ILMN_1664516	CENPF	0.096088	1.14638	1.2424675
ILMN_2128358	TPP2	-0.77651	-0.46595	1.242465
ILMN_1708164	EIF3A	-0.37737	0.865066	1.242439
ILMN_1672128	ATF4	0.289132	0.953177	1.242309
ILMN_1812721	LOC728014	0.04349	-1.19868	1.2421697
ILMN_3211132	LOC645173	1.07993	-0.16214	1.242069
ILMN_2337241	RPS15A	0.743256	0.498314	1.24157
ILMN_3238375	CENPT	1.19818	-0.04325	1.2414251
ILMN_2147863	QSER1	-0.91417	0.327004	1.241177
ILMN_1664068	ERGIC1	-0.19873	-1.04212	1.240851
ILMN_1689953	CD81	-0.07627	-1.16387	1.240138
ILMN_1708604	C7orf28A	0.20932	1.03031	1.23963
ILMN_1761422	LOC440349	1.18799	-0.05157	1.239561
ILMN_1748432	ZNF525	0.113991	-1.12538	1.239371
ILMN_1783303	HSP90AB4P	-0.02359	1.21576	1.2393505
ILMN_3243333	GAGE12J	-0.42814	0.810904	1.239044
ILMN_3213640	LOC100132444	0.65119	-0.58738	1.238569
ILMN_1733538	RGS10	0.722487	0.516061	1.238548
ILMN_1808305	RTCD1	0.630023	-0.60845	1.238468

ILMN_1714364	PTK2	-0.47135	-0.76703	1.238382
ILMN_1751034	ITPRIPL2	1.13945	0.098709	1.2381587
ILMN_1813264	ZMAT1	0.904392	-0.33354	1.23793
ILMN_3259712	LOC100129952	0.383824	0.853575	1.237399
ILMN_3222425	LOC729852	1.08932	-0.14802	1.23734
ILMN_1654552	MRPS31	0.25221	-0.98478	1.236993
ILMN_2391861	GSTM1	0.60082	0.635232	1.236052
ILMN_2062468	IGFBP7	0.683258	0.550802	1.23406
ILMN_3243152	AKR7L	-0.04372	1.18977	1.2334925
ILMN_3225571	LOC728624	0.540742	0.692738	1.23348
ILMN_3239135	LOC100132391	-0.35764	0.8757	1.233339
ILMN_3241136	RNASEK	0.073091	-1.16012	1.2332109
ILMN_1741564	DCTN4	0.625169	-0.60795	1.233123
ILMN_1748767	LOC730740	0.011024	-1.2213	1.2323238
ILMN_1806266	RAP1GDS1	-0.27329	0.95874	1.232031
ILMN_2098433	FAM10A4	1.10842	-0.12336	1.231782
ILMN_1809467	VAMP5	0.243934	0.987031	1.230965
ILMN_1681845	PAPD4	1.07094	0.159842	1.230782
ILMN_1780508	SNORD108	-0.85871	-0.37198	1.230688
ILMN_3239895	LOC100134053	-0.23828	0.992203	1.230482
ILMN_3250321	PTP4A3	0.093382	-1.13668	1.230062
ILMN_1662848	TXNDC15	0.737129	0.492924	1.230053
ILMN_1711383	STK4	0.932401	-0.29761	1.230008
ILMN_2175831	MTFMT	0.223245	1.00675	1.229995
ILMN_2249552	GTF2A1	1.12083	-0.10879	1.229623
ILMN_1788024	PCID2	0.862855	0.366698	1.229553
ILMN_1662331	PDSS2	-0.16523	-1.06295	1.228181
ILMN_1664357	LOC652202	-0.23677	0.991357	1.228124
ILMN_1758311	NET1	0.875347	-0.35266	1.228004
ILMN_1757336	LRCH3	0.920625	-0.30676	1.227386
ILMN_1774617	NAB1	-0.35684	-0.86973	1.226573
ILMN_1684306	S100A4	-1.0473	-0.17883	1.226132
ILMN_3219455	LOC644745	-1.07706	-0.14907	1.226127
ILMN_2216157	GNA12	0.747235	0.478822	1.226057
ILMN_1736500	GNB2L1	1.15465	-0.07113	1.2257841
ILMN_2388142	CD99L2	1.19786	0.027682	1.2255415
ILMN_2404903	PDPN	0.535331	0.689815	1.225146
ILMN_2212878	ESM1	0.035692	-1.18916	1.2248515
ILMN_2278433	LOC285074	0.200169	1.02467	1.224839
ILMN_1707727	ANGPTL4	-0.31642	0.907974	1.224394
ILMN_3211857	LOC648822	0.156563	-1.06771	1.224273
ILMN_1678757	BCYRN1	0.726387	-0.49712	1.223507
ILMN_3274045	LOC100130229	0.934072	0.289304	1.223376
ILMN_1757186	GIMAP1	0.370933	-0.8523	1.223236
ILMN_2169966	TM4SF18	0.217118	-1.00584	1.222958
ILMN_1739335	LOC400948	-0.4649	-0.75748	1.222379
ILMN_1752932	MPZL2	-0.05462	-1.16769	1.2223141
ILMN_3244749	LOC729706	0.847576	-0.37465	1.222222
ILMN_2395926	MANBAL	0.603048	-0.61897	1.22202
ILMN_3268048	LOC100130452	-0.2822	0.939792	1.221995
ILMN_3273198	LOC100129865	1.11661	0.105088	1.221698
ILMN_1714759	CNIH4	0.6083	-0.61337	1.221674

ILMN_1739943	SBNO1	0.734423	-0.48688	1.221306
ILMN_2408415	RPL9	-0.2791	0.941591	1.220691
ILMN_2038776	TXN	-0.11343	1.10697	1.220399
ILMN_1677697	LOC441377	0.42003	0.800205	1.220235
ILMN_1770244	CBX1	0.374275	-0.84579	1.220069
ILMN_3242238	LOC647988	-0.3048	0.91524	1.220042
ILMN_2180239	DOPEY2	-0.03441	1.18519	1.2196025
ILMN_2213247	SPCS2	-0.25157	0.967533	1.219101
ILMN_1777347	LOC647841	0.185926	1.03293	1.218856
ILMN_1691156	MT1A	-1.08104	-0.13778	1.218822
ILMN_1789405	C22orf25	0.931685	0.286967	1.218652
ILMN_3229652	LOC728953	0.868743	0.349502	1.218245
ILMN_1719599	SYTL4	-0.62142	-0.59682	1.218241
ILMN_1742866	F2R	0.801026	-0.41705	1.218077
ILMN_2363426	MAX	0.23176	0.986173	1.217933
ILMN_1807106	LDHA	-0.253	0.964267	1.217267
ILMN_1787931	LOC389517	0.159746	-1.05731	1.217056
ILMN_1772369	PDHA1	0.997731	-0.21863	1.216358
ILMN_2369682	HNRPA2B1	-1.08438	0.131645	1.216025
ILMN_2359332	CDKN2C	0.548541	0.667358	1.215899
ILMN_1690802	TRMT112	0.951499	-0.26397	1.215465
ILMN_3231577	LOC100134648	0.817498	0.397582	1.21508
ILMN_1702301	DOCK10	0.120596	-1.09402	1.214616
ILMN_1775759	NRAS	0.969562	-0.24472	1.214285
ILMN_1676407	LOC646100	0.769833	0.444282	1.214115
ILMN_3185118	LOC100129053	0.717872	-0.49598	1.21385
ILMN_3297503	LOC730185	-0.40817	0.80556	1.213728
ILMN_1727689	TNFAIP2	0.816384	-0.39728	1.213665
ILMN_1729417	GNE	0.220779	-0.99269	1.213468
ILMN_1764297	ARF6	0.615621	-0.59784	1.213462
ILMN_2193980	ABCB6	0.707528	-0.5059	1.213423
ILMN_2358202	ARS2	0.396237	-0.81716	1.213396
ILMN_2393450	C14orf173	-0.16706	-1.04632	1.213381
ILMN_3234783	LOC100132287	0.924365	-0.28883	1.213196
ILMN_1810810	EEF1A1	-0.03895	1.17424	1.2131881
ILMN_1704294	CDH3	-0.46785	-0.74481	1.21266
ILMN_1696510	GPR89C	0.854022	0.358568	1.21259
ILMN_2140974	TPM4	0.396158	-0.81642	1.212578
ILMN_3304519	LOC728576	0.532308	-0.68023	1.212538
ILMN_1782954	HIP2	1.06441	0.147664	1.212074
ILMN_2338785	RPS14	1.14311	0.068861	1.2119711
ILMN_3230157	PACRGL	-0.10985	1.1021	1.211948
ILMN_3225304	LOC729484	-0.22817	0.983759	1.211932
ILMN_1679277	CMTM3	0.202477	1.00939	1.211867
ILMN_3202315	LOC643779	1.0341	-0.17693	1.211032
ILMN_1692116	LOC650321	0.061951	1.14825	1.2102006
ILMN_1699362	IK	-0.24321	0.966486	1.209698
ILMN_2356578	TH	-0.58681	-0.6222	1.209006
ILMN_1790881	HNMT	0.759091	0.449845	1.208936
ILMN_2046315	GABARAPL2	0.644187	0.564572	1.208759
ILMN_1657278	DVL1	0.435706	-0.77251	1.208218
ILMN_1726025	ASXL1	0.096565	1.11107	1.207635

ILMN_2054928	XPNPEP3	-1.17891	-0.02853	1.2074443
ILMN_2377210	PPM1B	0.891183	-0.31615	1.207335
ILMN_1766154	MRPL30	0.045638	-1.16164	1.2072781
ILMN_3224952	LOC729903	0.689544	0.517073	1.206617
ILMN_1791149	ARL6IP4	0.174308	-1.03168	1.205988
ILMN_1687538	ETS1	-0.01396	1.19186	1.2058222
ILMN_1736077	LIAS	0.8097	0.395948	1.205648
ILMN_1768940	COL15A1	-0.27011	-0.93546	1.205569
ILMN_1757052	SLC16A1	0.492521	-0.71285	1.205373
ILMN_1743621	C17orf69	-1.1509	0.053209	1.2041094
ILMN_3284114	LOC399748	-0.95492	0.249119	1.204034
ILMN_1815124	LOC645317	-0.89317	-0.31077	1.203939
ILMN_1785272	COL1A2	-0.55625	-0.64761	1.20386
ILMN_2053538	RHBDL2	-0.19388	1.00985	1.203727
ILMN_3192003	LOC100128533	0.226428	-0.97713	1.203554
ILMN_1718734	MLLT6	0.052959	1.15025	1.2032089
ILMN_1813344	C20orf7	0.038238	1.16476	1.2029978
ILMN_1728426	INPPL1	0.570582	-0.63241	1.202993
ILMN_1798620	PQLC1	0.422099	0.780808	1.202907
ILMN_1651262	HNRNPAB	0.222609	-0.97993	1.202534
ILMN_2362832	STAG3L1	-0.04173	1.16071	1.2024432
ILMN_3241139	SNORD57	0.467137	-0.73503	1.202168
ILMN_1717366	MDFIC	-0.0933	1.10847	1.2017666
ILMN_1689123	CCNK	0.770629	0.430897	1.201526
ILMN_1704531	PTGR1	-0.17661	1.02483	1.20144
ILMN_3202673	LOC100131196	-0.05431	1.14695	1.2012558
ILMN_3287687	LOC100133479	0.219028	-0.98222	1.201243
ILMN_1768505	IL13RA1	1.18298	-0.01818	1.2011588
ILMN_2066066	HLA-DRB6	-0.23199	0.968971	1.200964
ILMN_3213930	LOC441533	0.257144	-0.94331	1.200452
ILMN_2166831	RPS4X	-0.22396	0.976208	1.200164
ILMN_1706957	BMPR1A	-0.19141	-1.00845	1.19986
ILMN_2315979	LBH	-0.0321	-1.16763	1.1997348
ILMN_1655710	LOC642989	-0.32971	0.870019	1.199727
ILMN_3240155	RNU105A	-0.01675	-1.18274	1.1994872
ILMN_2120340	RUVBL2	-0.2129	0.986575	1.19947
ILMN_1669553	UBE2E3	0.273934	-0.92534	1.199276
ILMN_2072296	CKS2	0.15346	1.04566	1.19912
ILMN_3239625	LOC641367	0.484484	-0.71436	1.198839
ILMN_2244108	ITGB1	0.700525	0.49719	1.197715
ILMN_1748632	MUC4	0.433	-0.76441	1.197405
ILMN_1731194	STRAP	0.409287	-0.78662	1.195906
ILMN_2390821	THOC2	0.439366	-0.75648	1.195848
ILMN_2051519	RPL37A	0.486792	0.709011	1.195803
ILMN_3245616	CWC22	1.0485	-0.14689	1.195389
ILMN_2204332	FRYL	-0.08478	1.11059	1.1953723
ILMN_1660723	RDH13	-1.12146	0.073465	1.1949248
ILMN_3301585	LOC730993	0.346743	0.848119	1.194862
ILMN_3230315	LOC100131868	-0.08757	-1.10675	1.1943173
ILMN_1669302	GOLGA8F	1.00219	-0.19191	1.194099
ILMN_3246219	LOC729623	-0.84304	0.350922	1.193957
ILMN_2392189	CTDSPL	1.02749	-0.16623	1.193723

ILMN_2363027	RAD51	-0.08192	1.11047	1.1923876
ILMN_1772888	LOC645688	0.821158	0.371089	1.192247
ILMN_1658992	DPM1	0.386211	0.805845	1.192056
ILMN_1695404	LY6E	0.886329	0.305689	1.192018
ILMN_3282292	LOC646316	-0.24862	-0.94315	1.191777
ILMN_3251944	LOC100130598	0.439048	-0.75254	1.191583
ILMN_1770065	FLJ44290	0.701639	0.48977	1.191409
ILMN_1686957	SS18	1.01914	-0.1716	1.190735
ILMN_3213531	LOC653737	-0.246	0.944402	1.1904
ILMN_1796900	NUDCD3	-0.21751	0.972782	1.190292
ILMN_1778673	GOLGA7	0.213219	-0.97704	1.190262
ILMN_3279017	LOC389223	-0.16101	1.02916	1.190173
ILMN_3274586	LOC401817	0.68103	0.508131	1.189161
ILMN_1750496	ZBTB16	1.13555	-0.05341	1.1889613
ILMN_1761176	GRIPAP1	0.184732	-1.00405	1.188782
ILMN_1660608	GABRB3	0.333375	-0.85526	1.188637
ILMN_2158548	NACA2	1.02009	0.168535	1.188625
ILMN_1752008	OTUD1	0.784316	0.404067	1.188383
ILMN_1693490	SEC11A	-0.82728	-0.3604	1.187682
ILMN_1682588	LOC643461	0.833572	-0.35339	1.186957
ILMN_2210837	CDC26	0.92158	-0.26521	1.186789
ILMN_3238237	LOC100134667	0.101419	-1.08534	1.186759
ILMN_1781149	INMT	-0.50716	-0.67843	1.185588
ILMN_1673352	IFITM2	0.983408	-0.2021	1.185507
ILMN_1750674	SDSL	0.356371	0.82897	1.185341
ILMN_1726308	FAM10A4	-0.30249	0.882671	1.185165
ILMN_2371964	MRPS12	0.318415	-0.86625	1.18466
ILMN_3248487	LOC100132678	0.409526	-0.7751	1.18463
ILMN_2188204	ATG12	0.445414	-0.73902	1.184433
ILMN_1806432	NT5C	-0.0076	-1.17657	1.18416631
ILMN_3235597	LOC147727	0.673789	-0.51004	1.183827
ILMN_2409078	SNHG10	0.800867	-0.38252	1.183389
ILMN_1673369	SEPHS1	-0.55084	0.632439	1.183281
ILMN_2320480	LOC400464	-1.0882	-0.09492	1.1831222
ILMN_1724598	RABL2A	0.931924	0.250971	1.182895
ILMN_1735360	SDAD1	-0.37904	0.80333	1.182374
ILMN_1709851	TCEA1	-0.38772	-0.79452	1.182236
ILMN_3245897	LOC728190	-0.69439	0.486326	1.180718
ILMN_1732216	NARS	0.620873	-0.55974	1.180611
ILMN_1776104	NDUFS5	-1.12019	-0.06007	1.1802584
ILMN_3205910	LOC100131541	0.210355	0.969876	1.180231
ILMN_1735930	KLF2	0.566206	-0.61392	1.18013
ILMN_1777680	LOC653589	0.276082	-0.9039	1.179979
ILMN_1815012	EXOC7	-0.37936	0.800158	1.17952
ILMN_2121207	FAM125A	0.80405	-0.37535	1.1794
ILMN_1696806	CTNND1	0.270671	-0.90845	1.179119
ILMN_2391178	STAR	0.3995	-0.77959	1.179089
ILMN_1767377	LOC153561	0.764423	-0.41463	1.179048
ILMN_1724295	LOC643007	0.729569	-0.44905	1.178621
ILMN_3270846	LOC100129343	0.435706	0.74238	1.178086
ILMN_2313730	RHOC	0.649598	-0.52843	1.178027
ILMN_1668369	CDC37	0.85068	-0.32698	1.177655

ILMN_1738356	LOC402694	-0.57567	-0.60184	1.177504
ILMN_1761155	PABPC1	-0.62532	0.552086	1.177409
ILMN_3284424	LOC100132779	0.04532	-1.1317	1.1770198
ILMN_2325574	CASC4	0.57846	-0.59695	1.175413
ILMN_2141523	MRPL44	-0.35653	0.818668	1.175193
ILMN_1814022	NR1H3	0.585065	-0.59	1.175065
ILMN_1753716	EIF2B1	-0.41079	0.763446	1.17424
ILMN_3244548	LOC729383	1.07412	-0.0996	1.173717
ILMN_1680390	GCNT2	0.323348	-0.85026	1.17361
ILMN_1782286	LOC402571	0.556418	0.616829	1.173247
ILMN_3208305	LOC728179	0.442469	-0.73023	1.172701
ILMN_1653412	RAXL1	-0.06441	1.10782	1.1722316
ILMN_1787844	C19orf10	0.33043	0.84129	1.17172
ILMN_3230608	NBPF8	-0.82402	0.34765	1.171667
ILMN_1745214	LOC642393	0.225473	-0.94576	1.171231
ILMN_3253990	LOC100130233	0.693523	-0.47749	1.171009
ILMN_1772132	ATP5B	0.389872	-0.78113	1.171006
ILMN_1758386	KIAA1644	0.865958	-0.30452	1.170476
ILMN_3234831	LOC645296	-0.06927	1.10082	1.1700855
ILMN_1661170	NDUFB8	0.456315	-0.71375	1.170061
ILMN_2190850	PPID	-0.34562	0.822212	1.167835
ILMN_1656393	PPP2R3A	0.767526	0.400144	1.16767
ILMN_1743217	SNORD32A	0.349528	-0.81804	1.167569
ILMN_2131177	GUCY1A3	-0.62262	-0.54479	1.167402
ILMN_2402416	DNAJB6	1.13213	-0.03518	1.1673096
ILMN_1778488	WDR41	0.79466	-0.37247	1.167129
ILMN_3243441	EEF1AL7	-0.30655	0.860498	1.167051
ILMN_1794333	POU2F1	-0.02033	-1.14632	1.166648
ILMN_1774287	CFB	0.522441	0.644203	1.166644
ILMN_1663489	UBR2	-0.03624	-1.13012	1.1663627
ILMN_2230902	CTNNA1	0.951897	0.214401	1.166298
ILMN_2351611	UBQLN1	0.263987	-0.90223	1.166216
ILMN_3187283	LOC100128460	-0.34419	0.82168	1.165871
ILMN_3227049	PRAMEF3	-0.08279	1.08185	1.164643
ILMN_1723020	MAP3K1	0.533103	-0.63027	1.163377
ILMN_1758398	GUK1	1.03704	0.125562	1.162602
ILMN_1665717	EIF2S3	-0.43276	0.729592	1.162348
ILMN_3251876	LOC100129657	0.367352	-0.79482	1.162173
ILMN_1799871	LTK	0.914497	-0.24753	1.16203
ILMN_1772644	EML3	0.870891	0.291079	1.16197
ILMN_1815878	C11orf59	0.802458	0.359284	1.161742
ILMN_3237966	FBXL11	-0.92786	-0.2337	1.161558
ILMN_3237632	NCLN	-0.0559	1.10489	1.1607872
ILMN_1796912	ARHGEF7	0.245685	-0.91504	1.160725
ILMN_2077160	C9orf41	0.082958	-1.07773	1.1606879
ILMN_3248663	SCARNA7	0.079536	-1.0811	1.1606363
ILMN_2197365	RGS2	1.06036	-0.10018	1.160543
ILMN_1693372	LOC644305	0.338706	-0.82141	1.16012
ILMN_1685378	LOC728973	-0.0146	1.14466	1.1592588
ILMN_3239272	SNORD71	-0.37833	0.780636	1.158964
ILMN_3247816	LOC100133005	0.738482	-0.42042	1.158906
ILMN_1801633	C20orf52	0.107148	-1.05149	1.158638

ILMN_1791093	PPHLN1	0.03219	-1.12643	1.1586203
ILMN_1659771	LOC645683	0.536127	0.622001	1.158128
ILMN_1698478	SNAPC2	-0.56485	0.592781	1.157628
ILMN_2050109	GABARAP	0.447323	0.710053	1.157376
ILMN_1654691	FKBP3	0.823625	-0.33293	1.156559
ILMN_1802434	JPH2	-1.06544	0.091081	1.1565211
ILMN_1771537	LOC643920	0.288177	-0.8681	1.15628
ILMN_2386354	CSNK2A1	0.10492	-1.05136	1.15628
ILMN_1680465	ARL5B	0.770709	-0.38551	1.156214
ILMN_3230382	LOC727978	-0.65548	-0.49969	1.155175
ILMN_2241187	CRYM	-0.37252	-0.78213	1.154653
ILMN_1662364	AARS	0.001555	-1.15276	1.1543146
ILMN_3217771	LOC100133154	0.212423	-0.94178	1.154204
ILMN_1692169	NAT5	-0.42122	-0.73291	1.154131
ILMN_1673917	GTF2I	0.37531	-0.77861	1.153917
ILMN_3307648	CS	0.172796	-0.98078	1.153579
ILMN_2146761	FABP5	0.067282	1.08627	1.153552
ILMN_1783226	SSR2	1.05208	0.100608	1.152688
ILMN_1806023	JUN	0.021607	1.13108	1.152687
ILMN_2339779	ATP6V1E1	0.261361	-0.89118	1.152536
ILMN_1759861	LOC646653	0.119482	-1.03244	1.151922
ILMN_2310589	DIABLO	0.638379	-0.51303	1.151405
ILMN_3234997	MAP7D1	-0.00879	-1.14184	1.15062991
ILMN_1656165	USP9X	-0.03696	-1.11355	1.1505088
ILMN_3242603	GTF2H2B	0.538116	0.61232	1.150436
ILMN_1738263	PIGU	0.447642	-0.70276	1.150405
ILMN_1786034	GPRC5B	0.42003	-0.73024	1.150268
ILMN_2286334	SR140	0.513051	-0.63704	1.150089
ILMN_2063584	CLIC4	0.534456	0.615533	1.149989
ILMN_1791867	EPB41L4A	0.341093	-0.80829	1.149382
ILMN_3194067	LOC100129686	0.618644	-0.53051	1.149156
ILMN_1704760	BZW1	0.542573	-0.6065	1.149071
ILMN_1781952	MGST1	-0.83627	0.312773	1.149044
ILMN_3248891	GLB1L3	0.158632	0.989984	1.148616
ILMN_1775243	LOC646766	0.716758	0.431832	1.14859
ILMN_1693404	LOC646358	1.14415	-0.00418	1.148334
ILMN_3246433	RNY5	-1.01929	-0.12874	1.148031
ILMN_1738677	PRPF8	0.222688	0.924827	1.147515
ILMN_1697567	TPM3	0.614745	0.531949	1.146694
ILMN_1695588	HNRPC	0.025347	1.12125	1.146597
ILMN_1772492	MCART1	0.150754	0.995623	1.146377
ILMN_2112730	SLC9A4	0.328282	0.817922	1.146204
ILMN_1699574	NRP1	0.267409	-0.87854	1.145944
ILMN_1768394	ARPC5	0.917442	0.228419	1.145861
ILMN_3264073	LOC100130070	0.437059	0.708781	1.14584
ILMN_2334121	PRKDC	0.238683	-0.90504	1.143723
ILMN_1721022	SHC1	0.583951	-0.55945	1.143399
ILMN_2058782	IFI27	1.03792	-0.10421	1.142133
ILMN_2101920	HNRPH1	1.00147	0.140539	1.142009
ILMN_2276820	NSL1	-0.22236	0.918832	1.141196
ILMN_1810559	RHOQ	0.903437	-0.23761	1.141047
ILMN_3248591	LTBP2	-0.1373	1.00363	1.140931

ILMN_1699253	LOC729317	-0.82744	-0.31348	1.140917
ILMN_1790529	LUM	0.341491	-0.79937	1.140857
ILMN_3285742	LOC100131786	0.053038	1.08773	1.1407684
ILMN_2109156	RANBP1	0.99041	-0.14962	1.140033
ILMN_3234841	LOC728517	0.203909	0.936117	1.140026
ILMN_2148527	H19	0.533262	-0.60649	1.139754
ILMN_3237534	LOC100133517	0.262316	-0.87706	1.139378
ILMN_1782349	LOC648066	0.216561	-0.92246	1.139022
ILMN_2355559	PSAP	-0.85258	0.286322	1.138906
ILMN_1686362	CRYBA4	0.055267	1.08315	1.1384165
ILMN_2324002	CALD1	0.184652	-0.95371	1.138357
ILMN_2063051	C11orf55	-0.28475	0.853498	1.138248
ILMN_1745904	CCDC6	0.033463	-1.10476	1.1382234
ILMN_3204890	LOC345041	0.584269	0.553601	1.13787
ILMN_3299955	LOC728937	0.407616	0.730202	1.137818
ILMN_2228044	TBC1D23	0.29701	0.840698	1.137708
ILMN_1769201	ELF3	-0.57782	-0.55977	1.137586
ILMN_2366041	ITM2C	-0.22244	0.914631	1.137075
ILMN_2357577	PRKAA1	0.933993	0.202548	1.136541
ILMN_3301168	LOC730052	-0.6554	0.481118	1.136519
ILMN_1703565	GLTSCR2	0.877416	-0.2589	1.136317
ILMN_1694686	KIAA0194	-0.12123	1.01492	1.136147
ILMN_1779875	THY1	-0.32939	-0.80672	1.136111
ILMN_3249667	LOC100133678	0.58403	0.551424	1.135454
ILMN_1763198	STAT6	1.03123	-0.10395	1.135176
ILMN_3183544	LOC100130168	0.848372	0.286653	1.135025
ILMN_1745813	KIAA1279	0.68843	0.446105	1.134535
ILMN_1862018	ATXN7L3	0.263191	0.871297	1.134488
ILMN_2038775	TUBB2A	1.07022	0.063873	1.1340926
ILMN_1651228	RPS28	0.839937	0.293541	1.133478
ILMN_1727043	GLT25D1	0.909325	0.223887	1.133212
ILMN_1689712	LOC440927	-0.63837	-0.49448	1.132848
ILMN_3246585	LOC100132620	0.284755	-0.84782	1.132579
ILMN_1806692	HEXB	-0.6601	-0.4724	1.132499
ILMN_3201843	LOC644604	0.43682	0.69533	1.13215
ILMN_2414014	RBM10	0.359554	-0.77259	1.132143
ILMN_1788742	LOC441246	1.03593	-0.09591	1.1318405
ILMN_1665823	LOC389787	-0.0925	1.03928	1.1317809
ILMN_1798256	UPP1	0.609334	0.522061	1.131395
ILMN_1690295	LOC648343	0.429977	0.701407	1.131384
ILMN_1662905	NME1-NME2	-0.08192	-1.04932	1.1312376
ILMN_3251171	DNASE1L1	-0.11948	1.01114	1.130616
ILMN_3262895	LOC100128519	0.939961	-0.1905	1.130456
ILMN_3204241	LOC645515	-0.46411	0.666346	1.130454
ILMN_1663836	LOC648374	0.769117	0.36042	1.129537
ILMN_1746856	RAB21	-0.27226	0.857178	1.129435
ILMN_1671045	LOC439992	0.609175	-0.52001	1.129189
ILMN_2079004	MDH2	-0.2635	-0.86564	1.129139
ILMN_1721876	TIMP2	0.428544	-0.70043	1.12897
ILMN_1753340	LOC728226	0.526897	-0.60204	1.128933
ILMN_3247452	LOC100128731	-0.88561	0.243154	1.128761
ILMN_3246658	LOC728253	0.091711	1.03672	1.128431

ILMN_1799969	SNAPIN	0.833015	0.295375	1.12839
ILMN_1709683	RASSF2	0.768242	-0.35966	1.127899
ILMN_2331501	UBC	0.395919	0.731853	1.127772
ILMN_2311826	USP6NL	1.04977	-0.07777	1.1275375
ILMN_3260783	DKFZP779L1853	-0.4065	0.720473	1.12697
ILMN_1781360	MPHOSPH6	0.128235	-0.99837	1.126605
ILMN_1734021	CLSTN3	0.32677	-0.7995	1.126272
ILMN_1684647	ILKAP	0.218312	-0.9079	1.126216
ILMN_1663321	LOC644781	-0.28189	0.844308	1.126193
ILMN_1768870	CAPZA2	0.103488	-1.02266	1.126148
ILMN_3229083	LOC728467	0.362896	-0.76316	1.126059
ILMN_3242591	LOC100133049	0.812007	0.313903	1.12591
ILMN_1808245	RPESP	0.267806	0.857989	1.125795
ILMN_1800436	LOC643154	0.068635	-1.05715	1.1257848
ILMN_1738821	GOLGA2	-0.1073	-1.01793	1.125231
ILMN_3178890	LOC100130624	-0.70267	-0.42197	1.124642
ILMN_1684017	GYG2	-0.2	0.924336	1.12434
ILMN_3291472	LOC442727	0.614188	0.509504	1.123692
ILMN_1759585	CHEK2	0.624374	-0.49911	1.123488
ILMN_2184250	SERPINB9	-0.07913	1.04408	1.1232126
ILMN_2096116	HSP90B1	0.245924	-0.87683	1.122749
ILMN_1755758	RIF1	1.00386	0.118645	1.122505
ILMN_1695576	MRPL24	-0.74898	0.373344	1.122323
ILMN_1778457	IL18	-0.07603	1.04597	1.1219992
ILMN_1778803	ZFAND6	0.257144	-0.86419	1.121329
ILMN_2195236	PGRMC2	0.400534	-0.72079	1.121322
ILMN_3260180	FLJ25363	-0.03386	1.08704	1.1208955
ILMN_1659895	MSN	0.534217	-0.58595	1.120164
ILMN_3181916	LOC100129343	0.367114	0.752741	1.119855
ILMN_1674228	LOC651751	1.0886	-0.03113	1.1197262
ILMN_1679721	CYB561	0.263509	-0.85607	1.119576
ILMN_1751958	NSUN5	0.509709	0.608551	1.11826
ILMN_1713406	FAM39DP	0.891342	0.226899	1.118241
ILMN_1674434	LOC644361	0.232794	-0.88478	1.117578
ILMN_1665554	BRF2	0.379448	-0.73791	1.117355
ILMN_3241462	LOC730754	0.266056	0.851024	1.11708
ILMN_2273595	TEX11	0.278628	-0.8381	1.116723
ILMN_2313901	PAM	0.081844	-1.03459	1.1164339
ILMN_2117171	LMO4	-0.27059	0.845332	1.115918
ILMN_1764266	CKMT2	1.03529	-0.08063	1.1159157
ILMN_1778078	VPS16	-0.77333	0.342289	1.115618
ILMN_2246661	FAHD1	0.525544	-0.58957	1.115118
ILMN_1655871	LOC647384	-0.18417	0.930827	1.114996
ILMN_1790891	CKAP4	0.081764	1.0329	1.1146643
ILMN_2103343	HMGB3L1	0.386848	-0.72766	1.114506
ILMN_1705848	FAM104B	-0.21767	0.896831	1.1145
ILMN_1680781	C14orf135	1.07237	-0.04211	1.1144849
ILMN_1741971	KIAA1161	0.024472	-1.08989	1.1143617
ILMN_1690138	PHF10	0.637981	-0.47628	1.114265
ILMN_2262444	PDE7A	0.836755	0.277398	1.114153
ILMN_3200539	LOC100131672	-0.25364	0.860344	1.113981
ILMN_3247665	LOC100134560	0.66042	0.453247	1.113667

ILMN_1659095	LOC644029	0.112161	1.00116	1.113321
ILMN_2373556	SPAST	0.437059	0.67624	1.113299
ILMN_2186626	ZNF485	0.434671	0.67859	1.113261
ILMN_1751589	NUDCD2	0.201761	0.91114	1.112901
ILMN_1695261	LOC285176	0.092745	1.01991	1.1126554
ILMN_1805255	RAB39	-0.26382	-0.84733	1.111155
ILMN_3206132	LOC388076	0.869618	-0.24141	1.111027
ILMN_3276794	LOC440027	0.459816	0.65092	1.110736
ILMN_1764228	DAB2	1.05574	0.05489	1.1106299
ILMN_1671288	ODF2	-0.2351	-0.87544	1.110536
ILMN_2104356	COL1A2	0.250619	-0.85952	1.110141
ILMN_2361807	OS9	-0.44087	0.668997	1.109869
ILMN_1727740	SYNCRIP	-0.6075	0.502161	1.109659
ILMN_2408815	NAP1L1	0.687634	-0.422	1.109638
ILMN_2305225	NDRG4	0.561829	0.547027	1.108856
ILMN_2402131	TAF15	0.253722	-0.855	1.108718
ILMN_1782749	ZNF468	-0.04205	1.06576	1.1078115
ILMN_1667043	EIF4A3	0.452655	-0.6548	1.107451
ILMN_2244140	UNC13A	0.475174	-0.6321	1.107271
ILMN_2038774	EEF1A1	0.123302	0.983788	1.10709
ILMN_1661886	APEX1	0.221097	-0.88584	1.10694
ILMN_1753582	RPA2	0.905665	0.20127	1.106935
ILMN_1737084	TXLNA	0.105636	1.00104	1.106676
ILMN_1664314	LOC644200	-0.91569	0.190956	1.106641
ILMN_1653228	C3orf22	-0.69121	0.415269	1.106478
ILMN_1751243	OSBPL7	0.812166	0.294251	1.106417
ILMN_1759910	SERPINA5	0.422337	0.683596	1.105933
ILMN_1775744	MRPS16	-0.186	0.919856	1.105855
ILMN_1692754	TMEM49	0.343003	0.762848	1.105851
ILMN_1763882	LOC644634	0.561591	0.543346	1.104937
ILMN_1739562	LOC653166	-0.21711	-0.88745	1.104559
ILMN_1757427	XAGE1D	0.078502	1.02548	1.1039818
ILMN_1662174	ORMDL3	0.33799	-0.76599	1.103975
ILMN_1706839	TCERG1	0.554031	-0.54882	1.102846
ILMN_1723087	MDK	0.526499	0.576241	1.10274
ILMN_3289302	LOC100131699	0.704424	0.397895	1.102319
ILMN_2169439	ITGAV	0.156165	0.945775	1.10194
ILMN_3240354	CYCSP52	0.556577	-0.5451	1.101676
ILMN_1775268	HECW2	0.213856	-0.88774	1.101599
ILMN_2172221	SUMO1P3	0.537241	-0.56423	1.101471
ILMN_2405009	NBL1	0.858001	0.243006	1.101007
ILMN_2362681	CES2	0.87304	0.227916	1.100956
ILMN_2209180	P15RS	0.549814	0.551015	1.100829
ILMN_3254322	LOC100129758	0.230089	-0.87053	1.100618
ILMN_3250345	LOC283788	-0.54901	-0.5516	1.100614
ILMN_1742238	SET	1.06553	0.034605	1.1001349
ILMN_1746552	WDR17	-0.13714	0.962574	1.099715
ILMN_2218277	RPS2	0.964072	0.13529	1.099362
ILMN_3238845	FAM165B	0.176456	0.922892	1.099348
ILMN_1742981	TUBA1A	0.793705	0.305435	1.09914
ILMN_3251312	TMEM69	-0.75757	0.341094	1.098667
ILMN_1652638	LRRC58	-0.37125	0.7269	1.098146

ILMN_3305938	SGK1	0.59151	-0.50618	1.097689
ILMN_1655245	MDM1	-1.07969	0.017953	1.0976432
ILMN_1771447	ZNF833	-0.0325	-1.06451	1.0970127
ILMN_1767595	LOC647488	0.14407	0.952627	1.096697
ILMN_1675354	TncRNA	0.334489	-0.76186	1.096344
ILMN_1804277	SPRED1	1.01014	-0.0862	1.0963399
ILMN_1663121	LOC653564	0.545676	-0.55047	1.096142
ILMN_1343295	GAPDH	0.346425	-0.74932	1.095747
ILMN_3182762	LOC100129685	-0.23955	0.855675	1.095227
ILMN_3285276	LOC727827	0.773733	0.321424	1.095157
ILMN_1674782	LOC375295	-0.00871	-1.08607	1.09478034
ILMN_1672662	SLC20A1	0.181708	-0.91284	1.094547
ILMN_1757693	C17orf51	0.411436	0.682963	1.094399
ILMN_1798619	KCTD17	-0.19396	0.900275	1.094232
ILMN_1651886	CWF19L1	-0.45233	0.641588	1.093919
ILMN_1680070	MUC6	0.750258	0.343259	1.093517
ILMN_1711862	RNF7	0.029564	-1.06337	1.0929344
ILMN_3246855	LOC100133981	0.46849	0.623415	1.091905
ILMN_2379130	IRAK1	0.526658	-0.56512	1.091781
ILMN_1665294	LOC644992	-0.35987	-0.73074	1.090602
ILMN_3246850	LOC648729	0.032827	1.05755	1.0903769
ILMN_1705617	CFL1	0.861024	0.229189	1.090213
ILMN_1706434	LOC440359	0.610289	0.479852	1.090141
ILMN_1805175	TGFA	0.534536	-0.55554	1.090079
ILMN_1800837	CFDP1	0.486394	0.603651	1.090045
ILMN_2180929	SLC16A12	0.112718	0.977249	1.089967
ILMN_2209027	RPS26	-0.20884	-0.88097	1.089804
ILMN_1703477	ARHGEF2	0.268761	-0.82092	1.089678
ILMN_1696415	LOC652076	1.04444	-0.04522	1.0896556
ILMN_1787591	XPA	0.819089	0.269522	1.088611
ILMN_1724825	PCBP2	0.660977	-0.42737	1.088348
ILMN_1685077	SNX7	-0.69073	-0.39757	1.088297
ILMN_1768510	MAN2B2	0.735617	-0.35249	1.088108
ILMN_3219340	LOC100131713	0.480108	0.607983	1.088091
ILMN_2213199	KIAA1712	0.832537	-0.25532	1.087858
ILMN_3237729	LOC100133551	0.335205	0.752292	1.087497
ILMN_2146766	FABP5	0.904949	0.182435	1.087384
ILMN_1678842	THBS2	0.812883	-0.2743	1.087187
ILMN_3237396	AAGAB	-0.05924	1.02764	1.0868793
ILMN_2157441	HLA-DRA	0.219187	0.86651	1.085697
ILMN_1809879	LOC645116	1.02845	0.057174	1.0856241
ILMN_1716006	C18orf54	0.429101	0.656091	1.085192
ILMN_2175737	ZNF826	-0.04213	1.04269	1.0848211
ILMN_1667932	LOC652726	0.50581	-0.57888	1.084691
ILMN_3261747	LOC100128729	0.281652	-0.80302	1.084669
ILMN_1729509	C1orf43	0.240831	0.843716	1.084547
ILMN_3246360	LOC100130553	0.323746	0.760754	1.0845
ILMN_2268381	RRBP1	0.223882	0.860551	1.084433
ILMN_3215461	LOC100131989	0.382153	0.702135	1.084288
ILMN_1697211	UBE1DC1	0.477243	-0.60684	1.084084
ILMN_1746704	TRIM8	-0.10117	-0.98285	1.084022
ILMN_2297710	PLEKHB2	0.20558	0.878298	1.083878

ILMN_1712390	CUTA	0.540185	-0.54351	1.083698
ILMN_2413898	MCM10	0.374037	0.708999	1.083036
ILMN_1760121	RRAGC	-1.04722	-0.0358	1.083015
ILMN_3248069	LOC653881	-0.11645	0.966421	1.082873
ILMN_2214734	FRRS1	0.458941	-0.6238	1.082736
ILMN_1677440	ATP6AP2	0.500399	-0.58196	1.082357
ILMN_1764163	LOC644330	0.44796	0.634025	1.081985
ILMN_2168933	TNIP2	0.239797	0.842053	1.08185
ILMN_2335718	HNRNPAB	0.815986	0.26577	1.081756
ILMN_1680692	NUCKS1	0.58228	-0.49939	1.081666
ILMN_2363273	ZNF226	0.247277	-0.83396	1.081236
ILMN_1703244	MAP1LC3B	0.843837	0.236899	1.080736
ILMN_1741148	ALDOA	-0.23717	-0.84343	1.080592
ILMN_1806032	SLC25A16	1.05478	0.02577	1.0805502
ILMN_1787746	LOC440345	-0.43944	0.64102	1.08046
ILMN_3251620	JMY	0.828638	-0.25117	1.079811
ILMN_1695827	PPP1CA	0.363055	0.716254	1.079309
ILMN_1736479	FLJ32679	0.392338	-0.68679	1.079124
ILMN_1677262	LOC388621	0.678483	-0.40049	1.078971
ILMN_1722634	NUCB1	0.790443	-0.28844	1.078884
ILMN_1789457	GNL1	0.454246	-0.62413	1.078378
ILMN_1696027	LOC642333	-0.13452	0.943804	1.07832
ILMN_1701434	RAP1B	0.561988	0.516067	1.078055
ILMN_1660144	TCF20	0.000918	-1.07707	1.07798801
ILMN_1658437	SFXN4	0.626124	0.451673	1.077797
ILMN_2207533	RPS17	-0.12704	0.950449	1.077485
ILMN_1769135	DPP7	-0.56151	0.514712	1.076217
ILMN_1807807	SKA2	0.871846	-0.20406	1.075904
ILMN_2181241	LOC649946	0.430693	0.644961	1.075654
ILMN_1774997	BCL2L11	0.363772	-0.71184	1.075607
ILMN_1747305	GIMAP8	0.098236	0.977149	1.075385
ILMN_1801822	C18orf25	-0.15672	0.918548	1.075264
ILMN_3283015	LOC100133185	0.688589	-0.38656	1.075147
ILMN_1778734	MTMR10	-0.57861	-0.49624	1.074857
ILMN_1666536	VSIG2	0.623498	-0.45096	1.074456
ILMN_1772692	DICER1	0.792034	0.282197	1.074231
ILMN_1688034	COIL	0.703231	-0.37058	1.073812
ILMN_3237779	TMEM184C	0.948077	0.125426	1.073503
ILMN_2394132	PRO1853	0.173035	0.900003	1.073038
ILMN_1685534	PILRB	0.840892	0.231757	1.072649
ILMN_3201216	LOC441550	0.073489	0.998754	1.0722427
ILMN_2388507	AKT1	0.128633	0.943378	1.072011
ILMN_1750158	ACOX1	-0.47501	0.596828	1.071837
ILMN_1745005	GGCT	0.989853	-0.08173	1.0715852
ILMN_1761068	MGC52000	0.819805	0.251592	1.071397
ILMN_2234758	SRP14	0.746519	-0.32464	1.071163
ILMN_1739423	RN7SK	-0.64776	-0.42326	1.071026
ILMN_3298167	ZSWIM7	0.79657	-0.27445	1.071022
ILMN_1787949	RPS15A	0.126962	0.944053	1.071015
ILMN_1667858	ZNF493	-0.03099	1.03946	1.0704508
ILMN_1653469	RPL12	0.381676	0.688697	1.070373
ILMN_1758672	FAM107B	0.350165	-0.71939	1.06955

ILMN_2175712	NDUFA11	1.05128	0.017793	1.0690734
ILMN_1770667	HECA	0.494749	-0.57429	1.069038
ILMN_1660554	VWA1	0.194838	-0.87419	1.06903
ILMN_1781184	MYBPC3	0.398625	0.670323	1.068948
ILMN_2207505	LEP	0.305206	0.762884	1.06809
ILMN_1815668	GTF2IP1	1.02526	0.042736	1.0679955
ILMN_1708627	LOC653226	-0.22977	0.838053	1.067818
ILMN_1750636	RPS26L	0.174547	0.892873	1.06742
ILMN_3293503	LOC442270	0.87296	-0.19432	1.067278
ILMN_1754517	LOC123876	-0.22738	0.839657	1.067034
ILMN_1723467	ITGB1	-0.4092	-0.65783	1.067028
ILMN_1654320	LOC644250	-0.01802	1.04856	1.0665804
ILMN_1761833	SLC40A1	0.566126	0.500202	1.066328
ILMN_1685022	CAPN7	0.96256	0.103656	1.066216
ILMN_3225102	ZNF738	0.105875	0.960036	1.065911
ILMN_1665630	LOC643872	0.868345	-0.19716	1.065503
ILMN_1794379	DHX40	0.522441	-0.54171	1.064149
ILMN_2103295	TINP1	-0.15099	0.912666	1.063653
ILMN_1677165	SF3B1	0.289928	0.773482	1.06341
ILMN_1709142	PURA	0.283801	-0.77945	1.063248
ILMN_1652113	LOC641989	0.011979	-1.05118	1.0631587
ILMN_3201517	LOC389101	0.374832	0.688247	1.063079
ILMN_1802027	MGST2	0.435069	0.627433	1.062502
ILMN_1696160	TRQ1	-0.17597	0.886405	1.062378
ILMN_1791523	RPE	0.269716	0.792335	1.062051
ILMN_1652512	C2CD2	0.64371	-0.41787	1.061577
ILMN_1651699	EPS8	0.548143	-0.51336	1.0615
ILMN_1731783	ATP1A1	0.729172	-0.332	1.061171
ILMN_1673738	LOC646483	0.487906	0.572756	1.060662
ILMN_1653251	HIST1H1B	0.545119	0.515416	1.060535
ILMN_1679083	ZNF93	-0.0298	1.02972	1.0595172
ILMN_2051408	PNPT1	0.502706	-0.55632	1.05903
ILMN_1702759	TMX4	0.495386	-0.56347	1.058852
ILMN_1691436	BLVRA	-0.57726	-0.48123	1.058492
ILMN_3214532	LOC100131205	-0.31539	0.742723	1.058108
ILMN_1780924	SLC43A1	0.830707	-0.22724	1.057949
ILMN_1676984	DDIT3	0.105716	-0.9521	1.057811
ILMN_1678522	LOC644934	0.618087	-0.4395	1.057583
ILMN_3226082	LOC728620	-0.18823	-0.86923	1.057454
ILMN_1806304	ENTPD4	0.698218	-0.35911	1.057331
ILMN_2393763	ARPC4	1.02304	-0.03419	1.0572255
ILMN_2403946	FEZ2	0.868822	-0.18825	1.057068
ILMN_1793846	BZW1	0.400853	-0.65575	1.056602
ILMN_3257030	FTHL16	-0.28801	0.768428	1.05644
ILMN_2324421	TXNRD1	0.149879	0.906134	1.056013
ILMN_1681675	RBM16	-0.5931	-0.46289	1.055989
ILMN_2156953	ZFAND6	0.47772	-0.57824	1.055956
ILMN_1809488	SPCS2	-0.80691	-0.2489	1.055809
ILMN_3208056	LOC648294	0.59342	-0.46186	1.055278
ILMN_2150708	FLJ45966	-0.43506	0.620031	1.055094
ILMN_2174369	ELOVL5	0.698616	-0.35569	1.054309
ILMN_1658748	LOC644714	0.743176	-0.31108	1.054251

ILMN_1664167	RPF2	0.362419	0.69133	1.053749
ILMN_2394264	UBTF	0.363931	-0.68947	1.053398
ILMN_1771003	HAUS4	0.408571	-0.64373	1.052302
ILMN_1699709	RFC5	-0.57368	0.478225	1.051905
ILMN_3297934	MGC42157	-0.25658	0.795323	1.051904
ILMN_1788604	WBP2	-1.00067	-0.05046	1.0511344
ILMN_1740819	STARD7	0.179003	-0.87157	1.050573
ILMN_2404589	KCNRG	-0.91067	0.138669	1.049341
ILMN_3274904	LOC284230	0.995901	0.053215	1.0491163
ILMN_1664449	ALG5	-0.31451	0.734155	1.048665
ILMN_1774261	DOK4	0.445573	0.602728	1.048301
ILMN_1675669	IBTK	0.088767	-0.95911	1.0478738
ILMN_3271312	LOC100130591	0.185607	-0.8614	1.047011
ILMN_1786843	KCTD13	0.653736	0.393096	1.046832
ILMN_1780382	LOC653566	-1.0317	-0.01511	1.0468135
ILMN_1713934	LITAF	-0.62882	0.417837	1.046661
ILMN_1712888	HSPH1	0.660182	0.385616	1.045798
ILMN_1703108	UBE2L6	-0.85179	-0.19345	1.045242
ILMN_1777312	FLJ45994	0.785509	-0.25936	1.044871
ILMN_2288740	ZNF266	-0.02375	1.02045	1.0441997
ILMN_1768719	RDH11	0.947361	0.096691	1.0440518
ILMN_1773079	COL3A1	0.308946	-0.73501	1.043959
ILMN_1758390	WHAMML1	-0.56063	-0.48298	1.043613
ILMN_1737576	RASA1	-0.75089	0.292665	1.043554
ILMN_2284327	WDR23	0.974973	0.067388	1.0423606
ILMN_1737343	FNIP1	-0.86961	0.172582	1.042194
ILMN_1761560	PHF13	0.96622	0.075512	1.0417323
ILMN_3236284	LOC100131844	0.231123	-0.81056	1.041679
ILMN_1746917	LOC729843	0.813997	0.227632	1.041629
ILMN_1700674	LOC728481	0.922296	0.118491	1.040787
ILMN_2215640	TUBA3D	-0.21719	-0.82288	1.040068
ILMN_1761994	SMAD5	-0.13826	0.901772	1.040027
ILMN_1703949	KPNB1	-0.39607	0.643126	1.039198
ILMN_1676440	LOC440776	-0.56533	0.473704	1.039029
ILMN_1735499	DCBLD2	0.205182	0.833302	1.038484
ILMN_2099301	UNC84B	-0.00036	1.03726	1.03761516
ILMN_1718271	MTIF3	0.469127	-0.56837	1.037493
ILMN_3223204	LOC730081	0.509948	-0.52709	1.03704
ILMN_2310703	RPS26L	0.652383	0.384392	1.036775
ILMN_1802808	LOC654103	-0.97059	0.065962	1.0365525
ILMN_1701855	PPP1CC	0.227702	0.807975	1.035677
ILMN_3199798	LOC389342	0.068317	0.966705	1.0350215
ILMN_1796085	PPM1B	-0.70553	0.32943	1.034963
ILMN_1745223	CDC42EP4	0.56175	-0.47278	1.034531
ILMN_2312228	FUT6	-0.09982	0.934567	1.0343886
ILMN_3229227	LOC729629	-0.04659	-0.98732	1.0339032
ILMN_1712155	RPL6	0.967095	0.066281	1.033376
ILMN_1687301	VCAN	1.03115	0.002177	1.03332726
ILMN_1802205	RHOB	0.39767	0.635197	1.032867
ILMN_1707124	TFPI	0.681269	-0.3515	1.032772
ILMN_1701749	UQCRFS1	-0.43864	-0.59404	1.032686
ILMN_1752935	TMEM30B	1.00227	-0.03024	1.0325145

ILMN_2082130	C1orf123	0.611085	-0.42131	1.03239
ILMN_1808041	RPL10A	0.565728	0.466532	1.03226
ILMN_1788180	RAB13	-0.55021	0.481757	1.031963
ILMN_2233279	C19orf31	-0.3952	0.636635	1.031832
ILMN_2389376	WDR23	0.242343	0.78898	1.031323
ILMN_3237324	MMS19	-0.84837	0.182754	1.03112
ILMN_3239225	RNY3	0.515677	-0.5153	1.030981
ILMN_2216852	PGK1	0.827206	-0.20374	1.030944
ILMN_1794522	EIF5A	0.600024	-0.43048	1.030501
ILMN_1716758	PCDH1	0.331147	-0.69933	1.030472
ILMN_1676792	LOC645138	0.071738	0.958036	1.0297741
ILMN_1769277	LOC651436	-0.31356	0.715586	1.029141
ILMN_1750079	PURB	0.000759	-1.02806	1.02881887
ILMN_2087080	RPL5	0.637742	0.391031	1.028773
ILMN_1684258	LOC646195	0.132532	0.896204	1.028736
ILMN_1715113	HARS2	-0.74468	-0.28404	1.028721
ILMN_1782237	GPR109B	-0.20939	0.8181	1.027494
ILMN_2226183	FCRL1	0.14216	-0.88533	1.027488
ILMN_1743367	FZD4	0.041023	-0.98627	1.0272909
ILMN_1725121	XPO1	-0.24043	-0.78674	1.027165
ILMN_1730118	ZNF644	-0.00807	-1.01907	1.02714375
ILMN_1657147	LOC390282	0.591828	-0.43514	1.026968
ILMN_2344373	MVP	0.846303	-0.18057	1.026874
ILMN_1801700	LOC647655	-0.63241	0.39438	1.026785
ILMN_2355423	TPO	0.870971	-0.15501	1.025985
ILMN_1739876	RAB3GAP1	0.74445	-0.28146	1.025908
ILMN_2381753	G3BP2	0.548541	-0.47737	1.025908
ILMN_1659762	BTF3	0.877337	0.148534	1.025871
ILMN_1672940	ZNF562	0.387644	0.637895	1.025539
ILMN_1715179	SNRPA1	0.536445	-0.48772	1.024162
ILMN_1809347	LOC729466	-0.45695	0.567081	1.024027
ILMN_3204210	LOC284393	0.014207	1.00929	1.0234967
ILMN_1793287	LOC642755	0.313561	0.709408	1.022969
ILMN_1682293	LOC644207	-0.95675	0.065695	1.0224402
ILMN_2075051	PGS1	0.625249	0.397156	1.022405
ILMN_3293730	LOC100133169	0.342605	0.679016	1.021621
ILMN_1745860	FLJ40852	-0.31188	0.70971	1.021594
ILMN_1805807	SLC30A3	-0.15632	-0.86504	1.021357
ILMN_1670625	LOC730952	0.90654	-0.11472	1.021262
ILMN_3242498	LOC727880	-1.01372	0.007361	1.02108095
ILMN_1708646	LOC643149	0.871767	-0.1493	1.021071
ILMN_1660880	RNH1	0.782963	-0.23802	1.020981
ILMN_3281594	LOC646093	0.671958	0.348372	1.02033
ILMN_3245458	SNORA61	0.432523	-0.58759	1.020109
ILMN_2248912	BCAS4	0.57655	-0.44315	1.019697
ILMN_1760575	PTP4A1	-0.02948	-0.98999	1.0194689
ILMN_3284584	LOC390345	0.361623	0.657701	1.019324
ILMN_1760890	SEPN1	-0.62214	-0.39697	1.019107
ILMN_1734317	DPF2	0.14964	0.869433	1.019073
ILMN_1696692	BAZ1B	-0.10165	0.916862	1.018514
ILMN_2383344	RSU1	-0.20661	0.811904	1.018513
ILMN_1738632	PRKAR1A	0.939563	0.078767	1.0183299

ILMN_1729495	TRIM41	-0.0746	-0.94347	1.0180699
ILMN_3218138	LOC283523	-0.37093	-0.64684	1.017764
ILMN_1714990	DBT	-0.73235	-0.28423	1.016577
ILMN_2110206	GNPTAB	0.102215	0.914004	1.016219
ILMN_2231985	HSPA13	0.543368	-0.47238	1.015747
ILMN_1751941	LOC728772	0.834288	-0.18049	1.014777
ILMN_2141482	SERPINF1	-0.44342	-0.57107	1.014489
ILMN_1667125	RPS6KL1	-0.40562	0.608083	1.013704
ILMN_2057399	ZBTB8OS	0.447642	0.565708	1.01335
ILMN_1781795	ERH	0.526419	0.486574	1.012993
ILMN_3287814	LOC401648	-0.45313	0.559838	1.012964
ILMN_3194949	LOC100127922	0.82267	0.190269	1.012939
ILMN_1677906	LOC643287	0.997651	0.014456	1.012107
ILMN_1671337	SLC2A5	0.053198	-0.9586	1.0118016
ILMN_3240220	RNU1F1	0.236693	0.775068	1.011761
ILMN_2078404	ZNF765	0.056301	0.955266	1.0115669
ILMN_2343757	Sep-10	0.613711	0.397599	1.01131
ILMN_1652604	GGT1	0.204705	-0.80647	1.011172
ILMN_1742869	TBC1D20	0.628432	0.381877	1.010309
ILMN_1746516	RPS25	-0.0212	0.989037	1.0102403
ILMN_3275275	LOC727962	0.025267	0.98467	1.0099374
ILMN_1785379	ZNF8	0.519258	-0.49052	1.00978
ILMN_2271894	ZNF654	-0.25698	0.752789	1.009768
ILMN_3207020	LOC388556	0.2616	0.74812	1.00972
ILMN_1795474	LOC644131	0.172796	-0.83614	1.008933
ILMN_3225669	RPS10P3	0.330669	0.677939	1.008608
ILMN_1652631	GLIPR2	0.063463	0.945076	1.0085385
ILMN_1680738	C5orf13	-0.22778	-0.78013	1.007903
ILMN_1747303	DDX39	0.911155	-0.09645	1.007604
ILMN_1745154	PARD6B	0.735219	-0.27216	1.007375
ILMN_1795183	RNASE1	0.282766	-0.72418	1.006944
ILMN_1663577	S100PBP	0.130941	0.875966	1.006907
ILMN_3268152	LOC100128507	-0.94378	0.063103	1.0068784
ILMN_1729816	VDAC3	-0.08144	0.92446	1.0059002
ILMN_3235912	C7orf38	0.004976	1.0009	1.00587625
ILMN_1761159	ESYT1	0.961048	0.04473	1.0057777
ILMN_3236408	SCARNA5	0.498728	-0.50701	1.005742
ILMN_3291594	LOC100132979	0.840256	-0.16542	1.005679
ILMN_1653745	LOC727958	0.813599	-0.1919	1.005496
ILMN_1735680	TMEM30A	0.141444	-0.8633	1.004748
ILMN_1736757	GNPTAB	0.631774	-0.37244	1.004213
ILMN_1724250	GRN	0.447244	-0.55695	1.004189
ILMN_1748916	C18orf55	0.849088	0.15509	1.004178
ILMN_2377900	MAP1B	0.52992	-0.47411	1.004033
ILMN_3251155	PCBP2	0.282846	-0.72046	1.003308
ILMN_1778991	NFIB	0.322234	0.680921	1.003155
ILMN_3195954	LOC100128836	0.406264	-0.5966	1.002862
ILMN_1752229	TIMM50	0.274729	0.727799	1.002528
ILMN_1676411	PMS2L2	0.118925	0.883422	1.002347
ILMN_1688089	PEBP1	0.241786	-0.76032	1.002108
ILMN_1752589	TMEM183A	0.4256	-0.5758	1.001404
ILMN_2394750	EXOC4	0.20383	0.797365	1.001195

ILMN_3213761	LOC100131480	-0.53055	0.470633	1.001184
ILMN_1652982	LOC23117	0.499126	0.501699	1.000825
ILMN_2113362	ARL6IP1	-0.41056	-0.59022	1.000774
ILMN_1790197	FLJ20674	0.259292	0.740078	0.99937
ILMN_2320574	RGPD5	0.77413	-0.22523	0.999361
ILMN_2049766	NFE2L3	0.079298	-0.91995	0.9992436
ILMN_2406132	LILRB3	0.320802	-0.67841	0.999209
ILMN_2106658	BLZF1	-0.11948	0.879653	0.999129
ILMN_1783333	C16orf61	-0.50079	-0.49828	0.999071
ILMN_1680436	CSHL1	0.656601	-0.34179	0.998388
ILMN_1739570	LOC646561	-0.95659	0.040848	0.9974338
ILMN_1810334	COMMD7	0.058927	-0.93837	0.9972939
ILMN_1751086	ATL3	-0.71755	0.279475	0.997023
ILMN_1740589	ZNF440	0.166828	0.830017	0.996845
ILMN_3232057	LOC100131243	0.704663	0.29157	0.996233
ILMN_3199438	LOC440366	0.646177	0.349922	0.996099
ILMN_1651850	RPS16	0.752566	0.243201	0.995767
ILMN_1815882	HNRNPA1	0.108581	0.886973	0.995554
ILMN_1727458	HDAC1	-0.01205	-0.98331	0.9953624
ILMN_3271122	LOC100129742	0.789249	0.205761	0.99501
ILMN_1815118	ZNF554	0.562864	-0.43206	0.994927
ILMN_1751258	NDUFA4	0.042217	-0.95224	0.9944595
ILMN_3242288	LOC641768	0.498091	0.495486	0.993577
ILMN_1673171	LOC651268	0.757022	0.236532	0.993554
ILMN_1794017	SERTAD1	0.640766	0.352609	0.993375
ILMN_3241834	LOC100134504	0.838107	0.155007	0.993114
ILMN_1778104	ACADM	0.581325	-0.41167	0.992997
ILMN_1751726	LOC649049	0.258258	-0.7347	0.992958
ILMN_3285153	LOC645979	0.463716	0.52847	0.992186
ILMN_1807298	NR4A3	-0.1373	-0.85464	0.991942
ILMN_3235113	TOMM6	0.523714	0.467319	0.991033
ILMN_3263099	LOC100129195	0.638379	-0.35244	0.990823
ILMN_3261439	LOC100128098	0.111286	0.879393	0.990679
ILMN_1683597	LOC652071	0.323587	0.667062	0.990649
ILMN_1697735	EWSR1	0.231441	-0.7592	0.990645
ILMN_1693685	LOC205251	0.596125	-0.39429	0.990417
ILMN_1739798	C7orf30	-0.29167	-0.69825	0.989927
ILMN_1717982	BZW1	0.315312	-0.67408	0.989387
ILMN_2407464	FASTK	0.245924	-0.74296	0.988884
ILMN_1726647	LOC650646	0.209241	0.779459	0.9887
ILMN_1760347	SRGN	0.535172	0.453354	0.988526
ILMN_1750457	ZNF268	-0.13953	0.847959	0.987488
ILMN_3210304	LOC100131139	0.97187	0.015592	0.9874621
ILMN_3240740	EIF3L	0.319211	0.668027	0.987238
ILMN_3243593	LOC100008588	0.006647	-0.98046	0.98710428
ILMN_1700628	DDX24	0.650394	0.335365	0.985759
ILMN_3277297	LOC391825	0.486712	0.498397	0.985109
ILMN_1734267	ZNF587	0.694716	-0.29036	0.98508
ILMN_1738938	TIMM8B	0.303455	0.68159	0.985045
ILMN_1744068	ELP3	0.225553	-0.75907	0.984621
ILMN_1800164	PPFIA1	0.60448	-0.37889	0.983369
ILMN_1685580	CBLB	0.545835	0.436785	0.98262

ILMN_1665873	DST	0.202397	-0.78019	0.98259
ILMN_2364022	SLC16A3	0.236057	0.746392	0.982449
ILMN_3290385	LOC401640	0.483131	-0.49913	0.982257
ILMN_1685574	TSC22D2	0.24282	-0.73891	0.981727
ILMN_1766222	LARP4B	0.369899	-0.61156	0.981456
ILMN_1745385	HNRPM	0.47032	-0.51072	0.981038
ILMN_2191331	RPS4Y2	0.888716	0.091833	0.9805486
ILMN_1710333	LOC646236	0.969005	-0.01118	0.9801834
ILMN_2214197	TP53INP1	-0.91632	-0.06384	0.9801599
ILMN_2351309	TIAL1	0.586417	-0.39346	0.979875
ILMN_3247538	LOC100134396	0.626522	0.352994	0.979516
ILMN_1674661	CIRBP	0.793387	0.186062	0.979449
ILMN_1703697	LANCL1	0.704106	0.275291	0.979397
ILMN_2407168	S100A13	0.853067	0.126272	0.979339
ILMN_3211314	LOC401648	-0.08033	-0.89883	0.9791582
ILMN_2150258	ZFP36L2	0.85625	0.122189	0.978439
ILMN_1808591	LOC731049	-0.16595	0.812484	0.978431
ILMN_1661495	KIAA0754	0.720498	0.25793	0.978428
ILMN_2273700	ZNF706	0.410163	-0.5677	0.97786
ILMN_1693145	BUB3	0.258417	-0.71926	0.977678
ILMN_1704446	SLC6A10P	-0.47565	-0.50195	0.977594
ILMN_1765701	LOC399942	0.941871	0.035641	0.9775115
ILMN_2353754	TPM3	0.464909	-0.51258	0.977491
ILMN_1686514	LOC642267	0.519735	-0.45754	0.977279
ILMN_1700344	KRT126P	0.24099	0.736255	0.977245
ILMN_2227800	ARFGAP3	-0.01898	-0.95802	0.9769993
ILMN_1679185	LEF1	0.975371	-0.00108	0.97644834
ILMN_2368617	FKRP	-0.64737	-0.32906	0.976429
ILMN_1806576	LOC651137	-0.20136	-0.77477	0.97613
ILMN_2405023	PPP1CB	0.575914	0.400185	0.976099
ILMN_3288268	LOC100132499	0.714689	0.261137	0.975826
ILMN_2344204	PRR13	-0.7275	-0.24817	0.975662
ILMN_1695585	RPS26L	0.720021	0.255237	0.975258
ILMN_1691276	CXXC1	-0.36528	0.609829	0.975107
ILMN_1695000	TFIP11	0.249425	-0.72552	0.974947
ILMN_3200234	LOC100131094	0.384063	0.590319	0.974382
ILMN_3281563	LOC648771	0.91593	-0.05822	0.9741463
ILMN_1661363	CDC14B	0.871448	0.102005	0.973453
ILMN_2413808	CD53	0.342207	0.631226	0.973433
ILMN_3238183	BMS1P5	0.120994	0.851646	0.97264
ILMN_1699598	AP2M1	0.955796	-0.01678	0.9725723
ILMN_2064355	MCF2L2	0.12553	0.846746	0.972276
ILMN_1786972	SARS	-0.14582	0.825899	0.971714
ILMN_2262901	RUFY3	-0.09934	-0.87235	0.9716902
ILMN_1727183	ZNF763	-0.17295	0.798607	0.971556
ILMN_1674908	HOXB5	0.470241	0.501036	0.971277
ILMN_1814327	AGTR1	0.211071	-0.7599	0.970973
ILMN_3286375	LOC728179	0.575914	-0.39499	0.970904
ILMN_1724609	SLC2A8	0.086539	0.884144	0.9706827
ILMN_2194688	DKK2	-0.27289	0.69746	0.970353
ILMN_1685870	HYDIN	-0.4291	0.540819	0.969914
ILMN_1720422	G3BP2	0.416051	0.553767	0.969818

ILMN_1695598	LOC441775	0.090279	0.879416	0.9696947
ILMN_1678052	C19orf24	0.43117	0.538423	0.969593
ILMN_1775522	MAGED1	0.912429	0.057074	0.9695025
ILMN_2287296	SYF2	0.251573	0.717544	0.969117
ILMN_1748090	SLC2A11	0.720816	0.248195	0.969011
ILMN_2156250	SOCS3	0.675937	0.293062	0.968999
ILMN_1781099	ISY1	0.56549	0.403014	0.968504
ILMN_1807042	MARCKS	0.873995	0.094442	0.9684372
ILMN_1708105	EZH2	0.361703	-0.60656	0.96826
ILMN_1658015	MBNL2	-0.04651	0.921531	0.9680386
ILMN_2120575	HTATSF1	0.559999	-0.40784	0.967836
ILMN_1800225	PPARG	0.040307	-0.92743	0.9677377
ILMN_3235221	LOC644936	0.898424	0.069281	0.9677052
ILMN_1746919	LOC643882	-0.21942	0.74825	0.96767
ILMN_1684964	ZNF212	0.769356	-0.19799	0.967348
ILMN_2096759	PSMC6	0.728376	0.238799	0.967175
ILMN_1735231	ZSWIM4	-0.35525	0.610592	0.965843
ILMN_2220184	GFPT1	0.875029	-0.09051	0.9655368
ILMN_3240829	ZSCAN12L1	0.524748	-0.44066	0.965403
ILMN_2116556	LSM5	0.39019	0.575135	0.965325
ILMN_3294007	LOC442442	0.405786	-0.55934	0.965122
ILMN_1666642	LOC339047	-0.0103	-0.9546	0.9648998
ILMN_1701877	AXL	-0.07022	0.893938	0.9641584
ILMN_3292551	LOC286157	0.368387	-0.59533	0.963719
ILMN_3294126	LOC100131160	0.628512	0.334797	0.963309
ILMN_2094360	NR2F2	0.846622	-0.116	0.962622
ILMN_3248586	LOC100133144	0.057574	0.904731	0.9623051
ILMN_1657129	SKAP2	0.076751	-0.88555	0.9622982
ILMN_2053546	LOC653314	0.050731	0.911536	0.9622668
ILMN_3192446	LOC100128410	0.512096	-0.44951	0.96161
ILMN_1654497	ATAD1	0.775085	0.186429	0.961514
ILMN_1684771	PGRMC1	0.750895	0.210389	0.961284
ILMN_1728168	C20orf45	0.508436	-0.45257	0.961009
ILMN_1740217	HACE1	0.26709	-0.69301	0.960101
ILMN_3199655	LOC646784	0.80039	0.159457	0.959847
ILMN_3282436	LOC651198	0.748508	-0.21121	0.95972
ILMN_1732923	SIPA1L2	0.451143	-0.5084	0.959541
ILMN_2407824	ATP1B1	0.624374	-0.33436	0.958734
ILMN_1767661	LOC648907	-0.19865	0.759529	0.95818
ILMN_1719392	FH	0.354302	-0.60368	0.957977
ILMN_2397024	SPOP	0.069908	-0.88805	0.9579589
ILMN_2050434	MGC16703	0.05089	0.905873	0.956763
ILMN_1694514	ZDHHC11	0.891262	-0.06535	0.9566147
ILMN_1651538	NUMBL	-0.45448	0.502119	0.956598
ILMN_2308903	WFDC3	-0.60034	0.356071	0.956408
ILMN_1663616	DNAJC7	0.50955	0.446655	0.956205
ILMN_1746241	SDHC	-0.1926	0.763357	0.955961
ILMN_1776021	EIF4H	0.013491	-0.94221	0.9556976
ILMN_1776674	SAC3D1	0.497773	-0.45755	0.955323
ILMN_3235851	SCARNA9L	-0.04038	-0.91481	0.9551895
ILMN_2364971	PPP2R5C	0.529443	-0.42548	0.95492
ILMN_1811754	NDUFB10	0.887522	-0.06738	0.9548985

ILMN_3283090	LOC391169	0.485678	-0.46878	0.954453
ILMN_1811779	MGC24103	-0.58784	-0.36655	0.954395
ILMN_3204734	LOC100134648	-0.0076	-0.94673	0.95432431
ILMN_1712035	TMEM115	0.793705	-0.16035	0.954057
ILMN_1779530	COG6	-0.25658	-0.69728	0.953859
ILMN_2139943	RPS3A	0.463556	0.49019	0.953746
ILMN_1721605	SMYD2	-0.0337	-0.91974	0.9534343
ILMN_1691731	PARP14	-0.13682	-0.81623	0.953053
ILMN_1774828	VEZT	0.606151	-0.34682	0.952967
ILMN_1689901	ZNF658B	-0.78216	0.170351	0.952512
ILMN_1781290	RHOA	0.411834	-0.54057	0.952406
ILMN_2038772	RPS9	0.265897	0.68617	0.952067
ILMN_1665909	LASP1	0.515756	-0.43627	0.952021
ILMN_1705261	CAPN1	0.150038	0.801584	0.951622
ILMN_1703005	IFP38	0.579733	-0.37187	0.951598
ILMN_1690099	ITGB1BP1	0.132055	-0.81933	0.951386
ILMN_1681890	DYNLT3	0.057415	0.893577	0.950992
ILMN_1815719	PLCG2	-0.83627	0.114301	0.950572
ILMN_1675848	MYL12A	0.361623	0.588656	0.950279
ILMN_1704873	TCEB1	0.184255	-0.76597	0.950223
ILMN_1803742	CAPZA1	0.587293	0.362225	0.949518
ILMN_1743911	SLC25A39	0.607584	-0.34172	0.949306
ILMN_1697639	OGT	0.591987	0.356828	0.948815
ILMN_1776953	MYL9	-0.47843	0.469289	0.94772
ILMN_1786242	LOC649821	-0.7933	-0.15407	0.947369
ILMN_3179396	LOC100129410	0.588964	-0.35785	0.946817
ILMN_1709339	LOC727832	0.423292	-0.52349	0.946786
ILMN_1739541	NMI	0.568036	-0.37825	0.946286
ILMN_3225121	LOC728484	0.428624	0.516872	0.945496
ILMN_3239653	RAX2	0.616178	0.329105	0.945283
ILMN_1695193	ANGPTL3	0.498887	-0.44637	0.945253
ILMN_1811696	FDXR	0.510266	-0.43441	0.944673
ILMN_1693311	TMBIM6	-0.17494	0.769198	0.944137
ILMN_1670561	TCEAL8	-0.87486	-0.06904	0.9439033
ILMN_1728802	SDCCAG8	0.931685	0.012089	0.943774
ILMN_1726762	LOC440577	0.122983	-0.82078	0.943764
ILMN_2169152	SRGN	0.139694	0.803992	0.943686
ILMN_3248282	LOC100134539	-0.15926	0.783749	0.943012
ILMN_3272378	EZR	-0.09035	0.852297	0.9426494
ILMN_1672605	C7orf41	0.930094	0.012432	0.9425262
ILMN_1749109	PSAP	-0.932	-0.01044	0.9424427
ILMN_1805658	LTV1	0.802379	0.139782	0.942161
ILMN_2196328	POSTN	0.482495	-0.4595	0.941992
ILMN_2054442	ZNF146	-0.38565	0.555471	0.941119
ILMN_3234762	RN5S9	0.256984	-0.68357	0.940557
ILMN_1656537	SNRPN	-0.38684	-0.55354	0.940385
ILMN_1679450	LOC730746	0.742301	-0.19776	0.940057
ILMN_3233135	FAM178A	0.149879	-0.79011	0.93999
ILMN_3301324	LOC729301	-0.19547	0.744185	0.939654
ILMN_1810725	FAM129A	-0.38541	-0.55396	0.939373
ILMN_1736460	MDM1	0.229452	-0.70984	0.939292
ILMN_3265143	LOC100129502	-0.13531	0.803543	0.938854

ILMN_2373010	TMEM70	0.707209	-0.2316	0.938807
ILMN_1744118	ASTN2	0.741346	-0.1974	0.938741
ILMN_1812926	ANTXR2	0.433	0.505226	0.938226
ILMN_1679177	MARS2	0.178684	0.759233	0.937917
ILMN_2286574	EPHA3	-0.08184	-0.85601	0.9378511
ILMN_3197732	LOC441642	0.661614	0.276138	0.937752
ILMN_1703337	LOC441763	0.199214	-0.73853	0.937748
ILMN_1803483	KIAA2013	0.327009	-0.61061	0.93762
ILMN_1751097	CREB3L2	0.33982	-0.59752	0.937335
ILMN_2124769	YBX1	0.737925	-0.19908	0.937006
ILMN_2084489	ZNF595	0.125211	0.811093	0.936304
ILMN_2127936	ZNF100	0.009751	0.926543	0.93629364
ILMN_1753885	YTHDF1	0.898265	-0.03788	0.9361489
ILMN_1677314	MUC1	-0.10889	-0.82684	0.935733
ILMN_1702383	CNGB1	0.553792	0.381711	0.935503
ILMN_1745329	PRR14	-0.32486	-0.61004	0.934892
ILMN_2233050	PLA2G2D	0.824659	0.110183	0.934842
ILMN_3199974	LOC100131787	0.386848	0.547814	0.934662
ILMN_3296181	LOC100131894	-0.67927	0.255077	0.93435
ILMN_3201523	LOC100132652	0.677608	-0.25668	0.934284
ILMN_3301451	LOC729200	0.718429	0.21504	0.933469
ILMN_1739744	LOC339879	0.05829	0.874203	0.9324933
ILMN_3281327	LOC100133129	-0.18584	0.746398	0.932238
ILMN_2381758	G3BP2	0.872244	0.059967	0.9322111
ILMN_1811405	CRMP1	0.705141	0.226697	0.931838
ILMN_2403458	SMARCB1	0.347141	-0.584	0.931141
ILMN_1798163	UPF3B	0.263112	-0.66795	0.931063
ILMN_2115125	CTGF	0.631456	-0.29921	0.930667
ILMN_1652927	LOC641851	0.642039	-0.2883	0.930338
ILMN_3238233	HIST2H4B	-0.22666	0.702686	0.929347
ILMN_1713482	CWC15	0.267806	0.661518	0.929324
ILMN_3289352	LOC642828	-0.21847	0.710763	0.929228
ILMN_3291053	LOC346085	0.628432	0.299589	0.928021
ILMN_1732988	KIAA1666	-0.70442	0.223567	0.927985
ILMN_1704512	SERINC4	0.809381	-0.11854	0.92792
ILMN_1651254	LPP	0.210593	0.716846	0.927439
ILMN_1800020	GART	-0.36973	0.557655	0.927389
ILMN_1757350	CTNNB1	0.323587	0.603663	0.92725
ILMN_1681526	SLC5A1	-0.12107	-0.80587	0.926937
ILMN_2363165	TACC2	0.61753	-0.30919	0.926723
ILMN_1677530	LOC728944	0.555145	-0.37142	0.926566
ILMN_2300186	DYNLL1	0.570344	0.355319	0.925663
ILMN_1745962	FBXO7	-0.01309	0.912477	0.9255639
ILMN_1809931	NDRG1	0.106671	0.818407	0.925078
ILMN_1692545	LOC646849	0.523316	-0.40109	0.924407
ILMN_1687858	CALM2	0.539787	-0.38442	0.924203
ILMN_1679979	PLK3	0.695512	-0.22758	0.923092
ILMN_1801467	KCNK4	-0.06783	0.854326	0.9221592
ILMN_1723607	GPHN	0.652383	0.269356	0.921739
ILMN_1672755	LOC649548	-0.84359	-0.07813	0.9217205
ILMN_1794370	SEMA5B	-0.1369	0.784707	0.92161
ILMN_3297915	LOC728522	0.482654	0.438939	0.921593

ILMN_1687864	POP5	-0.11176	0.809726	0.921484
ILMN_3213092	LOC728162	-0.12688	-0.7946	0.921478
ILMN_1746408	MIDN	0.605197	-0.31569	0.920888
ILMN_1795910	LOC647531	0.874472	-0.04635	0.9208238
ILMN_2241825	LDB3	-0.29828	0.622368	0.920645
ILMN_2389590	PRKAR1A	0.373798	-0.5465	0.920293
ILMN_1784046	DLEU2	-0.02558	-0.89468	0.9202578
ILMN_1662587	PNPLA7	0.72758	-0.19232	0.919904
ILMN_2215965	CYP2B6	-0.46085	0.458691	0.919536
ILMN_1666477	LOC390876	0.72766	-0.1918	0.919463
ILMN_2226763	H2AFB2	-0.3009	0.618072	0.918975
ILMN_1671260	GPR177	-0.65309	0.265605	0.918699
ILMN_1685630	SIGLEC6	0.35661	-0.56098	0.917585
ILMN_2121437	NCL	0.850362	0.06721	0.9175721
ILMN_1741942	STX16	0.456554	0.460875	0.917429
ILMN_1670189	FAM21C	0.916726	-0.00036	0.91708733
ILMN_1661733	FOLR1	0.690976	-0.22576	0.916733
ILMN_1660376	LOC387841	0.887761	0.02864	0.9164011
ILMN_1674983	LOC387841	0.447164	0.468431	0.915595
ILMN_3290019	LOC646753	0.440719	-0.47485	0.915565
ILMN_1676765	LOC390354	0.340695	0.574792	0.915487
ILMN_1757072	LOC642489	0.765059	0.149072	0.914131
ILMN_3194248	LOC100129539	0.413982	0.49935	0.913332
ILMN_2198515	ARRDC3	0.368944	-0.54426	0.913208
ILMN_1725366	SLC27A5	0.027575	0.885576	0.913151
ILMN_1761490	ZNF75D	0.36783	0.544991	0.912821
ILMN_1778629	RTEL1	-0.12043	0.792347	0.912778
ILMN_1708351	LOC123688	-0.01683	0.895861	0.9126878
ILMN_1666380	STARD13	0.042296	-0.86992	0.9122151
ILMN_3287033	LOC646785	0.350801	0.561323	0.912124
ILMN_1702487	SGK	0.580529	-0.33137	0.911901
ILMN_3301502	LOC728683	0.02105	-0.8908	0.911852
ILMN_1815933	FTSJ2	0.39942	-0.51211	0.911529
ILMN_1676719	LOC644330	-0.28244	0.627989	0.910431
ILMN_1763730	APPL1	0.522998	-0.38729	0.91029
ILMN_3299025	LOC728991	-0.19857	-0.71055	0.909117
ILMN_3251662	HINT3	-0.56079	0.347905	0.908694
ILMN_1677607	SC5DL	0.880758	-0.0279	0.9086592
ILMN_3211630	LOC645405	-0.44811	0.460531	0.908644
ILMN_3276209	LOC727865	-0.33074	0.577738	0.908481
ILMN_2054362	SLC25A40	0.174626	-0.73376	0.908385
ILMN_1739674	PDE4DIP	-0.25801	0.650056	0.908069
ILMN_3231952	ARL17B	-0.60638	0.301293	0.907677
ILMN_1810058	RABL2B	0.512176	0.395416	0.907592
ILMN_1814998	FKSG30	-0.50079	0.40628	0.907071
ILMN_1703015	ZRANB2	0.870414	0.036481	0.9068947
ILMN_1706426	DSTN	0.833731	-0.07305	0.9067764
ILMN_1657803	CFH	0.726148	-0.18042	0.906572
ILMN_3242357	LOC100132418	-0.19268	-0.71252	0.905198
ILMN_1746457	GTF3C2	0.31738	0.587769	0.905149
ILMN_2250853	C1orf84	-0.15775	0.747285	0.905036
ILMN_1714158	PON2	0.007602	0.897376	0.90497816

ILMN_1777687	LOC283711	0.364408	-0.53977	0.904181
ILMN_1735474	R3HCC1	0.391304	-0.51278	0.904087
ILMN_3280019	LOC100132795	0.884896	-0.01884	0.9037375
ILMN_1687805	LOC651894	0.782167	-0.12035	0.902517
ILMN_3298215	LOC729340	-0.21194	0.689963	0.901903
ILMN_2316278	MAGED4B	-0.56357	-0.33807	0.901639
ILMN_1651799	SLC38A2	-0.42488	0.476319	0.901197
ILMN_1725642	SUMO3	-0.01444	0.885967	0.9004066
ILMN_1691942	CCNI	0.805005	0.095081	0.9000863
ILMN_2069128	EPB41L2	0.799037	0.100472	0.899509
ILMN_1682402	SNORD46	-0.19666	-0.70199	0.898656
ILMN_1811648	DCAKD	-0.58339	0.314648	0.898036
ILMN_2046856	LOC401019	0.701878	0.195767	0.897645
ILMN_2389151	UGP2	0.322553	-0.57496	0.897517
ILMN_1674953	LOC642403	0.78161	-0.11579	0.897403
ILMN_2093231	WBP1	0.134521	-0.76278	0.897299
ILMN_2270299	TP53BP2	0.040148	-0.85658	0.8967286
ILMN_2169261	TNNI2	0.816065	-0.08035	0.8964185
ILMN_1691053	LOC91561	0.402922	0.492758	0.89568
ILMN_2404085	CLIP1	0.419791	0.475183	0.894974
ILMN_2346562	ZNF273	0.225871	0.668696	0.894567
ILMN_1708805	NCOA3	0.777154	-0.11683	0.893983
ILMN_1690586	HNRPA1P4	0.329237	0.564359	0.893596
ILMN_1702501	RPS6KA2	0.133726	-0.75901	0.892735
ILMN_3264813	LOC100130592	-0.71063	-0.18203	0.892658
ILMN_1696021	KPNA6	0.398704	0.493421	0.892125
ILMN_1703074	CPD	0.420587	0.471195	0.891782
ILMN_1712687	PAK2	0.670526	-0.22038	0.890904
ILMN_1741219	RRAGB	0.43483	-0.45541	0.890238
ILMN_3240962	DDRGK1	-0.18027	0.709786	0.890056
ILMN_1657722	RPS3A	0.856807	-0.03319	0.8899924
ILMN_1805842	FHL1	0.79649	0.093501	0.8899913
ILMN_1683204	GMEB2	-0.51273	-0.37715	0.889876
ILMN_3209193	LOC644191	0.545198	-0.34436	0.889553
ILMN_1859744	LOC732138	0.293986	-0.59556	0.889543
ILMN_3230683	RUNDC2B	0.371729	0.517002	0.888731
ILMN_1804444	PDIA2	-0.84176	0.046836	0.8885983
ILMN_1707780	C6orf165	0.322951	-0.56549	0.888441
ILMN_1657283	ALKBH5	0.279106	0.609042	0.888148
ILMN_2146657	FAHD2B	0.287938	0.600207	0.888145
ILMN_1760842	LOC650843	0.415335	-0.4728	0.888134
ILMN_1789086	LOC642334	0.29327	0.594372	0.887642
ILMN_2350357	ZNF254	-0.19022	0.697236	0.887453
ILMN_1760922	GIT2	0.340218	-0.54718	0.887399
ILMN_3249167	SNORA63	-0.09759	-0.78925	0.8868466
ILMN_1656625	RPS24	0.395521	0.491284	0.886805
ILMN_1770055	LOC389634	0.26343	0.623261	0.886691
ILMN_3199929	LOC390183	-0.17398	0.712278	0.886262
ILMN_3202576	LOC100131643	-0.03203	0.854208	0.8862333
ILMN_1774074	RXRB	0.405866	-0.48027	0.886133
ILMN_2204826	SEPP1	0.035692	0.849888	0.8855795
ILMN_1667418	LOC283953	-0.36806	0.517369	0.885432

ILMN_2384807	LRRCC1	0.174785	0.710485	0.88527
ILMN_1795922	CCDC16	0.331067	-0.55382	0.884888
ILMN_1767139	NDUFA13	0.872881	-0.01181	0.8846926
ILMN_3247325	LOC100190939	0.551246	-0.33335	0.8846
ILMN_3240389	CPOX	0.549814	0.333774	0.883588
ILMN_1759219	ZMAT5	0.21497	-0.66861	0.883578
ILMN_1712634	TIA1	-0.2487	0.634706	0.883409
ILMN_1661537	LEPROT	0.133248	0.749629	0.882877
ILMN_3301818	ST20	0.403001	-0.4795	0.882505
ILMN_1695422	NCL	0.25221	-0.62999	0.8822
ILMN_2364376	ILK	0.352552	-0.52931	0.881857
ILMN_2165753	HLA-A29.1	0.480744	0.400836	0.88158
ILMN_3225591	RPL14L	-0.67609	-0.20532	0.881414
ILMN_3238707	SNORA8	-0.75391	0.127468	0.881381
ILMN_3298266	LOC728774	0.20749	-0.6729	0.880394
ILMN_1676091	LOC388275	0.45353	0.42666	0.88019
ILMN_2079786	NUAK1	0.227304	-0.65268	0.879982
ILMN_3206804	LOC255167	-0.25865	0.621291	0.879941
ILMN_2220283	HNRPA1L-2	0.414221	0.465697	0.879918
ILMN_2221564	LYAR	0.189506	-0.69024	0.879742
ILMN_3289171	LOC100131572	0.402285	0.477006	0.879291
ILMN_1807455	DHRS7	0.745723	0.132515	0.878238
ILMN_1754400	AYP1p1	0.602969	-0.27487	0.877835
ILMN_1653470	KLC2	-0.48623	-0.39085	0.877077
ILMN_3291673	LOC100131452	0.013252	0.863818	0.8770699
ILMN_1679577	SETD3	0.769913	0.107135	0.877048
ILMN_3246910	LOC100190986	-0.27958	-0.59723	0.876808
ILMN_1733116	UBXD7	0.287859	-0.58875	0.876611
ILMN_3226875	LOC728590	0.639413	-0.23717	0.876579
ILMN_3177271	LOC100129585	0.192291	0.684247	0.876538
ILMN_1685397	ITGA3	0.04707	0.828893	0.8759634
ILMN_2331163	CUL4A	-0.39631	0.479195	0.875506
ILMN_1651378	AUP1	0.603366	-0.27131	0.874676
ILMN_1799728	ADAM15	0.096326	0.778103	0.8744292
ILMN_2062112	ZC3H15	0.302182	0.571762	0.873944
ILMN_2412521	KIAA0101	-0.07309	0.800241	0.873326
ILMN_3187497	LOC100127891	-0.059	-0.81411	0.8731126
ILMN_1672320	LOC441907	0.413982	-0.45878	0.872763
ILMN_3273946	CRCP	0.046752	0.825414	0.8721662
ILMN_1746485	LOC645899	0.635037	0.237059	0.872096
ILMN_2151541	DNAJC10	0.81328	0.057926	0.8712056
ILMN_1694479	WDR18	-0.00895	-0.86209	0.87104006
ILMN_1653203	EFEMP2	0.356451	-0.51401	0.870459
ILMN_1773154	NFKBIA	-0.14709	0.723249	0.870337
ILMN_1718034	LOC441454	0.267966	0.60213	0.870096
ILMN_1675939	IFNGR1	0.506128	0.363929	0.870057
ILMN_2387636	ITGB4BP	0.834924	-0.03511	0.8700326
ILMN_1772092	LOC152586	0.33043	0.539038	0.869468
ILMN_2127416	GSR	-0.12934	0.738173	0.867516
ILMN_1774077	GBP2	-0.40292	0.464455	0.867371
ILMN_3236653	RNU1-5	0.053834	-0.81292	0.8667572
ILMN_1695917	C5orf15	0.220222	0.646416	0.866638

ILMN_3216125	LOC728126	0.234067	-0.63199	0.866052
ILMN_1719749	PTGES3	-0.17605	-0.68992	0.865975
ILMN_2058251	VIM	0.307195	-0.55826	0.865454
ILMN_1651826	BASP1	0.379527	-0.48589	0.865415
ILMN_1726289	C12orf35	0.123859	0.741498	0.865357
ILMN_3246292	LOC647099	0.46093	0.404262	0.865192
ILMN_1787567	TSC22D1	-0.41215	0.452791	0.864937
ILMN_3304691	LOC729366	0.514642	-0.35014	0.864784
ILMN_1752591	LEPROTL1	-0.16444	0.699768	0.864203
ILMN_3270641	HNRNPH3	0.268443	0.595538	0.863981
ILMN_3267760	LOC100128936	0.238683	0.625273	0.863956
ILMN_3216377	LOC100132911	0.310617	0.553252	0.863869
ILMN_1714880	LIMS3	0.354541	-0.50926	0.863803
ILMN_2306565	MTX2	0.732991	0.130207	0.863198
ILMN_2096654	COMMD4	0.519258	0.343739	0.862997
ILMN_1733453	LOC284988	0.652065	0.209992	0.862057
ILMN_1701774	AP3M1	0.296612	0.56543	0.862042
ILMN_1661178	NR4A1	-0.08566	-0.776	0.8616556
ILMN_1660982	FLJ00312	-0.07929	0.782323	0.8616147
ILMN_3236627	LOC100134773	-0.40538	0.455602	0.860985
ILMN_2090558	C2orf25	0.185766	0.67453	0.860296
ILMN_1796751	KIAA1274	-0.55936	0.300861	0.860218
ILMN_3289037	LOC653794	-0.58331	-0.27672	0.860032
ILMN_2395496	KLK7	0.145741	-0.71378	0.859522
ILMN_2378048	HNRPK	0.502786	-0.3564	0.859189
ILMN_1721669	IDH3B	-0.85083	0.008089	0.8589218
ILMN_2140455	FLJ42258	0.158712	-0.69994	0.858653
ILMN_1791396	DGCR6	0.688191	0.16999	0.858181
ILMN_2319996	RPL3	0.420428	0.437672	0.8581
ILMN_1737813	PRNPIP	0.653895	-0.20397	0.857864
ILMN_2269002	PDCD10	0.421621	0.436175	0.857796
ILMN_1768973	HIST2H2AC	0.220301	-0.63728	0.857576
ILMN_3241426	LOC100133773	-0.13515	0.722402	0.857554
ILMN_2056032	CD99	-0.31992	-0.53634	0.856256
ILMN_3176771	LOC100127915	0.576152	-0.27973	0.855882
ILMN_2214098	BIVM	0.360748	-0.49509	0.855838
ILMN_1789535	DHDDS	0.757738	0.097993	0.8557307
ILMN_2364174	CSNK1A1	-0.3843	0.471414	0.85571
ILMN_1716246	FRZB	0.416449	0.438779	0.855228
ILMN_3241783	LOC100132485	0.293588	0.56143	0.855018
ILMN_1691860	SPRY1	0.400216	-0.45414	0.854351
ILMN_2367191	PSMF1	0.347141	0.507149	0.85429
ILMN_1767236	LOC648103	0.485598	-0.36863	0.854226
ILMN_3201445	LOC728128	-0.03179	0.822295	0.8540816
ILMN_3229052	SNRNP35	-0.71651	-0.13752	0.854036
ILMN_1679060	LOC642559	0.212026	-0.64165	0.85368
ILMN_3305397	LOC728843	0.065452	0.788139	0.8535908
ILMN_3240247	NOP10	0.062508	0.791039	0.8535466
ILMN_1803846	EIF1	-0.03059	0.822632	0.853225
ILMN_2367440	WDR17	-0.40212	0.450933	0.853053
ILMN_1657612	LOC285900	0.354382	0.498664	0.853046
ILMN_1708416	ARL6IP1	-0.65516	-0.1971	0.852262

ILMN_2232430	NMD3	0.600263	0.251935	0.852198
ILMN_3244678	SNORA5B	0.372684	-0.47914	0.851821
ILMN_3228688	LOC730415	-0.10086	0.750262	0.851118
ILMN_2056167	OSTC	0.434592	-0.41642	0.85101
ILMN_3288154	LOC646346	0.166748	-0.68385	0.850593
ILMN_1661158	LOC284701	0.476209	0.374367	0.850576
ILMN_1693905	HAT1	-0.32533	0.524949	0.850281
ILMN_1795778	P4HA2	-0.04786	-0.8019	0.8497643
ILMN_3242688	GTF2H2D	0.536048	-0.31288	0.848928
ILMN_2412564	NCBP2	0.605674	-0.24322	0.848894
ILMN_1797585	MYO1B	0.751293	0.097573	0.8488655
ILMN_2127298	F2RL3	0.628909	0.219448	0.848357
ILMN_1741736	DDX28	0.52252	0.325809	0.848329
ILMN_2258383	BAX	0.273854	-0.57334	0.847197
ILMN_3256674	LOC100128485	0.081526	0.765647	0.8471726
ILMN_1671568	ECHDC2	0.738323	-0.10875	0.847074
ILMN_3285198	LOC389168	0.503502	-0.3433	0.846798
ILMN_1670369	LBH	0.088528	-0.75817	0.8467021
ILMN_1755115	RPL23	0.275684	0.570697	0.846381
ILMN_3241051	LOC644907	0.728853	0.117118	0.845971
ILMN_3268697	LOC100129882	0.653179	-0.19274	0.845923
ILMN_1657950	RPS26P10	-0.14208	-0.70341	0.845489
ILMN_1730082	RPUSD4	0.149561	0.695472	0.845033
ILMN_1755822	SYDE1	0.557532	-0.28732	0.844848
ILMN_3224907	LOC728672	0.583951	0.260687	0.844638
ILMN_3234116	LOC730382	-0.21998	-0.62466	0.844636
ILMN_2341793	CCT7	0.401091	-0.44305	0.844143
ILMN_1740749	RPL7A	0.277753	0.566294	0.844047
ILMN_1800033	LOC649214	0.036328	-0.80599	0.8423211
ILMN_3247242	LOC728687	-0.00258	0.839686	0.8422692
ILMN_3290577	LOC391833	0.208604	0.63341	0.842014
ILMN_1773847	DYNC1I2	0.549655	-0.29203	0.841688
ILMN_1731354	PARL	0.640766	0.200678	0.841444
ILMN_1699112	COPB1	-0.24942	0.591201	0.84062
ILMN_1657701	TMEM137	-0.35008	0.489373	0.839452
ILMN_1710682	SUOX	-0.57105	-0.26783	0.838884
ILMN_1707810	RPS5	-0.32143	0.517096	0.838529
ILMN_1694106	GPD1L	0.710472	0.127053	0.837525
ILMN_3237414	LOC653877	-0.32716	0.510019	0.837181
ILMN_2220320	RPL7L1	0.169613	0.667199	0.836812
ILMN_2355831	FHL2	0.53008	0.306411	0.836491
ILMN_1757914	C19orf56	-0.59015	-0.24578	0.835927
ILMN_3307276	LOC401233	0.421383	-0.41444	0.835824
ILMN_2397750	IVNS1ABP	0.14216	-0.69328	0.835443
ILMN_1760683	SFRS9	0.04715	-0.78806	0.835213
ILMN_2146418	CRIM1	0.14598	0.688845	0.834825
ILMN_2407124	MCM8	0.640368	0.194216	0.834584
ILMN_2380754	CCNG1	-0.09576	0.7379	0.8336634
ILMN_2396410	CS	0.704424	-0.12884	0.833265
ILMN_1658709	LAMB1	0.446607	-0.38656	0.833171
ILMN_2408796	C19orf28	0.819089	-0.01404	0.8331256
ILMN_1667443	LOC402110	0.636787	-0.19607	0.832856

ILMN_1757343	PABPC4	-0.43292	-0.39983	0.83274
ILMN_1770692	WDR12	-0.43276	0.399842	0.832598
ILMN_1711994	TCIRG1	0.312845	-0.51944	0.832285
ILMN_2219618	LOC90586	0.163963	0.668175	0.832138
ILMN_1777726	USP48	0.103488	-0.72855	0.832039
ILMN_1657962	LOC728946	0.545755	0.285765	0.83152
ILMN_1651680	LOC649150	0.284278	0.547092	0.83137
ILMN_3232282	LOC100130445	-0.12353	0.707141	0.830675
ILMN_1737146	TRAM1	0.065452	-0.76516	0.8306088
ILMN_1661174	LOC731640	0.797366	0.032652	0.8300182
ILMN_2201580	GSTM2	0.348812	0.480686	0.829498
ILMN_1701839	EXOSC1	0.747314	-0.08153	0.828845
ILMN_3249546	RASA4P	0.005454	0.823372	0.82882568
ILMN_3235118	LOC729760	0.590873	-0.23735	0.828222
ILMN_2347888	LARP4	0.094098	-0.73406	0.8281592
ILMN_3299424	LOC729173	-0.04786	0.780234	0.8280943
ILMN_3268083	LOC100128689	0.674266	0.153818	0.828084
ILMN_3280695	LOC100131403	-0.27974	0.547252	0.826989
ILMN_1775692	EIF4G3	0.778905	-0.04777	0.8266711
ILMN_3215712	LOC100131609	0.513926	0.312222	0.826148
ILMN_2180866	RPS26P11	0.266215	0.559809	0.826024
ILMN_2366710	UCRC	0.094019	-0.73179	0.8258066
ILMN_1696843	LOC613037	0.467853	0.357899	0.825752
ILMN_2167616	NACA	-0.2016	0.62393	0.825526
ILMN_3218292	LOC202781	0.497932	0.327537	0.825469
ILMN_1714809	RPIA	0.512176	-0.31253	0.824701
ILMN_1668345	OAF	0.760285	-0.06421	0.8244957
ILMN_1775466	RPP38	0.375469	-0.44838	0.823853
ILMN_1799819	MARS	0.605197	0.218413	0.82361
ILMN_1722900	EIF4A1	0.059006	0.764564	0.8235704
ILMN_3213573	LOC645715	0.16078	0.662784	0.823564
ILMN_1740384	CDC25B	-0.4217	0.401676	0.823371
ILMN_1771815	C20orf43	0.454962	0.368391	0.823353
ILMN_2151441	FAM103A1	0.764263	-0.05883	0.8230888
ILMN_1766408	CBFB	0.61387	0.209158	0.823028
ILMN_1777579	TIGD1	0.387564	-0.43527	0.822829
ILMN_2380418	BICD2	0.448119	-0.37422	0.822339
ILMN_1739497	GTF2H5	-0.19093	-0.63071	0.821639
ILMN_2380946	EIF4G2	-0.03974	-0.78171	0.8214579
ILMN_2062524	RBBP4	-0.0477	-0.77346	0.8211602
ILMN_3304802	MGC12538	0.512016	-0.30897	0.82099
ILMN_1715886	CNOT7	0.318097	-0.50283	0.820927
ILMN_1784753	PAIP2	0.111604	-0.70911	0.820711
ILMN_1681016	SPNS1	-0.1603	0.660222	0.820519
ILMN_2159322	SLK	0.278549	-0.54193	0.820482
ILMN_1753196	PTTG1	0.365681	-0.45476	0.820444
ILMN_1876924	WNK1	-0.5997	-0.22072	0.820421
ILMN_3234993	ROMO1	-0.79792	0.021906	0.8198231
ILMN_2363668	YIF1B	0.030042	0.789589	0.8196308
ILMN_1674415	LOC650463	0.685486	-0.13387	0.819357
ILMN_2158164	ZNF430	-0.07396	0.745268	0.8192283
ILMN_1674385	YWHAQ	0.284039	-0.53498	0.819019

ILMN_1705876	NAP1L1	0.339184	0.479515	0.818699
ILMN_1710177	LOC644670	-0.43721	0.381107	0.818319
ILMN_3239113	LOC144438	0.351915	0.465762	0.817677
ILMN_1656292	LOC388532	0.660898	0.156374	0.817272
ILMN_1742400	CEP350	0.60464	-0.21241	0.817053
ILMN_2331266	NUMB	0.694398	-0.12253	0.816925
ILMN_3265343	LOC100130633	-0.48432	-0.33233	0.81665
ILMN_3187680	ACCS	-0.00441	-0.8122	0.81661439
ILMN_1847822	KIAA0368	0.75742	0.058352	0.8157717
ILMN_1725441	NFATC2IP	-0.00672	0.808519	0.81524001
ILMN_1670456	RBM42	-0.20836	0.606841	0.8152
ILMN_2307903	VCAM1	-0.31005	0.504977	0.815031
ILMN_3213568	LOC402112	0.710631	0.104159	0.81479
ILMN_1751708	ITM2B	-0.32072	0.493208	0.813925
ILMN_3215006	LOC100131831	0.1907	0.622889	0.813589
ILMN_3301052	LOC728791	0.325895	0.487539	0.813434
ILMN_1782890	SLC25A3	0.251892	-0.56106	0.81295
ILMN_2333367	FKBP1A	0.804527	0.008112	0.81263947
ILMN_2136576	POLH	0.778268	0.034351	0.8126185
ILMN_2189870	FCF1	-0.00449	0.808022	0.81251496
ILMN_1678939	VNN2	0.555066	-0.25645	0.811511
ILMN_1787823	LOC727948	-0.25594	0.553495	0.809439
ILMN_1784871	FASN	0.029326	0.779246	0.8085716
ILMN_3243986	FAM72B	0.331942	-0.47622	0.808161
ILMN_1705064	NDEL1	0.680314	-0.12781	0.80812
ILMN_1652185	IL4R	0.493953	-0.31382	0.807774
ILMN_1758623	HIST1H2BD	0.667105	-0.14031	0.807414
ILMN_1663195	MCM7	0.613631	0.193749	0.80738
ILMN_3294028	LOC100131256	-0.36066	0.446602	0.807264
ILMN_3201115	LOC440043	0.558089	0.248947	0.807036
ILMN_3224384	LOC729652	-0.01738	0.78937	0.8067538
ILMN_2200917	SLC4A7	0.332738	-0.47378	0.806513
ILMN_1789830	CFLAR	0.781929	0.024149	0.8060778
ILMN_3292678	LOC100133273	-0.06927	0.736811	0.8060765
ILMN_2374425	CCNE1	0.072056	-0.73397	0.8060224
ILMN_3244803	RAD54L2	0.168897	-0.63678	0.805675
ILMN_3270378	LOC100127982	0.084788	-0.72088	0.8056701
ILMN_1690708	SPTBN1	0.205421	-0.60024	0.805658
ILMN_1699545	PCSK7	0.609414	0.195832	0.805246
ILMN_1765204	ST13	-0.16587	-0.63928	0.805142
ILMN_1688127	LOC341457	0.246879	0.55785	0.804729
ILMN_2382687	NR4A3	-0.50119	-0.30348	0.804672
ILMN_1727348	NMD3	0.786146	0.018409	0.8045548
ILMN_2322375	MAFF	-0.24616	0.557951	0.804108
ILMN_3275224	LOC442162	-0.11072	0.693265	0.803988
ILMN_2342240	MGAT2	-0.12855	0.675436	0.803984
ILMN_1751607	FOSB	0.588725	-0.21501	0.803736
ILMN_2181191	TPI1	0.311094	-0.49247	0.803563
ILMN_3283569	LOC389053	0.58037	-0.22307	0.803441
ILMN_1770433	PIK3CG	0.267011	-0.53642	0.803429
ILMN_1770803	BNIP2	0.007602	0.795465	0.80306716
ILMN_3188174	LOC100129067	-0.19706	0.605858	0.802918

ILMN_1739586	FEZ2	0.640448	0.161996	0.802444
ILMN_1704305	NIP7	-0.6523	0.150054	0.802352
ILMN_3264481	LOC100128578	0.741824	0.060304	0.8021284
ILMN_1706117	LOC391656	0.230964	0.570714	0.801678
ILMN_2250830	SPDYE1	-0.14892	-0.65132	0.800241
ILMN_2265783	FHL2	0.248868	-0.55129	0.800156
ILMN_2413278	RPL13	0.623817	0.175298	0.799115
ILMN_1809928	COL6A2	-0.24329	-0.55563	0.798924
ILMN_1664610	LOC402057	-0.45711	0.341644	0.798749
ILMN_3270310	LOC100129795	0.210036	-0.5886	0.79864
ILMN_3274914	LOC648927	0.40881	-0.38975	0.798558
ILMN_3304022	LOC729102	0.560397	0.237674	0.798071
ILMN_1766171	SNF8	-0.17239	-0.62558	0.797968
ILMN_3250585	KIAA0194	-0.2047	0.593224	0.797923
ILMN_1663054	LOC653199	0.1459	-0.65183	0.797732
ILMN_2083593	TTC32	0.455281	0.342206	0.797487
ILMN_3297577	LOC729841	-0.79282	-0.00463	0.79745773
ILMN_1771149	MRPL19	0.594932	0.202471	0.797403
ILMN_3304898	LOC92755	0.558646	-0.23834	0.796984
ILMN_1787680	SELS	-0.52474	-0.27114	0.79588
ILMN_3261837	LOC100130356	0.661694	0.131521	0.793215
ILMN_3223148	LOC728362	-0.12966	0.663346	0.793008
ILMN_1707475	UBE2E2	0.17025	0.621622	0.791872
ILMN_3176040	LOC100130446	0.51878	0.272942	0.791722
ILMN_1724555	LOC651959	0.549177	-0.24233	0.791509
ILMN_1713369	RPL13A	0.259531	0.531837	0.791368
ILMN_1770454	AGRN	0.158234	-0.63283	0.791059
ILMN_3240187	TMEM111	0.445334	-0.34557	0.790908
ILMN_1782095	C20orf55	-0.25579	0.534819	0.790604
ILMN_1810577	RPS4X	-0.12537	0.665045	0.79041
ILMN_1653794	C6orf160	0.634161	-0.15536	0.789518
ILMN_1661833	ANKRD12	0.629148	0.160008	0.789156
ILMN_1658835	CAV2	0.368785	-0.42013	0.788913
ILMN_2072091	HNRNPUL2	0.538196	-0.25048	0.788676
ILMN_2395373	GABBR1	0.219187	-0.56925	0.788441
ILMN_1787103	LOC643612	0.224757	-0.56277	0.787531
ILMN_1772798	ARPP19	0.600104	-0.1871	0.787202
ILMN_1685088	RPL36	0.487269	0.299896	0.787165
ILMN_2283001	DSE	0.007125	0.779636	0.78676072
ILMN_1657550	MVD	0.743176	-0.04332	0.786498
ILMN_1806601	GRSF1	0.118129	-0.66821	0.786334
ILMN_3281165	LOC100132763	0.288814	0.497297	0.786111
ILMN_1765212	LARP1B	0.112002	-0.67379	0.785787
ILMN_2378100	FBXL5	-0.02956	-0.75613	0.7856855
ILMN_1654250	MRPL21	-0.38382	0.401813	0.785631
ILMN_1653504	EDG1	0.310935	-0.47442	0.78535
ILMN_3274339	LOC391655	0.29502	-0.48987	0.784885
ILMN_3187770	LOC100130246	0.079298	0.70507	0.7843676
ILMN_1754121	CSK	0.76498	0.017462	0.782442
ILMN_1751051	C7orf25	0.026859	0.754138	0.7809969
ILMN_3266482	LOC100129158	0.290246	0.490444	0.78069
ILMN_1740319	IFI27L2	0.300909	-0.4796	0.780513

ILMN_1771735	LOC642646	-0.08534	0.694774	0.7801133
ILMN_3250927	EIF4E	0.002509	0.77579	0.77829948
ILMN_3248833	LOC644928	0.705141	0.072844	0.7779845
ILMN_3249707	LOC728649	-0.59827	0.1796	0.777868
ILMN_1708632	ZNF771	0.761001	0.016711	0.7777115
ILMN_3287157	LOC440575	-0.07579	0.700857	0.7766475
ILMN_1800008	ACAT1	-0.14542	-0.63108	0.776496
ILMN_1754179	AP1G2	0.720578	0.055831	0.7764088
ILMN_1687384	IFI6	0.396556	0.37945	0.776006
ILMN_1702171	LPCAT1	0.27823	-0.49762	0.775853
ILMN_1793954	LOC652322	-0.18027	0.594834	0.775104
ILMN_1726391	MRPL39	0.171284	0.60371	0.774994
ILMN_1727041	EWSR1	-0.11478	-0.65964	0.774418
ILMN_1765031	LOC339047	-0.36106	0.413239	0.774299
ILMN_1794108	MED9	0.306638	0.467567	0.774205
ILMN_1736911	TMOD1	0.36775	0.405907	0.773657
ILMN_1772929	ATP5J	-0.08582	0.6873	0.7731167
ILMN_1672405	TMED7	0.322553	-0.45043	0.772978
ILMN_2345319	PREPL	0.370456	-0.40227	0.772725
ILMN_2094587	USP8	0.526419	0.245959	0.772378
ILMN_3203976	LOC392008	0.646018	0.125491	0.771509
ILMN_1654920	HNRPH3	0.238683	0.532701	0.771384
ILMN_3300797	LOC729090	-0.00043	0.770517	0.77095173
ILMN_1813191	LOC653080	0.030758	-0.74	0.77076
ILMN_2397484	GJC1	0.483609	0.287144	0.770753
ILMN_1712755	LRRC41	0.652224	-0.11846	0.770686
ILMN_1776963	LMAN2	-0.02335	0.747197	0.7705488
ILMN_3289650	LOC402112	0.304012	0.465242	0.769254
ILMN_1723212	SFRS3	-0.18019	-0.58843	0.768617
ILMN_1733256	PSMD8	0.038317	-0.73005	0.7683664
ILMN_2383300	PTPRU	0.033066	0.735078	0.7681436
ILMN_2387452	MBD2	0.385893	-0.38223	0.768125
ILMN_2401769	PHF14	-0.45655	0.311394	0.767942
ILMN_1806906	SSR3	0.345072	0.422831	0.767903
ILMN_3198545	LOC100132773	0.381039	0.386078	0.767117
ILMN_1670800	LOC651745	0.634559	0.132095	0.766654
ILMN_2409451	NCKAP1	-0.30552	0.460981	0.766499
ILMN_3237507	LOC552889	0.388837	-0.37625	0.765087
ILMN_2383975	PRDX5	-0.25945	0.505534	0.764979
ILMN_1676793	LOC643922	0.600104	-0.1644	0.764503
ILMN_1798838	CTTN	0.529284	-0.23456	0.76384
ILMN_1747598	PPP1R11	0.124416	0.639316	0.763732
ILMN_3245678	RNU1A3	0.623658	-0.13911	0.762772
ILMN_1742230	BAZ1A	0.201045	-0.56171	0.762754
ILMN_1810120	SYT7	0.462044	-0.30042	0.762462
ILMN_2351230	RUFY3	0.525703	0.236633	0.762336
ILMN_1660730	SLC7A3	0.410004	-0.35149	0.761489
ILMN_1716601	LOC441032	0.275286	0.485935	0.761221
ILMN_1805701	LOC647650	-0.74317	0.017894	0.761065
ILMN_1796063	TRIM44	0.407776	-0.35312	0.7609
ILMN_1737394	LMNA	-0.49824	-0.26188	0.760121
ILMN_3225938	LOC729402	0.583075	0.17644	0.759515

ILMN_1661945	C14orf156	-0.69328	0.066157	0.7594348
ILMN_1738150	SUMO2	0.410959	-0.34828	0.759243
ILMN_1745976	TRAF4	0.024074	-0.73513	0.7592058
ILMN_3234436	LOC100132528	0.270034	0.488338	0.758372
ILMN_1773042	SGEF	0.14598	-0.61239	0.758366
ILMN_2063586	CLIC4	0.493317	0.264971	0.758288
ILMN_1738075	CMIP	0.656999	0.100957	0.757956
ILMN_1742813	TMEM167A	-0.05391	0.704035	0.7579429
ILMN_2112474	PAQR5	-0.27711	0.480763	0.757874
ILMN_1794707	ATHL1	-0.58745	0.169771	0.757217
ILMN_3211887	LOC727821	0.401648	0.355378	0.757026
ILMN_3284366	LOC644745	0.058052	0.698892	0.7569435
ILMN_2041101	ANXA2P1	0.322951	0.433607	0.756558
ILMN_1695872	LOC641992	-0.15799	0.598538	0.756528
ILMN_1679134	NSMCE4A	0.705857	-0.05046	0.7563155
ILMN_1736461	LOC642313	0.137784	0.618374	0.756158
ILMN_3212833	LOC728139	-0.06751	0.688484	0.7559989
ILMN_1807662	IGF2R	0.447323	-0.30856	0.755883
ILMN_1757317	LARS	-0.09592	-0.65989	0.7558135
ILMN_1726589	CD248	-0.72853	0.02709	0.7556187
ILMN_1657547	CCDC34	0.115424	-0.63968	0.755101
ILMN_1737426	PCMTD1	0.568752	0.186269	0.755021
ILMN_1742135	SPAG16	0.668616	-0.08619	0.75481
ILMN_1666332	LOC654000	0.161815	-0.59239	0.7542
ILMN_3241870	FRMD8	-0.04468	-0.70866	0.7533344
ILMN_3288587	LOC100131785	0.448517	-0.30474	0.75326
ILMN_1661451	LOC645520	0.021925	0.731196	0.7531213
ILMN_3238735	LOC100132346	0.205421	-0.54752	0.75294
ILMN_1764082	MBOAT1	0.654771	-0.09802	0.7527881
ILMN_1679470	LOC149224	-0.63479	0.117396	0.752188
ILMN_2321416	DIAPH1	-0.06521	0.686975	0.7521823
ILMN_2357377	TERF1	-0.27512	0.477035	0.752156
ILMN_1683447	LOC440737	0.414858	0.336632	0.75149
ILMN_2395913	ARHGAP11A	0.051129	0.6992	0.7503287
ILMN_1709348	ALDH1A1	0.541379	0.208105	0.749484
ILMN_1786893	RBM5	-0.26939	-0.4795	0.748896
ILMN_3248676	CBWD3	0.672993	-0.07552	0.7485178
ILMN_1749447	RPS9	0.397033	0.351325	0.748358
ILMN_1705330	LOC648863	0.040466	-0.70754	0.7480099
ILMN_1676548	BZW2	0.309423	-0.43751	0.746936
ILMN_1766713	HSPD1	-0.49427	0.252551	0.746817
ILMN_1669523	FOS	0.490691	-0.25517	0.745858
ILMN_1728083	EIF4EBP2	0.258019	0.486769	0.744788
ILMN_3288218	LOC644315	0.252528	0.491995	0.744523
ILMN_1795937	VIL2	-0.22539	0.518984	0.744372
ILMN_2115011	FGD2	0.018822	0.724935	0.743757
ILMN_1737462	OXR1	0.636628	0.106715	0.743343
ILMN_2328280	ACTL6A	0.3399	0.403316	0.743216
ILMN_1656199	LOC650759	0.419632	0.323329	0.742961
ILMN_2227790	GRIN2C	-0.6484	-0.09416	0.7425579
ILMN_3213854	LOC344328	-0.45559	0.286736	0.742329
ILMN_1743347	AKT2	0.106273	-0.63562	0.741897

ILMN_1733295	ARS2	0.55793	-0.18306	0.740987
ILMN_2179579	SNHG3	-0.19754	0.543186	0.740723
ILMN_1803018	KIFC2	-0.16706	0.573419	0.74048
ILMN_1748546	Sep-02	0.56167	-0.17863	0.740301
ILMN_3290100	LOC645157	0.629148	0.110727	0.739875
ILMN_1720988	ABI2	-0.61466	-0.12456	0.739223
ILMN_1673604	YIPF3	-0.04372	-0.69542	0.7391425
ILMN_2119224	KIFAP3	0.687555	-0.04964	0.7371969
ILMN_3296816	LOC338739	0.117891	0.619279	0.73717
ILMN_3251332	POM121L10P	0.540026	-0.19706	0.737089
ILMN_1704713	CSNK1G1	0.581882	-0.15487	0.736754
ILMN_1657697	SAR1A	0.618167	-0.11848	0.736647
ILMN_1731984	HMGN3	-0.67148	-0.06509	0.7365674
ILMN_2152131	ACTB	-0.5245	0.211702	0.736206
ILMN_2110252	NPM3	0.267409	0.468331	0.73574
ILMN_2089977	FKBP9L	0.528329	-0.20727	0.735594
ILMN_1674050	COL8A2	-0.34141	0.393445	0.734851
ILMN_2285490	PPIL3	0.518541	-0.21577	0.73431
ILMN_1744147	CEBPZ	-0.01468	0.719426	0.7341043
ILMN_2115490	NBPF20	-0.66766	0.06608	0.7337359
ILMN_1715926	LOC642210	-0.51702	0.216034	0.733058
ILMN_1772845	SNRNP35	0.532546	-0.19999	0.732533
ILMN_2408663	RHCE	-0.51838	0.213744	0.73212
ILMN_1688676	LOC651361	-0.51909	-0.21297	0.732063
ILMN_2092516	HSP90B3P	-0.7014	-0.03038	0.7317756
ILMN_1742577	GTPBP4	0.552837	-0.17874	0.731574
ILMN_1672356	ANKRD13D	0.099987	-0.63064	0.7306216
ILMN_3239103	LOC340357	-0.03752	0.692987	0.7305028
ILMN_2323633	TPD52L2	0.185209	-0.54509	0.730302
ILMN_3216336	LOC285741	-0.21337	0.516777	0.73015
ILMN_3281599	LOC642741	0.33059	0.399434	0.730024
ILMN_1789614	TPT1	0.268045	0.46191	0.729955
ILMN_1678300	MGC40489	-0.15743	0.572478	0.729911
ILMN_3262031	BRD7P2	0.351199	0.378332	0.729531
ILMN_1687080	LOC402251	0.133646	0.595716	0.729362
ILMN_3225586	LOC728732	0.050333	0.678832	0.7291649
ILMN_2359935	CDC2L1	0.526976	0.202022	0.728998
ILMN_2297511	PODXL	0.219028	0.509848	0.728876
ILMN_1750763	LOC643699	0.639174	0.089661	0.7288349
ILMN_1689518	PECAM1	0.240433	-0.48814	0.728576
ILMN_1699829	CTGF	0.659863	-0.0684	0.7282632
ILMN_2371984	SMAD5	0.328919	0.398984	0.727903
ILMN_1730631	C2orf44	0.356769	0.371095	0.727864
ILMN_2407082	Sep-15	-0.66495	0.062174	0.7271243
ILMN_1754410	C12orf72	0.25778	-0.46907	0.726845
ILMN_2401978	STAT3	0.56923	0.157564	0.726794
ILMN_1789171	EEF2K	-0.50795	0.218247	0.726199
ILMN_3186853	LOC100130233	-0.112	0.613989	0.725985
ILMN_1714499	LOC646920	0.066168	-0.65921	0.725379
ILMN_1774027	ZNF37A	-0.26955	0.455312	0.724863
ILMN_1746252	LOC391811	0.330112	0.394445	0.724557
ILMN_1651642	GPC2	0.466739	0.257746	0.724485

ILMN_1695998	YKT6	0.09012	0.633605	0.7237245
ILMN_1721703	PNN	0.319529	-0.40397	0.723502
ILMN_3192316	LOC100129237	0.09935	0.62393	0.72328
ILMN_2296697	MAP3K3	-0.35724	0.365894	0.723135
ILMN_1810838	MTDH	0.665911	0.057103	0.7230141
ILMN_1794643	ZGPAT	0.324622	-0.39637	0.720991
ILMN_2381537	CRCP	-0.17303	0.54753	0.720559
ILMN_2398107	ASNS	0.417643	-0.30213	0.719777
ILMN_2053490	FAM53B	-0.12139	0.598029	0.719415
ILMN_2050761	EIF4E	-0.05025	0.669151	0.7193985
ILMN_3300471	LOC730255	-0.1482	0.571176	0.719378
ILMN_1676393	ATP5G1	0.670844	-0.0483	0.7191485
ILMN_1669878	GUSB	0.310776	-0.40789	0.718666
ILMN_2344850	VPS26A	0.416051	-0.30256	0.718611
ILMN_2214790	LAMB1	0.306718	-0.4118	0.71852
ILMN_1783852	CD164	0.109217	-0.60907	0.718289
ILMN_1686116	THBS1	0.209638	-0.50777	0.717409
ILMN_1770020	PPIL3	0.598831	-0.11813	0.716962
ILMN_2208802	NPIP	-0.39289	-0.32389	0.716776
ILMN_1761801	LOC147804	0.610767	0.105697	0.716464
ILMN_2357386	FKTN	0.531512	0.184784	0.716296
ILMN_1714449	OR5D18	-0.34714	-0.36911	0.716243
ILMN_1805990	BAK1	0.658431	-0.05778	0.7162153
ILMN_1660358	LOC388210	0.560715	0.15522	0.715935
ILMN_1666733	IL8	0.131418	0.584396	0.715814
ILMN_3255735	LOC100128031	0.293429	-0.42214	0.715569
ILMN_3199780	LOC401076	0.640368	-0.0752	0.7155674
ILMN_3296994	LOC728823	0.329316	0.386155	0.715471
ILMN_3207060	LOC647276	0.467535	-0.24775	0.715287
ILMN_1657423	SPG21	0.631774	-0.08315	0.7149205
ILMN_3293367	LOC391370	0.591828	0.12297	0.714798
ILMN_1659027	SLC2A1	-0.38804	-0.32674	0.714775
ILMN_1763685	LOC339047	0.320563	0.393712	0.714275
ILMN_1750205	ZNF409	-0.60097	0.113206	0.714179
ILMN_3225634	LOC728782	-0.05765	0.656376	0.7140238
ILMN_1795822	DIS3L	-0.60774	0.105573	0.71331
ILMN_2367215	PRCP	0.355098	0.357035	0.712133
ILMN_1755677	FAM158A	0.645858	-0.06609	0.7119504
ILMN_1655626	FAM193B	0.525783	-0.18528	0.711065
ILMN_1783636	COX6A1	-0.03258	-0.67833	0.7109123
ILMN_1759023	WFS1	0.456872	-0.25388	0.710749
ILMN_2257749	Mar-08	0.085663	-0.62508	0.7107424
ILMN_1656807	RPL27	0.406025	-0.30455	0.710579
ILMN_1783026	RNPC3	-0.57766	-0.13261	0.710269
ILMN_1690320	TTC23	0.70904	-0.00092	0.70996348
ILMN_3251132	TMOD2	0.193724	-0.5157	0.709425
ILMN_2238302	DST	0.658988	0.049765	0.7087534
ILMN_1771376	PEA15	0.524509	-0.18392	0.708424
ILMN_2308582	CYB5R3	0.330351	-0.37784	0.708193
ILMN_2348093	ATP5J	0.1198	0.588242	0.708042
ILMN_1674874	MFSD10	0.176616	-0.53035	0.706962
ILMN_1731374	CPE	-0.50907	-0.19753	0.706597

ILMN_3237404	LOC100132585	0.060916	0.645026	0.7059422
ILMN_2143795	MGC4677	0.251414	-0.45408	0.705496
ILMN_3207490	LOC441896	-0.38358	0.32127	0.70485
ILMN_1690494	RPL6	0.258417	0.446324	0.704741
ILMN_1776956	LOC400221	-0.16253	-0.54208	0.704606
ILMN_1710756	ENO1	0.602332	0.101608	0.70394
ILMN_1739641	MTMR3	-0.30051	-0.4033	0.703804
ILMN_1698940	LOC653232	-0.18115	-0.52246	0.703609
ILMN_3226214	LOC728755	-0.14144	0.562164	0.703602
ILMN_1701596	RPS20	0.534854	0.168002	0.702856
ILMN_3245057	ASAP1	0.148606	-0.55411	0.702711
ILMN_1777811	URG4	0.254359	-0.44827	0.702624
ILMN_2222688	TMSB4X	-0.15719	0.54521	0.702404
ILMN_1801852	SEC61B	0.567479	0.134734	0.702213
ILMN_1781819	PAPSS1	0.602412	0.099579	0.7019906
ILMN_1659564	SEC61A1	0.384381	-0.31743	0.701811
ILMN_2295511	LUC7L	-0.15592	0.545873	0.701794
ILMN_1779752	LOC23117	0.654055	-0.04717	0.7012234
ILMN_1782621	RPS12	0.668616	0.032084	0.7007001
ILMN_1669233	LOC401677	0.578699	-0.12192	0.700617
ILMN_1805737	PFKP	0.054869	-0.64515	0.7000196
ILMN_3271204	LOC100127893	-0.08232	0.617598	0.6999135
ILMN_1658266	LOC146053	0.387484	-0.31189	0.699376
ILMN_1785732	TNFAIP6	0.686282	-0.01263	0.6989102
ILMN_3285959	LOC645515	-0.38748	0.3114	0.698879
ILMN_1694057	EIF3K	-0.36568	0.332691	0.698367
ILMN_3298037	LOC728453	-0.34451	0.353846	0.698355
ILMN_2082810	BRD7	0.630262	0.067216	0.697478
ILMN_1773968	SERBP1	0.384779	-0.31269	0.697469
ILMN_1661917	LOC644039	-0.01134	0.686004	0.6973403
ILMN_1701696	LOC644863	0.432921	0.263812	0.696733
ILMN_1715864	NHS	0.463795	0.232739	0.696534
ILMN_2188264	CYR61	0.454167	0.241207	0.695374
ILMN_2389347	NR3C1	-0.01969	0.674731	0.6944224
ILMN_1729197	LOC400879	0.563341	-0.13102	0.69436
ILMN_1711729	LOC442454	0.260804	-0.43345	0.694252
ILMN_3247645	LOC550643	0.286984	-0.4072	0.694182
ILMN_1700276	C14orf100	0.331067	-0.36231	0.693381
ILMN_1809484	TMOD3	-0.67466	0.018586	0.6932444
ILMN_3285346	LOC643387	0.059882	-0.63221	0.6920907
ILMN_1780502	LOC641798	0.295577	-0.39594	0.691514
ILMN_1694323	GLE1	-0.05653	-0.63483	0.6913648
ILMN_1786215	ZNF3	0.630023	-0.06133	0.6913519
ILMN_3191789	LOC100130332	0.63249	-0.05876	0.6912507
ILMN_3211463	LOC644037	-0.03521	0.655944	0.6911522
ILMN_2381397	HSPD1	0.16078	0.530002	0.690782
ILMN_1793836	LOC389442	-0.14542	-0.54442	0.689841
ILMN_3231881	LOC728026	0.149402	0.539819	0.689221
ILMN_3192377	LOC100129060	0.617053	0.072151	0.6892042
ILMN_2098437	FAM10A4	-0.40061	0.28857	0.689178
ILMN_3248237	LOC100133402	-0.22539	0.46333	0.688718
ILMN_1755303	ZNF217	0.601457	0.087241	0.6886977

ILMN_1680887	LOC643550	0.471116	0.217182	0.688298
ILMN_3219008	LOC728073	-0.01635	0.671838	0.6881874
ILMN_1761865	LOC641704	0.576948	-0.11121	0.688161
ILMN_1680453	ITM2C	-0.18075	0.507244	0.687992
ILMN_3307877	C21orf58	0.217596	-0.47037	0.687969
ILMN_1720442	NCBP2	0.560795	-0.12669	0.687488
ILMN_3287952	LOC100133800	-0.08709	0.600396	0.6874859
ILMN_3295946	LOC100131713	-0.13682	-0.55016	0.686981
ILMN_2400663	AKAP9	0.356053	-0.33071	0.686762
ILMN_3279575	LOC100132521	0.268125	0.417985	0.68611
ILMN_3215367	LOC100131360	0.419552	-0.26635	0.685903
ILMN_1782538	VIM	-0.40355	0.282097	0.685649
ILMN_2189933	RPL36AL	0.103249	0.582052	0.685301
ILMN_3240685	INO80D	-0.27974	-0.40517	0.684905
ILMN_3240838	SLC25A6	0.507481	0.177127	0.684608
ILMN_3246279	LOC100134229	0.503184	-0.1811	0.684288
ILMN_1760779	ENSA	0.418677	-0.26489	0.683566
ILMN_1793476	PRKCDBP	-0.24815	0.434086	0.682232
ILMN_1752285	RPL4	0.294702	0.387368	0.68207
ILMN_3304003	LOC729208	0.411038	-0.27028	0.681318
ILMN_1763404	LOC653226	0.420109	0.261119	0.681228
ILMN_2171596	LOC441257	0.209638	-0.47113	0.680768
ILMN_1746968	PHF1	-0.30417	-0.37641	0.680575
ILMN_3211746	LOC100131166	0.21306	0.466899	0.679959
ILMN_1661500	B4GALT4	0.221017	0.458845	0.679862
ILMN_2101375	CCDC77	-0.09011	0.588437	0.6785507
ILMN_3236120	LOC100132913	0.25969	0.418725	0.678415
ILMN_1671928	PROS1	0.531353	0.146966	0.678319
ILMN_1767086	MGC57359	0.529125	0.148948	0.678073
ILMN_1770824	ARHGAP4	0.138818	0.539174	0.677992
ILMN_1669032	PPIC	0.57106	0.106662	0.677722
ILMN_1771599	PLOD2	0.015003	-0.66224	0.6772425
ILMN_1697469	SFRS6	-0.12632	-0.5509	0.677217
ILMN_1671386	OR2T2	-0.41899	-0.25809	0.67708
ILMN_1815500	ITPR3	-0.20486	0.472159	0.677017
ILMN_2179837	BANF1	0.58785	-0.08914	0.6769909
ILMN_3241164	KDSR	0.017628	0.658547	0.6761754
ILMN_1761260	COBLL1	0.332102	-0.34378	0.675883
ILMN_3287093	LOC646819	0.034339	0.641067	0.6754057
ILMN_1756674	ATP5EP2	-0.4057	-0.26947	0.67517
ILMN_3181439	LOC100130138	0.204466	0.469632	0.674098
ILMN_1737141	LOC654163	-0.53708	-0.13478	0.671858
ILMN_2355004	HMGN3	-0.46642	0.20498	0.671395
ILMN_1807833	HM13	0.408571	-0.26266	0.671229
ILMN_2170353	PTPLB	-0.21226	-0.45849	0.670743
ILMN_1689244	PARD6G	-0.10531	0.564933	0.670245
ILMN_1714861	CD68	0.468888	-0.20134	0.670224
ILMN_2242921	IKZF2	-0.19698	0.473118	0.670098
ILMN_2285506	ACSL4	0.003544	-0.66611	0.66965393
ILMN_1673885	HOXB6	0.094337	0.575206	0.6695429
ILMN_2157932	FKSG44	0.126962	0.542399	0.669361
ILMN_1806508	B4GALT2	0.131498	-0.53744	0.668939

ILMN_3238150	LOC730329	-0.11422	0.554412	0.668636
ILMN_1802557	HEBP1	0.051049	-0.61656	0.6676071
ILMN_1714433	MARCKSL1	0.298362	0.368574	0.666936
ILMN_1743583	MADD	0.443981	-0.22223	0.666211
ILMN_1665736	LOC648024	-0.42997	-0.23562	0.665587
ILMN_3247269	LOC400804	-0.04874	0.616421	0.6651566
ILMN_1743103	SH3PXD2A	-0.45313	-0.21198	0.665101
ILMN_1777340	DDX6	0.385654	-0.27904	0.664698
ILMN_1695899	LOC648659	0.520769	0.143462	0.664231
ILMN_1713706	ZNF786	0.620077	0.044073	0.6641498
ILMN_1770697	LOC644063	0.51504	-0.14894	0.663983
ILMN_1815552	NTAN1	0.353188	-0.30995	0.663133
ILMN_2334243	CREB1	0.325736	0.336472	0.662208
ILMN_1775939	SF3B2	-0.14916	-0.5123	0.661455
ILMN_1758085	PPIL5	0.333693	0.327223	0.660916
ILMN_1662438	SOD1	0.637265	0.023285	0.6605498
ILMN_1780861	LOC653506	-0.17215	0.487959	0.660113
ILMN_1688246	LOC642852	0.514006	-0.14539	0.659398
ILMN_1803825	CXCL12	0.006011	-0.65261	0.6586237
ILMN_1662359	HIST1H4K	0.143513	-0.51423	0.65774
ILMN_1747759	WSB1	0.183698	-0.47391	0.65761
ILMN_1731184	MELK	0.00434	0.653186	0.65752566
ILMN_1795839	SCCPDH	0.133328	0.523978	0.657306
ILMN_2355776	GIT2	0.227065	-0.42974	0.656809
ILMN_1781626	C1S	0.138659	-0.51786	0.656514
ILMN_1806405	CDKN2AIPNL	0.159746	0.496616	0.656362
ILMN_1811063	LOC649447	0.33807	0.317885	0.655955
ILMN_1795918	CENTG3	0.064258	0.591124	0.6553823
ILMN_3206111	LOC399881	-0.06513	0.590195	0.6553227
ILMN_3235148	FLJ43681	0.1646	0.490397	0.654997
ILMN_1737644	TMEM219	0.089563	-0.56464	0.6542005
ILMN_3244646	RNU1G2	-0.01189	-0.64073	0.6526233
ILMN_1699496	PHF21A	0.175979	-0.47458	0.650559
ILMN_1662334	DNAJA3	0.645222	-0.00497	0.6501871
ILMN_1661306	LOC643433	-0.11025	0.539038	0.649284
ILMN_1761058	ACAD11	0.234147	-0.41498	0.649127
ILMN_1675222	PLXND1	0.578937	-0.0701	0.6490414
ILMN_2279635	EIF4G2	0.628034	-0.02055	0.6485798
ILMN_1775182	GSR	-0.26987	0.377332	0.647201
ILMN_2145143	FKBP9	0.603923	0.042724	0.6466467
ILMN_3254152	LOC100130750	0.556816	-0.08978	0.646596
ILMN_1781769	CCRL1	-0.27624	-0.37025	0.646485
ILMN_1709880	RPLP0	0.582837	0.063109	0.6459463
ILMN_2387742	HNRNPA1L2	0.34364	0.301488	0.645128
ILMN_1743770	SLC25A14	-0.44931	0.195785	0.645092
ILMN_2079803	LSM14A	-0.3278	0.316477	0.644276
ILMN_1772677	CNOT4	0.155927	0.488243	0.64417
ILMN_3258657	LOC100128859	-0.53445	0.108774	0.643224
ILMN_3202024	LOC392437	-0.55132	-0.09144	0.6427628
ILMN_2270053	F11R	0.531591	0.110697	0.642288
ILMN_1674394	C20orf3	0.618326	0.023344	0.64167
ILMN_1782897	CAPRIN1	-0.1186	0.522718	0.641319

ILMN_2188119	ARL16	0.373161	0.268013	0.641174
ILMN_2404385	REPIN1	0.350005	-0.29112	0.64112
ILMN_1795341	SFRS1	-0.00807	-0.63288	0.64095175
ILMN_1726547	MAP3K5	-0.44835	0.192418	0.64077
ILMN_1799815	XYLT2	-0.23549	0.404381	0.639875
ILMN_1651664	SDHALP1	-0.3905	-0.24894	0.639444
ILMN_1670932	LOC645737	-0.21401	0.425228	0.639237
ILMN_1714623	TOMM22	-0.24568	0.39222	0.637899
ILMN_1719158	CTBP1	0.208047	-0.42973	0.637779
ILMN_2111187	ELOVL6	0.253563	0.383877	0.63744
ILMN_2374692	WAC	-0.24767	0.389723	0.637392
ILMN_1805330	KLHL26	0.566683	0.070477	0.6371595
ILMN_3192046	LOC100129146	-0.41294	0.223277	0.636219
ILMN_1708841	GOLPH3	0.378572	0.256995	0.635567
ILMN_1810628	KIAA0367	-0.31117	0.323187	0.634355
ILMN_3289172	LOC100132774	-0.53811	0.095868	0.6339793
ILMN_3297996	LOC728732	0.287859	0.346046	0.633905
ILMN_2197946	SCG3	0.271546	-0.36218	0.63373
ILMN_3205781	LOC646316	0.22062	0.412754	0.633374
ILMN_1793829	TMCO1	-0.14971	-0.48353	0.633247
ILMN_1703196	PRKD3	0.325815	-0.30673	0.632547
ILMN_2283216	LRRN4CL	-0.14088	-0.49146	0.632344
ILMN_1815745	SOX4	-0.06648	-0.56504	0.6315205
ILMN_3238782	LOC728244	0.406821	-0.22412	0.630945
ILMN_3292970	LOC100131085	-0.27751	0.352307	0.629815
ILMN_1807972	MICAL1	0.407219	-0.22259	0.62981
ILMN_3245578	LOC729510	0.524032	-0.10531	0.629345
ILMN_3219739	LOC727970	0.610051	-0.01901	0.6290582
ILMN_1691175	ADH6	-0.09433	-0.53445	0.6287781
ILMN_1729319	USP7	-0.34411	0.283884	0.627995
ILMN_1773063	OSBPL1A	0.536127	0.091454	0.6275809
ILMN_1656920	CRIP1	0.560317	-0.06676	0.6270722
ILMN_2153332	ATXN1	-0.42687	0.1994	0.626267
ILMN_1800354	CST3	-0.30329	0.322939	0.626229
ILMN_1661622	TBC1D7	-0.48352	0.142516	0.626039
ILMN_1707240	PTBP2	-0.30106	-0.32441	0.625469
ILMN_3279628	LOC344593	0.362021	-0.26328	0.625301
ILMN_2126038	STMN2	-0.48519	-0.14004	0.625231
ILMN_2240221	SYTL2	0.275127	0.350094	0.625221
ILMN_1795905	ZBTB4	-0.02892	0.595662	0.6245839
ILMN_3262439	FLJ46552	0.335046	0.288902	0.623948
ILMN_1663799	RPL32	0.257223	0.366408	0.623631
ILMN_1696302	FABP5	0.575595	-0.04785	0.6234439
ILMN_2142815	RPL35	0.206137	-0.41719	0.623324
ILMN_3245778	LOC100133539	0.088528	0.533943	0.6224711
ILMN_2191759	LOC440396	0.480028	0.141859	0.621887
ILMN_1737157	GRAMD1A	0.165555	-0.45608	0.621631
ILMN_2397231	USP16	-0.10324	0.518315	0.621558
ILMN_1700690	VAT1	0.510266	0.111159	0.621425
ILMN_1663684	ATP13A3	0.315312	0.306015	0.621327
ILMN_3282321	LOC643336	0.406025	-0.21528	0.621302
ILMN_1796099	LOC644380	0.070942	-0.55032	0.6212664

ILMN_3201239	LOC389873	0.158552	0.462679	0.621231
ILMN_3210538	LOC646785	0.14781	0.473295	0.621105
ILMN_1803953	LOC388789	0.475174	0.145829	0.621003
ILMN_3294106	LOC100190938	0.411038	-0.20993	0.620972
ILMN_3302177	RPL14L	-0.57392	-0.04667	0.6205903
ILMN_1733927	TCEB2	0.345868	0.274581	0.620449
ILMN_2094718	TPT1	0.00609	0.614208	0.62029827
ILMN_2268921	GOLGA7	0.294861	0.325418	0.620279
ILMN_2225735	CRBN	0.581325	-0.03779	0.6191142
ILMN_3239000	LOC642617	0.320245	0.298287	0.618532
ILMN_1698117	DSCR8	0.161974	0.456105	0.618079
ILMN_3289144	LOC100132910	0.359952	0.257888	0.61784
ILMN_3219558	LOC100131364	0.375151	-0.24211	0.617264
ILMN_1767766	PRDX2	0.501592	-0.11532	0.616912
ILMN_2129505	CYBASC3	0.296771	-0.31926	0.61603
ILMN_2205896	MEIS3P1	-0.29414	0.321885	0.616024
ILMN_1683658	FKBP1A	0.504855	-0.11076	0.615618
ILMN_2267914	CD68	0.539867	-0.07496	0.6148238
ILMN_3282587	LOC644914	0.061712	-0.55305	0.6147639
ILMN_2396444	CD14	0.042137	0.572436	0.6145729
ILMN_3299356	LOC729004	0.244253	-0.37016	0.614414
ILMN_1694587	EEF1B2	0.365841	0.248539	0.61438
ILMN_1674891	KIAA1618	0.215288	0.397593	0.612881
ILMN_1663562	OR2A1	0.548859	0.063985	0.6128441
ILMN_3235632	ANXA2P2	-0.04953	0.563099	0.6126304
ILMN_1681812	HIF1AN	0.049378	0.563182	0.6125601
ILMN_1792489	ARPC2	-0.36703	0.245491	0.612519
ILMN_2178618	WDR44	0.222131	0.388859	0.61099
ILMN_1702265	HDHD2	0.124256	-0.48527	0.609529
ILMN_3225673	LOC730060	0.300034	0.308778	0.608812
ILMN_2365711	Sep-02	-0.03783	-0.57076	0.6085971
ILMN_3272996	LOC100129141	0.585462	-0.02282	0.6082801
ILMN_3232389	LOC100131608	-0.00481	-0.60347	0.60827925
ILMN_1813314	HIST1H2BK	-0.10667	-0.50042	0.607081
ILMN_3305025	LOC729684	0.56732	0.039292	0.6066115
ILMN_2210064	DNAJC25-GNG1(	0.107705	0.49861	0.606315
ILMN_1699576	CDON	0.349528	0.256237	0.605765
ILMN_1670033	HIST1H4I	0.53374	0.072009	0.6057491
ILMN_3248428	NCRNA00028	0.357406	0.24816	0.605566
ILMN_2100437	HBB	-0.05749	-0.54805	0.6055337
ILMN_1701629	FLJ45684	-0.48392	-0.12156	0.605484
ILMN_1769024	LOC644113	0.163963	0.441495	0.605458
ILMN_2344956	ACP1	0.191098	0.414263	0.605361
ILMN_2379644	CD74	0.107307	0.497788	0.605095
ILMN_1696935	RBM39	0.509788	-0.0943	0.604083
ILMN_1710598	SKI	0.302262	-0.30168	0.60394
ILMN_3251629	EIF4A1	0.402365	0.200631	0.602996
ILMN_1698246	MTMR11	-0.21465	-0.38823	0.602873
ILMN_1686750	MGEA5	0.155051	0.446554	0.601605
ILMN_1664371	HIATL1	0.196429	0.40428	0.600709
ILMN_3226301	LOC729798	0.309821	-0.28983	0.599652
ILMN_1655827	COPS2	0.330192	-0.26829	0.598478

ILMN_2116127	NPEPPS	0.205898	-0.39253	0.598427
ILMN_1803110	SF3B3	0.481938	-0.11414	0.596074
ILMN_2401155	PUM1	0.372525	0.222531	0.595056
ILMN_1756806	MCL1	0.277673	-0.31524	0.592908
ILMN_1783709	RRAGA	0.462681	0.130089	0.59277
ILMN_1801833	ARHGAP24	-0.27281	0.319542	0.592356
ILMN_1686623	CSF1R	0.345231	-0.24685	0.592084
ILMN_2373689	DIAPH3	0.334807	-0.25499	0.589796
ILMN_2248863	ZBTB38	0.106512	0.482041	0.588553
ILMN_1675852	LOC650518	0.070863	0.516943	0.5878058
ILMN_2361324	WDR20	-0.36162	0.225792	0.587409
ILMN_3244728	LOC100134159	0.191337	0.395925	0.587262
ILMN_2366790	DDX52	-0.18481	-0.40238	0.587187
ILMN_2365544	NHP2	0.425123	0.161949	0.587072
ILMN_3301528	LOC729954	0.134521	0.452365	0.586886
ILMN_1777286	LOC388344	-0.07046	0.515546	0.5860051
ILMN_3286870	LOC644191	0.475492	-0.11048	0.585971
ILMN_1805448	EPB41L2	-0.12712	0.458058	0.585173
ILMN_3188806	LOC100127893	0.065929	0.518463	0.5843923
ILMN_3285410	LOC642738	0.177093	0.407227	0.58432
ILMN_3302508	LOC729992	-0.31992	0.26422	0.584141
ILMN_2184789	HSCB	0.037681	0.546122	0.5838028
ILMN_1738243	RPS29	0.233351	0.350218	0.583569
ILMN_1740742	UROD	0.002032	0.581384	0.58341604
ILMN_1769207	KCTD7	0.316903	-0.26643	0.583331
ILMN_1768754	PILRB	-0.05614	-0.52713	0.5832689
ILMN_1806867	PPM1G	-0.12091	0.462324	0.583232
ILMN_3294074	LOC338870	0.184891	0.397795	0.582686
ILMN_1772651	CNOT2	-0.12664	-0.45529	0.581927
ILMN_2176768	SEPHS1	-0.34427	0.237627	0.581897
ILMN_3282174	LOC646688	0.447721	-0.1334	0.581119
ILMN_1766981	UNC50	-0.37841	-0.20231	0.580719
ILMN_1739496	PRRX1	0.223802	0.356497	0.580299
ILMN_2223772	GP6	0.095928	-0.48386	0.5797874
ILMN_2392286	IP6K1	-0.55713	0.022451	0.5795795
ILMN_1666553	SLC25A19	0.364329	0.214797	0.579126
ILMN_1698605	TMEM43	-0.12019	-0.45807	0.578257
ILMN_2182704	BIRC2	-0.22268	0.355284	0.577967
ILMN_1783424	PUM1	0.206535	0.37119	0.577725
ILMN_3280459	LOC100131686	-0.24146	0.3362	0.577662
ILMN_1663090	SON	-0.44429	0.131941	0.576235
ILMN_2183885	C7orf11	0.150913	0.425175	0.576088
ILMN_1685985	LOC649150	0.166112	0.409878	0.57599
ILMN_2051684	LOC401152	0.152187	0.423482	0.575669
ILMN_1669458	LOC651987	0.430772	0.144498	0.57527
ILMN_1749068	LOC645863	-0.45265	-0.12211	0.574756
ILMN_3293097	LOC645715	0.149242	0.425453	0.574695
ILMN_3272603	FAM60A	0.001157	-0.57335	0.57450473
ILMN_2313889	ZNF682	0.288257	0.285162	0.573419
ILMN_3200597	LOC441154	0.317062	0.256137	0.573199
ILMN_3202863	LOC389662	-0.1069	0.465875	0.572779
ILMN_1677396	NDFIP2	0.363294	-0.20872	0.572009

ILMN_1807044	UBAC1	0.007761	-0.56412	0.57188431
ILMN_3274666	LOC727826	-0.07659	0.495196	0.5717822
ILMN_1663541	B4GALT7	-0.33424	0.236899	0.571143
ILMN_1678143	ARHGDIB	0.184812	0.384971	0.569783
ILMN_1726437	EFCAB3	-0.3009	0.268853	0.569756
ILMN_1669931	TM9SF3	0.397431	0.171683	0.569114
ILMN_2157277	AKR1D1	0.425998	0.142451	0.568449
ILMN_1713162	GSTM2	0.236614	0.331194	0.567808
ILMN_3228508	LOC731642	-0.01388	0.553796	0.5676786
ILMN_1782567	GUCY1B3	-0.24186	-0.32579	0.567646
ILMN_2245686	GYG2	0.112002	-0.45496	0.566966
ILMN_2160005	NUMA1	0.528568	-0.0383	0.566872
ILMN_1723522	APOLD1	0.273695	0.292482	0.566177
ILMN_2167805	LUM	-0.19412	0.371574	0.56569
ILMN_2206554	SLC35F5	-0.05614	-0.50954	0.5656709
ILMN_1731720	PDRG1	0.262316	-0.3023	0.564615
ILMN_3244004	LOC100134743	0.494351	0.070003	0.5643541
ILMN_1674941	ANO6	-0.05645	0.50712	0.5635742
ILMN_3226814	LOC729646	0.482177	0.081033	0.5632103
ILMN_3267476	LOC100129243	-0.12075	0.441306	0.562055
ILMN_1706057	CS	-0.01778	-0.54347	0.5612467
ILMN_2150894	ALDH1B1	-0.39934	-0.16003	0.559367
ILMN_1698307	DBNL	-0.09178	0.467384	0.5591687
ILMN_1798360	CXCR7	0.395283	0.1636	0.558883
ILMN_2319994	RPL3	0.448278	0.110354	0.558632
ILMN_1794150	LOC654074	0.438889	0.119716	0.558605
ILMN_3247960	LOC100133937	0.211548	-0.34657	0.558122
ILMN_2225887	ATP5EP2	0.478914	-0.07893	0.5578473
ILMN_1796712	S100A10	0.076831	-0.48025	0.5570798
ILMN_1778929	LOC339047	-0.14757	-0.40934	0.556906
ILMN_3236637	PIP4K2A	0.311412	0.245083	0.556495
ILMN_1803476	KCTD20	0.130941	0.42408	0.555021
ILMN_1748911	SNAP23	-0.22857	0.326406	0.554977
ILMN_1794260	FBXL10	0.483609	-0.07077	0.5543762
ILMN_3219534	LOC100131504	-0.39536	0.158623	0.553979
ILMN_1666449	FAM134C	0.008477	0.545198	0.55367546
ILMN_1661173	TRIP4	0.304012	0.249592	0.553604
ILMN_2386530	RPLP1	-0.25213	0.301316	0.553441
ILMN_1696046	SIVA	0.468172	0.085063	0.553235
ILMN_1705814	KRT80	0.527692	0.025173	0.5528645
ILMN_2130838	UTP11L	-0.23207	0.320649	0.552721
ILMN_1727617	XRN2	0.148367	0.402061	0.550428
ILMN_1773073	PHYH	-0.27854	-0.27177	0.550314
ILMN_3280952	LOC391777	0.319211	0.229236	0.548447
ILMN_3206390	LOC727808	-0.03911	0.509191	0.5482983
ILMN_1797604	CAP1	-0.09648	0.451052	0.5475315
ILMN_3308718	MIR1826	-0.28897	-0.25848	0.547442
ILMN_2232166	CCDC90B	0.000918	0.546317	0.54723501
ILMN_1724941	CDCP1	0.167942	0.378575	0.546517
ILMN_1804498	C7orf27	-0.23382	0.312595	0.546418
ILMN_3235013	LOC730288	-0.24417	0.302216	0.546383
ILMN_1775257	PROK2	-0.06958	0.476722	0.5463058

ILMN_1721977	ARD1A	0.304251	-0.24	0.544246
ILMN_1747730	NOTCH2NL	0.385973	0.157487	0.54346
ILMN_1754584	POLR2J4	0.210752	-0.33269	0.543443
ILMN_1706643	COL6A3	0.297089	0.245793	0.542882
ILMN_1718336	C7orf50	0.126803	-0.416	0.5428
ILMN_3236661	SDR16C5	0.309582	-0.23288	0.542458
ILMN_2200659	SNHG5	-0.06004	-0.48168	0.54171
ILMN_1768483	KCNK3	0.218869	-0.32277	0.541643
ILMN_2196569	NUP93	0.062508	0.479053	0.5415606
ILMN_1739259	UBE4A	-0.08367	-0.45691	0.5405733
ILMN_1662351	PLA2G4D	0.096088	-0.4442	0.5402875
ILMN_1700466	LOC389517	0.080253	-0.45955	0.5398025
ILMN_1696186	LOC644889	0.377299	0.162381	0.53968
ILMN_2161007	GABPB2	-0.08319	0.456046	0.5392368
ILMN_1697642	BCAP29	0.525623	-0.01297	0.5385944
ILMN_1670796	EXOSC10	0.291519	-0.24706	0.538579
ILMN_1718309	COX15	0.044445	-0.49361	0.5380555
ILMN_1802905	PIAS4	0.265578	-0.27191	0.537485
ILMN_3227529	LOC729236	-0.04651	0.490752	0.5372596
ILMN_2054554	DTWD2	-0.03497	0.501326	0.5362955
ILMN_2215545	C3orf26	0.0789	0.457028	0.5359277
ILMN_2089175	SYAP1	0.076194	0.459662	0.5358562
ILMN_1792679	ITGA5	0.025904	-0.50994	0.535841
ILMN_3178258	FABP5L2	0.457827	-0.07774	0.535565
ILMN_1765122	MAP3K2	0.113912	-0.42143	0.535342
ILMN_3241524	ZBTB22	0.383506	0.151149	0.534655
ILMN_3238006	LOC730323	0.201761	-0.33276	0.534523
ILMN_2341645	HNRPH3	-0.04436	0.489693	0.5340521
ILMN_1726239	TBCA	0.279981	-0.25382	0.533805
ILMN_1741356	PRICKLE1	-0.51885	0.014705	0.5335585
ILMN_1674080	LOC728729	0.421542	-0.11175	0.533288
ILMN_1771201	TNRC6B	0.219267	0.3125	0.531767
ILMN_3283592	LOC442609	0.326691	0.204033	0.530724
ILMN_1685174	CPB1	-0.16977	-0.36007	0.529838
ILMN_1709132	ELP2	-0.06696	0.462419	0.5293769
ILMN_3246273	RNU1-3	-0.24178	-0.28684	0.528617
ILMN_3241758	POTEF	0.162292	-0.36623	0.528524
ILMN_1781999	ABCF2	0.260008	-0.2682	0.528211
ILMN_1664776	EFR3A	-0.29788	0.229337	0.527216
ILMN_2223720	ATMIN	0.472071	0.054316	0.526387
ILMN_2294762	AMY1A	0.437059	-0.089	0.5260579
ILMN_1772981	EPN1	0.509152	-0.01688	0.5260349
ILMN_1656233	LOC389517	-0.13396	0.391984	0.525942
ILMN_1735361	MAPK11	-0.25181	0.273711	0.525517
ILMN_1708451	DSCR5	-0.20327	0.321164	0.524431
ILMN_3284119	LOC399804	0.239797	0.284351	0.524148
ILMN_3273493	LOC100130092	0.263112	-0.2606	0.523711
ILMN_2364272	MBNL2	0.235181	0.287576	0.522757
ILMN_1775192	BCLAF1	0.168977	-0.3536	0.522581
ILMN_2223903	PPIC	-0.42894	-0.09345	0.5223907
ILMN_3307832	PCBD2	0.158632	-0.36369	0.522319
ILMN_1689908	ANKRD13A	-0.3196	0.201832	0.521435

ILMN_1673119	AFF1	0.009671	0.511676	0.52134706
ILMN_2252309	DPP7	-0.394	0.127231	0.521235
ILMN_2396875	IGFBP3	-0.33058	-0.19041	0.52099
ILMN_3193623	LOC100129657	0.365284	-0.15562	0.520907
ILMN_1755077	HEBP2	-0.07229	-0.44847	0.5207553
ILMN_3228860	ZNF788	0.157518	-0.36215	0.519672
ILMN_1803213	MXRA5	0.307752	-0.21038	0.51813
ILMN_3209631	LOC728060	0.39568	-0.12215	0.517828
ILMN_2175474	MTRF1L	-0.39496	-0.12191	0.51687
ILMN_3230286	LOC731605	0.149879	-0.36626	0.51614
ILMN_3261601	LOC100129334	0.256905	-0.25908	0.515983
ILMN_2399300	NAV2	0.437456	0.078009	0.5154654
ILMN_3211302	LOC646909	0.29327	-0.22174	0.515009
ILMN_1750130	GSPT1	0.060518	-0.45409	0.5146063
ILMN_1712305	CYBRD1	-0.12696	-0.38729	0.514242
ILMN_1693996	ZNF578	0.085345	-0.42853	0.5138761
ILMN_3229446	LOC731308	0.11614	-0.39693	0.513065
ILMN_1777299	LOC652771	-0.23064	0.281972	0.512612
ILMN_2162799	AHR	-0.41804	-0.09454	0.5125726
ILMN_1741613	SERINC1	0.194997	0.317441	0.512438
ILMN_2350240	GTF2I	-0.27838	0.234023	0.512407
ILMN_3305508	LOC728779	-0.37546	0.135456	0.510919
ILMN_1746396	CTNNB1	0.393452	-0.11731	0.510766
ILMN_1794799	LOC727815	-0.04317	0.46623	0.5093955
ILMN_3242912	LOC100133564	0.120278	-0.38882	0.509097
ILMN_2183891	PEX5L	-0.29486	-0.21379	0.508641
ILMN_1675541	EEF1B2	0.081287	0.426843	0.5081299
ILMN_1778523	KLF9	0.307354	0.199495	0.506849
ILMN_3234963	LOC730153	0.063463	-0.44329	0.5067515
ILMN_1745885	POLR2F	0.438013	0.067991	0.5060042
ILMN_1746085	IGFBP3	-0.37801	-0.12797	0.505974
ILMN_1792660	CAMSAP1L1	-0.42297	-0.08276	0.5057299
ILMN_1681967	C10orf4	0.260645	0.244923	0.505568
ILMN_1795243	LOC220433	0.346584	0.158913	0.505497
ILMN_2290808	RPL21	0.170727	0.334602	0.505329
ILMN_3232572	LOC728531	-0.06767	0.437447	0.5051211
ILMN_3201600	LOC100132804	0.149004	-0.35582	0.504827
ILMN_3278146	LOC645231	0.217994	-0.28683	0.504825
ILMN_1680313	STX4	0.275764	-0.22819	0.503953
ILMN_3277137	LOC389404	-0.48989	0.013781	0.5036704
ILMN_2287168	SAR1B	0.343162	0.160227	0.503389
ILMN_1696031	C15orf21	-0.40308	-0.09917	0.5022401
ILMN_3177285	HNRNPR	-0.1677	-0.33435	0.502052
ILMN_2320850	UBE2D3	-0.05303	0.448158	0.5011906
ILMN_1708130	EHF	-0.03982	-0.46065	0.5004744
ILMN_3244157	SNORD83B	0.062269	-0.43782	0.5000899
ILMN_1800750	ZNF692	-0.36886	0.130509	0.499367
ILMN_3248966	MMADHC	0.230168	0.268043	0.498211
ILMN_3241707	LOC729339	0.209559	0.28841	0.497969
ILMN_1343291	EEF1A1	0.295577	-0.20237	0.497948
ILMN_2253286	PRKCZ	-0.34276	0.15438	0.497138
ILMN_1811328	DPP7	0.027575	0.469212	0.496787

ILMN_3306270	RPL31P10	0.357645	0.138581	0.496226
ILMN_2224300	SNAPC3	-0.16101	0.33507	0.496083
ILMN_2175131	TMEM14C	-0.02089	-0.47492	0.495808
ILMN_3291492	LOC100133013	0.129588	0.365663	0.495251
ILMN_1675640	OAS1	0.353905	0.140817	0.494722
ILMN_3211488	LOC400061	0.399023	-0.09534	0.4943654
ILMN_1707506	YTHDC1	0.330828	-0.16283	0.493659
ILMN_1653524	SIKE	0.059882	-0.43329	0.4931757
ILMN_2179778	PHLDB2	-0.21099	0.281747	0.492732
ILMN_3251472	TOMM20	0.470161	-0.02253	0.492695
ILMN_1862180	MGC3032	-0.05956	-0.43187	0.4914316
ILMN_1740620	FLJ40113	0.207172	-0.28348	0.490648
ILMN_3244025	LOC401650	0.053357	-0.43702	0.4903787
ILMN_1679897	IGFL3	-0.25618	0.233952	0.490135
ILMN_3256926	LOC100130764	0.108978	-0.38116	0.490133
ILMN_1800739	SPINT2	-0.18656	-0.30313	0.48969
ILMN_1803775	HSPE1	-0.35645	-0.13311	0.489553
ILMN_1733356	PREI3	-0.08637	0.402742	0.4891157
ILMN_1724194	NPEPL1	0.126246	0.362361	0.488607
ILMN_3307892	PARVA	0.184573	-0.3039	0.488476
ILMN_1786847	TGM3	-0.10062	0.387841	0.488458
ILMN_1696276	ZNF653	-0.17637	0.311666	0.488037
ILMN_2400183	DPH5	0.207172	-0.28009	0.487263
ILMN_1715169	HLA-DRB1	-0.34817	0.139001	0.48717
ILMN_2287707	POT1	-0.00338	0.483675	0.48705394
ILMN_3299407	LOC728553	-0.08733	0.399665	0.4869936
ILMN_1759563	NRIP3	0.372445	0.113934	0.486379
ILMN_1699208	NAP1L1	-0.0586	0.427713	0.4863157
ILMN_1665357	EPS15	0.128315	-0.35634	0.484659
ILMN_3245015	LOC440563	0.198737	-0.28539	0.484124
ILMN_2179018	NDUFAB1	0.074285	-0.40875	0.4830385
ILMN_3245000	LOC729617	0.388041	0.094501	0.4825424
ILMN_1800786	LOC727726	0.09744	-0.38459	0.4820283
ILMN_1659523	USP39	0.057733	0.423145	0.4808783
ILMN_1705148	LOC441052	0.357087	0.123645	0.480732
ILMN_1748844	CNKSR3	0.223007	-0.25729	0.480298
ILMN_1683538	CSDE1	0.374355	0.105324	0.479679
ILMN_3290353	LOC644790	0.149561	0.329886	0.479447
ILMN_1690252	ALKBH2	-0.09385	0.384238	0.4780916
ILMN_3275696	LOC100131940	-0.38931	-0.08833	0.4776392
ILMN_1796244	CD2BP2	0.458305	0.01732	0.475625
ILMN_3251251	GNL3L	0.189427	0.285582	0.475009
ILMN_1784352	CCM2	0.167863	0.306589	0.474452
ILMN_3249491	LOC100132733	-0.21305	-0.26048	0.473535
ILMN_2377862	RAB6A	0.367273	-0.10534	0.47261
ILMN_1719656	MRPL38	0.412311	0.059979	0.47229
ILMN_1789186	OBFC1	0.197464	-0.27389	0.471354
ILMN_1682685	MGC57359	0.467774	0.003071	0.4708448
ILMN_3290199	LOC441073	0.266613	0.20356	0.470173
ILMN_1721026	SAMHD1	-0.36854	0.101022	0.469562
ILMN_1762725	EIF3L	-0.25897	0.210537	0.469505
ILMN_3266411	LOC100130892	-0.18998	0.279191	0.469169

ILMN_1697166	C9orf78	0.284119	0.184677	0.468796
ILMN_1651486	COX11P	0.250857	0.217774	0.468631
ILMN_2113738	C8orf45	0.064099	0.403913	0.4680121
ILMN_1702341	LOC388237	-0.45886	-0.00717	0.4660224
ILMN_1749403	TSPAN33	-0.05876	0.407203	0.4659649
ILMN_2399893	RPS24	0.232635	-0.23312	0.465753
ILMN_1768820	CYP11A1	-0.15934	0.304222	0.463564
ILMN_1800871	RAB6A	-0.09178	0.37029	0.4620747
ILMN_3235216	IFT20	-0.42854	-0.03255	0.4610844
ILMN_1803312	DIMT1L	0.348096	0.112431	0.460527
ILMN_3205271	LOC100132863	-0.01953	-0.44061	0.4601463
ILMN_3280628	LOC100132658	0.339422	0.120574	0.459996
ILMN_1776047	LOC646197	-0.0029	0.455425	0.4583265
ILMN_1670841	CPNE1	0.150516	-0.30748	0.457999
ILMN_1703973	LOC654002	0.452098	0.005811	0.45790858
ILMN_3309453	RNU4-1	0.071818	0.385948	0.4577657
ILMN_1669424	LOC646531	0.020334	0.437305	0.4576389
ILMN_2389957	RNF7	-0.16483	0.291848	0.456681
ILMN_2312296	PCBP2	0.189427	-0.26667	0.456097
ILMN_2256765	CDC16	0.21306	-0.24282	0.455883
ILMN_2152010	CBWD1	-0.4283	-0.02698	0.455284
ILMN_1654289	ELK1	0.315471	0.139586	0.455057
ILMN_1703433	PLSCR3	0.337672	-0.11732	0.454992
ILMN_1753890	TMEM97	-0.34634	-0.10832	0.454658
ILMN_2052495	DNHL1	-0.01142	0.442738	0.4541538
ILMN_2386444	ANGPTL4	0.350881	0.102443	0.453324
ILMN_3309950	MIR644	-0.13452	-0.31858	0.453094
ILMN_1670948	TSR2	-0.27807	0.174872	0.452937
ILMN_2252295	ELF2	-0.18202	0.270788	0.452809
ILMN_1731546	RPL26	0.198259	-0.25328	0.451538
ILMN_2059797	NOL3	0.409367	0.042156	0.4515226
ILMN_1717707	PSTK	-0.22833	0.222774	0.451106
ILMN_1725862	USP3	0.189984	-0.2607	0.450684
ILMN_1708006	MICB	0.115822	0.331046	0.446868
ILMN_1736829	MDM2	-0.24934	0.197465	0.446805
ILMN_3226505	MSL3	0.039829	0.405902	0.4457313
ILMN_1658531	LOC653702	-0.05383	0.391759	0.4455873
ILMN_2365686	ALG8	0.244014	-0.20151	0.445521
ILMN_2175894	HNRPR	0.411038	-0.03441	0.4454484
ILMN_1688526	ARL5A	0.423929	0.021308	0.4452374
ILMN_1718807	SMC3	0.412391	0.032392	0.4447828
ILMN_1753745	HDDC2	0.219665	0.22462	0.444285
ILMN_3211055	LOC100133286	0.44048	-0.00282	0.44330299
ILMN_2323048	ERP29	0.058688	0.383468	0.4421561
ILMN_1805127	LOC653620	-0.23478	-0.20585	0.440623
ILMN_3200484	LOC126235	-0.03839	0.402108	0.4404991
ILMN_3238078	SNORA45	0.320325	0.119313	0.439638
ILMN_1657618	WTAP	0.197384	0.24087	0.438254
ILMN_3279935	LOC646909	0.110172	-0.32694	0.437112
ILMN_1654915	LOC646786	-0.40061	-0.0353	0.435906
ILMN_1748707	CRELD2	0.037044	0.398209	0.4352532
ILMN_1686135	CCDC45	-0.12258	0.312323	0.434903

ILMN_2180519	LOC729603	0.418598	-0.01626	0.4348595
ILMN_2219628	SSB	-0.4088	0.025942	0.4347458
ILMN_3288242	LOC100131071	0.363533	0.068832	0.4323645
ILMN_3198367	LOC646347	0.133646	0.298624	0.43227
ILMN_1683498	LOC441034	0.393532	0.038629	0.4321608
ILMN_1663415	LOC641750	-0.15719	0.274717	0.431911
ILMN_1673795	HSD17B4	0.257064	0.174062	0.431126
ILMN_1814526	ADD3	-0.38851	0.041576	0.4300887
ILMN_3286235	LOC100133172	0.086459	-0.34333	0.4297902
ILMN_1731248	LOC648705	0.287063	0.142273	0.429336
ILMN_1808939	RPS6	0.158712	0.270321	0.429033
ILMN_1813837	C9orf9	-0.41382	-0.0132	0.4270133
ILMN_1737078	LOC653059	0.269716	0.157138	0.426854
ILMN_2215061	ORC6L	0.425043	0.000313	0.42535627
ILMN_1691097	HSP90AA1	-0.07547	-0.34879	0.4242652
ILMN_1658283	RPL17	0.039113	0.385048	0.4241611
ILMN_1800796	RPL37	0.230884	0.190488	0.421372
ILMN_1710378	LOC653489	0.226349	-0.1946	0.420951
ILMN_1731174	LOC647856	0.028212	-0.39179	0.4200006
ILMN_1768480	VGLL4	0.379448	0.040475	0.419923
ILMN_1750402	SNORD107	-0.1903	0.228538	0.418834
ILMN_1698404	ERN1	-0.10022	0.317714	0.417933
ILMN_1738607	LOC646350	0.257064	-0.16061	0.41767
ILMN_3289726	LOC653079	0.331147	0.086507	0.4176539
ILMN_3280496	LOC100131526	0.370217	-0.04729	0.4175097
ILMN_1785990	LOC644097	0.357485	0.057701	0.4151857
ILMN_1694259	NSA2	-0.18727	0.226579	0.413852
ILMN_1759954	PTMA	0.41255	0.000207	0.41275675
ILMN_3250132	LOC653381	0.007363	-0.40528	0.41263844
ILMN_2375003	MAP4K4	0.002907	-0.40949	0.41240134
ILMN_1696699	FAM184A	-0.13865	0.273143	0.411796
ILMN_3283775	LOC100131205	0.194997	0.215857	0.410854
ILMN_3266894	LOC100128816	0.119402	-0.29118	0.410583
ILMN_2122669	TOPORS	0.199533	-0.20909	0.408627
ILMN_1778128	LOC642780	0.20749	0.200033	0.407523
ILMN_1708296	DEAF1	0.113912	0.293553	0.407465
ILMN_2122103	ETS1	0.306399	0.100632	0.407031
ILMN_1785113	MUT	0.275605	0.13087	0.406475
ILMN_1755138	NEK8	0.345072	0.060985	0.4060569
ILMN_2126832	SEC24A	0.120755	0.284546	0.405301
ILMN_2155719	NBPF10	-0.07643	0.328199	0.4046261
ILMN_1676385	PAK2	0.21871	-0.18575	0.404459
ILMN_1776480	HINFP	0.216004	0.187311	0.403315
ILMN_1709054	LOC648237	0.325656	0.077418	0.4030737
ILMN_1660577	ATP5G2	-0.38549	0.017279	0.4027676
ILMN_1774196	URM1	0.363612	0.037499	0.4011105
ILMN_1692973	BICD1	-0.31905	-0.08152	0.4005652
ILMN_2230162	FLJ44124	0.038715	0.361734	0.4004493
ILMN_3238058	LOC151162	-0.10221	0.298151	0.40036
ILMN_3294235	LOC100133177	0.165157	-0.23497	0.400128
ILMN_3219695	LOC441711	-0.34355	0.056245	0.399799
ILMN_3272424	LOC100128836	0.179321	-0.22017	0.399492

ILMN_2167617	NACA	0.286267	0.111585	0.397852
ILMN_2307450	ZNF302	0.317619	0.080039	0.3976581
ILMN_1756360	RPL35A	0.068317	0.328643	0.3969595
ILMN_1658067	LOC647474	0.029564	-0.36641	0.3959734
ILMN_1701308	COL1A1	-0.29215	-0.10357	0.395724
ILMN_1664675	ARMCX3	0.082162	-0.3119	0.3940602
ILMN_1764803	SMU1	0.260565	-0.133	0.39356
ILMN_1654663	LOC642843	0.224757	0.168765	0.393522
ILMN_1793525	KIR2DS3	-0.00696	0.386256	0.39321573
ILMN_2299795	CPM	0.052402	-0.34076	0.3931649
ILMN_2060115	SORL1	-0.23311	0.159759	0.392866
ILMN_2350122	TRIM13	0.2951	-0.09751	0.3926141
ILMN_1761574	CHRM2	-0.2935	-0.09858	0.3920822
ILMN_1682873	LOC388474	0.382551	-0.00721	0.38976474
ILMN_2082762	SNORD68	-0.00752	0.381841	0.38935774
ILMN_2265995	NBPF3	-0.03919	-0.34971	0.3888969
ILMN_3252113	LOC100130107	0.098316	0.289399	0.3877146
ILMN_3195253	LOC100130892	-0.00282	0.384267	0.38708892
ILMN_1761912	MGAT1	0.321996	0.064636	0.386632
ILMN_1752755	VWF	0.265658	0.120834	0.386492
ILMN_1709891	LOC647074	0.12736	0.258575	0.385935
ILMN_2067421	PP8961	0.305126	0.079885	0.3850113
ILMN_2149226	CAV1	-0.07977	-0.30464	0.3844122
ILMN_3289489	LOC387753	0.238842	0.145048	0.38389
ILMN_1784299	C19orf43	0.062269	0.318175	0.3804439
ILMN_1681490	ZNF568	0.176536	0.202856	0.379392
ILMN_1811104	KTELC1	0.124813	-0.25455	0.379365
ILMN_2190942	FIP1L1	-0.25817	-0.12017	0.378344
ILMN_3308138	RNU4-2	0.315869	-0.06168	0.3775529
ILMN_1746604	IL6ST	-0.15027	0.226289	0.37656
ILMN_3203666	LOC645001	0.350085	0.026179	0.3762635
ILMN_1692664	PRR5	0.237171	-0.13903	0.376196
ILMN_2346137	ZNF557	0.243537	0.132456	0.375993
ILMN_1738866	DEXI	0.17574	0.198755	0.374495
ILMN_2097546	NUDC	0.234067	0.139622	0.373689
ILMN_1800942	KCTD6	-0.01842	-0.35501	0.3734243
ILMN_2308849	MYADM	0.330112	-0.04331	0.3734222
ILMN_1660439	LOC651149	0.328759	0.044558	0.3733171
ILMN_1809010	PSMC3	0.086857	0.286298	0.373155
ILMN_1660832	LOC285053	0.103249	0.269108	0.372357
ILMN_1676159	MST4	-0.15576	-0.2157	0.371459
ILMN_1810532	CAV3	0.220063	-0.14931	0.369373
ILMN_3247872	LOC100134047	0.184971	0.179689	0.36466
ILMN_1660186	SYF2	0.326293	0.038002	0.3642945
ILMN_1705301	TEAD4	0.163168	-0.20031	0.36348
ILMN_1682711	LOC493754	0.190302	0.171452	0.361754
ILMN_2252554	MANBAL	0.235579	-0.12608	0.361657
ILMN_2093389	SNAPC1	0.158234	-0.20294	0.361173
ILMN_1664920	C19orf12	0.329635	-0.03149	0.3611221
ILMN_1661002	RFWD2	-0.03409	-0.32676	0.3608562
ILMN_1777424	GAGE1	0.280936	0.078353	0.3592887
ILMN_3292572	LOC390183	-0.08924	0.269907	0.3591454

ILMN_1697409	TNFRSF14	0.236693	0.122313	0.359006
ILMN_1651760	LOC646537	-0.23661	0.12168	0.358288
ILMN_1720745	LOC645385	0.08439	0.273818	0.3582083
ILMN_3242223	LOC100132228	0.304728	-0.05345	0.3581807
ILMN_2325008	DHX40	-0.11741	-0.24011	0.357514
ILMN_3236130	LOC100132547	0.308627	0.048878	0.3575048
ILMN_1743097	XRCC6	-0.3422	-0.01447	0.3566755
ILMN_1690224	LOC442572	-0.12155	0.234556	0.356101
ILMN_2162328	PTS	-0.11789	-0.23591	0.353791
ILMN_1790781	DHRS13	0.291042	0.062612	0.3536542
ILMN_1815707	CALML4	0.299477	0.054026	0.353503
ILMN_1655765	MRPS21	0.125768	-0.22705	0.352815
ILMN_1744963	ERO1L	0.114389	-0.23713	0.35152
ILMN_1808837	LOC644029	0.016117	0.334034	0.3501505
ILMN_2211950	SRP14P1	0.306956	-0.04249	0.3494496
ILMN_3279877	LOC645430	0.188074	0.160948	0.349022
ILMN_2311278	ADD3	0.117493	-0.22869	0.346185
ILMN_2165354	DCLK1	-0.13404	0.211004	0.345042
ILMN_3212284	LOC645693	0.111684	0.232277	0.343961
ILMN_1772973	FAM82B	-0.16165	-0.18212	0.343772
ILMN_1678968	GEM	0.16468	-0.17881	0.343488
ILMN_2306189	MAGED1	0.221972	-0.12124	0.343215
ILMN_3202734	LOC100132742	0.267409	-0.07566	0.343064
ILMN_2074860	RN7SK	-0.14661	0.195944	0.342555
ILMN_3211047	LOC646201	0.239797	0.102685	0.342482
ILMN_1777296	ACTB	-0.14184	-0.20051	0.342343
ILMN_1661332	ZNF334	0.157995	0.18389	0.341885
ILMN_1801313	SIAH2	0.223245	0.11784	0.341085
ILMN_1778032	SURF6	0.28396	-0.05661	0.3405667
ILMN_1694385	YWHAB	-0.03035	-0.30963	0.3399792
ILMN_1695034	LOC642817	0.240354	-0.09954	0.3398978
ILMN_1776347	TCP1	-0.17343	-0.16582	0.339246
ILMN_1810214	JUND	-0.04348	0.294943	0.3384268
ILMN_1805996	SIN3A	-0.29963	0.037037	0.336667
ILMN_3298544	LOC729157	0.092666	0.243633	0.3362989
ILMN_1695276	MAPRE2	-0.18019	0.156049	0.33624
ILMN_1690268	HNRPUL1	-0.06576	0.269735	0.3354993
ILMN_3231558	LOC100134648	0.266215	0.067376	0.3335908
ILMN_3259223	LOC100129685	0.107387	0.225271	0.332658
ILMN_1734428	LSM12	-0.23279	-0.09975	0.3325389
ILMN_1726153	MGC4677	0.029007	-0.30353	0.3325373
ILMN_1760647	HOXA3	0.139455	-0.19293	0.332382
ILMN_1758649	LOC647589	-0.08836	-0.24315	0.3315121
ILMN_1723185	ELOF1	0.261838	-0.06943	0.3312679
ILMN_2124757	RPL23AP13	0.290962	-0.04016	0.3311182
ILMN_1728870	DDX3X	0.269	0.061139	0.3301388
ILMN_3278157	LOC653156	0.182981	0.146806	0.329787
ILMN_2154566	RPL10A	0.16834	0.160339	0.328679
ILMN_3228108	LOC728693	0.066566	0.261397	0.3279629
ILMN_2117223	ROD1	0.299397	-0.02856	0.327955
ILMN_1719224	C17orf45	0.316744	-0.01115	0.3278928
ILMN_2389429	DCUN1D4	-0.20756	0.119822	0.327386

ILMN_3215954	LOC653079	0.144707	0.181843	0.32655
ILMN_2340643	INSC	0.089403	0.235863	0.3252664
ILMN_1679809	GSTP1	0.320086	-0.00411	0.32419899
ILMN_2247594	RPLP1	-0.23374	0.089022	0.3227648
ILMN_3238837	MSTO2P	0.263907	-0.05832	0.3222298
ILMN_1709439	CHMP1A	0.179719	-0.14037	0.320093
ILMN_1758918	BRD2	0.232635	-0.08672	0.3193557
ILMN_1743396	ACOX3	0.309503	0.008828	0.31833148
ILMN_3241568	LOC100134152	0.301148	0.016906	0.3180538
ILMN_2180827	MEPCE	0.238285	0.076708	0.3149926
ILMN_3280294	LOC100131737	0.064656	0.250249	0.3149051
ILMN_2310685	FOXK2	0.103249	0.210921	0.31417
ILMN_1777096	TDG	-0.01388	0.299961	0.3138436
ILMN_3280943	LOC389156	0.11248	0.196873	0.309353
ILMN_1809583	CREBBP	0.11622	0.189488	0.305708
ILMN_1672863	C3orf46	0.023915	0.280801	0.3047157
ILMN_3216979	LOC646949	0.291599	0.010971	0.3025696
ILMN_1673638	RPS3A	0.073728	-0.22869	0.3024195
ILMN_1775703	TRAPPC6A	0.244332	0.057866	0.3021984
ILMN_2133316	GIMAP7	0.095928	0.204335	0.3002634
ILMN_1747119	FBXO46	0.277196	0.022504	0.2996997
ILMN_1787251	DAAM1	0.189745	-0.10936	0.299106
ILMN_3246388	MED14	0.280618	0.018007	0.2986245
ILMN_3241598	LOC100134563	0.041262	-0.25672	0.2979786
ILMN_2374865	ATF3	-0.03298	0.264989	0.2979692
ILMN_3275447	LOC391126	-0.14231	0.155463	0.297777
ILMN_1761858	MID1	-0.11677	-0.17967	0.296437
ILMN_1724986	WDR53	-0.11518	0.181032	0.296211
ILMN_1786343	LOC649768	0.043251	-0.24876	0.2920149
ILMN_3249658	LOC100134868	0.105955	0.185885	0.29184
ILMN_1675085	UBA6	0.277992	0.013781	0.2917734
ILMN_1810127	ZNF789	0.122824	-0.16866	0.291484
ILMN_1762426	DHFRL1	0.280459	0.010627	0.2910864
ILMN_3227604	LOC729342	0.125211	0.165144	0.290355
ILMN_3247979	LOC100132024	0.207092	0.081613	0.2887052
ILMN_1803385	GOLGA8B	0.196986	-0.09092	0.2879021
ILMN_1728907	SCAMP1	0.06776	-0.21995	0.2877055
ILMN_3306215	LOC729926	0.214811	0.072281	0.2870923
ILMN_1786359	LOC346950	-0.03871	0.248373	0.2870824
ILMN_1772998	LOC647436	-0.06839	0.215513	0.2839032
ILMN_1652000	FAM156A	-0.01205	-0.26901	0.2810604
ILMN_1738276	TMEM185A	0.179321	0.101389	0.28071
ILMN_1672614	LOC120364	0.16842	0.112124	0.280544
ILMN_2258689	SORBS1	0.181151	0.098792	0.2799425
ILMN_2101650	MBTD1	0.20001	0.077731	0.2777413
ILMN_1660477	LOC652608	0.027655	-0.2474	0.2750516
ILMN_1669818	ESD	0.112639	-0.16219	0.274831
ILMN_1736847	MED8	0.024233	-0.24899	0.273222
ILMN_1741054	SLC5A6	0.019936	0.253196	0.273132
ILMN_3279144	LOC100131672	0.150913	0.120864	0.271777
ILMN_1694240	MAP2K1	-0.1303	-0.1414	0.271702
ILMN_2412380	TSC22D1	-0.22626	0.045156	0.2714187

ILMN_1710354	KIFC3	0.17582	-0.09485	0.2706653
ILMN_2346649	ZNF473	-0.00847	-0.26077	0.26923662
ILMN_2409720	SLA2	0.070783	-0.19835	0.2691363
ILMN_2411384	MGC57359	0.090279	-0.17852	0.2687967
ILMN_1754145	CAPRIN1	0.217437	-0.04938	0.2668185
ILMN_1676016	C1orf152	-0.07046	-0.19621	0.2666701
ILMN_1756049	NT5DC3	0.262157	-0.0033	0.2654593
ILMN_3200362	LOC727908	-0.22881	-0.0342	0.2630132
ILMN_2377174	SYPL1	-0.01293	-0.24964	0.2625677
ILMN_1712031	LOC653629	0.203511	-0.05559	0.2591058
ILMN_2251895	CBFA2T2	0.073011	-0.18577	0.2587783
ILMN_1678859	LOC649210	-0.15489	-0.10314	0.258022
ILMN_1682572	KIAA0528	0.084868	0.172263	0.2571307
ILMN_3302139	LOC729687	-0.08271	0.173458	0.2561714
ILMN_1686948	CASC4	0.172319	-0.07875	0.2510688
ILMN_1733985	SIRT3	0.115822	0.134509	0.250331
ILMN_1667001	LOC642516	-0.06266	0.187648	0.2503089
ILMN_1775473	LOC400652	0.160223	-0.08951	0.2497308
ILMN_1754529	SPG7	0.098475	-0.15088	0.2493587
ILMN_3242428	LOC100134624	0.069431	-0.17757	0.2470015
ILMN_1680644	MIOS	-0.04531	0.201098	0.246412
ILMN_1737297	LOC642377	-0.09178	0.154161	0.2459457
ILMN_2358783	ASB3	0.029326	0.214454	0.2437796
ILMN_2137464	DVL3	0.054869	-0.18605	0.2409136
ILMN_3296267	LOC727970	0.133407	-0.10702	0.24043
ILMN_3225449	LOC729677	-0.12377	-0.11521	0.238986
ILMN_1656285	METTL7A	0.095212	-0.14367	0.2388822
ILMN_2204664	NBPF14	0.149322	0.08946	0.2387817
ILMN_3211906	LOC647030	0.180355	0.058399	0.238754
ILMN_1791749	CLEC2L	0.085982	0.150072	0.2360537
ILMN_1742143	HIST1H2AD	-0.19809	-0.0344	0.2324984
ILMN_1780291	NFAT5	0.156245	0.072352	0.2285974
ILMN_2285708	C6orf48	0.063303	0.16267	0.2259734
ILMN_1666638	LOC645790	0.188313	-0.03562	0.2239364
ILMN_1779264	PSMG1	0.218153	0.005586	0.22373872
ILMN_1712455	RBM4	0.174945	-0.04813	0.223072
ILMN_3228482	LOC728485	-0.01722	-0.20522	0.2224427
ILMN_1693107	MLH3	-0.06974	-0.15221	0.221952
ILMN_1666690	ACRC	0.026859	-0.19461	0.2214729
ILMN_1743395	LOC728320	-0.16085	0.060512	0.2213655
ILMN_1730622	EVL	0.14789	0.071838	0.2197275
ILMN_2191003	KRTAP5-1	-0.21258	0.005325	0.21790235
ILMN_1670438	PRAMEF7	-0.18576	0.029066	0.2148272
ILMN_3241904	LOC100134000	0.177173	-0.03712	0.2142936
ILMN_2192694	EIF3M	0.119959	-0.09285	0.2128101
ILMN_2087528	CPSF3	-0.05996	0.151575	0.2115305
ILMN_3299187	LOC728782	0.07142	0.139752	0.2111718
ILMN_3208229	LOC642738	0.093939	-0.11672	0.210661
ILMN_3292163	LOC391532	0.06577	0.144397	0.2101671
ILMN_2192032	SRP19	0.208684	0.00071	0.20939374
ILMN_1784827	C1orf147	0.156245	-0.05222	0.208461
ILMN_2313158	MBNL1	-0.00051	0.203797	0.2043113

ILMN_1688113	LOC645974	0.090358	-0.11346	0.2038202
ILMN_1662879	UBE2MP1	-0.17176	-0.03177	0.2035213
ILMN_1749396	LSR	-0.19857	0.004532	0.20310441
ILMN_1737588	C21orf33	0.180594	0.022137	0.2027308
ILMN_3263329	GAR1	0.018981	-0.1816	0.2005761
ILMN_1664956	SSU72	-0.19507	0.002065	0.19713583
ILMN_1654414	ACSL3	0.167226	0.029338	0.1965644
ILMN_1745900	LOC641849	0.110888	-0.08519	0.196082
ILMN_3235584	TRIM66	-0.02606	0.169351	0.1954083
ILMN_1764494	ATP5A1	-0.16157	0.033066	0.1946364
ILMN_1668411	FHL2	-0.15194	-0.04022	0.1921574
ILMN_1753468	CD63	-0.04293	0.14738	0.1903068
ILMN_1726434	UNC45A	0.153141	-0.03634	0.1894804
ILMN_1772645	AGK	0.137147	0.051174	0.1883208
ILMN_2275098	DTX2	0.078979	0.10704	0.1860193
ILMN_2138435	MRPS27	0.155927	0.028433	0.18436
ILMN_1661596	FLVCR1	0.08813	-0.09518	0.1833069
ILMN_1661687	IL21R	0.110968	0.072281	0.1832493
ILMN_3210171	LOC389156	0.137863	0.041138	0.1790008
ILMN_1754912	GLE1	0.061235	-0.11635	0.1775835
ILMN_1682495	FOXP1	-0.12385	0.050523	0.1743759
ILMN_3239395	LOC100132883	-0.02868	0.14335	0.1720332
ILMN_2401779	FAM102A	0.034339	0.136853	0.1711917
ILMN_1693341	SNRPN	-0.08932	0.075838	0.1651557
ILMN_3198499	LOC654350	0.135635	0.028303	0.1639378
ILMN_1677239	CCDC14	0.054073	-0.1097	0.1637709
ILMN_1782579	IMMT	0.081605	-0.08173	0.1633374
ILMN_1748563	LOC85391	0.152903	-0.00965	0.16255473
ILMN_2203876	CCDC68	-0.09059	-0.07166	0.1622518
ILMN_3199955	LOC645430	0.008716	0.15193	0.16064618
ILMN_1815292	RPL7	-0.08279	0.069092	0.1518848
ILMN_3230572	NCRNA00152	0.113196	-0.03842	0.1516125
ILMN_1740772	APBB3	0.014048	0.13661	0.1506576
ILMN_2299072	CROP	0.012934	-0.13458	0.1475086
ILMN_1791332	ATP5O	0.068078	0.075323	0.1434007
ILMN_1731619	DAD1	0.11606	-0.02704	0.1431031
ILMN_1687896	PIK3C3	0.094019	0.044771	0.1387897
ILMN_1813669	ANKS1A	0.100623	-0.03772	0.1383412
ILMN_3243606	C14orf174	-0.0582	0.079779	0.1379836
ILMN_2290732	ZNF626	-0.05176	0.086075	0.1378343
ILMN_1666361	SLC23A2	0.025347	-0.1123	0.137643
ILMN_1800889	FIG4	0.0415	0.094655	0.1361555
ILMN_3236892	LOC100134333	0.023676	0.111443	0.1351189
ILMN_1746465	FJX1	-0.12202	0.010432	0.1324551
ILMN_1719316	TMED3	-0.1182	0.011704	0.1299074
ILMN_1748034	KLHDC4	-0.09513	0.034232	0.1293589
ILMN_1683129	CCNL1	-0.0734	0.055695	0.129098
ILMN_2053178	ACTG1	-0.09489	0.028646	0.1235341
ILMN_1743747	RUSC1	-0.09338	-0.02846	0.1218336
ILMN_2357770	TCEA1	-0.08375	0.037871	0.1216191
ILMN_1743638	ABCG1	0.099589	-0.02049	0.1200812
ILMN_1690371	MRPL11	0.019061	0.088625	0.1076861

ILMN_1807535	YWHAE	-0.0921	0.002846	0.09494894
ILMN_1680937	HIST1H2BC	0.087653	0.005118	0.09277104
ILMN_2077130	MSH3	0.06585	0.025415	0.0912648
ILMN_1763437	CHIC1	0.021528	0.069317	0.0908442
ILMN_2065773	SCG5	0.049458	-0.03936	0.0888149
ILMN_3202396	LOC390735	-0.08478	0.002822	0.08760457
ILMN_1798485	ATP6V1E1	-0.01484	-0.07211	0.086948
ILMN_3197504	LOC100129026	0.017469	0.064109	0.0815785
ILMN_3282395	LOC646966	-0.0146	-0.06272	0.0773183
ILMN_3304130	LOC729236	0.036567	0.03986	0.0764264
ILMN_1708059	USP13	-0.04746	-0.02382	0.0712806
ILMN_1715684	LAMB3	-0.01102	-0.05483	0.0658495
ILMN_1720285	ESD	0.042057	0.017835	0.0598921
ILMN_1695370	LOC645968	-0.00632	0.051002	0.05732534
ILMN_1769673	ZNF655	-0.02852	-0.01564	0.0441643
ILMN_2382990	HK1	-0.00799	0.029711	0.03770538
ILMN_2245676	BTF3	0.023358	0.008947	0.03230443
ILMN_1755391	ARL2BP	0.005613	-0.01788	0.02349573
ILMN_1773026	VWA1	0.002191	-0.0013	0.00348747

PHAX	Target	Target	Target	ExpreSunitinib
ILMN_2190779___PHAX	ILMN	1688480	CCND1	4.81
ILMN_2190779___PHAX	ILMN	2074860	RN7SK	7.06
ILMN_2190779___PHAX	ILMN	3246805	LOC100134364	9.96
ILMN_2190779___PHAX	ILMN	3291472	LOC442727	5.92
ILMN_2190779___PHAX	ILMN	3243644	LOC100132564	8.72
ILMN_2190779___PHAX	ILMN	3234762	RN5S9	2.19
ILMN_2190779___PHAX	ILMN	3243593	LOC100008588	10.10
ILMN_2190779___PHAX	ILMN	3309453	RNU4-1	2.14
ILMN_2190779___PHAX	ILMN	3249578	LOC100132394	9.41
ILMN_2190779___PHAX	ILMN	2338452	SERPINA1	1.89
ILMN_2190779___PHAX	ILMN	1729408	LOC647357	4.92
ILMN_2190779___PHAX	ILMN	1699878	RAB27A	3.62
ILMN_2190779___PHAX	ILMN	1750130	GSPT1	3.74
ILMN_2190779___PHAX	ILMN	1786720	PROM1	1.80
ILMN_2190779___PHAX	ILMN	1781285	DUSP1	2.43
ILMN_2190779___PHAX	ILMN	1736178	AEBP1	1.40
ILMN_2190779___PHAX	ILMN	2360710	TPM1	5.03
ILMN_2190779___PHAX	ILMN	1697567	TPM3	2.28
ILMN_2190779___PHAX	ILMN	2148527	H19	2.12
ILMN_2190779___PHAX	ILMN	3236653	RNU1-5	1.79
ILMN_2190779___PHAX	ILMN	1716687	TPM1	4.86
ILMN_2190779___PHAX	ILMN	1676719	LOC644330	2.48
ILMN_2190779___PHAX	ILMN	1718034	LOC441454	4.29
ILMN_2190779___PHAX	ILMN	2155719	NBPF10	4.76
ILMN_2190779___PHAX	ILMN	1720829	ZFP36	1.80
ILMN_2190779___PHAX	ILMN	2362832	STAG3L1	6.81
ILMN_2190779___PHAX	ILMN	2149226	CAV1	2.32
ILMN_2190779___PHAX	ILMN	3308138	RNU4-2	1.77
ILMN_2190779___PHAX	ILMN	1798975	EGFR	1.53
ILMN_2190779___PHAX	ILMN	2157932	FKSG44	6.92
ILMN_2190779___PHAX	ILMN	3247242	LOC728687	7.67
ILMN_2190779___PHAX	ILMN	1733559	LOC100008589	9.98
ILMN_2190779___PHAX	ILMN	3251404	NUCKS1	4.13
ILMN_2190779___PHAX	ILMN	3244646	RNU1G2	1.64
ILMN_2190779___PHAX	ILMN	1710406	PSMC1	3.01
ILMN_2190779___PHAX	ILMN	1745214	LOC642393	5.03
ILMN_2190779___PHAX	ILMN	1660655	SUGT1P	6.37
ILMN_2190779___PHAX	ILMN	3249496	LOC653340	1.31
ILMN_2190779___PHAX	ILMN	3273946	CRCP	4.69
ILMN_2190779___PHAX	ILMN	1726437	EFCAB3	1.52
ILMN_2190779___PHAX	ILMN	1784141	JAKMIP1	2.96
ILMN_2190779___PHAX	ILMN	2110206	GNPTAB	7.60
ILMN_2190779___PHAX	ILMN	3251662	HINT3	1.74
ILMN_2190779___PHAX	ILMN	2391861	GSTM1	1.07
ILMN_2190779___PHAX	ILMN	3259146	LOC100129681	2.34
ILMN_2190779___PHAX	ILMN	1661599	DDIT4	2.41
ILMN_2190779___PHAX	ILMN	2252309	DPP7	1.46
ILMN_2190779___PHAX	ILMN	2150708	FLJ45966	7.07
ILMN_2190779___PHAX	ILMN	3246273	RNU1-3	1.38
ILMN_2190779___PHAX	ILMN	2378868	SFRS5	3.71
ILMN_2190779___PHAX	ILMN	1803988	MCL1	2.07

ILMN_2190779___PHAX	ILMN	3230608	NBPF8		2.57
ILMN_2190779___PHAX	ILMN	1690321	BCLAF1		2.34
ILMN_2190779___PHAX	ILMN	1803882	VEGFA		2.25
ILMN_2190779___PHAX	ILMN	2396875	IGFBP3		2.19
ILMN_2190779___PHAX	ILMN	3248663	SCARNA7		1.63
ILMN_2190779___PHAX	ILMN	3198090	LOC100132947		2.80
ILMN_2190779___PHAX	ILMN	2211065	TMEM91		1.78
ILMN_2190779___PHAX	ILMN	3232048	LOC100128405		5.41
ILMN_2190779___PHAX	ILMN	1343295	GAPDH		4.15
ILMN_2190779___PHAX	ILMN	3249707	LOC728649		1.37
ILMN_2190779___PHAX	ILMN	2175737	ZNF826		4.45
ILMN_2190779___PHAX	ILMN	2366463	FN1		2.82
ILMN_2190779___PHAX	ILMN	1778237	FN1		2.87
ILMN_2190779___PHAX	ILMN	1682402	SNORD46		4.51
ILMN_2190779___PHAX	ILMN	1735930	KLF2		1.42
ILMN_2190779___PHAX	ILMN	3249167	SNORA63		3.85
ILMN_2190779___PHAX	ILMN	1717990	CALD1		2.40
ILMN_2190779___PHAX	ILMN	2383934	ITGB1		2.57
ILMN_2190779___PHAX	ILMN	1768117	RBM25		2.00
ILMN_2190779___PHAX	ILMN	1660869	LOC643438		4.18
ILMN_2190779___PHAX	ILMN	3242688	GTF2H2D		3.86
ILMN_2190779___PHAX	ILMN	1762573	LOC401630		4.21
ILMN_2190779___PHAX	ILMN	1699829	CTGF		1.23
ILMN_2190779___PHAX	ILMN	3237826	FLJ37512		3.73
ILMN_2190779___PHAX	ILMN	3292224	LOC100131609		3.04
ILMN_2190779___PHAX	ILMN	2407168	S100A13		3.05
ILMN_2190779___PHAX	ILMN	2364022	SLC16A3		1.40
ILMN_2190779___PHAX	ILMN	2330552	CDC2L2		1.77
ILMN_2190779___PHAX	ILMN	1767556	C10orf10		1.73
ILMN_2190779___PHAX	ILMN	2201580	GSTM2		1.00
ILMN_2190779___PHAX	ILMN	2058841	LILRA6		4.18
ILMN_2190779___PHAX	ILMN	1659990	C7orf68		1.51
ILMN_2190779___PHAX	ILMN	1663866	TGFBI		1.65
ILMN_2190779___PHAX	ILMN	1810462	C10orf115		0.93
ILMN_2190779___PHAX	ILMN	1757552	PTRF		2.18
ILMN_2190779___PHAX	ILMN	1778668	TAGLN		2.11
ILMN_2190779___PHAX	ILMN	3246209	SCARNA10		1.14
ILMN_2190779___PHAX	ILMN	1665865	IGFBP4		1.56
ILMN_2190779___PHAX	ILMN	3308961	MIR1974		3.86
ILMN_2190779___PHAX	ILMN	1737141	LOC654163		3.42
ILMN_2190779___PHAX	ILMN	1764850	HPCAL1		3.01
ILMN_2190779___PHAX	ILMN	1730777	KRT19		1.08
ILMN_2190779___PHAX	ILMN	1782538	VIM		2.87
ILMN_2190779___PHAX	ILMN	1732988	KIAA1666		1.37
ILMN_2190779___PHAX	ILMN	1678143	ARHGDIB		1.94
ILMN_2190779___PHAX	ILMN	1805701	LOC647650		1.76
ILMN_2190779___PHAX	ILMN	3239969	LOC100133795		1.20
ILMN_2190779___PHAX	ILMN	1806023	JUN		3.10
ILMN_2190779___PHAX	ILMN	2190850	PPID		5.93
ILMN_2190779___PHAX	ILMN	2115490	NBPF20		2.10
ILMN_2190779___PHAX	ILMN	1805750	IFITM3		1.36
ILMN_2190779___PHAX	ILMN	2402131	TAF15		5.03

ILMN_2190779___PHAX	ILMN	1718977	GADD45B	1.09
ILMN_2190779___PHAX	ILMN	1761801	LOC147804	2.26
ILMN_2190779___PHAX	ILMN	1690138	PHF10	0.83
ILMN_2190779___PHAX	ILMN	3236408	SCARNA5	1.54
ILMN_2190779___PHAX	ILMN	1673352	IFITM2	1.34
ILMN_2190779___PHAX	ILMN	1660847	PFKFB3	1.40
ILMN_2190779___PHAX	ILMN	1701629	FLJ45684	3.02
ILMN_2190779___PHAX	ILMN	1746085	IGFBP3	1.75
ILMN_2190779___PHAX	ILMN	3220265	LOC100132553	1.62
ILMN_2190779___PHAX	ILMN	1756920	ADAM15	2.82
ILMN_2190779___PHAX	ILMN	1716876	MED27	2.77
ILMN_2190779___PHAX	ILMN	3200362	LOC727908	1.62
ILMN_2190779___PHAX	ILMN	2374865	ATF3	1.09
ILMN_2190779___PHAX	ILMN	2143795	MGC4677	2.15
ILMN_2190779___PHAX	ILMN	2265783	FHL2	3.94
ILMN_2190779___PHAX	ILMN	1789614	TPT1	1.55
ILMN_2190779___PHAX	ILMN	2366634	PKM2	3.22
ILMN_2190779___PHAX	ILMN	2122103	ETS1	2.42
ILMN_2190779___PHAX	ILMN	1652123	HMGN1	1.30
ILMN_2190779___PHAX	ILMN	2336609	SYTL2	1.34
ILMN_2190779___PHAX	ILMN	3245441	LOC100133042	2.31
ILMN_2190779___PHAX	ILMN	1651980	LOC283767	0.90
ILMN_2190779___PHAX	ILMN	2184869	ZMYM6	7.52
ILMN_2190779___PHAX	ILMN	3279092	LOC645691	2.26
ILMN_2190779___PHAX	ILMN	1698519	LOC642267	0.86
ILMN_2190779___PHAX	ILMN	2188264	CYR61	0.88
ILMN_2190779___PHAX	ILMN	2196328	POSTN	1.33
ILMN_2190779___PHAX	ILMN	3206827	LOC100131737	1.28
ILMN_2190779___PHAX	ILMN	1666192	DCTN5	1.21
ILMN_2190779___PHAX	ILMN	1653039	LOC642934	1.76
ILMN_2190779___PHAX	ILMN	1664922	FLNB	1.20
ILMN_2190779___PHAX	ILMN	1796629	EDNRA	1.65
ILMN_2190779___PHAX	ILMN	1684982	PDK4	1.47
ILMN_2190779___PHAX	ILMN	1711823	C17orf70	1.34
ILMN_2190779___PHAX	ILMN	1661903	CYLC2	4.32
ILMN_2190779___PHAX	ILMN	1758311	NET1	0.87
ILMN_2190779___PHAX	ILMN	1752798	LOC642780	0.82
ILMN_2190779___PHAX	ILMN	1686664	MT2A	1.15
ILMN_2190779___PHAX	ILMN	1691156	MT1A	2.11
ILMN_2190779___PHAX	ILMN	1712515	FOXO3	1.81
ILMN_2190779___PHAX	ILMN	2115125	CTGF	0.94
ILMN_2190779___PHAX	ILMN	1701308	COL1A1	1.93
ILMN_2190779___PHAX	ILMN	1723522	APOLD1	1.12
ILMN_2190779___PHAX	ILMN	1665510	ERRFI1	0.98
ILMN_2190779___PHAX	ILMN	2146418	CRIM1	1.11
ILMN_2190779___PHAX	ILMN	3247341	LOC100134739	1.61
ILMN_2190779___PHAX	ILMN	3224384	LOC729652	1.93
ILMN_2190779___PHAX	ILMN	1682775	EDN1	2.06
ILMN_2190779___PHAX	ILMN	3239108	SNORA3	0.94
ILMN_2190779___PHAX	ILMN	1782050	CEBPD	1.49
ILMN_2190779___PHAX	ILMN	1678968	GEM	0.83
ILMN_2190779___PHAX	ILMN	1762899	EGR1	2.57

ILMN_2190779___PHAX	ILMN	3248635	LOC100133744	1.52
ILMN_2190779___PHAX	ILMN	2389347	NR3C1	1.00
ILMN_2190779___PHAX	ILMN	1693107	MLH3	0.90
ILMN_2190779___PHAX	ILMN	1751034	ITPRIPL2	1.00
ILMN_2190779___PHAX	ILMN	1789965	LOC643313	0.76
ILMN_2190779___PHAX	ILMN	2395926	MANBAL	1.11
ILMN_2190779___PHAX	ILMN	1777296	ACTB	2.60
ILMN_2190779___PHAX	ILMN	1785379	ZNF8	0.87
ILMN_2190779___PHAX	ILMN	1794707	ATHL1	0.91
ILMN_2190779___PHAX	ILMN	3250257	ACVRL1	1.89
ILMN_2190779___PHAX	ILMN	1711617	GMFG	1.14
ILMN_2190779___PHAX	ILMN	1670926	CHST15	1.13
ILMN_2190779___PHAX	ILMN	1809957	AP2S1	2.14
ILMN_2190779___PHAX	ILMN	1768940	COL15A1	1.24
ILMN_2190779___PHAX	ILMN	2286574	EPHA3	3.92
ILMN_2190779___PHAX	ILMN	3230286	LOC731605	0.97
ILMN_2190779___PHAX	ILMN	2267535	NET1	0.73
ILMN_2190779___PHAX	ILMN	1676563	HTRA1	1.24
ILMN_2190779___PHAX	ILMN	1714861	CD68	2.31
ILMN_2190779___PHAX	ILMN	2161357	C6orf111	1.79
ILMN_2190779___PHAX	ILMN	3235559	CTGLF6	2.51
ILMN_2190779___PHAX	ILMN	2203858	OR10J1	0.57
ILMN_2190779___PHAX	ILMN	3281988	LOC728157	0.94
ILMN_2190779___PHAX	ILMN	2388975	CERK	0.66
ILMN_2190779___PHAX	ILMN	1723480	BST2	1.49
ILMN_2190779___PHAX	ILMN	3242833	LOC730375	0.78
ILMN_2190779___PHAX	ILMN	2133316	GIMAP7	0.85
ILMN_2190779___PHAX	ILMN	3310840	MIR21	1.29
ILMN_2190779___PHAX	ILMN	1702231	C1orf54	1.09
ILMN_2190779___PHAX	ILMN	3286809	LOC100131139	1.06
ILMN_2190779___PHAX	ILMN	3266894	LOC100128816	0.66
ILMN_2190779___PHAX	ILMN	2339835	PTGS1	0.88
ILMN_2190779___PHAX	ILMN	1687440	HIPK2	1.54
ILMN_2190779___PHAX	ILMN	1814971	TCF25	2.01
ILMN_2190779___PHAX	ILMN	2413158	PODXL	1.10
ILMN_2190779___PHAX	ILMN	1802205	RHOB	1.13
ILMN_2190779___PHAX	ILMN	1790761	POSTN	1.82
ILMN_2190779___PHAX	ILMN	2379762	NPM1	2.04
ILMN_2190779___PHAX	ILMN	3247960	LOC100133937	1.15
ILMN_2190779___PHAX	ILMN	1756992	MUC1	1.41
ILMN_2190779___PHAX	ILMN	1677314	MUC1	0.79
ILMN_2190779___PHAX	ILMN	3246247	LOC399491	0.53
ILMN_2190779___PHAX	ILMN	3247269	LOC400804	2.09
ILMN_2190779___PHAX	ILMN	1810431	LOC642299	1.62
ILMN_2190779___PHAX	ILMN	1802252	GAPDH	3.17
ILMN_2190779___PHAX	ILMN	1810910	CFH	1.05
ILMN_2190779___PHAX	ILMN	2173524	FOXD4	0.69
ILMN_2190779___PHAX	ILMN	1707727	ANGPTL4	3.00
ILMN_2190779___PHAX	ILMN	1749776	LOC642816	0.68
ILMN_2190779___PHAX	ILMN	1666276	LOC643451	0.67
ILMN_2190779___PHAX	ILMN	1669523	FOS	1.25
ILMN_2190779___PHAX	ILMN	1781769	CCRL1	1.13

ILMN_2190779___PHAX	ILMN	1806405	CDKN2AIPNL	1.42
ILMN_2190779___PHAX	ILMN	2398159	DKK3	0.84
ILMN_2190779___PHAX	ILMN	1770338	TM4SF1	2.11
ILMN_2190779___PHAX	ILMN	2393254	CAPNS1	1.26
ILMN_2190779___PHAX	ILMN	1783142	RPS4Y1	1.17
ILMN_2190779___PHAX	ILMN	1656920	CRIP1	0.79
ILMN_2190779___PHAX	ILMN	3246206	SNORA79	0.93
ILMN_2190779___PHAX	ILMN	1811117	LOC400986	1.19
ILMN_2190779___PHAX	ILMN	2079786	NUAK1	1.03
ILMN_2190779___PHAX	ILMN	1671925	SDHAP3	1.33
ILMN_2190779___PHAX	ILMN	2038778	GAPDH	3.54
ILMN_2190779___PHAX	ILMN	1801616	EMP1	0.64
ILMN_2190779___PHAX	ILMN	1659462	DUSP23	1.13
ILMN_2190779___PHAX	ILMN	1729453	TSPAN9	1.37
ILMN_2190779___PHAX	ILMN	2063114	TAF1D	0.69
ILMN_2190779___PHAX	ILMN	1704446	SLC6A10P	1.10
ILMN_2190779___PHAX	ILMN	3241798	SNORA41	0.72
ILMN_2190779___PHAX	ILMN	3217522	LOC339970	2.72
ILMN_2190779___PHAX	ILMN	1769191	GNAS	4.05
ILMN_2190779___PHAX	ILMN	1756071	MFGE8	1.49
ILMN_2190779___PHAX	ILMN	2058251	VIM	3.22
ILMN_2190779___PHAX	ILMN	1665909	LASP1	2.39
ILMN_2190779___PHAX	ILMN	1713156	MSL3L1	0.87
ILMN_2190779___PHAX	ILMN	1773079	COL3A1	0.88
ILMN_2190779___PHAX	ILMN	1733116	UBXD7	1.34
ILMN_2190779___PHAX	ILMN	2204876	FLVCR2	0.65
ILMN_2190779___PHAX	ILMN	1764266	CKMT2	0.83
ILMN_2190779___PHAX	ILMN	1653687	GALNT9	0.82
ILMN_2190779___PHAX	ILMN	2082273	RGS5	1.24
ILMN_2190779___PHAX	ILMN	3238837	MSTO2P	5.65
ILMN_2190779___PHAX	ILMN	1742866	F2R	0.66
ILMN_2190779___PHAX	ILMN	1651554	RGS5	1.69
ILMN_2190779___PHAX	ILMN	1773154	NFKBIA	0.88
ILMN_2190779___PHAX	ILMN	1722872	MYH9	2.31
ILMN_2190779___PHAX	ILMN	1745697	MGRN1	0.83
ILMN_2190779___PHAX	ILMN	1815673	DKK3	0.82
ILMN_2190779___PHAX	ILMN	2128750	PTTG1IP	1.91
ILMN_2190779___PHAX	ILMN	1802459	LOC643138	0.85
ILMN_2190779___PHAX	ILMN	1664675	ARMCX3	0.66
ILMN_2190779___PHAX	ILMN	1761058	ACAD11	1.10
ILMN_2190779___PHAX	ILMN	1788062	SH3GL1	0.79
ILMN_2190779___PHAX	ILMN	2412192	CFH	0.97
ILMN_2190779___PHAX	ILMN	1805345	MOSPD3	0.85
ILMN_2190779___PHAX	ILMN	1761281	LOC441019	0.71
ILMN_2190779___PHAX	ILMN	2241825	LDB3	0.64
ILMN_2190779___PHAX	ILMN	1712031	LOC653629	0.59
ILMN_2190779___PHAX	ILMN	1696394	IL6R	1.14
ILMN_2190779___PHAX	ILMN	2071809	MGP	1.39
ILMN_2190779___PHAX	ILMN	1665515	MGC4677	0.53
ILMN_2190779___PHAX	ILMN	2093027	MYO1B	1.50
ILMN_2190779___PHAX	ILMN	1703330	FEM1C	0.80
ILMN_2190779___PHAX	ILMN	2086105	SPRY4	0.93

ILMN_2190779___PHAX	ILMN	1801999	LOC644474		2.37
ILMN_2190779___PHAX	ILMN	1800697	LDB2		1.10
ILMN_2190779___PHAX	ILMN	1792860	MED22		0.58
ILMN_2190779___PHAX	ILMN	2273700	ZNF706		5.31
ILMN_2190779___PHAX	ILMN	2183891	PEX5L		0.59
ILMN_2190779___PHAX	ILMN	1683096	ASB1		1.24
ILMN_2190779___PHAX	ILMN	1676449	SLIT2		0.98
ILMN_2190779___PHAX	ILMN	2086077	JUNB		0.95
ILMN_2190779___PHAX	ILMN	2135898	GPR42		0.68
ILMN_2190779___PHAX	ILMN	1706643	COL6A3		1.60
ILMN_2190779___PHAX	ILMN	1853824	MGAT3		1.08
ILMN_2190779___PHAX	ILMN	1757052	SLC16A1		0.76
ILMN_2190779___PHAX	ILMN	2169261	TNNI2		0.44
ILMN_2190779___PHAX	ILMN	3240150	SNORA75		0.96
ILMN_2190779___PHAX	ILMN	3278745	LOC253482		1.87
ILMN_2190779___PHAX	ILMN	1776157		Sep-04	0.91
ILMN_2190779___PHAX	ILMN	1704730	CD93		0.65
ILMN_2190779___PHAX	ILMN	1680692	NUCKS1		0.65
ILMN_2190779___PHAX	ILMN	3179762	LOC100130483		1.12
ILMN_2190779___PHAX	ILMN	1675130	NFIC		0.64
ILMN_2190779___PHAX	ILMN	1656940	ABLIM3		0.96
ILMN_2190779___PHAX	ILMN	2332368	VPS13B		0.93
ILMN_2190779___PHAX	ILMN	2395913	ARHGAP11A		0.49
ILMN_2190779___PHAX	ILMN	1747730	NOTCH2NL		0.71
ILMN_2190779___PHAX	ILMN	1658044	LOC648814		0.98
ILMN_2190779___PHAX	ILMN	3241996	C6orf59		0.68
ILMN_2190779___PHAX	ILMN	3248828	LOC100133758		0.54
ILMN_2190779___PHAX	ILMN	1686388	LOC644330		0.68
ILMN_2190779___PHAX	ILMN	2191331	RPS4Y2		0.66
ILMN_2190779___PHAX	ILMN	1797596	SPRY4		0.94
ILMN_2190779___PHAX	ILMN	1654268	HMGB2		0.64
ILMN_2190779___PHAX	ILMN	1691860	SPRY1		0.78
ILMN_2190779___PHAX	ILMN	1712231	SH3YL1		0.90
ILMN_2190779___PHAX	ILMN	3217610	LOC645181		0.80
ILMN_2190779___PHAX	ILMN	1706505	COL5A1		1.13
ILMN_2190779___PHAX	ILMN	1658926	NOTCH3		1.00
ILMN_2190779___PHAX	ILMN	3264100	LOC100130564		0.76
ILMN_2190779___PHAX	ILMN	2386444	ANGPTL4		1.39
ILMN_2190779___PHAX	ILMN	1740441	CYB5R3		0.86
ILMN_2190779___PHAX	ILMN	3230572	NCRNA00152		0.73
ILMN_2190779___PHAX	ILMN	1737576	RASA1		0.94
ILMN_2190779___PHAX	ILMN	1693293	LOC648176		0.76
ILMN_2190779___PHAX	ILMN	2150654	ZSWIM4		1.21
ILMN_2190779___PHAX	ILMN	1653283	APP		2.02
ILMN_2190779___PHAX	ILMN	3259283	LOC100128747		0.66
ILMN_2190779___PHAX	ILMN	2375003	MAP4K4		0.75
ILMN_2190779___PHAX	ILMN	1714449	OR5D18		0.62
ILMN_2190779___PHAX	ILMN	3235647	SIK1		0.71
ILMN_2190779___PHAX	ILMN	2286334	SR140		0.66
ILMN_2190779___PHAX	ILMN	1663836	LOC648374		0.73
ILMN_2190779___PHAX	ILMN	1733985	SIRT3		2.99
ILMN_2190779___PHAX	ILMN	1707175	NSD1		0.72

ILMN_2190779___PHAX	ILMN	1720249	LOC648057		0.64
ILMN_2190779___PHAX	ILMN	2082762	SNORD68		0.78
ILMN_2190779___PHAX	ILMN	1759460	TAF7		0.62
ILMN_2190779___PHAX	ILMN	1735014	KLF6		1.19
ILMN_2190779___PHAX	ILMN	2253286	PRKCZ		0.53
ILMN_2190779___PHAX	ILMN	2261784	CCNY		0.59
ILMN_2190779___PHAX	ILMN	3211047	LOC646201		0.90
ILMN_2190779___PHAX	ILMN	1664912	IL11RA		0.82
ILMN_2190779___PHAX	ILMN	1779875	THY1		1.14
ILMN_2190779___PHAX	ILMN	1654998	SMEK1		0.68
ILMN_2190779___PHAX	ILMN	1774513	DDX17		2.27
ILMN_2190779___PHAX	ILMN	1714412	ZNF292		0.52
ILMN_2190779___PHAX	ILMN	3240155	RNU105A		0.68
ILMN_2190779___PHAX	ILMN	1704424	PLA2R1		0.52
ILMN_2190779___PHAX	ILMN	1801456	CROP		0.62
ILMN_2190779___PHAX	ILMN	1693983	MUC5B		0.73
ILMN_2190779___PHAX	ILMN	1734553	LOC653234		0.84
ILMN_2190779___PHAX	ILMN	2173611	MT1E		1.01
ILMN_2190779___PHAX	ILMN	1784661	TMEM2		1.51
ILMN_2190779___PHAX	ILMN	1806576	LOC651137		0.57
ILMN_2190779___PHAX	ILMN	1684391	PLOD1		0.88
ILMN_2190779___PHAX	ILMN	1807042	MARCKS		1.00
ILMN_2190779___PHAX	ILMN	1779677	ZCCHC6		0.85
ILMN_2190779___PHAX	ILMN	1803213	MXRA5		1.10
ILMN_2190779___PHAX	ILMN	1813669	ANKS1A		0.65
ILMN_2190779___PHAX	ILMN	1697469	SFRS6		3.19
ILMN_2190779___PHAX	ILMN	1717639	SIK1		0.70
ILMN_2190779___PHAX	ILMN	3251332	POM121L10P		0.69
ILMN_2190779___PHAX	ILMN	1805842	FHL1		0.76
ILMN_2190779___PHAX	ILMN	1766499	HSPA2		0.76
ILMN_2190779___PHAX	ILMN	1698144	CFH		0.49
ILMN_2190779___PHAX	ILMN	1782543	EEF1D		0.41
ILMN_2190779___PHAX	ILMN	3205781	LOC646316		4.60
ILMN_2190779___PHAX	ILMN	3205656	LOC391075		1.94
ILMN_2190779___PHAX	ILMN	2213136	LEF1		0.47
ILMN_2190779___PHAX	ILMN	2367215	PRCP		1.36
ILMN_2190779___PHAX	ILMN	1768973	HIST2H2AC		0.65
ILMN_2190779___PHAX	ILMN	1760922	GIT2		0.99
ILMN_2190779___PHAX	ILMN	1797236	TGM2		1.09
ILMN_2190779___PHAX	ILMN	3248712	SNORD67		1.08
ILMN_2190779___PHAX	ILMN	1759670	AMACR		0.92
ILMN_2190779___PHAX	ILMN	1785402	LTBP1		0.76
ILMN_2190779___PHAX	ILMN	3295109	LOC653557		0.54
ILMN_2190779___PHAX	ILMN	1723978	LGALS1		1.15
ILMN_2190779___PHAX	ILMN	1776480	HINFP		1.50
ILMN_2190779___PHAX	ILMN	1679754	ADRA1B		1.19
ILMN_2190779___PHAX	ILMN	1778377	ERGIC1		0.90
ILMN_2190779___PHAX	ILMN	1658978	LOC650155		0.86
ILMN_2190779___PHAX	ILMN	1689518	PECAM1		0.73
ILMN_2190779___PHAX	ILMN	1785272	COL1A2		1.09
ILMN_2190779___PHAX	ILMN	1680738	C5orf13		0.73
ILMN_2190779___PHAX	ILMN	2389876	TGFB1I1		0.71

ILMN_2190779___PHAX	ILMN	1770281	ERC1		0.57
ILMN_2190779___PHAX	ILMN	1778128	LOC642780		3.14
ILMN_2190779___PHAX	ILMN	2379788	HIF1A		0.63
ILMN_2190779___PHAX	ILMN	2107184	SNRK		1.15
ILMN_2190779___PHAX	ILMN	3299407	LOC728553		0.61
ILMN_2190779___PHAX	ILMN	3308718	MIR1826		1.03
ILMN_2190779___PHAX	ILMN	1772876	ZNF395		0.69
ILMN_2190779___PHAX	ILMN	1756806	MCL1		0.63
ILMN_2190779___PHAX	ILMN	1723300	FAM70B		0.64
ILMN_2190779___PHAX	ILMN	2287707	POT1		3.94
ILMN_2190779___PHAX	ILMN	2203876	CCDC68		3.49
ILMN_2190779___PHAX	ILMN	2394264	UBTF		0.96
ILMN_2190779___PHAX	ILMN	1745607	A2M		2.11
ILMN_2190779___PHAX	ILMN	1736834	SKIL		0.94
ILMN_2190779___PHAX	ILMN	3245616	CWC22		0.60
ILMN_2190779___PHAX	ILMN	2062468	IGFBP7		1.67
ILMN_2190779___PHAX	ILMN	2087702	MYH9		1.31
ILMN_2190779___PHAX	ILMN	3245116	GOLIM4		1.60
ILMN_2190779___PHAX	ILMN	1698067	NBPF14		0.98
ILMN_2190779___PHAX	ILMN	1789196	TPM2		1.50
ILMN_2190779___PHAX	ILMN	1680070	MUC6		0.66
ILMN_2190779___PHAX	ILMN	1807710	HINT1		1.01
ILMN_2190779___PHAX	ILMN	2184184	ANXA1		0.73
ILMN_2190779___PHAX	ILMN	1806320	PPFIBP1		0.95
ILMN_2190779___PHAX	ILMN	2115862	ESPNL		0.55
ILMN_2190779___PHAX	ILMN	3239785	LOC100134304		1.02
ILMN_2190779___PHAX	ILMN	1675249	SGTB		1.22
ILMN_2190779___PHAX	ILMN	1671516	CIR1		0.59
ILMN_2190779___PHAX	ILMN	1675519	LOC644611		0.66
ILMN_2190779___PHAX	ILMN	1652306	MEGF10		0.55
ILMN_2190779___PHAX	ILMN	3238018	LOC100134372		2.19
ILMN_2190779___PHAX	ILMN	1666894	CSPG4		1.17
ILMN_2190779___PHAX	ILMN	2142185	CLEC14A		0.69
ILMN_2190779___PHAX	ILMN	1751607	FOSB		1.49
ILMN_2190779___PHAX	ILMN	1680344	MYOM1		0.97
ILMN_2190779___PHAX	ILMN	2136089	MTE		0.81
ILMN_2190779___PHAX	ILMN	1689976	EDIL3		0.53
ILMN_2190779___PHAX	ILMN	1692249	LOC641750		1.30
ILMN_2190779___PHAX	ILMN	2224300	SNAPC3		2.89
ILMN_2190779___PHAX	ILMN	2056606	PPP1R1A		1.22
ILMN_2190779___PHAX	ILMN	1696757	TTC14		0.65
ILMN_2190779___PHAX	ILMN	2384857	DHRS2		0.60
ILMN_2190779___PHAX	ILMN	1713163	SMARCA5		0.56
ILMN_2190779___PHAX	ILMN	2167805	LUM		0.62
ILMN_2190779___PHAX	ILMN	1761083	HNRNPA3		1.21
ILMN_2190779___PHAX	ILMN	2190942	FIP1L1		0.48
ILMN_2190779___PHAX	ILMN	1781560	ST3GAL6		0.58
ILMN_2190779___PHAX	ILMN	1662879	UBE2MP1		0.59
ILMN_2190779___PHAX	ILMN	2412624	TP53I11		0.58
ILMN_2190779___PHAX	ILMN	1742547	NRP1		0.59
ILMN_2190779___PHAX	ILMN	1787212	CDKN1A		0.51
ILMN_2190779___PHAX	ILMN	2393450	C14orf173		1.16

ILMN_2190779___PHAX	ILMN	3195198	KRT17P3	0.68
ILMN_2190779___PHAX	ILMN	1739821	EIF2S1	0.59
ILMN_2190779___PHAX	ILMN	1685870	HYDIN	0.64
ILMN_2190779___PHAX	ILMN	2355423	TPO	0.42
ILMN_2190779___PHAX	ILMN	1796734	SPARC	0.72
ILMN_2190779___PHAX	ILMN	2350634	EFEMP1	1.52
ILMN_2190779___PHAX	ILMN	1791057	IFNAR2	0.85
ILMN_2190779___PHAX	ILMN	2063586	CLIC4	0.84
ILMN_2190779___PHAX	ILMN	1792014	FAM70B	0.55
ILMN_2190779___PHAX	ILMN	2132982	IGFBP5	1.31
ILMN_2190779___PHAX	ILMN	1801077	PLIN2	1.06
ILMN_2190779___PHAX	ILMN	1744487	C1QTNF5	1.13
ILMN_2190779___PHAX	ILMN	1739161	PPAP2A	1.17
ILMN_2190779___PHAX	ILMN	1703123	AXUD1	0.58
ILMN_2190779___PHAX	ILMN	1761946	PROM2	1.42
ILMN_2190779___PHAX	ILMN	3251312	TMEM69	2.30
ILMN_2190779___PHAX	ILMN	1811921	CSRP1	0.62
ILMN_2190779___PHAX	ILMN	3237623	RNY1	1.76
ILMN_2190779___PHAX	ILMN	3235264	SNORA48	3.30
ILMN_2190779___PHAX	ILMN	1805737	PFKP	0.96
ILMN_2190779___PHAX	ILMN	1651285	BCL6B	1.28
ILMN_2190779___PHAX	ILMN	1652604	GGT1	0.90
ILMN_2190779___PHAX	ILMN	1787648	GPR125	0.91
ILMN_2190779___PHAX	ILMN	2104356	COL1A2	0.97
ILMN_2190779___PHAX	ILMN	1667791	PPFIA4	0.68
ILMN_2190779___PHAX	ILMN	1741356	PRICKLE1	0.65
ILMN_2190779___PHAX	ILMN	2206746	BGN	3.11
ILMN_2190779___PHAX	ILMN	3197504	LOC100129026	0.49
ILMN_2190779___PHAX	ILMN	1719828	LOC653052	1.30
ILMN_2190779___PHAX	ILMN	3239445	ZBTB42	0.55
ILMN_2190779___PHAX	ILMN	1709650	LOC652815	0.55
ILMN_2190779___PHAX	ILMN	1651787	MSH5	1.05
ILMN_2190779___PHAX	ILMN	2165369	HIST1H4B	0.39
ILMN_2190779___PHAX	ILMN	3219534	LOC100131504	0.58
ILMN_2190779___PHAX	ILMN	1708296	DEAF1	3.90
ILMN_2190779___PHAX	ILMN	1730698	ODF2	0.55
ILMN_2190779___PHAX	ILMN	2393765	IGLL1	1.49
ILMN_2190779___PHAX	ILMN	3250927	EIF4E	5.57
ILMN_2190779___PHAX	ILMN	2397024	SPOP	1.47
ILMN_2190779___PHAX	ILMN	2355225	LSP1	0.66
ILMN_2190779___PHAX	ILMN	1756573	NDUFA4L2	1.21
ILMN_2190779___PHAX	ILMN	1701857	LOC644373	0.65
ILMN_2190779___PHAX	ILMN	2052208	GADD45A	0.57
ILMN_2190779___PHAX	ILMN	2096719	GRK5	0.70
ILMN_2190779___PHAX	ILMN	1757604	TPM2	2.02
ILMN_2190779___PHAX	ILMN	1738598	LOC651483	7.47
ILMN_2190779___PHAX	ILMN	1800164	PPFIA1	0.54
ILMN_2190779___PHAX	ILMN	1725247	GTF2A1	0.52
ILMN_2190779___PHAX	ILMN	1807945	ANP32A	0.56
ILMN_2190779___PHAX	ILMN	1796099	LOC644380	2.53
ILMN_2190779___PHAX	ILMN	1674250	NCKAP1L	1.00
ILMN_2190779___PHAX	ILMN	1670379	ANTXR1	1.00

ILMN_2190779___PHAX	ILMN	1657529	CYP2B6	3.20
ILMN_2190779___PHAX	ILMN	1660806	CSRP2	0.90
ILMN_2190779___PHAX	ILMN	1788468	ARGLU1	0.63
ILMN_2190779___PHAX	ILMN	1746243	TES	0.79
ILMN_2190779___PHAX	ILMN	1791466	HMGB1	3.17
ILMN_2190779___PHAX	ILMN	2216157	GNA12	0.62
ILMN_2190779___PHAX	ILMN	1708130	EHF	0.73
ILMN_2190779___PHAX	ILMN	1708130	EHF	0.73
ILMN_2190779___PHAX	ILMN	1728445	IGFBP1	0.51
ILMN_2190779___PHAX	ILMN	1723874	MRPS6	1.06
ILMN_2190779___PHAX	ILMN	1794534	CCDC81	0.65
ILMN_2190779___PHAX	ILMN	1677404	RAP2A	1.10
ILMN_2190779___PHAX	ILMN	1787931	LOC389517	0.49
ILMN_2190779___PHAX	ILMN	1805807	SLC30A3	0.43
ILMN_2190779___PHAX	ILMN	3244515	LOC100134009	0.55
ILMN_2190779___PHAX	ILMN	1734276	PMEPA1	0.96
ILMN_2190779___PHAX	ILMN	1698117	DSCR8	1.21
ILMN_2190779___PHAX	ILMN	3243782	LOC100133939	0.68
ILMN_2190779___PHAX	ILMN	1807372	ADORA2A	0.56
ILMN_2190779___PHAX	ILMN	1764090	AK3L1	0.88
ILMN_2190779___PHAX	ILMN	1780894	ANTXR1	0.51
ILMN_2190779___PHAX	ILMN	1759861	LOC646653	0.70
ILMN_2190779___PHAX	ILMN	1810922	PCNT	0.53
ILMN_2190779___PHAX	ILMN	2137789	KLF4	0.42
ILMN_2190779___PHAX	ILMN	1671337	SLC2A5	0.75
ILMN_2190779___PHAX	ILMN	1750352	C14orf78	0.82
ILMN_2190779___PHAX	ILMN	1754410	C12orf72	0.81
ILMN_2190779___PHAX	ILMN	3245507	LOC100133627	0.72
ILMN_2190779___PHAX	ILMN	1739423	RN7SK	0.54
ILMN_2190779___PHAX	ILMN	2211800	HMGB1L1	1.33
ILMN_2190779___PHAX	ILMN	3236942	SNORA5A	1.36
ILMN_2190779___PHAX	ILMN	2387078	MPZL2	0.63
ILMN_2190779___PHAX	ILMN	1692169	NAT5	0.35
ILMN_2190779___PHAX	ILMN	1663321	LOC644781	0.68
ILMN_2190779___PHAX	ILMN	2341229	CD34	0.73
ILMN_2190779___PHAX	ILMN	1655930	ELL2	0.48
ILMN_2190779___PHAX	ILMN	1714880	LIMS3	0.39
ILMN_2190779___PHAX	ILMN	1664176	FBLN5	0.77
ILMN_2190779___PHAX	ILMN	3191393	LOC100128892	1.06
ILMN_2190779___PHAX	ILMN	1775257	PROK2	0.76
ILMN_2190779___PHAX	ILMN	1730622	EVL	0.81
ILMN_2190779___PHAX	ILMN	2324002	CALD1	1.22
ILMN_2190779___PHAX	ILMN	1666269	CTSZ	0.83
ILMN_2190779___PHAX	ILMN	1747119	FBXO46	0.57
ILMN_2190779___PHAX	ILMN	1698732	PALLD	0.69
ILMN_2190779___PHAX	ILMN	3245282	LOC100192426	0.80
ILMN_2190779___PHAX	ILMN	1682717	IER3	0.78
ILMN_2190779___PHAX	ILMN	1778010	IL32	0.59
ILMN_2190779___PHAX	ILMN	1780058	DEGS1	1.22
ILMN_2190779___PHAX	ILMN	1815057	PDGFRB	0.73
ILMN_2190779___PHAX	ILMN	1792495	AHNAK	0.68
ILMN_2190779___PHAX	ILMN	1653496	GLUL	0.73

ILMN_2190779___PHAX	ILMN	1795442	LAMA4	0.92
ILMN_2190779___PHAX	ILMN	3224126	LOC729222	0.71
ILMN_2190779___PHAX	ILMN	1804248	FDPS	0.72
ILMN_2190779___PHAX	ILMN	3240022	SNORA73A	0.57
ILMN_2190779___PHAX	ILMN	1730007	MPZL2	0.76
ILMN_2190779___PHAX	ILMN	2307861	COL6A3	0.86
ILMN_2190779___PHAX	ILMN	1807611	PPIG	1.11
ILMN_2190779___PHAX	ILMN	1803788	LGALS3	0.87
ILMN_2190779___PHAX	ILMN	2395496	KLK7	3.92
ILMN_2190779___PHAX	ILMN	1683859	SLC7A1	0.63
ILMN_2190779___PHAX	ILMN	1653028	COL4A1	2.70
ILMN_2190779___PHAX	ILMN	2355004	HMGN3	0.61
ILMN_2190779___PHAX	ILMN	1654324	HEYL	1.45
ILMN_2190779___PHAX	ILMN	3265797	LOC100130561	0.52
ILMN_2190779___PHAX	ILMN	2309156	PMEPA1	1.66
ILMN_2190779___PHAX	ILMN	2315979	LBH	0.84
ILMN_2190779___PHAX	ILMN	1711566	TIMP1	1.71
ILMN_2190779___PHAX	ILMN	1714700	TRIB2	0.54
ILMN_2190779___PHAX	ILMN	1809439	HMGB1L1	0.42
ILMN_2190779___PHAX	ILMN	1791576	CHSY1	0.92
ILMN_2190779___PHAX	ILMN	1803312	DIMT1L	0.60
ILMN_2190779___PHAX	ILMN	3243813	LOC727914	0.52
ILMN_2190779___PHAX	ILMN	1726547	MAP3K5	1.08
ILMN_2190779___PHAX	ILMN	1731374	CPE	0.74
ILMN_2190779___PHAX	ILMN	2353202	PTK7	0.53
ILMN_2190779___PHAX	ILMN	2237428	SCD5	0.54
ILMN_2190779___PHAX	ILMN	1732575	SEC14L1	0.83
ILMN_2190779___PHAX	ILMN	1719599	SYTL4	0.63
ILMN_2190779___PHAX	ILMN	2259119	PRMT2	1.08
ILMN_2190779___PHAX	ILMN	3241234	LOC730278	0.53
ILMN_2190779___PHAX	ILMN	1804444	PDIA2	0.61
ILMN_2190779___PHAX	ILMN	2411282	QSOX1	0.62
ILMN_2190779___PHAX	ILMN	3195815	LOC100130561	0.62
ILMN_2190779___PHAX	ILMN	2338997	PTP4A2	0.99
ILMN_2190779___PHAX	ILMN	1803429	CD44	0.84
ILMN_2190779___PHAX	ILMN	1794740	CD151	0.56
ILMN_2190779___PHAX	ILMN	1727248	INF2	0.75
ILMN_2190779___PHAX	ILMN	3283772	LOC644237	0.68
ILMN_2190779___PHAX	ILMN	1687315	RXRA	0.52
ILMN_2190779___PHAX	ILMN	1708064	MAP4	0.57
ILMN_2190779___PHAX	ILMN	2342695	PDGFA	0.69
ILMN_2190779___PHAX	ILMN	2129161	LRRC32	0.69
ILMN_2190779___PHAX	ILMN	1781514	PCDH17	1.20
ILMN_2190779___PHAX	ILMN	1806733	COL18A1	1.12
ILMN_2190779___PHAX	ILMN	1671703	ACTA2	2.12
ILMN_2190779___PHAX	ILMN	3238283	LOC100130123	0.73
ILMN_2190779___PHAX	ILMN	1796126	MXRA7	0.72
ILMN_2190779___PHAX	ILMN	1686116	THBS1	0.83
ILMN_2190779___PHAX	ILMN	1731353	CHPF	0.72
ILMN_2190779___PHAX	ILMN	2262044	PARP10	0.96
ILMN_2190779___PHAX	ILMN	1767448	LHFP	1.06
ILMN_2190779___PHAX	ILMN	1795429	VCL	0.65

ILMN_2190779___PHAX	ILMN	3246713	SNORA22		0.77
ILMN_2190779___PHAX	ILMN	1752755	VWF		1.00
ILMN_2190779___PHAX	ILMN	1724718	NCK2		1.39
ILMN_2190779___PHAX	ILMN	1676629	INSIG2		1.04
ILMN_2190779___PHAX	ILMN	1726589	CD248		0.80
ILMN_2190779___PHAX	ILMN	1693060	VEGFA		0.51
ILMN_2190779___PHAX	ILMN	2292592	PCSK6		0.65
ILMN_2190779___PHAX	ILMN	3243677	SNORA73B		0.67
ILMN_2190779___PHAX	ILMN	2374425	CCNE1		0.49
ILMN_2190779___PHAX	ILMN	1711078	CDC2L2		0.58
ILMN_2190779___PHAX	ILMN	1731984	HMGN3		0.60
ILMN_2190779___PHAX	ILMN	3270378	LOC100127982		0.38
ILMN_2190779___PHAX	ILMN	1760338	LOC643357		1.11
ILMN_2190779___PHAX	ILMN	2394305	PDGFB		0.45
ILMN_2190779___PHAX	ILMN	2313901	PAM		0.65
ILMN_2190779___PHAX	ILMN	1802251	PTTG1IP		0.62
ILMN_2190779___PHAX	ILMN	1769575	JAM3		0.59
ILMN_2190779___PHAX	ILMN	1728019	PML		0.77
ILMN_2190779___PHAX	ILMN	1725750	LOC644695		0.48
ILMN_2190779___PHAX	ILMN	1740426	RASD1		1.18
ILMN_2190779___PHAX	ILMN	2149782	RAMP2		0.56
ILMN_2190779___PHAX	ILMN	1775466	RPP38		0.53
ILMN_2190779___PHAX	ILMN	3270833	LOC100129547		0.87
ILMN_2190779___PHAX	ILMN	3220952	LOC730658		1.24
ILMN_2190779___PHAX	ILMN	1694878	LOC644979		0.52
ILMN_2190779___PHAX	ILMN	1712755	LRRC41		0.85
ILMN_2190779___PHAX	ILMN	3244963	WDR42A		0.88
ILMN_2190779___PHAX	ILMN	2104141	FGD5		0.96
ILMN_2190779___PHAX	ILMN	1682636	CXCL2		0.39
ILMN_2190779___PHAX	ILMN	1804357	GNG4		0.32
ILMN_2190779___PHAX	ILMN	1689318	NUAK1		1.10
ILMN_2190779___PHAX	ILMN	1752932	MPZL2		0.98
ILMN_2190779___PHAX	ILMN	1704753	EPAS1		1.41
ILMN_2190779___PHAX	ILMN	1685275	MCAM		0.91
ILMN_2190779___PHAX	ILMN	3237946	LOC100134134		1.19
ILMN_2190779___PHAX	ILMN	1710598	SKI		0.74
ILMN_2190779___PHAX	ILMN	1733851	DACT3		0.95
ILMN_2190779___PHAX	ILMN	1737163	SH3BGRL3		0.71
ILMN_2190779___PHAX	ILMN	2056032	CD99		0.69
ILMN_2190779___PHAX	ILMN	3246424	LOC100133600		0.65
ILMN_2190779___PHAX	ILMN	2396020	DUSP6		0.77
ILMN_2190779___PHAX	ILMN	1740430	SLC2A4RG		0.59
ILMN_2190779___PHAX	ILMN	1784300	TUBA4A		2.70
ILMN_2190779___PHAX	ILMN	1685625	UCP2		0.86
ILMN_2190779___PHAX	ILMN	2165753	HLA-A29.1		0.88
ILMN_2190779___PHAX	ILMN	2147133	NBPF15		1.13
ILMN_2190779___PHAX	ILMN	1811786	CCDC102B		0.56
ILMN_2190779___PHAX	ILMN	1741688	CPXM2		0.20
ILMN_2190779___PHAX	ILMN	2347145	DCN		0.68
ILMN_2190779___PHAX	ILMN	3238662	SNORD89		3.05
ILMN_2190779___PHAX	ILMN	3243069	LOC100133692		0.64
ILMN_2190779___PHAX	ILMN	1704477	COX5A		0.39

ILMN_2190779___PHAX	ILMN	1768534	BHLHB2		0.51
ILMN_2190779___PHAX	ILMN	3227912	LOC728576		2.38
ILMN_2190779___PHAX	ILMN	1743911	SLC25A39		0.30
ILMN_2190779___PHAX	ILMN	1730611	RTN4		1.68
ILMN_2190779___PHAX	ILMN	1745820	RASSF6		0.62
ILMN_2190779___PHAX	ILMN	2184049	COX7B		0.21
ILMN_2190779___PHAX	ILMN	1678730	NOMO1		0.40
ILMN_2190779___PHAX	ILMN	1758705	IRX6		0.45
ILMN_2190779___PHAX	ILMN	1695317	RCBTB1		0.31
ILMN_2190779___PHAX	ILMN	1753182	COL20A1		0.45
ILMN_2190779___PHAX	ILMN	1755926	DBI		0.24
ILMN_2190779___PHAX	ILMN	1659782	STK19		0.18
ILMN_2190779___PHAX	ILMN	1741133	NME1		0.88
ILMN_2190779___PHAX	ILMN	1773262	ESM1		1.77
ILMN_2190779___PHAX	ILMN	3217771	LOC100133154		2.60
ILMN_2190779___PHAX	ILMN	1656145	GOT1		0.57
ILMN_2190779___PHAX	ILMN	1676393	ATP5G1		0.23
ILMN_2190779___PHAX	ILMN	1711516	ATP6V1A		1.08
ILMN_2190779___PHAX	ILMN	2407389	GPNMB		1.36
ILMN_2190779___PHAX	ILMN	1694731	CLCN7		0.35
ILMN_2190779___PHAX	ILMN	2223903	PPIC		0.73
ILMN_2190779___PHAX	ILMN	2129572	F3		0.59
ILMN_2190779___PHAX	ILMN	1744381	SERPINE1		1.12
ILMN_2190779___PHAX	ILMN	1744381	SERPINE1		1.12
ILMN_2190779___PHAX	ILMN	1727194	CALU		0.36
ILMN_2190779___PHAX	ILMN	1721876	TIMP2		0.63
ILMN_2190779___PHAX	ILMN	3291344	LOC202227		1.06
ILMN_2190779___PHAX	ILMN	2364072	CLCNKA		0.63
ILMN_2190779___PHAX	ILMN	1804601	LOC649923		1.59
ILMN_2190779___PHAX	ILMN	2212878	ESM1		1.39
ILMN_2190779___PHAX	ILMN	1686871	PARP1		0.63
ILMN_2190779___PHAX	ILMN	1702396	PACSIN2		0.29
ILMN_2190779___PHAX	ILMN	3278906	LOC644464		0.63
ILMN_2190779___PHAX	ILMN	3202432	LOC644464		1.58
ILMN_2190779___PHAX	ILMN	1699214	LOC647450		1.57
ILMN_2190779___PHAX	ILMN	2380946	EIF4G2		1.95
ILMN_2190779___PHAX	ILMN	1669113	ATF5		0.66
ILMN_2190779___PHAX	ILMN	2313821	AIFM1		0.25
ILMN_2190779___PHAX	ILMN	1703006	MTCH1		0.77
ILMN_2190779___PHAX	ILMN	2279961	LAMP2		0.45
ILMN_2190779___PHAX	ILMN	2191428	UBB		1.50
ILMN_2190779___PHAX	ILMN	3243492	LOC729501		0.30
ILMN_2190779___PHAX	ILMN	1811702	GRN		0.60
ILMN_2190779___PHAX	ILMN	1707775	ZNF257		0.45
ILMN_2190779___PHAX	ILMN	1810172	SFRP4		0.74
ILMN_2190779___PHAX	ILMN	2363621	RBBP8		0.25
ILMN_2190779___PHAX	ILMN	1751258	NDUFA4		0.42
ILMN_2190779___PHAX	ILMN	1657949	RHEB		0.19
ILMN_2190779___PHAX	ILMN	2414007	NME2		1.21
ILMN_2190779___PHAX	ILMN	1663751	CYCSL1		0.49
ILMN_2190779___PHAX	ILMN	1739274	PDHB		0.51
ILMN_2190779___PHAX	ILMN	1705814	KRT80		0.19

ILMN_2190779___PHAX	ILMN	2378952	GPX4	0.32
ILMN_2190779___PHAX	ILMN	3285198	LOC389168	3.03
ILMN_2190779___PHAX	ILMN	1753584	KRT8	0.44
ILMN_2190779___PHAX	ILMN	1773073	PHYH	0.26
ILMN_2190779___PHAX	ILMN	2113470	GSTA1	0.69
ILMN_2190779___PHAX	ILMN	2113470	GSTA1	0.69
ILMN_2190779___PHAX	ILMN	1805826	BIVM	0.42
ILMN_2190779___PHAX	ILMN	3302177	RPL14L	1.44
ILMN_2190779___PHAX	ILMN	2052891	PKD2	0.86
ILMN_2190779___PHAX	ILMN	1752968	LAMB2	0.77
ILMN_2190779___PHAX	ILMN	2119555	TMTC3	0.29
ILMN_2190779___PHAX	ILMN	3203186	LOC100133045	0.66
ILMN_2190779___PHAX	ILMN	2341363	ATP5A1	0.44
ILMN_2190779___PHAX	ILMN	1705213	TMBIM1	0.30
ILMN_2190779___PHAX	ILMN	1655613	GSTA2	0.83
ILMN_2190779___PHAX	ILMN	2345872	SUMF2	0.55
ILMN_2190779___PHAX	ILMN	3248759	LOC732272	0.28
ILMN_2190779___PHAX	ILMN	3294965	LOC100133211	0.22
ILMN_2190779___PHAX	ILMN	1746704	TRIM8	0.30
ILMN_2190779___PHAX	ILMN	1729816	VDAC3	0.37
ILMN_2190779___PHAX	ILMN	1691506	NGRN	0.78
ILMN_2190779___PHAX	ILMN	2113362	ARL6IP1	0.45
ILMN_2190779___PHAX	ILMN	1679809	GSTP1	0.99
ILMN_2190779___PHAX	ILMN	1772369	PDHA1	0.61
ILMN_2190779___PHAX	ILMN	1731714	CREB5	0.25
ILMN_2190779___PHAX	ILMN	1683120	UNG	0.15
ILMN_2190779___PHAX	ILMN	2396410	CS	0.63
ILMN_2190779___PHAX	ILMN	2151281	GABARAPL1	0.43
ILMN_2190779___PHAX	ILMN	3225591	RPL14L	1.40
ILMN_2190779___PHAX	ILMN	2402936	LOC440926	4.63
ILMN_2190779___PHAX	ILMN	2188722	GLS	1.15
ILMN_2190779___PHAX	ILMN	1811063	LOC649447	1.13
ILMN_2190779___PHAX	ILMN	2341815	TFG	0.38
ILMN_2190779___PHAX	ILMN	1732216	NARS	0.19
ILMN_2190779___PHAX	ILMN	2214098	BIVM	0.42
ILMN_2190779___PHAX	ILMN	1715508	NNMT	1.93
ILMN_2190779___PHAX	ILMN	1783636	COX6A1	0.32
ILMN_2190779___PHAX	ILMN	1663684	ATP13A3	0.55
ILMN_2190779___PHAX	ILMN	1701749	UQCRFS1	0.20
ILMN_2190779___PHAX	ILMN	2122952	CISD1	0.46
ILMN_2190779___PHAX	ILMN	1693490	SEC11A	1.41
ILMN_2190779___PHAX	ILMN	1706057	CS	0.21
ILMN_2190779___PHAX	ILMN	2305225	NDRG4	0.42
ILMN_2190779___PHAX	ILMN	2061446	AADACL1	0.72
ILMN_2190779___PHAX	ILMN	3238818	LOC649076	2.84
ILMN_2190779___PHAX	ILMN	1800008	ACAT1	0.30
ILMN_2190779___PHAX	ILMN	1656662	LOC731365	2.66
ILMN_2190779___PHAX	ILMN	1775522	MAGED1	0.68
ILMN_2190779___PHAX	ILMN	2377174	SYPL1	0.68
ILMN_2190779___PHAX	ILMN	2369924	NDUFB6	0.25
ILMN_2190779___PHAX	ILMN	1701293	COX7A2	0.51
ILMN_2190779___PHAX	ILMN	3188449	LOC100129751	0.57

ILMN_2190779___PHAX	ILMN	1806017	PSME1	0.69
ILMN_2190779___PHAX	ILMN	1768870	CAPZA2	0.28
ILMN_2190779___PHAX	ILMN	2243687	LAMP2	0.74
ILMN_2190779___PHAX	ILMN	1732053	SNRNP70	2.95
ILMN_2190779___PHAX	ILMN	1677440	ATP6AP2	0.43
ILMN_2190779___PHAX	ILMN	1698243	C1orf85	0.39
ILMN_2190779___PHAX	ILMN	2372403	ALDH5A1	0.38
ILMN_2190779___PHAX	ILMN	2242463	CTSC	0.45
ILMN_2190779___PHAX	ILMN	1753111	NAMPT	1.82
ILMN_2190779___PHAX	ILMN	1753111	NAMPT	1.82
ILMN_2190779___PHAX	ILMN	1745223	CDC42EP4	0.16
ILMN_2190779___PHAX	ILMN	1719695	NFKBIZ	0.51
ILMN_2190779___PHAX	ILMN	1772612	ANGPTL2	0.34
ILMN_2190779___PHAX	ILMN	1721669	IDH3B	0.63
ILMN_2190779___PHAX	ILMN	3283680	LOC345041	0.17
ILMN_2190779___PHAX	ILMN	1689097	NAT5	0.23
ILMN_2190779___PHAX	ILMN	3307648	CS	0.21
ILMN_2190779___PHAX	ILMN	1693287	POMP	3.39
ILMN_2190779___PHAX	ILMN	1674385	YWHAQ	1.54
ILMN_2190779___PHAX	ILMN	1739798	C7orf30	0.37
ILMN_2190779___PHAX	ILMN	1713450	MYL6B	0.28
ILMN_2190779___PHAX	ILMN	1802557	HEBP1	0.26
ILMN_2190779___PHAX	ILMN	1806692	HEXB	0.39
ILMN_2190779___PHAX	ILMN	1727813	BRP44	0.25
ILMN_2190779___PHAX	ILMN	1773742	DNAJB9	0.21
ILMN_2190779___PHAX	ILMN	2340065	UBL5	0.34
ILMN_2190779___PHAX	ILMN	2045419	BNIP3L	0.58
ILMN_2190779___PHAX	ILMN	1742135	SPAG16	0.39
ILMN_2190779___PHAX	ILMN	3243859	NDUFB9	0.39
ILMN_2190779___PHAX	ILMN	2246661	FAHD1	0.29
ILMN_2190779___PHAX	ILMN	1784523	ATP6V1G1	0.70
ILMN_2190779___PHAX	ILMN	1739263	LOC643284	0.61
ILMN_2190779___PHAX	ILMN	3274045	LOC100130229	0.18
ILMN_2190779___PHAX	ILMN	2122953	CISD1	0.38
ILMN_2190779___PHAX	ILMN	1737146	TRAM1	0.26
ILMN_2190779___PHAX	ILMN	1766981	UNC50	0.31
ILMN_2190779___PHAX	ILMN	1718924	ETFA	0.47
ILMN_2190779___PHAX	ILMN	1752351	LAMP2	0.42
ILMN_2190779___PHAX	ILMN	1654151	COX6C	0.95
ILMN_2190779___PHAX	ILMN	2120210	RCAN2	1.06
ILMN_2190779___PHAX	ILMN	3254413	LOC100129426	0.24
ILMN_2190779___PHAX	ILMN	2352036	RTN4	1.26
ILMN_2190779___PHAX	ILMN	1783852	CD164	0.48
ILMN_2190779___PHAX	ILMN	3247906	RNF114	0.24
ILMN_2190779___PHAX	ILMN	2174369	ELOVL5	0.28
ILMN_2190779___PHAX	ILMN	3213792	LOC439953	3.43
ILMN_2190779___PHAX	ILMN	1756261	GPR143	0.44
ILMN_2190779___PHAX	ILMN	2391765	C6orf48	0.36
ILMN_2190779___PHAX	ILMN	1774062	SLC25A5	0.62
ILMN_2190779___PHAX	ILMN	2205211	LOC134997	2.38
ILMN_2190779___PHAX	ILMN	1815719	PLCG2	0.70
ILMN_2190779___PHAX	ILMN	2409062	ISCU	0.25

ILMN_2190779___PHAX	ILMN	1728512	YWHAH		2.38
ILMN_2190779___PHAX	ILMN	3219455	LOC644745		0.32
ILMN_2190779___PHAX	ILMN	3240838	SLC25A6		0.49
ILMN_2190779___PHAX	ILMN	1807662	IGF2R		0.64
ILMN_2190779___PHAX	ILMN	1801421	EMD		0.34
ILMN_2190779___PHAX	ILMN	1798485	ATP6V1E1		0.58
ILMN_2190779___PHAX	ILMN	1782292	LAMP1		0.72
ILMN_2190779___PHAX	ILMN	2065773	SCG5		0.32
ILMN_2190779___PHAX	ILMN	1809347	LOC729466		0.94
ILMN_2190779___PHAX	ILMN	1801633	C20orf52		0.97
ILMN_2190779___PHAX	ILMN	2362681	CES2		0.32
ILMN_2190779___PHAX	ILMN	1711729	LOC442454		0.35
ILMN_2190779___PHAX	ILMN	1808238	RBPMS2		0.38
ILMN_2190779___PHAX	ILMN	1755391	ARL2BP		0.23
ILMN_2190779___PHAX	ILMN	3234993	ROMO1		0.50
ILMN_2190779___PHAX	ILMN	2225887	ATP5EP2		0.40
ILMN_2190779___PHAX	ILMN	3178252	LOC100129379		0.69
ILMN_2190779___PHAX	ILMN	1688089	PEBP1		0.57
ILMN_2190779___PHAX	ILMN	1672536	FBLN1		0.40
ILMN_2190779___PHAX	ILMN	2230566	RAB40B		0.31
ILMN_2190779___PHAX	ILMN	1741613	SERINC1		0.66
ILMN_2190779___PHAX	ILMN	3242315	SNORD3D		0.64
ILMN_2190779___PHAX	ILMN	1716524	RAB7A		0.68
ILMN_2190779___PHAX	ILMN	1654118	BCL2L1		0.72
ILMN_2190779___PHAX	ILMN	1786759	C11orf10		0.38
ILMN_2190779___PHAX	ILMN	1775937	DDB1		1.04
ILMN_2190779___PHAX	ILMN	1697499	HLA-DRB5		0.86
ILMN_2190779___PHAX	ILMN	3292572	LOC390183		0.30
ILMN_2190779___PHAX	ILMN	1700690	VAT1		0.43
ILMN_2190779___PHAX	ILMN	3202734	LOC100132742		0.57
ILMN_2190779___PHAX	ILMN	3248941	C6orf225		1.59
ILMN_2190779___PHAX	ILMN	3201445	LOC728128		0.45
ILMN_2190779___PHAX	ILMN	2308582	CYB5R3		0.82
ILMN_2190779___PHAX	ILMN	1814526	ADD3		0.41
ILMN_2190779___PHAX	ILMN	1718136	UQCRHL		0.54
ILMN_2190779___PHAX	ILMN	1654430	LOC731170		0.31
ILMN_2190779___PHAX	ILMN	3256742	LOC100129902		0.76
ILMN_2190779___PHAX	ILMN	1736256	CALR		1.55
ILMN_2190779___PHAX	ILMN	3243890	NDUFA2		0.26
ILMN_2190779___PHAX	ILMN	1762531	FGF9		0.52
ILMN_2190779___PHAX	ILMN	3216125	LOC728126		0.98
ILMN_2190779___PHAX	ILMN	3238735	LOC100132346		0.27
ILMN_2190779___PHAX	ILMN	1774375	LOC284422		0.53
ILMN_2190779___PHAX	ILMN	3253304	BRI3P1		0.66
ILMN_2190779___PHAX	ILMN	3235006	LOC728105		1.31
ILMN_2190779___PHAX	ILMN	3246850	LOC648729		0.61
ILMN_2190779___PHAX	ILMN	2415722	RPS29		0.83
ILMN_2190779___PHAX	ILMN	3284177	LOC100132425		2.40
ILMN_2190779___PHAX	ILMN	1803846	EIF1		0.46
ILMN_2190779___PHAX	ILMN	1792138	UQCRH		0.53
ILMN_2190779___PHAX	ILMN	3283449	LOC440991		0.64
ILMN_2190779___PHAX	ILMN	2092536	HSPE1		0.59

ILMN_2190779___PHAX	ILMN	3237507	LOC552889	0.62
ILMN_2190779___PHAX	ILMN	2179018	NDUFAB1	0.61
ILMN_2190779___PHAX	ILMN	3216979	LOC646949	0.73
ILMN_2190779___PHAX	ILMN	1773389	PLTP	0.97
ILMN_2190779___PHAX	ILMN	3275033	LOC646841	0.62
ILMN_2190779___PHAX	ILMN	1659095	LOC644029	0.62
ILMN_2190779___PHAX	ILMN	2396444	CD14	1.35
ILMN_2190779___PHAX	ILMN	1795419	C2orf24	0.29
ILMN_2190779___PHAX	ILMN	2046315	GABARAPL2	0.44
ILMN_2190779___PHAX	ILMN	1798360	CXCR7	1.06
ILMN_2190779___PHAX	ILMN	3248113	SNRNP70	2.36
ILMN_2190779___PHAX	ILMN	3235013	LOC730288	0.51
ILMN_2190779___PHAX	ILMN	1689953	CD81	2.76
ILMN_2190779___PHAX	ILMN	1784286	NDUFA1	0.37
ILMN_2190779___PHAX	ILMN	2160388	RPL24	2.49
ILMN_2190779___PHAX	ILMN	2294762	AMY1A	1.87
ILMN_2190779___PHAX	ILMN	3236259	PPIAL4A	3.00
ILMN_2190779___PHAX	ILMN	1699265	TNFRSF10B	0.90
ILMN_2190779___PHAX	ILMN	1785732	TNFAIP6	0.51
ILMN_2190779___PHAX	ILMN	1728132	LDHB	1.93
ILMN_2190779___PHAX	ILMN	1724811	PARN	0.97
ILMN_2190779___PHAX	ILMN	1738632	PRKAR1A	1.10
ILMN_2190779___PHAX	ILMN	1666471	UQCRQ	0.40
ILMN_2190779___PHAX	ILMN	1687824	SEPHS2	0.24
ILMN_2190779___PHAX	ILMN	1671039	GALNT3	0.82
ILMN_2190779___PHAX	ILMN	2316918	PANK1	0.37
ILMN_2190779___PHAX	ILMN	3287583	LOC648390	0.64
ILMN_2190779___PHAX	ILMN	3237665	COX7A2L	0.38
ILMN_2190779___PHAX	ILMN	1745885	POLR2F	0.48
ILMN_2190779___PHAX	ILMN	2069128	EPB41L2	0.85
ILMN_2190779___PHAX	ILMN	2334760	ARMCX3	0.37
ILMN_2190779___PHAX	ILMN	1658437	SFXN4	1.70
ILMN_2190779___PHAX	ILMN	3282395	LOC646966	0.49
ILMN_2190779___PHAX	ILMN	2169839	CNBP	2.23
ILMN_2190779___PHAX	ILMN	1782439	CNN3	1.20
ILMN_2190779___PHAX	ILMN	2312296	PCBP2	0.72
ILMN_2190779___PHAX	ILMN	1756674	ATP5EP2	0.90
ILMN_2190779___PHAX	ILMN	1652230	LOC647361	1.15
ILMN_2190779___PHAX	ILMN	1671928	PROS1	0.57
ILMN_2190779___PHAX	ILMN	1731174	LOC647856	1.10
ILMN_2190779___PHAX	ILMN	2321451	HNRNPD	1.96
ILMN_2190779___PHAX	ILMN	1708416	ARL6IP1	1.30
ILMN_2190779___PHAX	ILMN	3235357	LOC100133329	0.53
ILMN_2190779___PHAX	ILMN	1656285	METTL7A	0.48
ILMN_2190779___PHAX	ILMN	3210917	LOC389168	0.84
ILMN_2190779___PHAX	ILMN	1772218	HLA-DPA1	0.70
ILMN_2190779___PHAX	ILMN	1728467	GHITM	1.67
ILMN_2190779___PHAX	ILMN	1735499	DCBLD2	0.31
ILMN_2190779___PHAX	ILMN	1740170	CHCHD10	0.47
ILMN_2190779___PHAX	ILMN	1711606	PRDX5	0.75
ILMN_2190779___PHAX	ILMN	1796339	PLEKHA2	0.63
ILMN_2190779___PHAX	ILMN	1713934	LITAF	0.42

ILMN_2190779___PHAX	ILMN	3292056	LOC727984	1.11
ILMN_2190779___PHAX	ILMN	3285785	LOC647307	1.44
ILMN_2190779___PHAX	ILMN	1793287	LOC642755	0.38
ILMN_2190779___PHAX	ILMN	1777344	RPS13	0.72
ILMN_2190779___PHAX	ILMN	2399300	NAV2	0.32
ILMN_2190779___PHAX	ILMN	1658283	RPL17	0.68
ILMN_2190779___PHAX	ILMN	1695588	HNRPC	0.54
ILMN_2190779___PHAX	ILMN	2117330	NDUFB2	1.54
ILMN_2190779___PHAX	ILMN	3290497	LOC643863	0.82
ILMN_2190779___PHAX	ILMN	2404688	NUPR1	1.15
ILMN_2190779___PHAX	ILMN	3225121	LOC728484	1.49
ILMN_2190779___PHAX	ILMN	2389590	PRKAR1A	1.66
ILMN_2190779___PHAX	ILMN	1715896	PMVK	0.53
ILMN_2190779___PHAX	ILMN	3298037	LOC728453	0.50
ILMN_2190779___PHAX	ILMN	2099783	ATP6V1F	1.19
ILMN_2190779___PHAX	ILMN	3235775	LOC553137	1.04
ILMN_2190779___PHAX	ILMN	1651735	TGOLN2	0.35
ILMN_2190779___PHAX	ILMN	3275447	LOC391126	0.61
ILMN_2190779___PHAX	ILMN	1789074	HSPA1A	0.63
ILMN_2190779___PHAX	ILMN	2113535	PCYOX1	0.71
ILMN_2190779___PHAX	ILMN	1756360	RPL35A	0.61
ILMN_2190779___PHAX	ILMN	1795228	ZFAND5	0.73
ILMN_2190779___PHAX	ILMN	1718988	DAZAP2	1.07
ILMN_2190779___PHAX	ILMN	1652207	COX4I1	2.28
ILMN_2190779___PHAX	ILMN	3256674	LOC100128485	0.92
ILMN_2190779___PHAX	ILMN	1764494	ATP5A1	0.87
ILMN_2190779___PHAX	ILMN	1685365	ZNF773	0.97
ILMN_2190779___PHAX	ILMN	2290808	RPL21	1.08
ILMN_2190779___PHAX	ILMN	3304130	LOC729236	0.74
ILMN_2190779___PHAX	ILMN	1687858	CALM2	3.47
ILMN_2190779___PHAX	ILMN	3241034	SNORD3C	0.64
ILMN_2190779___PHAX	ILMN	1809013	MYL6	4.41
ILMN_2190779___PHAX	ILMN	2038772	RPS9	0.73
ILMN_2190779___PHAX	ILMN	1694742	RPS29	1.55
ILMN_2190779___PHAX	ILMN	2225974	GCLM	1.00
ILMN_2190779___PHAX	ILMN	1750636	RPS26L	0.50
ILMN_2190779___PHAX	ILMN	1768392	LOC648059	3.18
ILMN_2190779___PHAX	ILMN	2175465	RSL24D1	1.64
ILMN_2190779___PHAX	ILMN	1663799	RPL32	3.61
ILMN_2190779___PHAX	ILMN	1651354	SPP1	1.09
ILMN_2190779___PHAX	ILMN	3304203	LOC729342	0.58
ILMN_2190779___PHAX	ILMN	1749447	RPS9	0.68
ILMN_2190779___PHAX	ILMN	3188174	LOC100129067	0.56
ILMN_2190779___PHAX	ILMN	1795251	SPARCL1	0.39
ILMN_2190779___PHAX	ILMN	1811367	MAT2B	0.85
ILMN_2190779___PHAX	ILMN	1661917	LOC644039	0.79
ILMN_2190779___PHAX	ILMN	3279277	LOC387930	0.85
ILMN_2190779___PHAX	ILMN	1782890	SLC25A3	0.91
ILMN_2190779___PHAX	ILMN	2203950	HLA-A	3.59
ILMN_2190779___PHAX	ILMN	1772929	ATP5J	0.64
ILMN_2190779___PHAX	ILMN	1766221	B4GALT1	0.69
ILMN_2190779___PHAX	ILMN	1750507	RPL9	1.87

ILMN_2190779___PHAX	ILMN	2053178	ACTG1		5.21
ILMN_2190779___PHAX	ILMN	3285162	LOC100131541		1.20
ILMN_2190779___PHAX	ILMN	3296979	LOC730187		1.11
ILMN_2190779___PHAX	ILMN	2352097	GPR56		0.66
ILMN_2190779___PHAX	ILMN	2233401	FTHL8		0.37
ILMN_2190779___PHAX	ILMN	2383975	PRDX5		0.87
ILMN_2190779___PHAX	ILMN	1683447	LOC440737		1.81
ILMN_2190779___PHAX	ILMN	1794912	ATP5H		2.77
ILMN_2190779___PHAX	ILMN	3293097	LOC645715		1.10
ILMN_2190779___PHAX	ILMN	2110751	CHRNA5		4.90
ILMN_2190779___PHAX	ILMN	1726647	LOC650646		0.64
ILMN_2190779___PHAX	ILMN	1651680	LOC649150		2.14
ILMN_2190779___PHAX	ILMN	2181241	LOC649946		2.98
ILMN_2190779___PHAX	ILMN	3235118	LOC729760		1.75
ILMN_2190779___PHAX	ILMN	3271204	LOC100127893		0.91
ILMN_2190779___PHAX	ILMN	1655710	LOC642989		0.89
ILMN_2190779___PHAX	ILMN	3178553	LOC100128126		4.39
ILMN_2190779___PHAX	ILMN	3204210	LOC284393		1.16
ILMN_2190779___PHAX	ILMN	1719224	C17orf45		2.17
ILMN_2190779___PHAX	ILMN	1654722	MPV17L		1.66
ILMN_2190779___PHAX	ILMN	1665717	EIF2S3		0.97
ILMN_2190779___PHAX	ILMN	2218208	SPARCL1		0.82
ILMN_2190779___PHAX	ILMN	3301324	LOC729301		2.09
ILMN_2190779___PHAX	ILMN	3287093	LOC646819		1.50
ILMN_2190779___PHAX	ILMN	1746856	RAB21		1.36
ILMN_2190779___PHAX	ILMN	1651499	ERGIC1		1.80
ILMN_2190779___PHAX	ILMN	3226613	LOC729686		0.69
ILMN_2190779___PHAX	ILMN	3274666	LOC727826		0.98
ILMN_2190779___PHAX	ILMN	3246292	LOC647099		3.64
ILMN_2190779___PHAX	ILMN	1655690	CDR1		1.44
ILMN_2190779___PHAX	ILMN	1732328	LOC646200		3.83
ILMN_2190779___PHAX	ILMN	3234841	LOC728517		2.19
ILMN_2190779___PHAX	ILMN	2196588	C18orf32		1.51
ILMN_2190779___PHAX	ILMN	1725642	SUMO3		0.58
ILMN_2190779___PHAX	ILMN	2381397	HSPD1		1.44
ILMN_2190779___PHAX	ILMN	1693225	LOC646897		3.10
ILMN_2190779___PHAX	ILMN	1800602	GCA		1.06
ILMN_2190779___PHAX	ILMN	1679587	LOC651202		1.37
ILMN_2190779___PHAX	ILMN	3289171	LOC100131572		1.28
ILMN_2190779___PHAX	ILMN	1744517	GNS		1.03
ILMN_2190779___PHAX	ILMN	2152257	SSTR2		1.59
ILMN_2190779___PHAX	ILMN	2139943	RPS3A		2.42
ILMN_2190779___PHAX	ILMN	2130411	KDELR1		1.78
ILMN_2190779___PHAX	ILMN	1789005	ATP6V0C		2.58
ILMN_2190779___PHAX	ILMN	2348093	ATP5J		0.99
ILMN_2190779___PHAX	ILMN	3293676	LOC100131387		1.63
ILMN_2190779___PHAX	ILMN	3300471	LOC730255		1.73
ILMN_2190779___PHAX	ILMN	1657283	ALKBH5		0.54
ILMN_2190779___PHAX	ILMN	1737988	PRNP		1.00
ILMN_2190779___PHAX	ILMN	2154566	RPL10A		2.89
ILMN_2190779___PHAX	ILMN	2337241	RPS15A		2.70
ILMN_2190779___PHAX	ILMN	2051684	LOC401152		1.34

ILMN_2190779___PHAX	ILMN	1743078	LOC643031		5.71
ILMN_2190779___PHAX	ILMN	1697448	TXNIP		1.04
ILMN_2190779___PHAX	ILMN	2164152	ENPEP		0.95
ILMN_2190779___PHAX	ILMN	1798189	COX7C		0.64
ILMN_2190779___PHAX	ILMN	1661519	KIAA0408		2.02
ILMN_2190779___PHAX	ILMN	3176403	FTHL16		1.50
ILMN_2190779___PHAX	ILMN	1795937	VIL2		2.09
ILMN_2190779___PHAX	ILMN	1683597	LOC652071		2.40
ILMN_2190779___PHAX	ILMN	3201517	LOC389101		3.52
ILMN_2190779___PHAX	ILMN	1791114	CDR1		1.44
ILMN_2190779___PHAX	ILMN	1751708	ITM2B		1.39
ILMN_2190779___PHAX	ILMN	3246360	LOC100130553		1.69
ILMN_2190779___PHAX	ILMN	1716678	NPC2		2.76
ILMN_2190779___PHAX	ILMN	1693311	TMBIM6		1.38
ILMN_2190779___PHAX	ILMN	3275224	LOC442162		0.96
ILMN_2190779___PHAX	ILMN	1685608	NPTX2		1.53
ILMN_2190779___PHAX	ILMN	3239574	SNORD3A		1.35
ILMN_2190779___PHAX	ILMN	3277297	LOC391825		2.07
ILMN_2190779___PHAX	ILMN	1810577	RPS4X		2.25
ILMN_2190779___PHAX	ILMN	3203515	LOC401717		1.36
ILMN_2190779___PHAX	ILMN	2405684	BIRC3		3.37
ILMN_2190779___PHAX	ILMN	2206188	SHROOM4		4.80
ILMN_2190779___PHAX	ILMN	1713394	LOC441876		1.16
ILMN_2190779___PHAX	ILMN	1656807	RPL27		2.75
ILMN_2190779___PHAX	ILMN	3228294	LOC729279		1.76
ILMN_2190779___PHAX	ILMN	1660436	HSPA1B		1.02
ILMN_2190779___PHAX	ILMN	3298215	LOC729340		1.97
ILMN_2190779___PHAX	ILMN	2219134	RPS15		1.61
ILMN_2190779___PHAX	ILMN	1786359	LOC346950		1.39
ILMN_2190779___PHAX	ILMN	1769508	PIGX		2.30
ILMN_2190779___PHAX	ILMN	2099594	SRP9		1.32
ILMN_2190779___PHAX	ILMN	1726666	GPX3		2.31
ILMN_2190779___PHAX	ILMN	3202673	LOC100131196		2.99
ILMN_2190779___PHAX	ILMN	3268914	LOC100128410		1.11
ILMN_2190779___PHAX	ILMN	3276794	LOC440027		3.79
ILMN_2190779___PHAX	ILMN	3225505	LOC728553		3.78
ILMN_2190779___PHAX	ILMN	1667813	LOC648000		2.06
ILMN_2190779___PHAX	ILMN	2115949	ZNF394		5.90
ILMN_2190779___PHAX	ILMN	2383097	RPL17		1.42
ILMN_2190779___PHAX	ILMN	3238491	LOC100129055		2.20
ILMN_2190779___PHAX	ILMN	1677697	LOC441377		1.20
ILMN_2190779___PHAX	ILMN	2157441	HLA-DRA		2.33
ILMN_2190779___PHAX	ILMN	3251605	KLHL28		7.44
ILMN_2190779___PHAX	ILMN	3200484	LOC126235		1.79
ILMN_2190779___PHAX	ILMN	2336781	SOD2		1.37
ILMN_2190779___PHAX	ILMN	3199798	LOC389342		1.74
ILMN_2190779___PHAX	ILMN	1662618	SQSTM1		1.74
ILMN_2190779___PHAX	ILMN	2228196	C6orf170		2.01
ILMN_2190779___PHAX	ILMN	1760089	LOC645018		2.92
ILMN_2190779___PHAX	ILMN	3237729	LOC100133551		2.74
ILMN_2190779___PHAX	ILMN	3271244	LOC100130775		1.46
ILMN_2190779___PHAX	ILMN	2386530	RPLP1		2.18

ILMN_2190779___PHAX	ILMN	1685378	LOC728973		3.40
ILMN_2190779___PHAX	ILMN	2323048	ERP29		2.20
ILMN_2190779___PHAX	ILMN	1663416	LOC642250		1.46
ILMN_2190779___PHAX	ILMN	2338785	RPS14		5.07
ILMN_2190779___PHAX	ILMN	1659405	LOC653773		1.99
ILMN_2190779___PHAX	ILMN	3286204	LOC646547		3.61
ILMN_2190779___PHAX	ILMN	3234831	LOC645296		1.30
ILMN_2190779___PHAX	ILMN	1812995	CTSL1		3.22
ILMN_2190779___PHAX	ILMN	2148459	B2M		4.93
ILMN_2190779___PHAX	ILMN	2344204	PRR13		3.93
ILMN_2190779___PHAX	ILMN	1684771	PGRMC1		1.04
ILMN_2190779___PHAX	ILMN	3225761	MGC87895		1.06
ILMN_2190779___PHAX	ILMN	1757387	UCHL1		0.68
ILMN_2190779___PHAX	ILMN	2114876	RPL11		4.19
ILMN_2190779___PHAX	ILMN	1674236	HSPB1		1.63
ILMN_2190779___PHAX	ILMN	1791280	HSPB8		1.98
ILMN_2190779___PHAX	ILMN	3207020	LOC388556		1.29
ILMN_2190779___PHAX	ILMN	3235912	C7orf38		1.97
ILMN_2190779___PHAX	ILMN	1810810	EEF1A1		3.74
ILMN_2190779___PHAX	ILMN	1695261	LOC285176		1.66
ILMN_2190779___PHAX	ILMN	3180420	LOC100129269		4.85
ILMN_2190779___PHAX	ILMN	3288161	LOC100132488		2.75
ILMN_2190779___PHAX	ILMN	2207533	RPS17		2.04
ILMN_2190779___PHAX	ILMN	3243441	EEF1AL7		3.12
ILMN_2190779___PHAX	ILMN	2184789	HSCB		2.61
ILMN_2190779___PHAX	ILMN	3264073	LOC100130070		2.13
ILMN_2190779___PHAX	ILMN	2107004	GPR1		2.86
ILMN_2190779___PHAX	ILMN	3285742	LOC100131786		2.49
ILMN_2190779___PHAX	ILMN	3267800	LOC100130276		5.37
ILMN_2190779___PHAX	ILMN	2374036	CTSL1		3.62
ILMN_2190779___PHAX	ILMN	2174127	DCBLD2		1.80
ILMN_2190779___PHAX	ILMN	3275936	LOC100133277		3.87
ILMN_2190779___PHAX	ILMN	2394132	PRO1853		2.14
ILMN_2190779___PHAX	ILMN	2247594	RPLP1		2.56
ILMN_2190779___PHAX	ILMN	1666078	HLA-H		2.53
ILMN_2190779___PHAX	ILMN	1725427	B2M		2.64
ILMN_2190779___PHAX	ILMN	1737015	RPL39		1.41
ILMN_2190779___PHAX	ILMN	3245403	LOC100133931		1.65
ILMN_2190779___PHAX	ILMN	3194217	LOC100129424		3.91
ILMN_2190779___PHAX	ILMN	2051519	RPL37A		1.71
ILMN_2190779___PHAX	ILMN	1785703	LMOD3		6.34
ILMN_2190779___PHAX	ILMN	2219437	PRRG4		6.51
ILMN_2190779___PHAX	ILMN	3291986	LOC642892		2.47
ILMN_2190779___PHAX	ILMN	2230624	RPL18		3.51
ILMN_2190779___PHAX	ILMN	2096970	MYO3B		3.85
ILMN_2190779___PHAX	ILMN	2391178	STAR		2.13
ILMN_2190779___PHAX	ILMN	3190442	LOC100128591		2.84
ILMN_2190779___PHAX	ILMN	3199929	LOC390183		2.93
ILMN_2190779___PHAX	ILMN	2210386	TMEM17		7.34
ILMN_2190779___PHAX	ILMN	3224952	LOC729903		3.81
ILMN_2190779___PHAX	ILMN	2091846	FTHL2		0.97
ILMN_2190779___PHAX	ILMN	2234016	FTHL7		2.04

ILMN_2190779___PHAX	ILMN	3285153	LOC645979		1.72
ILMN_2190779___PHAX	ILMN	1672446	RPL11		4.24
ILMN_2190779___PHAX	ILMN	3301585	LOC730993		3.25
ILMN_2190779___PHAX	ILMN	1737298	MAT2A		2.68
ILMN_2190779___PHAX	ILMN	1772132	ATP5B		3.21
ILMN_2190779___PHAX	ILMN	3297317	LOC728658		2.51
ILMN_2190779___PHAX	ILMN	1810628	KIAA0367		0.89
ILMN_2190779___PHAX	ILMN	3260286	LOC100128062		6.24
ILMN_2190779___PHAX	ILMN	3272378	EZR		2.86
ILMN_2190779___PHAX	ILMN	2101651	MBTD1		2.55
ILMN_2190779___PHAX	ILMN	1746516	RPS25		6.21
ILMN_2190779___PHAX	ILMN	2149400	SPC25		3.78
ILMN_2190779___PHAX	ILMN	1677691	LOC648852		6.95
ILMN_2190779___PHAX	ILMN	1773080	OAZ1		5.19
ILMN_2190779___PHAX	ILMN	1780141	TMEM66		1.16
ILMN_2190779___PHAX	ILMN	1651850	RPS16		3.97
ILMN_2190779___PHAX	ILMN	1666635	RPS14		2.39
ILMN_2190779___PHAX	ILMN	2343775	RPL38		4.11
ILMN_2190779___PHAX	ILMN	3199974	LOC100131787		2.75
ILMN_2190779___PHAX	ILMN	1814823	FTL		3.21
ILMN_2190779___PHAX	ILMN	1778202	FLJ40722		4.53
ILMN_2190779___PHAX	ILMN	1740429	FTL		3.12
ILMN_2190779___PHAX	ILMN	3213573	LOC645715		5.13
ILMN_2190779___PHAX	ILMN	1738243	RPS29		3.18
ILMN_2190779___PHAX	ILMN	1652073	LOC653658		3.59
ILMN_2190779___PHAX	ILMN	3266128	LOC100129553		2.41
ILMN_2190779___PHAX	ILMN	2176882	ZNF69		7.92
ILMN_2190779___PHAX	ILMN	2064898	CCDC56		2.27
ILMN_2190779___PHAX	ILMN	2117569	DEM1		7.09
ILMN_2190779___PHAX	ILMN	1787949	RPS15A		2.13
ILMN_2190779___PHAX	ILMN	2370135	HNRNPU		6.18
ILMN_2190779___PHAX	ILMN	3202024	LOC392437		3.13
ILMN_2190779___PHAX	ILMN	1696839	RPS27		4.08
ILMN_2190779___PHAX	ILMN	3209317	LOC644949		2.31
ILMN_2190779___PHAX	ILMN	2204909	XRCC2		6.09
ILMN_2190779___PHAX	ILMN	3225673	LOC730060		2.84
ILMN_2190779___PHAX	ILMN	2370573	XAF1		3.55
ILMN_2190779___PHAX	ILMN	1801040	SPN		3.57
ILMN_2190779___PHAX	ILMN	3201216	LOC441550		2.62
ILMN_2190779___PHAX	ILMN	3252113	LOC100130107		4.87
ILMN_2190779___PHAX	ILMN	3248069	LOC653881		3.35
ILMN_2190779___PHAX	ILMN	3280952	LOC391777		5.35
ILMN_2190779___PHAX	ILMN	2209993	PLIN5		5.70
ILMN_2190779___PHAX	ILMN	1754303	RPL30		2.48
ILMN_2190779___PHAX	ILMN	2227495	ZNF549		8.89
ILMN_2190779___PHAX	ILMN	2279367	FCAR		3.12
ILMN_2190779___PHAX	ILMN	3208014	LOC100131866		3.31
ILMN_2190779___PHAX	ILMN	3275345	LOC100132291		2.87
ILMN_2190779___PHAX	ILMN	2154322	SEMA3E		6.33
ILMN_2190779___PHAX	ILMN	2235745	GRIPAP1		6.74
ILMN_2190779___PHAX	ILMN	1722492	DUSP19		8.24
ILMN_2190779___PHAX	ILMN	3274601	LOC343184		3.67

ILMN_2190779___PHAX	ILMN	3291673	LOC100131452	3.12
ILMN_2190779___PHAX	ILMN	1792528	LOC401206	6.46
ILMN_2190779___PHAX	ILMN	1765043	RPL38	3.49
ILMN_2190779___PHAX	ILMN	1696911	FTHL8	4.54
ILMN_2190779___PHAX	ILMN	3201843	LOC644604	5.39
ILMN_2190779___PHAX	ILMN	1746919	LOC643882	3.05
ILMN_2190779___PHAX	ILMN	1737878	FLJ34047	4.66
ILMN_2190779___PHAX	ILMN	3220792	LOC728658	3.21
ILMN_2190779___PHAX	ILMN	1755115	RPL23	4.48
ILMN_2190779___PHAX	ILMN	3275275	LOC727962	5.20
ILMN_2190779___PHAX	ILMN	2352245	RASSF6	3.99
ILMN_2190779___PHAX	ILMN	2059173	SLC35E1	4.53
ILMN_2190779___PHAX	ILMN	2173835	FTHL3	3.28
ILMN_2190779___PHAX	ILMN	1652955	LOC648622	2.83
ILMN_2190779___PHAX	ILMN	1736008	YRDC	8.27
ILMN_2190779___PHAX	ILMN	2049364	FAM119A	8.96
ILMN_2190779___PHAX	ILMN	2150294	FKBP14	7.45
ILMN_2190779___PHAX	ILMN	1680437	LOC731096	3.24
ILMN_2190779___PHAX	ILMN	3257030	FTHL16	2.41
ILMN_2190779___PHAX	ILMN	2415979	KIAA1751	10.36
ILMN_2190779___PHAX	ILMN	3275489	LOC100131905	2.10
ILMN_2190779___PHAX	ILMN	3275771	LOC642567	4.55
ILMN_2190779___PHAX	ILMN	1736567	CD74	2.51
ILMN_2190779___PHAX	ILMN	3246255	LOC100133516	7.69
ILMN_2190779___PHAX	ILMN	3245380	ZNF860	2.55
ILMN_2190779___PHAX	ILMN	2059844	ANKRD44	5.01
ILMN_2190779___PHAX	ILMN	1729216	CRYAB	2.97
ILMN_2190779___PHAX	ILMN	3245600	LRRC37B2	7.13
ILMN_2190779___PHAX	ILMN	2285713	TDP1	6.20
ILMN_2190779___PHAX	ILMN	3229038	LOC728310	2.75
ILMN_2190779___PHAX	ILMN	2073543	C15orf63	5.32
ILMN_2190779___PHAX	ILMN	2290732	ZNF626	3.87
ILMN_2190779___PHAX	ILMN	1764721	RPL8	3.45
ILMN_2190779___PHAX	ILMN	1755808	LOC654194	2.38
ILMN_2190779___PHAX	ILMN	2214997	LRRFIP1	4.47
ILMN_2190779___PHAX	ILMN	2117809	DUXAP3	8.21
ILMN_2190779___PHAX	ILMN	1706013	FTHL11	2.38
ILMN_2190779___PHAX	ILMN	2212823	ZNF577	6.33
ILMN_2190779___PHAX	ILMN	3304396	LOC730202	5.84
ILMN_2190779___PHAX	ILMN	3226663	MGC26356	10.43
ILMN_2190779___PHAX	ILMN	1798581	MCM8	6.61
ILMN_2190779___PHAX	ILMN	1699383	POLR2J4	6.02
ILMN_2190779___PHAX	ILMN	3300797	LOC729090	10.64
ILMN_2190779___PHAX	ILMN	1676792	LOC645138	4.07
ILMN_2190779___PHAX	ILMN	1738383	EEF2	5.16
ILMN_2190779___PHAX	ILMN	1709348	ALDH1A1	0.92
ILMN_2190779___PHAX	ILMN	3235410	HIATL2	5.23
ILMN_2190779___PHAX	ILMN	2346137	ZNF557	5.14
ILMN_2190779___PHAX	ILMN	1671661	HSD17B7	7.11
ILMN_2190779___PHAX	ILMN	2055310	MBD4	5.58
ILMN_2190779___PHAX	ILMN	1660498	RPS27	3.09
ILMN_2190779___PHAX	ILMN	1749109	PSAP	4.59

ILMN_2190779___PHAX	ILMN	2121068	ADAM17	3.48
ILMN_2190779___PHAX	ILMN	1690443	C14orf82	2.74
ILMN_2190779___PHAX	ILMN	3238640	LOC100133812	3.08
ILMN_2190779___PHAX	ILMN	1701314	LOC399900	7.70
ILMN_2190779___PHAX	ILMN	1782977	UBA52	4.82
ILMN_2190779___PHAX	ILMN	3249244	TMEM106A	5.82
ILMN_2190779___PHAX	ILMN	1721349	MAGT1	6.66
ILMN_2190779___PHAX	ILMN	1680279	USP49	6.21
ILMN_2190779___PHAX	ILMN	2133360	LOC91561	5.91
ILMN_2190779___PHAX	ILMN	2160819	RPS27	4.33
ILMN_2190779___PHAX	ILMN	3227315	LOC729009	3.52
ILMN_2190779___PHAX	ILMN	3223500	LOC728903	10.53
ILMN_2190779___PHAX	ILMN	2211724	C21orf24	9.26
ILMN_2190779___PHAX	ILMN	2279834	ZNF483	6.34
ILMN_2190779___PHAX	ILMN	2355559	PSAP	4.65
ILMN_2190779___PHAX	ILMN	3181480	FLJ36131	8.63
ILMN_2190779___PHAX	ILMN	2407851	IL17RD	6.55
ILMN_2190779___PHAX	ILMN	1740587	RPS11	3.98
ILMN_2190779___PHAX	ILMN	2105033	PLDN	6.70
ILMN_2190779___PHAX	ILMN	2407824	ATP1B1	2.99
ILMN_2190779___PHAX	ILMN	2189870	FCF1	5.72
ILMN_2190779___PHAX	ILMN	1743476	LOC653829	7.11
ILMN_2190779___PHAX	ILMN	3240943	LOC100133772	5.45
ILMN_2190779___PHAX	ILMN	1750278	FTHL12	4.64
ILMN_2190779___PHAX	ILMN	2207505	LEP	4.61
ILMN_2190779___PHAX	ILMN	1665823	LOC389787	4.52
ILMN_2190779___PHAX	ILMN	3215461	LOC100131989	6.74
ILMN_2190779___PHAX	ILMN	3294213	LOC401098	6.86
ILMN_2190779___PHAX	ILMN	3302499	LOC730990	6.19
ILMN_2190779___PHAX	ILMN	2297069	FAM115A	10.22
ILMN_2190779___PHAX	ILMN	1658071	ATP1B1	4.26
ILMN_2190779___PHAX	ILMN	1701596	RPS20	4.28
ILMN_2190779___PHAX	ILMN	2048811	NUBPL	6.16
ILMN_2190779___PHAX	ILMN	2094718	TPT1	6.49
ILMN_2190779___PHAX	ILMN	3187470	LOC100129094	7.49
ILMN_2190779___PHAX	ILMN	2188119	ARL16	7.70
ILMN_2190779___PHAX	ILMN	3239895	LOC100134053	5.61
ILMN_2190779___PHAX	ILMN	3262849	LOC100128510	7.73
ILMN_2190779___PHAX	ILMN	2091123	HCG2P7	6.82
ILMN_2190779___PHAX	ILMN	2307903	VCAM1	2.57
ILMN_2190779___PHAX	ILMN	2286014	CATSPER2	8.55
ILMN_2190779___PHAX	ILMN	2313889	ZNF682	8.29
ILMN_2190779___PHAX	ILMN	2235785	KCNH6	7.49
ILMN_2190779___PHAX	ILMN	2243516	C11orf63	4.98
ILMN_2190779___PHAX	ILMN	2123559	FAM73A	8.73
ILMN_2190779___PHAX	ILMN	3261439	LOC100128098	10.07
ILMN_2190779___PHAX	ILMN	3255792	LOC100128505	8.86
ILMN_2190779___PHAX	ILMN	3225102	ZNF738	6.43
ILMN_2190779___PHAX	ILMN	2215382	DDX51	7.77
ILMN_2190779___PHAX	ILMN	2134538	FTHL11	4.31
ILMN_2190779___PHAX	ILMN	1811221	SLC5A8	6.79
ILMN_2190779___PHAX	ILMN	1746525	FTHL2	4.01

ILMN_2190779___PHAX	ILMN	2262275	TRIM13	8.38
ILMN_2190779___PHAX	ILMN	3289685	LOC645452	9.69
ILMN_2190779___PHAX	ILMN	3238053	LOC100129211	10.31
ILMN_2190779___PHAX	ILMN	1759872	LOC643509	8.55
ILMN_2190779___PHAX	ILMN	2148290	PDCD7	10.79
ILMN_2190779___PHAX	ILMN	2276758	POFUT1	7.82
ILMN_2190779___PHAX	ILMN	3305169	LOC728809	9.84
ILMN_2190779___PHAX	ILMN	2051900	EID2B	7.74
ILMN_2190779___PHAX	ILMN	3232828	LOC728620	9.50
ILMN_2190779___PHAX	ILMN	2053538	RHBDL2	5.80
ILMN_2190779___PHAX	ILMN	3213185	LOC645452	7.33
ILMN_2190779___PHAX	ILMN	3187612	LOC100128084	8.44
ILMN_2190779___PHAX	ILMN	3187283	LOC100128460	6.53
ILMN_2190779___PHAX	ILMN	2075794	NLRP8	8.69
ILMN_2190779___PHAX	ILMN	3187425	LOC100127975	7.52
ILMN_2190779___PHAX	ILMN	1693789	ALPP	7.05
ILMN_2190779___PHAX	ILMN	2129545	GNB4	5.35
ILMN_2190779___PHAX	ILMN	2050434	MGC16703	10.71
ILMN_2190779___PHAX	ILMN	2061419	CYCSL1	7.26
ILMN_2190779___PHAX	ILMN	1692145	ZNF14	9.28
ILMN_2190779___PHAX	ILMN	3231550	LOC100131718	7.77
ILMN_2190779___PHAX	ILMN	2162367	DMC1	8.37
ILMN_2190779___PHAX	ILMN	1772492	MCART1	8.01
ILMN_2190779___PHAX	ILMN	3237404	LOC100132585	9.60
ILMN_2190779___PHAX	ILMN	2130078	CDKN2AIPNL	9.64
ILMN_2190779___PHAX	ILMN	1711156	LOC645895	10.33
ILMN_2190779___PHAX	ILMN	2122511	CCBE1	8.76
ILMN_2190779___PHAX	ILMN	3227321	LOC731542	9.46
ILMN_2190779___PHAX	ILMN	3294134	LOC389765	8.03
ILMN_2190779___PHAX	ILMN	3231944	LOC100130516	7.66
ILMN_2190779___PHAX	ILMN	2249473	SPTLC1	7.75
ILMN_2190779___PHAX	ILMN	2105253	PTGR2	7.29

ExpreControl	CoexpSunitinib	CoexpControl	DE	A	PIF	Abs(DC)
4.34	-0.51	0.82	0.47	4.57	2.13	1.33
6.71	-0.67	0.48	0.35	6.88	2.39	1.15
9.49	0.50	-0.01	0.46	9.72	4.50	0.51
5.59	0.69	-0.33	0.33	5.76	1.92	1.01
8.25	-0.26	0.19	0.47	8.49	3.96	0.45
1.15	-0.72	0.27	1.04	1.67	1.73	0.99
9.81	-0.53	0.05	0.29	9.95	2.84	0.57
1.26	-0.60	0.39	0.88	1.70	1.50	0.99
9.03	0.50	0.10	0.39	9.22	3.58	0.40
0.88	0.74	-0.20	1.01	1.38	1.39	0.94
4.72	0.89	-0.39	0.21	4.82	1.01	1.28
3.21	0.69	-0.21	0.41	3.42	1.41	0.90
3.33	-0.60	0.26	0.41	3.54	1.46	0.86
0.76	-0.76	0.10	1.04	1.28	1.34	0.86
1.97	0.79	-0.33	0.46	2.20	1.01	1.12
0.70	0.69	-0.84	0.70	1.05	0.73	1.53
4.44	-0.49	-0.10	0.59	4.74	2.79	0.39
1.83	0.77	-0.39	0.45	2.06	0.93	1.16
0.74	-0.60	-0.08	1.38	1.43	1.97	0.53
0.86	-0.63	0.21	0.93	1.33	1.24	0.83
4.28	-0.52	-0.13	0.58	4.57	2.67	0.39
2.10	0.79	-0.36	0.38	2.29	0.87	1.15
3.98	0.72	-0.06	0.31	4.13	1.27	0.78
4.29	0.80	0.34	0.47	4.52	2.14	0.45
1.27	0.92	-0.26	0.53	1.53	0.81	1.18
6.55	0.70	0.17	0.26	6.68	1.76	0.53
1.87	-0.63	0.36	0.45	2.09	0.94	0.99
1.00	-0.50	0.33	0.76	1.39	1.06	0.83
1.03	-0.59	0.77	0.50	1.28	0.64	1.36
6.64	0.51	0.06	0.28	6.78	1.92	0.45
7.39	0.66	0.25	0.27	7.53	2.04	0.40
9.56	-0.28	-0.08	0.42	9.77	4.13	0.20
3.78	0.30	-0.29	0.35	3.96	1.37	0.59
0.82	-0.68	0.11	0.82	1.23	1.01	0.79
2.74	0.82	-0.15	0.27	2.88	0.78	0.96
4.61	-0.53	-0.17	0.42	4.82	2.01	0.36
6.10	0.65	0.21	0.27	6.23	1.65	0.44
0.63	-0.82	0.28	0.68	0.97	0.66	1.10
4.42	0.59	0.00	0.27	4.55	1.23	0.59
0.88	-0.82	0.11	0.65	1.20	0.77	0.93
2.61	0.58	-0.16	0.34	2.78	0.96	0.74
7.33	0.62	0.25	0.26	7.47	1.96	0.36
1.24	-0.81	0.12	0.50	1.49	0.75	0.93
0.52	-0.84	0.73	0.55	0.79	0.44	1.57
2.03	0.58	-0.43	0.31	2.18	0.68	1.01
2.20	0.89	-0.46	0.22	2.31	0.50	1.34
0.66	-0.77	0.02	0.80	1.06	0.85	0.79
6.84	0.61	0.20	0.23	6.96	1.59	0.41
0.60	-0.72	0.12	0.78	0.99	0.77	0.84
3.53	0.07	-0.95	0.17	3.62	0.63	1.02
1.87	0.94	-0.63	0.21	1.97	0.41	1.57

2.13	0.86	0.26	0.44	2.35	1.04	0.60
2.02	0.84	-0.06	0.32	2.18	0.69	0.90
1.37	-0.08	0.31	0.88	1.81	1.59	0.39
1.41	-0.58	-0.14	0.77	1.80	1.39	0.44
1.09	-0.56	0.26	0.54	1.36	0.74	0.83
2.45	0.50	-0.15	0.34	2.62	0.90	0.66
1.39	0.74	-0.23	0.38	1.59	0.61	0.96
4.98	0.06	-0.20	0.43	5.19	2.24	0.26
3.91	-0.50	0.09	0.24	4.03	0.97	0.58
0.75	-0.80	0.06	0.62	1.06	0.65	0.86
4.22	0.66	0.09	0.23	4.33	0.98	0.57
1.93	-0.38	-0.12	0.89	2.38	2.11	0.26
1.97	-0.36	-0.11	0.90	2.42	2.18	0.25
4.19	0.53	0.14	0.32	4.35	1.41	0.39
0.92	0.50	-0.44	0.50	1.17	0.58	0.93
3.55	0.26	0.74	0.30	3.70	1.12	0.48
1.42	-0.56	-0.28	0.98	1.91	1.86	0.28
2.22	-0.33	0.30	0.35	2.40	0.83	0.63
1.57	-0.58	0.10	0.42	1.79	0.76	0.68
3.80	0.05	-0.29	0.38	3.99	1.52	0.34
3.59	-0.03	0.47	0.28	3.72	1.04	0.50
3.91	0.66	0.24	0.30	4.06	1.20	0.42
0.81	0.38	-0.81	0.42	1.02	0.43	1.19
3.49	0.42	-0.18	0.23	3.61	0.84	0.60
2.79	0.59	-0.12	0.25	2.92	0.72	0.71
2.87	0.72	-0.20	0.18	2.96	0.52	0.92
0.97	-0.66	0.28	0.43	1.18	0.51	0.94
1.36	0.51	-0.23	0.41	1.56	0.64	0.74
1.40	0.71	-0.20	0.33	1.56	0.52	0.91
0.60	-0.84	0.66	0.39	0.80	0.31	1.50
3.93	0.82	0.36	0.25	4.05	1.01	0.46
1.07	0.43	-0.40	0.43	1.29	0.56	0.84
1.09	0.22	-0.38	0.56	1.37	0.77	0.60
0.52	-0.85	0.71	0.41	0.72	0.30	1.56
1.76	-0.06	-0.61	0.42	1.97	0.83	0.55
1.25	-0.32	-0.63	0.86	1.68	1.45	0.31
0.60	-0.56	0.40	0.54	0.87	0.47	0.96
1.31	0.83	-0.41	0.25	1.44	0.36	1.24
3.23	-0.07	-0.27	0.62	3.54	2.21	0.20
2.88	-0.58	-0.32	0.55	3.15	1.72	0.26
2.42	-0.44	-0.16	0.59	2.71	1.60	0.28
0.49	-0.89	0.05	0.59	0.79	0.47	0.94
2.38	0.59	0.26	0.49	2.63	1.28	0.34
0.98	-0.74	0.22	0.38	1.17	0.45	0.96
1.63	0.56	-0.21	0.31	1.78	0.55	0.78
1.46	-0.62	0.28	0.30	1.61	0.47	0.90
0.35	-0.87	-0.23	0.85	0.77	0.66	0.64
2.95	0.90	0.01	0.15	3.02	0.47	0.89
5.66	0.57	0.31	0.27	5.80	1.58	0.26
1.66	0.81	0.32	0.44	1.88	0.83	0.48
1.02	0.79	-0.20	0.33	1.19	0.40	0.99
4.53	-0.26	-0.43	0.50	4.78	2.38	0.16

0.70	0.66	-0.46	0.39	0.89	0.35	1.13
2.04	0.75	-0.09	0.21	2.15	0.45	0.84
0.33	-0.66	0.63	0.50	0.58	0.29	1.29
1.13	-0.15	-0.83	0.41	1.33	0.55	0.67
0.97	0.68	-0.20	0.36	1.15	0.42	0.87
1.04	0.75	-0.07	0.37	1.22	0.45	0.82
2.54	-0.63	-0.36	0.49	2.78	1.35	0.27
0.99	-0.58	-0.24	0.76	1.37	1.05	0.34
1.01	0.22	-0.22	0.62	1.32	0.81	0.44
2.00	-0.01	-0.19	0.82	2.41	1.97	0.18
2.38	-0.71	-0.35	0.38	2.58	0.98	0.36
1.17	0.78	0.22	0.44	1.40	0.62	0.56
0.72	0.63	-0.38	0.37	0.91	0.34	1.02
1.78	0.25	-0.22	0.36	1.96	0.71	0.48
3.69	-0.11	0.24	0.25	3.81	0.97	0.35
1.29	0.67	-0.22	0.26	1.42	0.38	0.90
3.02	0.16	0.69	0.20	3.12	0.64	0.53
2.10	0.43	-0.03	0.32	2.26	0.72	0.46
0.79	0.47	-0.14	0.51	1.05	0.53	0.60
1.05	-0.36	0.57	0.28	1.19	0.34	0.93
2.01	0.57	0.08	0.30	2.16	0.64	0.49
0.58	0.47	-0.88	0.31	0.74	0.23	1.35
7.22	0.58	0.72	0.30	7.37	2.19	0.14
2.06	0.57	-0.16	0.20	2.16	0.43	0.73
0.59	-0.96	0.62	0.27	0.73	0.20	1.58
0.56	0.79	-0.52	0.32	0.72	0.23	1.30
0.72	0.03	-0.46	0.60	1.02	0.62	0.49
1.05	0.93	-0.21	0.23	1.17	0.26	1.14
0.91	0.49	-0.45	0.30	1.06	0.32	0.94
1.47	0.38	-0.25	0.29	1.62	0.47	0.63
0.83	-0.51	0.30	0.36	1.01	0.37	0.80
1.09	-0.75	-0.37	0.56	1.37	0.77	0.38
1.16	0.41	-0.30	0.31	1.31	0.41	0.72
1.01	-0.70	0.05	0.33	1.17	0.39	0.74
4.09	0.82	0.52	0.23	4.20	0.97	0.30
0.43	0.73	-0.27	0.44	0.65	0.29	1.00
0.55	-0.94	0.61	0.27	0.68	0.19	1.55
0.83	0.84	-0.07	0.32	0.99	0.31	0.91
1.94	0.74	-0.08	0.17	2.02	0.35	0.82
1.53	0.42	-0.19	0.28	1.67	0.46	0.61
0.59	0.25	-0.81	0.35	0.77	0.27	1.06
0.96	-0.50	-0.70	0.97	1.44	1.39	0.20
0.87	0.69	-0.47	0.25	0.99	0.24	1.16
0.71	0.87	-0.39	0.26	0.84	0.22	1.26
0.89	0.81	-0.42	0.23	1.00	0.23	1.23
0.84	-0.62	-0.91	0.77	1.23	0.94	0.29
1.71	0.70	0.05	0.23	1.82	0.41	0.66
1.26	-0.47	-0.27	0.80	1.66	1.32	0.20
0.64	-0.81	0.32	0.30	0.79	0.23	1.13
1.28	0.62	-0.28	0.21	1.38	0.29	0.89
0.46	-0.76	0.32	0.37	0.65	0.24	1.08
1.95	0.62	0.44	0.62	2.26	1.39	0.18

0.85	-0.75	-0.43	0.67	1.19	0.80	0.31
0.74	0.82	-0.28	0.26	0.87	0.23	1.10
0.48	-0.74	0.14	0.41	0.69	0.28	0.88
0.71	-0.71	0.28	0.30	0.86	0.25	0.99
0.50	-0.95	0.59	0.26	0.63	0.16	1.55
0.73	-0.33	0.37	0.38	0.92	0.35	0.71
2.40	0.33	-0.16	0.20	2.50	0.51	0.49
0.57	-0.56	0.60	0.30	0.72	0.21	1.16
0.59	-0.69	0.34	0.32	0.75	0.24	1.03
1.50	0.11	-0.25	0.40	1.70	0.68	0.36
0.92	0.90	-0.19	0.22	1.03	0.22	1.10
0.80	-0.47	0.30	0.33	0.97	0.32	0.76
1.82	0.31	-0.06	0.33	1.98	0.65	0.37
0.38	-0.29	-0.63	0.86	0.81	0.70	0.34
3.59	-0.27	-0.08	0.33	3.76	1.24	0.19
0.62	-0.38	0.46	0.35	0.79	0.28	0.83
0.32	0.64	-0.44	0.41	0.52	0.21	1.08
0.92	-0.02	-0.68	0.32	1.08	0.35	0.66
2.11	0.67	0.15	0.20	2.21	0.44	0.52
1.40	0.04	-0.33	0.39	1.60	0.62	0.36
2.28	0.86	0.46	0.23	2.39	0.55	0.40
0.27	-0.77	0.96	0.30	0.42	0.13	1.74
0.67	-0.37	0.63	0.27	0.80	0.22	1.00
0.31	0.93	-0.34	0.35	0.49	0.17	1.27
1.39	0.73	-0.64	0.11	1.44	0.15	1.37
0.46	-0.44	0.60	0.33	0.62	0.20	1.04
0.56	0.87	-0.15	0.29	0.70	0.20	1.02
0.63	0.38	0.05	0.66	0.96	0.63	0.33
0.85	0.22	-0.63	0.25	0.97	0.24	0.85
0.52	-0.76	-0.28	0.54	0.79	0.42	0.48
0.34	-0.60	0.64	0.33	0.50	0.16	1.24
0.47	-0.46	0.26	0.41	0.68	0.28	0.72
1.28	0.51	-0.05	0.25	1.41	0.36	0.56
1.79	0.04	-0.43	0.22	1.90	0.42	0.47
0.65	0.26	-0.23	0.45	0.88	0.40	0.49
0.91	0.78	-0.09	0.22	1.02	0.22	0.87
1.03	-0.35	-0.18	0.79	1.43	1.12	0.17
1.81	0.41	-0.03	0.22	1.93	0.43	0.44
0.67	-0.32	-0.75	0.48	0.91	0.44	0.43
0.83	-0.01	-0.30	0.58	1.12	0.65	0.29
0.40	-0.48	0.33	0.39	0.59	0.23	0.81
0.25	-0.76	0.95	0.28	0.39	0.11	1.71
1.85	0.55	0.17	0.25	1.97	0.48	0.38
1.42	0.28	-0.32	0.20	1.52	0.31	0.60
2.92	-0.16	0.07	0.26	3.04	0.78	0.24
0.83	0.52	-0.36	0.22	0.94	0.21	0.88
0.37	-0.51	0.55	0.32	0.53	0.17	1.07
2.62	0.74	0.57	0.38	2.81	1.06	0.17
0.48	-0.92	0.60	0.20	0.58	0.12	1.51
0.43	-0.92	0.39	0.25	0.55	0.14	1.31
0.77	0.42	0.05	0.49	1.01	0.49	0.36
0.89	-0.47	0.26	0.24	1.01	0.24	0.73

1.16	0.27	0.80	0.26	1.29	0.34	0.53
0.49	0.12	-0.64	0.35	0.66	0.23	0.76
1.79	0.31	0.59	0.32	1.95	0.62	0.28
1.03	0.96	0.30	0.23	1.14	0.26	0.66
0.62	0.01	-0.34	0.55	0.90	0.49	0.35
0.35	-0.27	-0.97	0.44	0.57	0.25	0.70
0.56	-0.13	0.49	0.37	0.74	0.28	0.62
0.88	0.46	-0.08	0.31	1.03	0.32	0.54
0.69	-0.19	-0.79	0.33	0.86	0.29	0.60
0.88	-0.41	-0.07	0.45	1.10	0.50	0.34
3.28	0.27	0.08	0.26	3.41	0.88	0.19
0.29	0.24	-0.79	0.35	0.46	0.16	1.02
0.90	0.51	-0.19	0.23	1.02	0.23	0.70
1.10	-0.69	-0.20	0.27	1.23	0.34	0.49
0.47	0.54	-0.76	0.22	0.58	0.13	1.30
0.64	-0.53	-0.12	0.46	0.87	0.40	0.41
0.46	-0.86	0.15	0.27	0.59	0.16	1.02
2.45	0.61	0.84	0.27	2.59	0.70	0.23
3.76	-0.70	-0.56	0.29	3.91	1.15	0.14
0.98	-0.40	-0.15	0.51	1.24	0.63	0.25
2.74	0.24	0.35	0.48	2.98	1.43	0.11
2.22	0.27	-0.15	0.16	2.30	0.38	0.42
0.58	-0.68	0.05	0.29	0.72	0.21	0.74
0.39	-0.16	-0.65	0.50	0.63	0.31	0.50
1.16	0.58	-0.11	0.18	1.25	0.23	0.69
0.46	0.77	-0.69	0.19	0.55	0.10	1.46
0.63	-0.69	0.38	0.20	0.73	0.14	1.07
0.39	-0.70	-0.12	0.43	0.60	0.26	0.58
0.76	-0.32	-0.01	0.47	1.00	0.47	0.32
5.33	0.28	0.20	0.32	5.49	1.75	0.08
0.39	0.27	-0.77	0.27	0.53	0.14	1.04
1.49	-0.35	0.10	0.20	1.59	0.33	0.45
0.69	0.86	-0.10	0.19	0.79	0.15	0.96
2.00	-0.62	-0.41	0.31	2.16	0.67	0.22
0.53	-0.62	0.08	0.30	0.68	0.20	0.70
0.43	0.00	-0.59	0.39	0.63	0.24	0.59
1.62	-0.04	-0.33	0.28	1.77	0.50	0.28
0.24	-0.86	-0.44	0.61	0.54	0.33	0.42
0.34	-0.75	0.13	0.32	0.50	0.16	0.88
0.91	-0.34	0.37	0.19	1.01	0.19	0.71
0.45	-0.97	-0.31	0.34	0.62	0.21	0.66
0.75	0.39	-0.33	0.22	0.86	0.19	0.72
0.55	-0.39	0.26	0.30	0.70	0.21	0.65
0.36	0.78	0.04	0.34	0.53	0.18	0.74
0.33	-0.48	0.41	0.31	0.49	0.15	0.90
0.28	-0.53	0.46	0.31	0.44	0.14	0.99
0.96	0.45	-0.26	0.18	1.05	0.19	0.71
1.14	-0.05	-0.47	0.25	1.27	0.32	0.42
0.21	0.33	-0.78	0.32	0.37	0.12	1.11
1.00	-0.46	-0.24	0.49	1.25	0.62	0.21
0.58	0.49	-0.35	0.22	0.69	0.15	0.84
0.52	-0.28	-0.70	0.42	0.72	0.30	0.43

2.07	0.49	0.30	0.30	2.22	0.66	0.19
0.84	0.28	-0.23	0.26	0.97	0.25	0.51
0.26	-0.62	0.30	0.33	0.42	0.14	0.92
5.02	0.06	0.15	0.29	5.17	1.48	0.08
0.31	-0.75	0.21	0.29	0.45	0.13	0.97
0.83	-0.86	-0.56	0.41	1.04	0.42	0.29
0.59	-0.56	-0.16	0.39	0.78	0.31	0.40
0.72	0.22	-0.40	0.23	0.84	0.20	0.62
0.37	-0.19	0.58	0.30	0.53	0.16	0.77
0.71	-0.51	-0.63	0.90	1.15	1.03	0.12
0.83	0.17	-0.33	0.25	0.96	0.24	0.50
0.58	-0.59	0.45	0.17	0.67	0.12	1.03
0.16	-0.61	0.79	0.29	0.30	0.09	1.40
0.76	-0.74	-0.07	0.21	0.86	0.18	0.68
1.60	-0.04	0.22	0.27	1.74	0.46	0.26
0.56	-0.79	-0.32	0.35	0.73	0.25	0.47
0.43	0.30	-0.68	0.22	0.54	0.12	0.98
0.39	-0.37	0.49	0.26	0.52	0.14	0.87
0.81	0.15	0.55	0.31	0.96	0.29	0.40
0.41	-0.07	0.88	0.24	0.53	0.12	0.95
0.88	0.90	-0.70	0.08	0.92	0.07	1.60
0.58	-0.06	0.37	0.35	0.75	0.27	0.43
0.23	-0.78	0.46	0.26	0.36	0.09	1.24
0.38	-0.50	0.12	0.34	0.55	0.18	0.62
0.68	-0.01	-0.48	0.29	0.83	0.24	0.47
0.37	-0.86	-0.17	0.31	0.52	0.16	0.69
0.24	0.21	-0.72	0.31	0.39	0.12	0.93
0.41	0.62	-0.13	0.27	0.55	0.15	0.75
0.46	0.68	-0.27	0.21	0.56	0.12	0.95
0.45	-0.01	-0.34	0.49	0.69	0.34	0.32
0.27	-0.28	0.36	0.37	0.45	0.17	0.65
0.59	0.54	-0.29	0.19	0.69	0.13	0.83
0.58	-0.01	-0.47	0.32	0.74	0.23	0.46
0.57	-0.17	0.52	0.23	0.68	0.16	0.69
0.32	-0.72	-0.54	0.81	0.73	0.59	0.18
0.64	-0.76	-0.39	0.36	0.82	0.30	0.36
0.34	-0.82	-0.35	0.42	0.55	0.23	0.46
1.10	0.68	0.38	0.29	1.25	0.36	0.29
0.61	0.30	-0.28	0.25	0.73	0.19	0.57
0.49	0.30	-0.43	0.24	0.61	0.14	0.73
0.64	-0.71	-0.25	0.29	0.79	0.23	0.46
0.39	-0.41	0.08	0.37	0.57	0.21	0.49
0.77	0.33	0.57	0.44	0.99	0.44	0.24
1.88	-0.14	-0.52	0.14	1.95	0.27	0.38
0.36	-0.05	-0.74	0.30	0.51	0.15	0.69
0.29	-0.63	-0.19	0.46	0.52	0.24	0.43
0.27	-0.67	0.00	0.35	0.45	0.16	0.67
0.33	-0.50	0.03	0.37	0.52	0.19	0.53
0.35	0.14	0.81	0.31	0.50	0.15	0.66
0.39	-0.76	-0.21	0.33	0.56	0.19	0.54
2.72	0.28	0.42	0.27	2.85	0.76	0.13
0.43	-0.58	0.01	0.30	0.57	0.17	0.59

0.21	-0.90	-0.36	0.44	0.43	0.19	0.54
0.58	-0.38	0.38	0.19	0.68	0.13	0.76
0.35	0.06	-0.71	0.27	0.49	0.13	0.77
1.11	0.87	-0.18	0.08	1.15	0.09	1.04
0.19	-0.78	0.02	0.34	0.36	0.12	0.80
0.27	-0.63	0.09	0.32	0.43	0.14	0.71
0.74	-0.19	-0.92	0.16	0.82	0.13	0.73
0.45	-0.55	-0.15	0.38	0.63	0.24	0.40
0.65	-0.36	-0.58	0.48	0.90	0.43	0.22
0.36	0.26	-0.30	0.32	0.52	0.17	0.56
2.00	-0.39	-0.23	0.27	2.14	0.58	0.16
0.27	0.65	-0.30	0.25	0.40	0.10	0.95
0.40	-0.10	0.50	0.28	0.54	0.15	0.60
0.18	0.06	-0.72	0.33	0.35	0.12	0.78
0.33	-0.87	-0.22	0.30	0.48	0.14	0.65
0.48	0.32	-0.27	0.25	0.60	0.15	0.60
0.50	-0.10	0.30	0.34	0.67	0.23	0.39
0.79	0.59	0.15	0.22	0.90	0.20	0.45
0.90	-0.48	-0.60	0.61	1.20	0.73	0.12
0.31	-0.58	0.22	0.26	0.44	0.11	0.80
0.61	-0.36	0.09	0.27	0.75	0.20	0.44
0.89	0.19	-0.68	0.11	0.94	0.10	0.87
0.55	0.10	0.53	0.30	0.70	0.21	0.43
0.52	-0.40	-0.59	0.58	0.81	0.47	0.19
0.31	-0.72	-0.18	0.34	0.48	0.16	0.54
2.95	-0.49	-0.37	0.24	3.07	0.74	0.12
0.35	-0.48	0.00	0.35	0.52	0.18	0.48
0.46	-0.14	0.52	0.23	0.58	0.13	0.66
0.52	0.69	0.11	0.24	0.64	0.15	0.57
0.49	-0.18	-0.69	0.27	0.62	0.17	0.51
0.20	0.55	-0.32	0.29	0.35	0.10	0.86
0.18	0.36	-0.89	0.23	0.29	0.07	1.26
4.35	0.32	0.40	0.25	4.48	1.14	0.08
1.76	0.34	0.08	0.18	1.85	0.33	0.26
0.15	0.26	-0.59	0.32	0.31	0.10	0.85
1.26	0.47	-0.16	0.10	1.31	0.13	0.63
0.37	-0.44	0.16	0.28	0.51	0.14	0.60
0.78	-0.08	0.37	0.21	0.88	0.18	0.45
0.96	0.63	0.04	0.14	1.03	0.14	0.59
0.80	0.02	0.34	0.28	0.94	0.26	0.32
0.55	0.02	-0.28	0.37	0.74	0.27	0.30
0.38	-0.36	-0.73	0.38	0.57	0.22	0.38
0.32	0.14	-0.70	0.22	0.43	0.10	0.84
0.64	0.19	0.01	0.51	0.89	0.46	0.17
1.17	0.43	0.25	0.33	1.34	0.44	0.18
1.05	-0.21	0.28	0.14	1.12	0.16	0.49
0.63	0.47	0.09	0.27	0.76	0.21	0.38
0.47	-0.12	0.19	0.39	0.66	0.26	0.30
0.56	0.45	-0.28	0.17	0.64	0.11	0.72
0.41	-0.46	-0.61	0.67	0.75	0.51	0.15
0.41	-0.27	-0.69	0.32	0.57	0.18	0.42
0.44	-0.54	-0.06	0.27	0.58	0.16	0.49

0.28	-0.34	0.27	0.29	0.43	0.13	0.61
2.93	0.33	0.44	0.21	3.03	0.65	0.12
0.38	0.25	-0.36	0.25	0.50	0.13	0.61
0.97	0.10	-0.30	0.18	1.06	0.19	0.40
0.43	0.76	-0.03	0.18	0.52	0.10	0.79
0.68	-0.61	-0.36	0.35	0.85	0.30	0.25
0.56	0.78	-0.16	0.13	0.62	0.08	0.94
0.41	0.63	-0.01	0.23	0.52	0.12	0.64
0.34	-0.44	0.06	0.30	0.49	0.15	0.50
3.56	0.42	0.47	0.38	3.75	1.42	0.05
3.17	0.31	0.38	0.31	3.33	1.05	0.07
0.51	-0.46	-0.24	0.45	0.73	0.33	0.22
1.73	-0.15	-0.25	0.38	1.92	0.73	0.10
0.61	-0.41	-0.69	0.33	0.78	0.26	0.28
0.34	0.01	0.62	0.25	0.47	0.12	0.61
1.61	-0.27	-0.98	0.06	1.64	0.10	0.71
1.06	-0.24	-0.49	0.25	1.18	0.30	0.24
1.34	-0.13	-0.32	0.26	1.47	0.38	0.19
0.51	0.01	-0.19	0.47	0.75	0.35	0.21
0.66	-0.34	-0.42	0.84	1.08	0.91	0.08
0.34	-0.71	-0.28	0.32	0.50	0.16	0.44
0.87	0.63	0.10	0.14	0.94	0.13	0.54
0.51	0.81	0.28	0.21	0.62	0.13	0.53
0.48	-0.39	-0.18	0.47	0.72	0.33	0.21
0.30	-0.70	-0.03	0.25	0.43	0.10	0.67
0.58	-0.66	-0.86	0.45	0.80	0.36	0.20
0.97	0.63	0.38	0.26	1.09	0.28	0.25
0.34	0.52	-0.08	0.25	0.46	0.11	0.60
0.36	-0.55	-0.10	0.30	0.51	0.15	0.45
0.22	-0.24	0.31	0.33	0.38	0.12	0.55
2.06	0.65	0.40	0.13	2.13	0.28	0.25
0.54	-0.53	-0.40	0.63	0.85	0.54	0.13
0.36	-0.56	-0.16	0.33	0.52	0.17	0.40
1.40	-0.02	0.53	0.09	1.44	0.12	0.55
0.69	-0.71	-0.41	0.27	0.83	0.22	0.30
0.69	0.75	0.02	0.13	0.75	0.09	0.72
0.26	-0.36	0.29	0.27	0.39	0.10	0.65
1.15	0.47	0.11	0.15	1.22	0.19	0.36
2.59	0.40	0.49	0.30	2.74	0.82	0.08
1.12	-0.14	0.41	0.10	1.17	0.12	0.55
0.35	-0.58	-0.12	0.29	0.50	0.15	0.46
0.14	0.31	-0.08	0.46	0.37	0.17	0.39
0.30	-0.16	0.42	0.27	0.43	0.11	0.58
0.40	-0.65	-0.06	0.22	0.51	0.11	0.59
0.89	0.80	0.61	0.32	1.05	0.34	0.20
0.22	-0.43	0.29	0.26	0.35	0.09	0.72
0.22	-0.85	-0.39	0.35	0.40	0.14	0.45
0.34	0.71	0.16	0.25	0.47	0.12	0.55
0.35	0.04	-0.56	0.23	0.46	0.11	0.60
0.40	0.69	0.00	0.19	0.49	0.09	0.68
0.18	-0.59	-0.04	0.33	0.35	0.12	0.55
0.74	-0.24	-0.40	0.42	0.95	0.40	0.16

0.24	-0.86	-0.55	0.44	0.46	0.20	0.32
0.28	-0.70	-0.22	0.30	0.43	0.13	0.48
0.31	-0.61	-0.21	0.33	0.48	0.16	0.40
0.11	-0.24	0.52	0.31	0.27	0.08	0.75
0.47	-0.08	-0.50	0.25	0.60	0.15	0.42
1.12	-0.24	-0.36	0.40	1.32	0.53	0.12
0.67	-0.05	-0.49	0.18	0.76	0.14	0.45
0.75	0.75	-0.18	0.08	0.79	0.07	0.94
0.31	-0.48	0.13	0.23	0.43	0.10	0.61
0.72	-0.85	-0.95	0.60	1.02	0.61	0.10
0.98	0.97	0.19	0.08	1.02	0.08	0.78
0.72	-0.45	-0.60	0.41	0.92	0.38	0.16
0.96	0.14	-0.12	0.21	1.06	0.22	0.27
0.34	0.45	-0.08	0.24	0.46	0.11	0.53
0.52	-0.19	-0.25	0.89	0.97	0.87	0.07
2.03	-0.64	-0.54	0.27	2.16	0.58	0.10
0.28	-0.25	-0.64	0.34	0.45	0.15	0.39
1.58	-0.08	0.11	0.18	1.67	0.31	0.19
2.89	0.69	0.64	0.41	3.09	1.27	0.05
0.82	-0.37	0.13	0.13	0.89	0.12	0.50
0.82	-0.35	-0.47	0.46	1.05	0.49	0.12
0.49	-0.22	-0.41	0.41	0.70	0.28	0.20
0.19	-0.69	-0.55	0.72	0.55	0.40	0.14
0.39	-0.19	-0.33	0.58	0.68	0.39	0.14
0.42	-0.09	-0.47	0.26	0.55	0.14	0.38
0.36	-0.70	-0.32	0.28	0.51	0.14	0.38
2.52	-0.30	-0.27	0.59	2.82	1.66	0.03
0.36	0.75	-0.22	0.13	0.43	0.05	0.98
1.14	0.49	0.22	0.16	1.22	0.20	0.26
0.26	0.44	-0.02	0.29	0.40	0.12	0.46
0.18	-0.77	-0.39	0.37	0.37	0.14	0.38
0.37	-0.52	-0.63	0.68	0.71	0.48	0.11
0.16	-0.46	0.34	0.23	0.27	0.06	0.81
0.28	-0.71	-0.32	0.30	0.43	0.13	0.40
3.65	0.36	0.41	0.25	3.77	0.96	0.05
0.26	-0.61	-0.16	0.28	0.41	0.11	0.45
1.40	0.86	0.48	0.09	1.44	0.13	0.38
5.30	0.47	0.51	0.27	5.43	1.47	0.03
1.12	-0.29	-0.19	0.35	1.30	0.46	0.11
0.55	0.66	-0.06	0.11	0.61	0.07	0.72
0.76	0.22	0.33	0.45	0.98	0.44	0.11
0.33	-0.18	-0.48	0.32	0.49	0.16	0.30
0.38	0.33	-0.20	0.19	0.47	0.09	0.53
0.40	-0.44	-0.16	0.30	0.55	0.16	0.29
1.08	-0.40	-0.43	0.94	1.55	1.47	0.03
6.99	-0.03	-0.05	0.48	7.23	3.49	0.01
0.23	-0.53	-0.14	0.30	0.39	0.12	0.40
0.20	-0.18	0.22	0.32	0.36	0.12	0.41
0.28	-0.12	0.27	0.28	0.42	0.12	0.38
2.32	0.10	0.19	0.21	2.43	0.51	0.09
0.91	0.82	0.25	0.08	0.95	0.08	0.57
0.60	-0.49	-0.36	0.39	0.80	0.32	0.14

2.92	-0.20	-0.25	0.29	3.06	0.87	0.05
0.51	-0.60	-0.75	0.39	0.70	0.27	0.16
0.46	0.00	-0.47	0.16	0.54	0.09	0.48
0.62	0.14	-0.20	0.18	0.70	0.12	0.34
2.97	-0.23	-0.16	0.20	3.07	0.60	0.07
0.33	-0.68	-0.39	0.29	0.47	0.14	0.30
0.47	-0.35	-0.61	0.26	0.60	0.15	0.26
0.47	-0.35	-0.61	0.26	0.60	0.15	0.26
0.29	0.12	0.58	0.22	0.40	0.09	0.45
0.79	-0.02	0.14	0.27	0.93	0.25	0.16
0.41	-0.57	-0.87	0.25	0.53	0.13	0.30
0.86	-0.14	-0.30	0.25	0.98	0.24	0.16
0.34	-0.12	0.48	0.15	0.41	0.06	0.60
0.18	-0.23	-0.72	0.25	0.31	0.08	0.50
0.31	-0.25	0.11	0.25	0.43	0.11	0.35
0.43	-0.63	-0.73	0.53	0.70	0.37	0.10
0.97	0.42	0.28	0.24	1.09	0.26	0.14
0.37	0.09	-0.14	0.31	0.53	0.16	0.23
0.30	-0.53	-0.86	0.26	0.43	0.11	0.33
0.76	0.52	0.14	0.11	0.82	0.09	0.38
0.24	-0.57	-0.92	0.27	0.38	0.10	0.35
0.41	-0.21	0.01	0.29	0.56	0.16	0.22
0.16	-0.69	-0.41	0.36	0.35	0.13	0.27
0.32	0.40	-0.52	0.10	0.37	0.04	0.92
0.59	0.32	0.01	0.16	0.67	0.11	0.31
0.47	0.09	0.24	0.35	0.64	0.23	0.15
0.51	0.20	0.36	0.31	0.66	0.20	0.17
0.42	-0.35	-0.16	0.30	0.57	0.17	0.19
0.34	-0.37	0.00	0.20	0.44	0.09	0.37
0.95	0.11	0.04	0.38	1.14	0.43	0.08
1.31	0.32	0.81	0.05	1.34	0.07	0.48
0.41	-0.25	-0.53	0.22	0.52	0.11	0.28
0.09	-0.26	-0.82	0.26	0.22	0.06	0.56
0.42	0.49	0.71	0.26	0.55	0.15	0.22
0.51	-0.26	-0.50	0.22	0.62	0.14	0.24
0.43	0.81	-0.49	0.05	0.46	0.02	1.29
0.12	-0.40	0.07	0.26	0.26	0.07	0.47
0.56	-0.62	-0.40	0.22	0.66	0.15	0.21
0.76	0.13	0.02	0.30	0.91	0.27	0.11
0.33	-0.50	-0.63	0.43	0.54	0.24	0.13
0.48	-0.47	-0.61	0.33	0.64	0.21	0.14
0.81	-0.17	-0.25	0.41	1.01	0.42	0.07
0.58	0.59	0.43	0.25	0.71	0.18	0.17
0.33	-0.39	-0.12	0.24	0.45	0.11	0.27
0.50	-0.34	-0.59	0.20	0.59	0.12	0.25
0.34	-0.36	-0.47	0.46	0.57	0.26	0.11
0.67	0.68	0.32	0.11	0.72	0.08	0.36
0.35	0.25	0.01	0.25	0.47	0.12	0.23
0.76	-0.51	-0.46	0.45	0.99	0.45	0.06
0.43	-0.38	-0.53	0.30	0.58	0.17	0.15
0.29	-0.23	-0.36	0.39	0.48	0.19	0.14
0.51	-0.21	-0.41	0.22	0.62	0.13	0.19

0.68	-0.34	-0.47	0.24	0.80	0.19	0.14
0.42	-0.48	-0.32	0.28	0.57	0.16	0.16
0.41	-0.02	-0.17	0.31	0.57	0.18	0.15
0.38	0.04	0.32	0.19	0.47	0.09	0.28
0.41	-0.47	-0.34	0.35	0.58	0.21	0.12
0.50	-0.24	-0.14	0.36	0.68	0.24	0.10
0.69	0.35	0.29	0.42	0.90	0.38	0.07
0.63	0.01	0.15	0.24	0.75	0.18	0.14
3.57	0.15	0.13	0.35	3.75	1.32	0.02
0.16	-0.74	-0.87	0.47	0.39	0.19	0.13
1.96	-0.40	-0.39	0.75	2.33	1.74	0.01
0.34	-0.55	-0.36	0.27	0.47	0.13	0.19
1.00	-0.49	-0.44	0.45	1.23	0.55	0.04
0.20	-0.23	-0.02	0.32	0.36	0.12	0.20
0.72	-0.71	-0.69	0.94	1.19	1.11	0.02
0.43	-0.14	-0.23	0.42	0.64	0.27	0.09
1.26	-0.01	0.03	0.45	1.48	0.67	0.03
0.29	-0.53	-0.31	0.24	0.42	0.10	0.22
0.18	-0.31	0.00	0.24	0.30	0.07	0.31
0.75	-0.21	-0.37	0.16	0.84	0.14	0.16
0.32	-0.49	-0.32	0.28	0.46	0.13	0.17
0.27	-0.60	-0.39	0.25	0.39	0.10	0.21
0.85	-0.54	-0.64	0.22	0.97	0.21	0.10
0.44	-0.55	-0.67	0.30	0.59	0.17	0.12
0.24	-0.58	-0.40	0.29	0.39	0.11	0.18
0.25	-0.48	-0.66	0.29	0.39	0.11	0.18
0.55	-0.36	-0.47	0.27	0.69	0.19	0.11
0.42	-0.49	-0.31	0.21	0.52	0.11	0.18
0.77	0.23	0.16	0.31	0.92	0.28	0.07
0.38	0.61	0.34	0.16	0.46	0.07	0.27
0.36	-0.52	-0.67	0.25	0.49	0.12	0.16
0.39	-0.15	-0.31	0.23	0.51	0.12	0.16
0.25	-0.26	-0.15	0.38	0.43	0.16	0.11
0.84	0.25	0.38	0.15	0.91	0.14	0.13
0.78	0.43	0.06	0.06	0.81	0.05	0.37
0.41	-0.03	-0.27	0.16	0.49	0.08	0.24
0.44	-0.62	-0.72	0.31	0.60	0.18	0.10
0.50	-0.05	0.12	0.18	0.59	0.10	0.17
0.32	-0.52	-0.72	0.21	0.42	0.09	0.20
0.31	0.00	0.15	0.26	0.44	0.11	0.15
0.35	-0.52	-0.43	0.34	0.52	0.18	0.10
0.54	-0.20	-0.02	0.15	0.61	0.09	0.18
0.67	-0.57	-0.60	0.53	0.94	0.49	0.03
0.81	-0.64	-0.59	0.30	0.97	0.29	0.05
1.64	-0.31	-0.33	0.48	1.88	0.90	0.02
0.35	-0.43	-0.36	0.37	0.54	0.20	0.08
0.43	-0.62	-0.72	0.29	0.58	0.17	0.09
0.81	0.42	-0.34	0.02	0.82	0.02	0.76
0.47	-0.69	-0.79	0.25	0.59	0.15	0.10
0.73	0.08	0.01	0.24	0.85	0.20	0.07
0.85	-0.26	-0.33	0.21	0.96	0.20	0.07
0.43	-0.57	-0.46	0.22	0.54	0.12	0.11

0.61	0.63	0.52	0.16	0.69	0.11	0.12
0.98	0.65	-0.19	0.02	0.99	0.01	0.83
0.97	-0.44	-0.42	0.42	1.18	0.49	0.02
0.86	-0.09	-0.16	0.18	0.95	0.17	0.07
0.58	-0.64	-0.56	0.21	0.69	0.15	0.08
0.14	-0.43	-0.34	0.37	0.32	0.12	0.10
0.36	-0.39	-0.47	0.29	0.51	0.15	0.08
0.40	-0.01	0.07	0.27	0.53	0.15	0.08
0.23	-0.41	-0.53	0.25	0.36	0.09	0.12
0.31	0.66	0.57	0.27	0.45	0.12	0.09
0.33	-0.52	-0.44	0.28	0.46	0.13	0.08
0.19	-0.19	0.00	0.19	0.29	0.05	0.19
0.78	-0.13	-0.17	0.33	0.94	0.31	0.03
0.16	-0.49	-0.60	0.29	0.30	0.09	0.11
0.58	-0.20	-0.42	0.07	0.61	0.04	0.22
0.31	-0.27	-0.34	0.30	0.46	0.14	0.06
0.29	-0.42	-0.49	0.30	0.44	0.13	0.07
0.43	-0.37	-0.33	0.35	0.60	0.21	0.04
0.21	-0.82	-0.73	0.27	0.35	0.09	0.09
0.82	-0.50	-0.52	0.35	1.00	0.35	0.02
0.28	-0.45	-0.52	0.28	0.42	0.12	0.06
0.31	0.13	0.21	0.22	0.42	0.09	0.08
0.65	-0.43	-0.47	0.22	0.76	0.17	0.04
0.68	-0.17	-0.19	0.56	0.96	0.53	0.01
0.22	-0.78	-0.72	0.30	0.37	0.11	0.06
0.45	-0.34	-0.36	0.40	0.65	0.26	0.02
0.67	-0.65	-0.61	0.21	0.77	0.17	0.04
0.51	-0.46	-0.47	0.45	0.74	0.34	0.02
0.23	0.45	0.33	0.16	0.31	0.05	0.11
0.11	-0.29	-0.41	0.21	0.21	0.05	0.11
0.74	-0.54	-0.56	0.36	0.92	0.33	0.01
0.59	-0.29	-0.27	0.39	0.78	0.31	0.01
1.06	-0.19	-0.20	0.35	1.23	0.43	0.01
0.58	-0.59	-0.60	0.33	0.74	0.25	0.01
0.71	-0.45	-0.45	0.47	0.95	0.45	0.01
0.50	0.18	0.20	0.24	0.62	0.15	0.02
0.57	-0.64	-0.64	0.38	0.76	0.29	0.01
0.51	0.56	0.54	0.20	0.61	0.12	0.02
0.47	-0.44	-0.42	0.22	0.58	0.13	0.01
0.31	-0.55	-0.56	0.33	0.48	0.16	0.01
0.55	0.58	0.59	0.23	0.66	0.15	0.01
0.35	-0.44	-0.43	0.24	0.47	0.11	0.01
2.59	0.33	0.33	0.12	2.65	0.31	0.00
0.57	0.17	0.16	0.29	0.72	0.21	0.00
0.88	0.69	-0.43	0.00	0.88	0.00	1.12
0.92	0.53	0.53	0.20	1.03	0.21	0.00
0.24	-0.38	-0.38	0.32	0.40	0.13	0.00
0.19	-0.77	-0.88	0.01	0.19	0.00	0.12
0.40	-0.63	-0.63	0.28	0.54	0.15	0.00
2.54	0.02	0.02	0.51	2.80	1.43	0.00
0.26	0.16	0.16	0.38	0.45	0.17	0.00
0.91	0.24	0.24	-0.52	0.65	-0.34	0.00

0.73	0.33	0.33	-0.22	0.62	-0.13	0.01
2.49	0.25	0.26	-0.11	2.44	-0.27	0.00
0.63	-0.34	-0.36	-0.32	0.47	-0.15	0.02
2.05	0.89	0.89	-0.37	1.87	-0.69	0.01
0.88	0.02	-0.01	-0.26	0.75	-0.19	0.03
0.53	-0.08	-0.01	-0.32	0.37	-0.12	0.06
0.62	-0.13	-0.06	-0.22	0.51	-0.11	0.07
0.65	-0.17	-0.09	-0.20	0.55	-0.11	0.07
0.54	-0.25	-0.34	-0.23	0.43	-0.10	0.09
0.62	-0.74	-0.84	-0.17	0.54	-0.09	0.10
0.56	0.23	0.15	-0.32	0.40	-0.13	0.07
0.32	-0.01	-0.28	-0.14	0.25	-0.04	0.27
1.07	0.06	-0.01	-0.19	0.97	-0.19	0.06
1.83	0.09	-0.02	-0.06	1.80	-0.11	0.11
2.81	-0.01	-0.04	-0.21	2.70	-0.57	0.02
0.86	-0.16	-0.22	-0.29	0.71	-0.21	0.06
0.43	-0.37	-0.60	-0.20	0.33	-0.07	0.23
1.75	0.46	0.47	-0.67	1.41	-0.94	0.02
1.59	0.15	0.11	-0.23	1.48	-0.34	0.05
0.58	-0.32	-0.17	-0.23	0.46	-0.11	0.15
0.86	-0.57	-0.74	-0.13	0.79	-0.10	0.17
0.70	0.19	0.46	-0.11	0.65	-0.07	0.27
1.24	0.35	0.22	-0.13	1.18	-0.15	0.13
1.24	0.35	0.22	-0.13	1.18	-0.15	0.13
0.57	-0.37	-0.16	-0.21	0.47	-0.10	0.21
0.67	0.02	-0.80	-0.04	0.65	-0.03	0.81
1.39	0.55	0.49	-0.33	1.23	-0.41	0.05
1.14	-0.11	-0.07	-0.51	0.89	-0.45	0.05
1.63	0.89	0.55	-0.04	1.61	-0.07	0.34
1.48	0.02	-0.15	-0.09	1.44	-0.13	0.17
0.84	0.07	0.22	-0.21	0.74	-0.15	0.15
0.47	-0.62	-0.96	-0.18	0.38	-0.07	0.34
0.95	0.06	-0.03	-0.32	0.79	-0.25	0.09
1.90	0.01	-0.03	-0.31	1.74	-0.55	0.05
1.73	0.55	0.45	-0.15	1.65	-0.26	0.10
2.15	0.21	0.15	-0.20	2.05	-0.40	0.06
0.99	0.41	0.50	-0.33	0.83	-0.27	0.09
0.47	-0.08	-0.41	-0.22	0.36	-0.08	0.33
1.00	0.79	0.67	-0.23	0.88	-0.21	0.13
0.81	-0.04	-0.16	-0.36	0.63	-0.23	0.12
1.87	0.78	0.82	-0.37	1.69	-0.63	0.04
0.55	-0.41	-0.16	-0.26	0.42	-0.11	0.24
0.82	-0.02	-0.20	-0.22	0.71	-0.15	0.17
0.72	-0.30	-0.13	-0.28	0.58	-0.16	0.17
1.05	-0.93	-0.83	-0.31	0.89	-0.28	0.10
0.61	0.14	-0.03	-0.37	0.43	-0.16	0.18
0.94	0.27	0.35	-0.51	0.68	-0.35	0.08
0.45	0.38	0.04	-0.27	0.32	-0.08	0.34
1.46	0.29	0.38	-0.26	1.33	-0.34	0.09
0.82	-0.28	-0.13	-0.33	0.65	-0.21	0.14
0.79	0.34	0.51	-0.27	0.65	-0.18	0.17
0.42	0.06	0.52	-0.23	0.30	-0.07	0.46

0.69	0.20	0.03	-0.37	0.51	-0.19	0.17
3.31	0.26	0.22	-0.28	3.17	-0.88	0.04
0.54	-0.62	0.07	-0.10	0.49	-0.05	0.69
0.68	0.55	0.38	-0.42	0.47	-0.20	0.18
0.89	0.08	-0.15	-0.20	0.79	-0.16	0.22
0.89	0.08	-0.15	-0.20	0.79	-0.16	0.22
0.57	0.48	-0.01	-0.15	0.50	-0.07	0.50
1.74	0.44	0.36	-0.30	1.59	-0.48	0.08
1.19	0.11	-0.01	-0.34	1.03	-0.35	0.12
0.91	-0.03	-0.41	-0.13	0.84	-0.11	0.38
0.55	0.18	-0.21	-0.26	0.42	-0.11	0.39
0.94	-0.23	-0.04	-0.28	0.80	-0.22	0.19
0.86	0.10	-0.06	-0.42	0.65	-0.28	0.15
0.62	0.75	0.46	-0.31	0.46	-0.14	0.30
1.03	0.15	-0.09	-0.20	0.93	-0.18	0.24
0.81	-0.02	0.24	-0.25	0.68	-0.17	0.26
0.54	-0.59	-0.17	-0.26	0.41	-0.11	0.43
0.43	0.45	-0.27	-0.21	0.32	-0.07	0.72
0.67	0.37	0.10	-0.37	0.48	-0.18	0.28
0.66	0.46	0.80	-0.29	0.51	-0.15	0.34
1.01	-0.30	-0.06	-0.24	0.89	-0.21	0.24
0.88	0.40	0.22	-0.43	0.67	-0.28	0.18
1.06	0.44	-0.22	-0.08	1.03	-0.08	0.66
0.91	0.33	0.56	-0.29	0.76	-0.22	0.23
0.75	-0.21	0.00	-0.50	0.50	-0.25	0.21
0.41	0.82	0.08	-0.26	0.28	-0.07	0.74
0.97	0.56	0.36	-0.34	0.80	-0.27	0.20
0.72	-0.49	-0.16	-0.29	0.57	-0.17	0.33
1.63	0.46	0.29	-0.22	1.52	-0.34	0.17
4.83	-0.02	0.04	-0.20	4.73	-0.92	0.06
1.57	0.15	0.05	-0.42	1.36	-0.57	0.10
1.28	0.66	0.34	-0.15	1.20	-0.19	0.32
0.57	-0.12	0.53	-0.20	0.47	-0.09	0.65
0.51	0.10	0.67	-0.32	0.35	-0.11	0.56
0.60	0.67	-0.03	-0.18	0.51	-0.09	0.70
1.96	0.72	-0.40	-0.03	1.95	-0.06	1.13
0.67	0.52	0.15	-0.35	0.50	-0.18	0.37
0.71	0.46	-0.18	-0.16	0.63	-0.10	0.64
0.52	0.48	-0.08	-0.33	0.36	-0.12	0.56
0.76	0.45	0.08	-0.30	0.61	-0.18	0.37
1.67	0.40	0.23	-0.26	1.54	-0.40	0.17
0.60	0.51	0.08	-0.40	0.41	-0.16	0.42
0.70	-0.76	-0.32	-0.28	0.56	-0.16	0.43
1.06	0.84	0.61	-0.34	0.89	-0.30	0.23
3.10	0.13	0.22	-0.26	2.97	-0.78	0.09
0.66	0.54	0.13	-0.36	0.48	-0.17	0.41
2.92	0.83	0.73	-0.26	2.79	-0.72	0.10
0.79	-0.05	-0.96	-0.11	0.74	-0.08	0.91
1.12	0.39	0.21	-0.44	0.90	-0.40	0.18
0.43	-0.89	0.29	-0.19	0.34	-0.06	1.18
0.79	0.07	-0.34	-0.28	0.65	-0.18	0.42
0.79	0.18	-0.31	-0.23	0.68	-0.16	0.49

0.95	0.88	0.53	-0.27	0.82	-0.22	0.35
0.68	0.27	-0.13	-0.40	0.48	-0.19	0.40
0.94	-0.34	0.12	-0.20	0.84	-0.17	0.47
3.23	0.78	0.69	-0.28	3.09	-0.85	0.09
0.87	0.26	-0.02	-0.44	0.65	-0.28	0.28
0.65	0.53	-0.06	-0.26	0.52	-0.14	0.59
0.62	0.51	-0.16	-0.24	0.50	-0.12	0.67
0.81	0.72	0.36	-0.36	0.63	-0.23	0.36
1.96	0.57	0.24	-0.13	1.89	-0.25	0.33
1.96	0.57	0.24	-0.13	1.89	-0.25	0.33
0.47	-0.49	0.34	-0.31	0.32	-0.10	0.83
0.72	-0.15	0.49	-0.21	0.61	-0.13	0.64
0.57	-0.07	-0.87	-0.22	0.46	-0.10	0.80
0.90	0.78	0.38	-0.27	0.76	-0.21	0.40
0.57	0.67	0.09	-0.39	0.37	-0.15	0.58
0.52	0.78	-0.01	-0.29	0.38	-0.11	0.79
0.67	0.44	0.02	-0.47	0.44	-0.20	0.42
3.59	0.15	0.28	-0.20	3.49	-0.71	0.12
1.72	0.27	-0.02	-0.18	1.63	-0.30	0.30
0.68	0.41	-0.15	-0.31	0.53	-0.16	0.56
0.54	0.94	0.07	-0.26	0.41	-0.11	0.87
0.57	0.45	-0.28	-0.31	0.42	-0.13	0.73
0.65	0.47	-0.21	-0.26	0.52	-0.14	0.68
0.62	0.61	0.04	-0.37	0.44	-0.16	0.58
0.65	0.29	-0.21	-0.43	0.43	-0.19	0.50
0.63	0.63	-0.03	-0.29	0.49	-0.14	0.66
0.71	0.89	-0.31	-0.12	0.65	-0.08	1.20
0.60	0.43	-0.49	-0.21	0.49	-0.10	0.92
0.78	0.08	0.51	-0.39	0.58	-0.23	0.42
0.55	0.70	-0.18	-0.26	0.42	-0.11	0.88
0.96	0.43	0.88	-0.26	0.83	-0.21	0.45
0.72	0.46	-0.90	-0.11	0.67	-0.07	1.36
0.47	0.73	-0.29	-0.29	0.32	-0.10	1.02
0.78	0.36	-0.07	-0.40	0.58	-0.23	0.42
0.59	0.61	-0.09	-0.33	0.43	-0.14	0.71
0.66	0.49	-0.10	-0.35	0.48	-0.17	0.59
0.78	0.74	0.23	-0.31	0.63	-0.19	0.51
0.72	-0.48	0.10	-0.30	0.57	-0.17	0.59
1.32	0.07	0.30	-0.38	1.13	-0.43	0.24
1.52	0.15	0.32	-0.46	1.29	-0.60	0.17
0.49	-0.95	0.20	-0.25	0.36	-0.09	1.15
1.59	0.89	0.67	-0.33	1.42	-0.47	0.22
0.76	0.42	-0.20	-0.28	0.62	-0.17	0.61
0.53	0.88	-0.08	-0.29	0.38	-0.11	0.97
0.51	0.25	-0.94	-0.23	0.40	-0.09	1.18
3.67	0.29	0.17	-0.24	3.55	-0.86	0.13
0.65	-0.90	0.09	-0.21	0.54	-0.11	0.98
0.61	0.04	-0.85	-0.25	0.49	-0.12	0.89
0.98	0.12	-0.27	-0.35	0.80	-0.28	0.39
2.65	0.25	0.09	-0.27	2.51	-0.68	0.16
1.05	0.54	0.17	-0.35	0.88	-0.31	0.37
0.64	0.83	0.16	-0.39	0.45	-0.17	0.67

2.56	-0.22	0.03	-0.19	2.47	-0.46	0.25
0.76	0.64	0.15	-0.43	0.54	-0.23	0.50
0.65	0.54	-0.72	-0.16	0.57	-0.09	1.26
0.92	0.49	-0.07	-0.28	0.78	-0.22	0.56
0.61	0.71	-0.29	-0.27	0.47	-0.13	0.99
0.91	0.66	0.15	-0.33	0.74	-0.25	0.50
1.01	-0.19	-0.69	-0.29	0.86	-0.25	0.51
0.56	-0.75	0.44	-0.24	0.44	-0.11	1.19
1.21	0.62	0.18	-0.27	1.07	-0.29	0.44
1.18	0.39	-0.17	-0.21	1.08	-0.23	0.55
0.63	0.81	-0.09	-0.31	0.47	-0.15	0.90
0.65	0.50	-0.40	-0.30	0.50	-0.15	0.90
0.65	-0.46	0.53	-0.27	0.51	-0.14	0.98
0.57	0.53	-0.47	-0.34	0.40	-0.14	1.00
0.74	0.64	-0.29	-0.24	0.62	-0.15	0.92
0.71	0.25	-0.54	-0.32	0.55	-0.17	0.79
0.86	0.45	-0.60	-0.17	0.77	-0.13	1.05
0.89	0.42	-0.18	-0.32	0.73	-0.23	0.59
0.65	-0.92	0.13	-0.25	0.52	-0.13	1.06
0.69	-0.58	0.14	-0.38	0.50	-0.19	0.73
0.93	0.58	-0.09	-0.26	0.80	-0.21	0.67
1.19	0.36	0.64	-0.55	0.91	-0.50	0.28
1.11	0.25	-0.13	-0.43	0.90	-0.38	0.37
1.02	0.74	0.20	-0.31	0.87	-0.27	0.55
0.63	0.85	-0.38	-0.24	0.51	-0.12	1.23
1.28	0.07	-0.49	-0.23	1.16	-0.27	0.56
1.10	0.70	0.05	-0.24	0.98	-0.24	0.64
0.59	0.77	-0.43	-0.29	0.44	-0.13	1.20
0.70	0.68	-0.32	-0.27	0.57	-0.15	1.00
0.90	0.48	-0.17	-0.33	0.73	-0.24	0.65
1.95	0.91	0.66	-0.36	1.77	-0.64	0.25
0.68	0.74	-0.45	-0.23	0.57	-0.13	1.19
0.99	0.60	-0.45	-0.17	0.90	-0.15	1.05
0.71	0.76	-0.18	-0.30	0.56	-0.17	0.94
0.97	0.77	0.28	-0.43	0.76	-0.32	0.49
0.64	0.61	-0.41	-0.33	0.48	-0.16	1.02
0.93	0.45	-0.70	-0.17	0.85	-0.14	1.15
1.72	0.42	-0.15	-0.18	1.64	-0.29	0.58
0.55	0.77	-0.65	-0.29	0.41	-0.12	1.42
0.82	-0.41	0.43	-0.30	0.67	-0.20	0.85
1.34	0.31	-0.09	-0.37	1.16	-0.43	0.40
0.69	0.46	-0.38	-0.42	0.48	-0.20	0.84
0.93	-0.46	0.14	-0.40	0.73	-0.29	0.60
1.06	0.77	0.27	-0.41	0.86	-0.35	0.50
1.57	0.92	0.47	-0.27	1.44	-0.39	0.45
0.81	0.65	-0.59	-0.20	0.71	-0.14	1.24
1.01	0.41	-0.63	-0.19	0.92	-0.17	1.04
2.64	0.46	0.17	-0.25	2.52	-0.62	0.29
0.69	0.90	-0.50	-0.23	0.57	-0.13	1.39
0.97	0.71	0.15	-0.44	0.75	-0.33	0.56
0.81	0.62	-0.93	-0.17	0.73	-0.12	1.55
1.03	0.78	0.25	-0.44	0.81	-0.36	0.53

0.96	0.45	-0.25	-0.34	0.79	-0.27	0.70
0.95	0.46	-0.26	-0.34	0.78	-0.27	0.72
0.94	0.54	-0.56	-0.21	0.84	-0.18	1.10
1.40	0.28	-0.10	-0.43	1.19	-0.51	0.38
0.78	0.72	-0.99	-0.16	0.70	-0.11	1.71
0.82	0.76	-0.59	-0.20	0.72	-0.14	1.35
1.78	0.78	0.49	-0.43	1.56	-0.67	0.29
0.57	0.78	-0.86	-0.28	0.43	-0.12	1.64
0.72	0.46	-0.77	-0.28	0.58	-0.16	1.24
1.28	0.59	-0.22	-0.21	1.17	-0.25	0.80
2.66	0.78	0.52	-0.30	2.51	-0.75	0.27
0.84	0.71	-0.19	-0.33	0.68	-0.22	0.89
2.91	0.09	-0.39	-0.15	2.84	-0.42	0.47
0.72	0.80	-0.25	-0.35	0.55	-0.19	1.05
2.86	0.79	0.59	-0.38	2.68	-1.01	0.20
2.03	0.36	-0.27	-0.17	1.95	-0.32	0.63
3.30	0.36	0.15	-0.30	3.15	-0.95	0.21
1.23	0.81	0.21	-0.32	1.06	-0.34	0.59
0.86	-0.14	0.71	-0.35	0.68	-0.24	0.85
2.31	-0.26	-0.51	-0.39	2.12	-0.82	0.25
1.33	0.46	-0.03	-0.36	1.15	-0.42	0.50
1.37	0.21	-0.42	-0.27	1.23	-0.33	0.63
0.76	0.75	-0.26	-0.35	0.58	-0.20	1.01
0.62	0.49	-0.80	-0.38	0.43	-0.16	1.29
1.08	0.98	0.11	-0.26	0.95	-0.24	0.86
0.81	-0.43	0.38	-0.44	0.59	-0.26	0.82
1.10	0.81	0.28	-0.46	0.87	-0.40	0.54
0.67	0.77	-0.67	-0.28	0.52	-0.15	1.44
0.76	0.50	-0.74	-0.28	0.62	-0.17	1.25
1.08	-0.02	-0.99	-0.23	0.97	-0.22	0.97
0.64	0.76	-0.88	-0.27	0.50	-0.13	1.64
2.09	0.43	0.74	-0.38	1.89	-0.73	0.30
0.81	0.73	-0.33	-0.32	0.65	-0.21	1.06
2.46	0.95	0.55	-0.24	2.34	-0.56	0.40
1.49	0.18	-0.40	-0.29	1.35	-0.38	0.59
1.02	0.49	-0.39	-0.30	0.87	-0.26	0.88
1.23	0.36	-0.29	-0.33	1.06	-0.35	0.65
1.32	0.52	-0.58	-0.17	1.24	-0.21	1.10
0.94	0.74	-0.09	-0.37	0.76	-0.28	0.83
1.49	0.39	-0.07	-0.39	1.30	-0.50	0.47
2.23	0.63	0.23	-0.28	2.10	-0.58	0.40
1.82	0.46	0.17	-0.52	1.56	-0.82	0.29
0.77	0.70	-0.83	-0.24	0.65	-0.16	1.53
0.80	0.81	-0.38	-0.32	0.64	-0.20	1.19
1.09	0.45	-0.55	-0.25	0.96	-0.24	1.00
1.01	0.89	-0.03	-0.31	0.86	-0.27	0.92
2.09	0.65	0.33	-0.42	1.88	-0.79	0.31
0.67	-0.95	0.43	-0.36	0.49	-0.18	1.39
0.75	-0.74	0.76	-0.27	0.61	-0.17	1.50
1.05	0.72	-0.22	-0.30	0.90	-0.27	0.94
0.99	0.95	0.08	-0.36	0.81	-0.29	0.86
0.82	0.61	-0.41	-0.40	0.62	-0.25	1.02

1.32	0.85	-0.18	-0.20	1.21	-0.25	1.02
1.78	0.84	0.38	-0.34	1.61	-0.55	0.46
0.69	0.82	-0.73	-0.31	0.54	-0.17	1.55
1.02	0.71	-0.26	-0.31	0.87	-0.27	0.98
0.79	-0.61	0.42	-0.47	0.56	-0.26	1.03
1.04	0.58	-0.28	-0.36	0.86	-0.31	0.87
0.81	0.79	-0.72	-0.27	0.68	-0.18	1.51
1.95	0.46	0.08	-0.41	1.75	-0.72	0.38
1.06	0.78	-0.46	-0.24	0.94	-0.22	1.24
1.59	0.56	0.10	-0.44	1.37	-0.61	0.46
1.64	0.71	-0.40	-0.16	1.56	-0.25	1.11
1.85	0.16	-0.69	-0.19	1.75	-0.33	0.85
0.95	0.33	-0.59	-0.42	0.74	-0.31	0.92
0.90	0.66	-0.35	-0.40	0.70	-0.28	1.01
1.58	0.79	0.25	-0.39	1.39	-0.54	0.53
1.32	0.65	-0.22	-0.28	1.18	-0.34	0.87
0.71	0.76	-0.76	-0.36	0.53	-0.19	1.52
0.97	0.78	-0.26	-0.36	0.79	-0.28	1.04
1.15	0.65	0.01	-0.52	0.89	-0.46	0.64
1.08	0.93	0.04	-0.38	0.89	-0.34	0.89
0.97	0.65	-0.40	-0.36	0.79	-0.29	1.05
0.96	0.97	-0.61	-0.22	0.84	-0.19	1.58
1.36	0.74	-0.11	-0.29	1.22	-0.35	0.85
2.63	0.71	0.36	-0.35	2.46	-0.87	0.35
1.18	0.61	-0.53	-0.25	1.05	-0.26	1.15
1.43	0.52	0.04	-0.56	1.15	-0.64	0.48
1.25	0.97	-0.03	-0.28	1.11	-0.31	1.00
1.32	0.63	-0.47	-0.23	1.20	-0.28	1.10
1.11	0.57	-0.35	-0.36	0.92	-0.34	0.92
3.84	0.26	0.02	-0.37	3.65	-1.34	0.23
1.24	0.05	0.60	-0.60	0.94	-0.56	0.55
4.48	0.00	-0.98	-0.07	4.45	-0.32	0.99
1.02	0.61	-0.65	-0.29	0.88	-0.25	1.26
1.72	0.41	-0.70	-0.18	1.64	-0.29	1.11
1.43	0.92	0.30	-0.43	1.22	-0.52	0.62
0.83	0.76	-0.75	-0.32	0.66	-0.22	1.51
3.43	0.97	0.57	-0.25	3.31	-0.82	0.40
1.98	0.86	0.32	-0.33	1.81	-0.60	0.55
3.79	0.33	0.82	-0.18	3.70	-0.68	0.49
2.26	0.50	0.33	-1.17	1.68	-1.97	0.17
0.95	0.81	-0.37	-0.37	0.76	-0.29	1.18
0.99	0.59	-0.71	-0.32	0.83	-0.26	1.30
0.93	0.78	-0.44	-0.38	0.75	-0.28	1.22
0.94	-0.65	0.29	-0.55	0.67	-0.37	0.94
1.18	0.96	-0.08	-0.33	1.02	-0.34	1.04
1.08	0.79	-0.50	-0.29	0.94	-0.28	1.29
1.14	0.71	-0.55	-0.29	0.99	-0.28	1.26
1.19	0.55	-0.68	-0.28	1.05	-0.30	1.23
3.86	0.74	0.38	-0.27	3.72	-1.02	0.36
1.04	0.66	-0.44	-0.41	0.84	-0.34	1.10
0.95	0.86	-0.94	-0.26	0.82	-0.21	1.80
2.12	0.72	-0.05	-0.25	1.99	-0.50	0.76

5.33	0.35	-0.26	-0.12	5.27	-0.62	0.61
1.62	0.92	0.25	-0.41	1.41	-0.58	0.67
1.32	0.79	-0.75	-0.21	1.22	-0.25	1.54
1.18	0.57	-0.25	-0.52	0.92	-0.47	0.81
0.88	0.69	-0.51	-0.52	0.63	-0.32	1.20
1.24	0.65	-0.35	-0.37	1.05	-0.39	1.01
1.97	0.62	-0.71	-0.16	1.89	-0.29	1.33
3.09	0.89	0.47	-0.32	2.93	-0.94	0.42
1.38	0.67	-0.51	-0.27	1.24	-0.34	1.18
5.12	0.98	0.62	-0.22	5.01	-1.12	0.36
0.98	0.75	-0.75	-0.33	0.81	-0.27	1.50
2.32	0.62	-0.42	-0.18	2.23	-0.40	1.04
3.11	0.71	-0.34	-0.13	3.04	-0.41	1.05
2.04	0.61	-0.16	-0.30	1.89	-0.56	0.77
1.22	0.77	-0.53	-0.31	1.06	-0.33	1.31
1.29	0.83	-0.17	-0.40	1.09	-0.43	1.01
4.66	0.90	0.53	-0.26	4.53	-1.20	0.36
1.41	0.81	-0.57	-0.25	1.28	-0.32	1.39
2.44	0.69	0.00	-0.28	2.30	-0.64	0.69
1.85	0.87	-0.43	-0.19	1.76	-0.34	1.31
1.29	0.88	-0.33	-0.33	1.13	-0.37	1.21
1.22	-0.59	0.51	-0.40	1.02	-0.41	1.10
2.31	0.79	-0.13	-0.22	2.20	-0.48	0.93
1.72	0.75	-0.51	-0.22	1.61	-0.36	1.26
1.62	0.88	-0.28	-0.26	1.49	-0.39	1.16
2.05	0.95	0.01	-0.25	1.93	-0.48	0.94
1.08	0.88	-0.44	-0.39	0.88	-0.34	1.32
1.29	0.86	-0.42	-0.32	1.14	-0.36	1.27
3.76	0.66	-0.34	-0.12	3.70	-0.46	1.00
1.86	0.85	0.16	-0.41	1.65	-0.68	0.69
3.98	0.67	-0.13	-0.15	3.90	-0.59	0.80
2.43	0.81	-0.04	-0.24	2.31	-0.57	0.84
1.83	-0.13	0.77	-0.32	1.67	-0.54	0.90
0.99	0.87	-0.64	-0.41	0.79	-0.32	1.52
2.08	0.76	0.33	-0.64	1.76	-1.13	0.43
3.34	0.88	0.23	-0.23	3.22	-0.75	0.65
1.30	0.92	-0.83	-0.24	1.18	-0.28	1.75
1.60	0.81	-0.69	-0.22	1.49	-0.33	1.50
1.55	0.88	-0.43	-0.27	1.42	-0.38	1.31
1.45	0.58	-0.38	-0.42	1.24	-0.52	0.96
1.85	0.97	-0.13	-0.27	1.72	-0.46	1.10
2.66	0.44	-0.36	-0.25	2.54	-0.63	0.81
1.99	0.88	-0.39	-0.21	1.88	-0.40	1.27
2.97	0.67	0.20	-0.39	2.78	-1.09	0.47
1.39	0.59	-0.46	-0.41	1.19	-0.49	1.06
1.84	0.71	-0.68	-0.21	1.73	-0.37	1.38
2.00	0.78	-0.24	-0.27	1.87	-0.51	1.02
0.98	0.76	-0.77	-0.44	0.76	-0.34	1.53
1.52	0.83	0.04	-0.51	1.26	-0.65	0.80
3.05	0.62	-0.53	-0.15	2.97	-0.45	1.15
2.85	0.48	-0.82	-0.14	2.77	-0.40	1.29
1.64	0.89	-0.28	-0.30	1.49	-0.44	1.17

6.03	-0.33	-0.06	-0.32	5.87	-1.89	0.28
1.32	0.98	-0.64	-0.27	1.18	-0.32	1.62
1.32	-0.50	0.74	-0.38	1.13	-0.43	1.23
1.14	0.81	-0.39	-0.50	0.89	-0.44	1.20
2.24	0.90	-0.20	-0.23	2.13	-0.48	1.10
1.83	0.86	-0.10	-0.33	1.66	-0.55	0.96
2.35	0.78	-0.14	-0.26	2.22	-0.57	0.92
2.60	0.81	-0.26	-0.20	2.50	-0.50	1.07
3.67	0.66	-0.33	-0.15	3.59	-0.54	0.99
1.90	0.84	0.14	-0.46	1.67	-0.78	0.70
1.84	0.77	0.01	-0.45	1.62	-0.73	0.76
1.93	0.64	-0.66	-0.24	1.81	-0.43	1.30
2.99	0.81	-0.08	-0.22	2.88	-0.63	0.89
1.75	0.94	-0.03	-0.37	1.56	-0.58	0.98
1.34	0.87	-0.42	-0.38	1.15	-0.44	1.28
2.12	0.32	-0.21	-0.59	1.82	-1.07	0.53
2.05	0.08	0.56	-0.70	1.70	-1.18	0.49
2.31	0.71	-0.41	-0.24	2.19	-0.52	1.12
2.48	0.87	-0.20	-0.23	2.37	-0.54	1.06
1.74	0.89	-0.09	-0.38	1.55	-0.60	0.98
3.69	0.88	0.36	-0.32	3.53	-1.12	0.52
5.02	0.97	0.43	-0.22	4.91	-1.08	0.54
1.49	0.83	-0.53	-0.33	1.33	-0.44	1.36
2.95	0.40	-0.61	-0.21	2.85	-0.59	1.01
2.07	0.82	-0.18	-0.31	1.92	-0.60	0.99
1.84	0.57	0.07	-0.82	1.43	-1.18	0.51
2.29	0.78	-0.11	-0.32	2.13	-0.68	0.89
1.85	0.87	-0.59	-0.24	1.73	-0.41	1.47
1.72	0.75	-0.42	-0.33	1.56	-0.52	1.17
2.59	0.95	0.10	-0.29	2.45	-0.71	0.85
1.60	0.77	-0.76	-0.28	1.46	-0.41	1.53
2.57	0.69	-0.27	-0.27	2.44	-0.65	0.96
3.17	0.83	-0.27	-0.18	3.08	-0.57	1.10
1.46	0.88	-0.49	-0.36	1.28	-0.46	1.37
3.95	0.68	-0.39	-0.15	3.87	-0.59	1.07
3.94	0.86	-0.21	-0.15	3.86	-0.59	1.07
2.34	0.81	-0.25	-0.28	2.20	-0.61	1.06
6.08	0.85	0.22	-0.17	5.99	-1.03	0.63
1.72	0.83	-0.56	-0.30	1.57	-0.47	1.39
2.45	0.95	-0.17	-0.25	2.33	-0.58	1.13
1.50	0.76	-0.83	-0.31	1.35	-0.42	1.60
2.64	0.88	0.03	-0.31	2.49	-0.78	0.86
7.65	0.95	0.54	-0.21	7.54	-1.61	0.42
2.08	0.76	-0.44	-0.29	1.94	-0.57	1.20
2.04	0.70	0.10	-0.67	1.70	-1.14	0.60
2.00	0.81	-0.58	-0.26	1.87	-0.49	1.38
2.40	0.73	0.23	-0.66	2.07	-1.37	0.50
2.30	0.93	-0.19	-0.29	2.16	-0.62	1.12
3.22	0.53	-0.21	-0.31	3.07	-0.94	0.74
2.93	0.67	-0.64	-0.19	2.84	-0.54	1.30
1.80	0.89	-0.43	-0.33	1.63	-0.54	1.32
2.71	0.52	-0.03	-0.54	2.44	-1.31	0.55

3.65	0.75	-0.05	-0.26	3.53	-0.90	0.80
2.56	0.39	-0.45	-0.36	2.38	-0.86	0.84
1.81	0.83	-0.46	-0.35	1.63	-0.57	1.29
5.26	0.72	-0.04	-0.19	5.16	-0.96	0.76
2.21	0.73	-0.87	-0.22	2.10	-0.46	1.60
3.99	0.95	0.43	-0.38	3.80	-1.46	0.51
1.70	0.75	-0.48	-0.40	1.50	-0.61	1.23
3.54	0.87	0.18	-0.32	3.38	-1.08	0.69
5.29	0.84	0.44	-0.36	5.11	-1.85	0.41
4.41	0.72	0.34	-0.48	4.17	-1.98	0.38
1.47	0.82	-0.58	-0.43	1.26	-0.54	1.40
1.40	0.83	-0.99	-0.34	1.23	-0.42	1.82
1.18	-0.75	0.90	-0.51	0.93	-0.47	1.65
4.37	0.87	-0.16	-0.18	4.28	-0.75	1.03
2.05	0.61	-0.40	-0.42	1.84	-0.77	1.02
2.30	0.60	-0.56	-0.32	2.14	-0.68	1.17
1.64	0.84	-0.71	-0.35	1.47	-0.51	1.55
2.26	0.96	-0.32	-0.29	2.12	-0.62	1.28
3.91	0.78	-0.45	-0.17	3.82	-0.65	1.23
1.96	0.83	-0.64	-0.30	1.81	-0.54	1.48
5.01	0.84	-0.18	-0.16	4.93	-0.79	1.02
2.98	0.92	-0.34	-0.22	2.87	-0.64	1.26
2.35	0.69	-0.49	-0.31	2.19	-0.69	1.18
3.49	0.75	0.07	-0.37	3.31	-1.22	0.68
2.97	0.88	0.08	-0.37	2.79	-1.03	0.80
2.41	0.90	-0.44	-0.27	2.27	-0.62	1.34
3.09	0.95	-0.29	-0.23	2.97	-0.67	1.24
2.77	0.82	-0.34	-0.27	2.63	-0.72	1.17
5.75	0.94	0.53	-0.38	5.56	-2.10	0.41
3.95	0.90	0.24	-0.34	3.78	-1.28	0.67
2.31	-0.53	0.29	-0.51	2.05	-1.04	0.82
4.06	0.91	-0.23	-0.19	3.96	-0.76	1.14
2.45	0.93	-0.29	-0.31	2.29	-0.71	1.23
3.17	0.48	-0.01	-0.62	2.87	-1.77	0.49
2.75	0.93	-0.61	-0.22	2.64	-0.58	1.54
3.15	0.95	0.34	-0.51	2.90	-1.47	0.61
1.82	0.74	-0.63	-0.41	1.62	-0.66	1.37
2.08	0.76	-0.37	-0.43	1.86	-0.81	1.13
4.10	0.84	-0.39	-0.19	4.01	-0.75	1.23
2.06	0.62	-0.75	-0.36	1.89	-0.67	1.38
6.55	0.97	0.25	-0.20	6.44	-1.30	0.71
6.72	0.94	0.25	-0.21	6.62	-1.38	0.69
2.74	0.53	-0.84	-0.27	2.60	-0.70	1.37
3.74	0.83	-0.32	-0.23	3.63	-0.85	1.14
4.06	0.97	-0.18	-0.22	3.96	-0.85	1.14
2.57	0.69	-0.26	-0.44	2.35	-1.03	0.95
3.15	0.93	-0.16	-0.30	3.00	-0.90	1.09
3.20	0.84	-0.36	-0.27	3.07	-0.82	1.20
7.56	0.91	0.32	-0.22	7.45	-1.66	0.60
4.11	0.82	-0.01	-0.30	3.96	-1.19	0.84
1.63	0.82	-0.34	-0.66	1.30	-0.85	1.16
2.51	0.83	-0.12	-0.46	2.27	-1.06	0.96

2.07	0.74	-0.79	-0.35	1.89	-0.66	1.54
4.44	0.89	-0.28	-0.20	4.34	-0.86	1.17
3.58	0.93	0.01	-0.32	3.42	-1.11	0.92
2.93	0.78	-0.65	-0.26	2.81	-0.72	1.43
3.70	0.39	-0.23	-0.48	3.46	-1.66	0.62
2.81	0.75	-0.58	-0.29	2.66	-0.78	1.33
1.58	0.79	-0.43	-0.69	1.23	-0.85	1.22
6.47	0.98	0.27	-0.23	6.36	-1.47	0.71
3.18	0.93	-0.17	-0.32	3.02	-0.96	1.09
2.87	0.99	-0.22	-0.32	2.71	-0.88	1.21
6.36	0.79	-0.35	-0.15	6.28	-0.95	1.14
4.02	0.89	-0.26	-0.24	3.90	-0.94	1.15
7.23	0.87	0.33	-0.28	7.09	-2.00	0.54
5.49	0.80	0.11	-0.30	5.34	-1.62	0.69
1.76	0.90	-0.37	-0.60	1.46	-0.88	1.28
4.31	0.80	-0.02	-0.34	4.14	-1.41	0.82
2.68	0.87	-0.72	-0.29	2.54	-0.73	1.59
4.34	0.71	-0.50	-0.23	4.22	-0.96	1.21
3.06	0.88	-0.43	-0.31	2.91	-0.89	1.31
3.60	0.83	-0.04	-0.40	3.41	-1.36	0.87
4.82	0.98	0.09	-0.29	4.67	-1.33	0.89
3.54	0.82	-0.03	-0.42	3.33	-1.41	0.85
5.34	0.70	-0.44	-0.20	5.23	-1.06	1.13
3.52	0.68	-0.38	-0.34	3.35	-1.14	1.06
3.82	0.70	-0.69	-0.23	3.70	-0.87	1.39
2.74	0.80	-0.65	-0.33	2.58	-0.84	1.44
8.16	0.84	0.21	-0.24	8.04	-1.95	0.63
2.76	0.40	-0.59	-0.49	2.51	-1.23	1.00
7.32	0.86	0.12	-0.23	7.21	-1.65	0.74
2.59	0.62	-0.51	-0.47	2.36	-1.10	1.13
6.45	0.93	0.18	-0.26	6.31	-1.66	0.76
3.73	0.76	0.15	-0.59	3.43	-2.04	0.62
4.28	0.85	-0.63	-0.20	4.18	-0.85	1.48
2.71	0.92	-0.36	-0.39	2.51	-0.99	1.28
6.32	0.89	0.02	-0.23	6.20	-1.45	0.87
3.23	0.86	-0.22	-0.39	3.04	-1.17	1.08
3.88	0.94	-0.09	-0.33	3.72	-1.23	1.03
3.88	0.99	-0.12	-0.31	3.72	-1.15	1.10
2.94	0.84	-0.59	-0.32	2.78	-0.89	1.43
5.09	0.73	-0.43	-0.22	4.98	-1.10	1.16
3.62	0.89	-0.48	-0.27	3.49	-0.93	1.37
5.56	0.78	-0.34	-0.21	5.46	-1.16	1.12
5.94	0.98	0.03	-0.24	5.82	-1.38	0.95
2.90	0.79	-0.35	-0.43	2.69	-1.15	1.14
9.04	0.91	-0.06	-0.15	8.96	-1.36	0.98
3.43	0.99	-0.32	-0.31	3.27	-1.01	1.32
3.74	0.62	-0.26	-0.43	3.53	-1.52	0.88
3.21	0.91	-0.42	-0.34	3.04	-1.02	1.33
6.52	0.97	-0.16	-0.19	6.43	-1.22	1.13
6.95	0.97	0.02	-0.21	6.84	-1.44	0.95
8.41	0.91	-0.07	-0.17	8.32	-1.43	0.97
4.08	0.65	-0.23	-0.41	3.87	-1.60	0.88

3.48	0.97	-0.20	-0.36	3.30	-1.20	1.17
6.66	0.85	-0.23	-0.20	6.56	-1.30	1.09
3.75	0.63	-0.87	-0.27	3.62	-0.96	1.50
4.90	0.85	-0.01	-0.36	4.72	-1.70	0.86
5.61	0.90	-0.31	-0.22	5.50	-1.21	1.22
3.44	0.95	-0.21	-0.39	3.25	-1.28	1.15
4.95	0.98	-0.12	-0.28	4.81	-1.35	1.10
3.54	0.78	-0.58	-0.33	3.37	-1.10	1.35
4.73	0.65	-0.65	-0.25	4.60	-1.14	1.31
5.45	0.72	-0.39	-0.25	5.32	-1.35	1.10
4.31	0.95	-0.17	-0.32	4.15	-1.33	1.13
4.79	0.97	-0.26	-0.26	4.66	-1.22	1.23
3.81	0.83	0.02	-0.53	3.55	-1.87	0.81
3.24	0.90	-0.34	-0.41	3.03	-1.23	1.24
8.51	0.84	0.08	-0.24	8.39	-2.03	0.75
9.19	0.86	0.11	-0.23	9.08	-2.08	0.74
7.73	0.94	0.21	-0.28	7.59	-2.15	0.72
3.52	0.86	-0.80	-0.28	3.38	-0.96	1.65
2.93	0.83	-0.34	-0.51	2.67	-1.37	1.17
10.59	0.83	0.16	-0.23	10.48	-2.40	0.67
2.54	0.82	-0.77	-0.44	2.32	-1.02	1.59
4.94	0.72	-0.16	-0.39	4.75	-1.83	0.88
3.01	0.87	-0.31	-0.50	2.76	-1.37	1.19
7.96	0.88	0.09	-0.26	7.82	-2.05	0.80
3.06	0.90	-0.27	-0.51	2.81	-1.42	1.17
5.37	0.98	0.06	-0.35	5.19	-1.82	0.92
3.43	0.67	-0.48	-0.45	3.20	-1.46	1.15
7.41	0.93	0.09	-0.28	7.27	-2.03	0.84
6.40	0.89	-0.48	-0.20	6.30	-1.26	1.37
3.15	0.98	-0.48	-0.40	2.95	-1.18	1.46
5.62	0.98	-0.10	-0.29	5.47	-1.61	1.08
4.30	0.85	-0.14	-0.43	4.09	-1.76	0.99
3.84	0.82	-0.42	-0.39	3.64	-1.42	1.24
2.80	0.81	-0.82	-0.42	2.59	-1.08	1.63
4.77	0.98	-0.27	-0.31	4.62	-1.42	1.25
8.51	0.98	0.27	-0.30	8.36	-2.51	0.71
3.09	0.86	-0.08	-0.71	2.74	-1.93	0.94
6.56	0.99	-0.20	-0.24	6.44	-1.54	1.19
6.17	0.91	-0.04	-0.32	6.00	-1.94	0.95
10.59	0.84	-0.24	-0.16	10.51	-1.70	1.08
6.86	0.94	-0.16	-0.25	6.74	-1.69	1.10
6.33	0.92	-0.06	-0.31	6.18	-1.91	0.98
10.86	0.70	-0.11	-0.22	10.75	-2.33	0.81
4.49	0.89	-0.18	-0.43	4.28	-1.82	1.07
5.40	0.89	-0.65	-0.24	5.28	-1.26	1.55
1.91	0.83	-0.59	-0.99	1.41	-1.40	1.41
5.54	0.95	-0.26	-0.31	5.38	-1.66	1.21
5.49	0.86	-0.21	-0.36	5.32	-1.89	1.06
7.40	0.96	-0.01	-0.29	7.25	-2.11	0.96
5.91	0.98	-0.09	-0.33	5.74	-1.90	1.07
3.46	0.89	-0.79	-0.37	3.28	-1.21	1.68
5.08	0.59	-0.29	-0.49	4.83	-2.39	0.88

3.88	0.94	-0.48	-0.40	3.68	-1.48	1.42
3.34	0.92	-0.24	-0.59	3.04	-1.80	1.17
3.54	0.93	-0.50	-0.45	3.31	-1.50	1.43
8.00	0.95	0.06	-0.31	7.85	-2.41	0.89
5.46	0.91	0.24	-0.63	5.14	-3.25	0.66
6.22	0.97	0.07	-0.41	6.02	-2.44	0.90
6.98	0.97	-0.06	-0.31	6.82	-2.15	1.03
6.47	0.92	-0.45	-0.26	6.34	-1.63	1.36
6.33	0.63	-0.26	-0.41	6.12	-2.52	0.89
4.68	0.91	-0.53	-0.35	4.50	-1.57	1.44
4.04	0.85	-0.32	-0.52	3.78	-1.95	1.17
10.76	0.92	-0.02	-0.23	10.65	-2.43	0.94
9.47	0.82	-0.33	-0.21	9.37	-1.99	1.15
6.60	0.89	-0.47	-0.26	6.47	-1.69	1.36
5.14	0.61	-0.35	-0.49	4.89	-2.40	0.97
8.98	0.97	0.23	-0.35	8.80	-3.10	0.75
6.94	0.99	0.11	-0.40	6.74	-2.68	0.88
4.41	0.87	-0.46	-0.43	4.20	-1.80	1.33
7.00	0.93	-0.22	-0.31	6.85	-2.10	1.15
3.62	0.77	-0.39	-0.63	3.31	-2.07	1.16
6.03	0.97	-0.35	-0.31	5.88	-1.83	1.31
7.41	0.98	-0.18	-0.29	7.26	-2.11	1.16
5.76	0.93	-0.49	-0.31	5.60	-1.76	1.42
5.16	0.85	-0.13	-0.52	4.90	-2.55	0.98
5.01	0.94	-0.35	-0.41	4.81	-1.95	1.29
4.98	0.85	-0.32	-0.46	4.75	-2.17	1.17
7.05	0.92	-0.30	-0.31	6.90	-2.14	1.22
7.21	0.94	-0.15	-0.34	7.03	-2.40	1.09
6.54	0.97	-0.21	-0.35	6.37	-2.25	1.17
10.42	0.79	-0.51	-0.20	10.32	-2.06	1.29
4.81	0.70	-0.36	-0.55	4.54	-2.51	1.06
4.69	0.76	-0.70	-0.41	4.48	-1.83	1.46
6.46	0.97	-0.42	-0.31	6.31	-1.93	1.39
6.84	0.72	-0.43	-0.35	6.66	-2.35	1.15
7.75	0.99	-0.39	-0.26	7.62	-1.96	1.38
7.97	0.87	-0.43	-0.27	7.83	-2.09	1.30
6.00	0.94	-0.27	-0.39	5.80	-2.26	1.21
8.05	0.97	-0.12	-0.32	7.89	-2.54	1.08
7.21	0.99	-0.03	-0.39	7.01	-2.72	1.02
3.40	0.83	-0.34	-0.82	2.98	-2.45	1.17
8.85	0.91	-0.22	-0.30	8.70	-2.57	1.13
8.57	0.89	-0.40	-0.28	8.43	-2.35	1.29
7.74	0.97	-0.61	-0.26	7.61	-1.95	1.58
5.38	0.82	-0.68	-0.40	5.18	-2.07	1.51
9.04	0.98	-0.14	-0.32	8.89	-2.82	1.13
10.35	0.74	-0.39	-0.27	10.21	-2.80	1.14
9.21	1.00	-0.03	-0.35	9.03	-3.13	1.02
6.80	0.96	-0.35	-0.37	6.62	-2.46	1.31
8.07	1.00	-0.46	-0.29	7.92	-2.31	1.45
4.89	0.85	-0.42	-0.58	4.60	-2.67	1.27
7.23	0.99	-0.12	-0.44	7.01	-3.06	1.11
4.56	0.86	-0.58	-0.55	4.29	-2.37	1.44

8.64	0.97	-0.62	-0.26	8.51	-2.18	1.59
9.93	0.99	-0.51	-0.24	9.81	-2.35	1.50
10.54	0.90	-0.57	-0.23	10.43	-2.42	1.47
8.84	0.91	-0.55	-0.28	8.70	-2.46	1.46
11.09	0.94	-0.16	-0.30	10.94	-3.27	1.10
8.09	0.92	-0.78	-0.27	7.96	-2.15	1.70
10.08	0.89	-0.61	-0.25	9.96	-2.44	1.50
8.14	0.99	-0.18	-0.39	7.94	-3.13	1.17
9.75	0.97	-0.57	-0.25	9.62	-2.40	1.53
6.23	0.98	-0.43	-0.43	6.02	-2.61	1.41
7.69	0.93	-0.47	-0.36	7.51	-2.69	1.40
8.82	0.99	-0.16	-0.38	8.63	-3.27	1.16
6.97	0.96	-0.29	-0.45	6.75	-3.03	1.25
9.06	0.97	-0.20	-0.36	8.88	-3.23	1.17
7.87	0.97	-0.47	-0.35	7.69	-2.66	1.44
7.44	1.00	-0.40	-0.39	7.24	-2.82	1.40
5.84	0.98	-0.46	-0.49	5.60	-2.76	1.44
10.99	0.95	-0.35	-0.28	10.85	-3.07	1.30
7.67	0.95	-0.37	-0.41	7.46	-3.08	1.32
9.68	0.96	-0.13	-0.40	9.48	-3.75	1.09
8.19	0.99	-0.25	-0.42	7.98	-3.37	1.24
8.73	0.97	-0.41	-0.36	8.55	-3.05	1.39
8.39	0.98	-0.41	-0.38	8.20	-3.16	1.39
9.94	0.95	-0.39	-0.34	9.77	-3.34	1.34
9.95	0.97	-0.52	-0.31	9.79	-3.02	1.49
10.63	0.95	-0.52	-0.30	10.48	-3.11	1.47
9.11	0.98	-0.49	-0.35	8.93	-3.13	1.47
9.72	0.99	-0.92	-0.26	9.59	-2.48	1.91
8.41	0.99	-0.55	-0.38	8.22	-3.14	1.54
8.00	0.97	-0.92	-0.34	7.83	-2.63	1.88
8.16	0.98	-0.67	-0.41	7.96	-3.28	1.66
7.77	0.92	-0.62	-0.47	7.53	-3.57	1.54

PHAX_Impact	Function	ABS(PIF)	PHAX_ABS_Impact
2.83	cell cycle	2.13	2.83
2.75	small nuclear RNA	2.39	2.75
2.31		4.50	2.31
1.94		1.92	1.94
1.78		3.96	1.78
1.72	small nuclear RNA	1.73	1.72
1.63		2.84	1.63
1.48	small nuclear RNA	1.50	1.48
1.42		3.58	1.42
1.30	oncostatin m pathway	1.39	1.30
1.29		1.01	1.29
1.27		1.41	1.27
1.26		1.46	1.26
1.16		1.34	1.16
1.13		1.01	1.13
1.12		0.73	1.12
1.08		2.79	1.08
1.08		0.93	1.08
1.04	long non coding RNA	1.97	1.04
1.03	small nuclear RNA	1.24	1.03
1.03		2.67	1.03
1.00		0.87	1.00
0.99		1.27	0.99
0.97		2.14	0.97
0.95		0.81	0.95
0.93		1.76	0.93
0.93		0.94	0.93
0.88	small nuclear RNA	1.06	0.88
0.87		0.64	0.87
0.86		1.92	0.86
0.82		2.04	0.82
0.82		4.13	0.82
0.80		1.37	0.80
0.79	small nuclear RNA	1.01	0.79
0.75		0.78	0.75
0.73		2.01	0.73
0.73		1.65	0.73
0.73		0.66	0.73
0.72		1.23	0.72
0.72		0.77	0.72
0.71		0.96	0.71
0.71		1.96	0.71
0.70		0.75	0.70
0.69		0.44	0.69
0.68		0.68	0.68
0.68		0.50	0.68
0.67		0.85	0.67
0.65		1.59	0.65
0.65	small nuclear RNA	0.77	0.65
0.64		0.63	0.64
0.64		0.41	0.64

0.63		1.04	0.63
0.63		0.69	0.63
0.62		1.59	0.62
0.62		1.39	0.62
0.61	small nuclear RNA	0.74	0.61
0.59		0.90	0.59
0.59		0.61	0.59
0.57		2.24	0.57
0.57		0.97	0.57
0.56		0.65	0.56
0.56		0.98	0.56
0.56		2.11	0.56
0.56		2.18	0.56
0.55	small nuclear RNA	1.41	0.55
0.54		0.58	0.54
0.54	small nuclear RNA	1.12	0.54
0.53		1.86	0.53
0.52		0.83	0.52
0.52		0.76	0.52
0.52		1.52	0.52
0.52		1.04	0.52
0.51		1.20	0.51
0.51		0.43	0.51
0.51		0.84	0.51
0.51		0.72	0.51
0.48		0.52	0.48
0.48		0.51	0.48
0.47		0.64	0.47
0.47		0.52	0.47
0.47		0.31	0.47
0.47		1.01	0.47
0.47		0.56	0.47
0.46		0.77	0.46
0.46		0.30	0.46
0.46		0.83	0.46
0.46		1.45	0.46
0.45		0.47	0.45
0.45		0.36	0.45
0.45		2.21	0.45
0.44		1.72	0.44
0.44		1.60	0.44
0.44		0.47	0.44
0.43		1.28	0.43
0.43		0.45	0.43
0.43		0.55	0.43
0.43		0.47	0.43
0.42		0.66	0.42
0.42		0.47	0.42
0.41		1.58	0.41
0.40		0.83	0.40
0.39		0.40	0.39
0.39		2.38	0.39

0.39	0.35	0.39
0.38	0.45	0.38
0.37	0.29	0.37
0.37	0.55	0.37
0.37	0.42	0.37
0.37	0.45	0.37
0.36	1.35	0.36
0.36	1.05	0.36
0.36	0.81	0.36
0.36	1.97	0.36
0.36	0.98	0.36
0.35	0.62	0.35
0.34	0.34	0.34
0.34	0.71	0.34
0.34	0.97	0.34
0.34	0.38	0.34
0.34	0.64	0.34
0.33	0.72	0.33
0.32	0.53	0.32
0.32	0.34	0.32
0.31	0.64	0.31
0.31	0.23	0.31
0.31	2.19	0.31
0.31	0.43	0.31
0.31	0.20	0.31
0.30	0.23	0.30
0.30	0.62	0.30
0.30	0.26	0.30
0.30	0.32	0.30
0.30	0.47	0.30
0.30	0.37	0.30
0.29	0.77	0.29
0.29	0.41	0.29
0.29	0.39	0.29
0.29	0.97	0.29
0.29	0.29	0.29
0.29	0.19	0.29
0.29	0.31	0.29
0.29	0.35	0.29
0.28	0.46	0.28
0.28	0.27	0.28
0.28	1.39	0.28
0.28	0.24	0.28
0.28	0.22	0.28
0.28	0.23	0.28
0.27	0.94	0.27
0.27	0.41	0.27
0.27	1.32	0.27
0.26	0.23	0.26
0.26	0.29	0.26
0.26	0.24	0.26
0.26	1.39	0.26

0.25	0.80	0.25
0.25	0.23	0.25
0.25	0.28	0.25
0.25	0.25	0.25
0.25	0.16	0.25
0.25	0.35	0.25
0.25	0.51	0.25
0.25	0.21	0.25
0.25	0.24	0.25
0.25	0.68	0.25
0.24	0.22	0.24
0.24	0.32	0.24
0.24	0.65	0.24
0.24	0.70	0.24
0.24	1.24	0.24
0.23	0.28	0.23
0.23	0.21	0.23
0.23	0.35	0.23
0.23	0.44	0.23
0.22	0.62	0.22
0.22	0.55	0.22
0.22	0.13	0.22
0.22	0.22	0.22
0.22	0.17	0.22
0.21	0.15	0.21
0.21	0.20	0.21
0.21	0.20	0.21
0.21	0.63	0.21
0.21	0.24	0.21
0.20	0.42	0.20
0.20	0.16	0.20
0.20	0.28	0.20
0.20	0.36	0.20
0.20	0.42	0.20
0.20	0.40	0.20
0.19	0.22	0.19
0.19	1.12	0.19
0.19	0.43	0.19
0.19	0.44	0.19
0.19	0.65	0.19
0.19	0.23	0.19
0.19	0.11	0.19
0.19	0.48	0.19
0.19	0.31	0.19
0.18	0.78	0.18
0.18	0.21	0.18
0.18	0.17	0.18
0.18	1.06	0.18
0.18	0.12	0.18
0.18	0.14	0.18
0.18	0.49	0.18
0.18	0.24	0.18

0.18	0.34	0.18
0.18	0.23	0.18
0.17	0.62	0.17
0.17	0.26	0.17
0.17	0.49	0.17
0.17	0.25	0.17
0.17	0.28	0.17
0.17	0.32	0.17
0.17	0.29	0.17
0.17	0.50	0.17
0.17	0.88	0.17
0.17	0.16	0.17
0.16	0.23	0.16
0.16	0.34	0.16
0.16	0.13	0.16
0.16	0.40	0.16
0.16	0.16	0.16
0.16	0.70	0.16
0.16	1.15	0.16
0.16	0.63	0.16
0.16	1.43	0.16
0.16	0.38	0.16
0.16	0.21	0.16
0.16	0.31	0.16
0.16	0.23	0.16
0.15	0.10	0.15
0.15	0.14	0.15
0.15	0.26	0.15
0.15	0.47	0.15
0.15	1.75	0.15
0.15	0.14	0.15
0.15	0.33	0.15
0.15	0.15	0.15
0.14	0.67	0.14
0.14	0.20	0.14
0.14	0.24	0.14
0.14	0.50	0.14
0.14	0.33	0.14
0.14	0.16	0.14
0.14	0.19	0.14
0.14	0.21	0.14
0.14	0.19	0.14
0.14	0.21	0.14
0.14	0.18	0.14
0.14	0.15	0.14
0.13	0.14	0.13
0.13	0.19	0.13
0.13	0.32	0.13
0.13	0.12	0.13
0.13	0.62	0.13
0.13	0.15	0.13
0.13	0.30	0.13

0.13	0.66	0.13
0.13	0.25	0.13
0.13	0.14	0.13
0.13	1.48	0.13
0.13	0.13	0.13
0.12	0.42	0.12
0.12	0.31	0.12
0.12	0.20	0.12
0.12	0.16	0.12
0.12	1.03	0.12
0.12	0.24	0.12
0.12	0.12	0.12
0.12	0.09	0.12
0.12	0.18	0.12
0.12	0.46	0.12
0.12	0.25	0.12
0.12	0.12	0.12
0.12	0.14	0.12
0.12	0.29	0.12
0.12	0.12	0.12
0.12	0.07	0.12
0.12	0.27	0.12
0.12	0.09	0.12
0.11	0.18	0.11
0.11	0.24	0.11
0.11	0.16	0.11
0.11	0.12	0.11
0.11	0.15	0.11
0.11	0.12	0.11
0.11	0.34	0.11
0.11	0.17	0.11
0.11	0.13	0.11
0.11	0.23	0.11
0.11	0.16	0.11
0.11	0.59	0.11
0.11	0.30	0.11
0.11	0.23	0.11
0.11	0.36	0.11
0.11	0.19	0.11
0.11	0.14	0.11
0.11	0.23	0.11
0.11	0.21	0.11
0.10	0.44	0.10
0.10	0.27	0.10
0.10	0.15	0.10
0.10	0.24	0.10
0.10	0.16	0.10
0.10	0.19	0.10
0.10	0.15	0.10
0.10	0.19	0.10
0.10	0.76	0.10
0.10	0.17	0.10

0.10	0.19	0.10
0.10	0.13	0.10
0.10	0.13	0.10
0.10	0.09	0.10
0.10	0.12	0.10
0.10	0.14	0.10
0.10	0.13	0.10
0.10	0.24	0.10
0.10	0.43	0.10
0.09	0.17	0.09
0.09	0.58	0.09
0.09	0.10	0.09
0.09	0.15	0.09
0.09	0.12	0.09
0.09	0.14	0.09
0.09	0.15	0.09
0.09	0.23	0.09
0.09	0.20	0.09
0.09	0.73	0.09
0.09	0.11	0.09
0.09	0.20	0.09
0.09	0.10	0.09
0.09	0.21	0.09
0.09	0.47	0.09
0.09	0.16	0.09
0.09	0.74	0.09
0.09	0.18	0.09
0.09	0.13	0.09
0.09	0.15	0.09
0.09	0.17	0.09
0.09	0.10	0.09
0.09	0.07	0.09
0.09	1.14	0.09
0.09	0.33	0.09
0.09	0.10	0.09
0.08	0.13	0.08
0.08	0.14	0.08
0.08	0.18	0.08
0.08	0.14	0.08
0.08	0.26	0.08
0.08	0.27	0.08
0.08	0.22	0.08
0.08	0.10	0.08
0.08	0.46	0.08
0.08	0.44	0.08
0.08	0.16	0.08
0.08	0.21	0.08
0.08	0.26	0.08
0.08	0.11	0.08
0.08	0.51	0.08
0.08	0.18	0.08
0.08	0.16	0.08

0.08	0.13	0.08
0.08	0.65	0.08
0.08	0.13	0.08
0.08	0.19	0.08
0.07	0.10	0.07
0.07	0.30	0.07
0.07	0.08	0.07
0.07	0.12	0.07
0.07	0.15	0.07
0.07	1.42	0.07
0.07	1.05	0.07
0.07	0.33	0.07
0.07	0.73	0.07
0.07	0.26	0.07
0.07	0.12	0.07
0.07	0.10	0.07
0.07	0.30	0.07
0.07	0.38	0.07
0.07	0.35	0.07
0.07	0.91	0.07
0.07	0.16	0.07
0.07	0.13	0.07
0.07	0.13	0.07
0.07	0.33	0.07
0.07	0.10	0.07
0.07	0.36	0.07
0.07	0.28	0.07
0.07	0.11	0.07
0.07	0.15	0.07
0.07	0.12	0.07
0.07	0.28	0.07
0.07	0.54	0.07
0.07	0.17	0.07
0.07	0.12	0.07
0.07	0.22	0.07
0.07	0.09	0.07
0.07	0.10	0.07
0.07	0.19	0.07
0.07	0.82	0.07
0.07	0.12	0.07
0.07	0.15	0.07
0.07	0.17	0.07
0.07	0.11	0.07
0.07	0.11	0.07
0.07	0.34	0.07
0.07	0.09	0.07
0.06	0.14	0.06
0.06	0.12	0.06
0.06	0.11	0.06
0.06	0.09	0.06
0.06	0.12	0.06
0.06	0.40	0.06

0.06	0.20	0.06
0.06	0.13	0.06
0.06	0.16	0.06
0.06	0.08	0.06
0.06	0.15	0.06
0.06	0.53	0.06
0.06	0.14	0.06
0.06	0.07	0.06
0.06	0.10	0.06
0.06	0.61	0.06
0.06	0.08	0.06
0.06	0.38	0.06
0.06	0.22	0.06
0.06	0.11	0.06
0.06	0.87	0.06
0.06	0.58	0.06
0.06	0.15	0.06
0.06	0.31	0.06
0.06	1.27	0.06
0.06	0.12	0.06
0.06	0.49	0.06
0.06	0.28	0.06
0.06	0.40	0.06
0.06	0.39	0.06
0.05	0.14	0.05
0.05	0.14	0.05
0.05	1.66	0.05
0.05	0.05	0.05
0.05	0.20	0.05
0.05	0.12	0.05
0.05	0.14	0.05
0.05	0.48	0.05
0.05	0.06	0.05
0.05	0.13	0.05
0.05	0.96	0.05
0.05	0.11	0.05
0.05	0.13	0.05
0.05	1.47	0.05
0.05	0.46	0.05
0.05	0.07	0.05
0.05	0.44	0.05
0.05	0.16	0.05
0.05	0.09	0.05
0.05	0.16	0.05
0.05	1.47	0.05
0.05	3.49	0.05
0.05	0.12	0.05
0.05	0.12	0.05
0.05	0.12	0.05
0.04	0.51	0.04
0.04	0.08	0.04
0.04	0.32	0.04

0.04	0.87	0.04
0.04	0.27	0.04
0.04	0.09	0.04
0.04	0.12	0.04
0.04	0.60	0.04
0.04	0.14	0.04
0.04	0.15	0.04
0.04	0.15	0.04
0.04	0.09	0.04
0.04	0.25	0.04
0.04	0.13	0.04
0.04	0.24	0.04
0.04	0.06	0.04
0.04	0.08	0.04
0.04	0.11	0.04
0.04	0.37	0.04
0.04	0.26	0.04
0.04	0.16	0.04
0.04	0.11	0.04
0.04	0.09	0.04
0.04	0.10	0.04
0.03	0.16	0.03
0.03	0.13	0.03
0.03	0.04	0.03
0.03	0.11	0.03
0.03	0.23	0.03
0.03	0.20	0.03
0.03	0.17	0.03
0.03	0.09	0.03
0.03	0.43	0.03
0.03	0.07	0.03
0.03	0.11	0.03
0.03	0.06	0.03
0.03	0.15	0.03
0.03	0.14	0.03
0.03	0.02	0.03
0.03	0.07	0.03
0.03	0.15	0.03
0.03	0.27	0.03
0.03	0.24	0.03
0.03	0.21	0.03
0.03	0.42	0.03
0.03	0.18	0.03
0.03	0.11	0.03
0.03	0.12	0.03
0.03	0.26	0.03
0.03	0.08	0.03
0.03	0.12	0.03
0.03	0.45	0.03
0.03	0.17	0.03
0.03	0.19	0.03
0.03	0.13	0.03

0.03	0.19	0.03
0.03	0.16	0.03
0.03	0.18	0.03
0.03	0.09	0.03
0.03	0.21	0.03
0.03	0.24	0.03
0.02	0.38	0.02
0.02	0.18	0.02
0.02	1.32	0.02
0.02	0.19	0.02
0.02	1.74	0.02
0.02	0.13	0.02
0.02	0.55	0.02
0.02	0.12	0.02
0.02	1.11	0.02
0.02	0.27	0.02
0.02	0.67	0.02
0.02	0.10	0.02
0.02	0.07	0.02
0.02	0.14	0.02
0.02	0.13	0.02
0.02	0.10	0.02
0.02	0.21	0.02
0.02	0.17	0.02
0.02	0.11	0.02
0.02	0.11	0.02
0.02	0.19	0.02
0.02	0.11	0.02
0.02	0.28	0.02
0.02	0.07	0.02
0.02	0.12	0.02
0.02	0.12	0.02
0.02	0.16	0.02
0.02	0.14	0.02
0.02	0.05	0.02
0.02	0.08	0.02
0.02	0.18	0.02
0.02	0.10	0.02
0.02	0.09	0.02
0.02	0.11	0.02
0.02	0.18	0.02
0.02	0.09	0.02
0.02	0.49	0.02
0.02	0.29	0.02
0.02	0.90	0.02
0.02	0.20	0.02
0.02	0.17	0.02
0.02	0.02	0.02
0.02	0.15	0.02
0.01	0.20	0.01
0.01	0.20	0.01
0.01	0.12	0.01

0.01	0.11	0.01
0.01	0.01	0.01
0.01	0.49	0.01
0.01	0.17	0.01
0.01	0.15	0.01
0.01	0.12	0.01
0.01	0.15	0.01
0.01	0.15	0.01
0.01	0.09	0.01
0.01	0.12	0.01
0.01	0.13	0.01
0.01	0.05	0.01
0.01	0.31	0.01
0.01	0.09	0.01
0.01	0.04	0.01
0.01	0.14	0.01
0.01	0.13	0.01
0.01	0.21	0.01
0.01	0.09	0.01
0.01	0.35	0.01
0.01	0.12	0.01
0.01	0.09	0.01
0.01	0.17	0.01
0.01	0.53	0.01
0.01	0.11	0.01
0.01	0.26	0.01
0.01	0.17	0.01
0.01	0.34	0.01
0.01	0.05	0.01
0.01	0.05	0.01
0.00	0.33	0.00
0.00	0.31	0.00
0.00	0.43	0.00
0.00	0.25	0.00
0.00	0.45	0.00
0.00	0.15	0.00
0.00	0.29	0.00
0.00	0.12	0.00
0.00	0.13	0.00
0.00	0.16	0.00
0.00	0.15	0.00
0.00	0.11	0.00
0.00	0.31	0.00
0.00	0.21	0.00
0.00	0.00	0.00
0.00	0.21	0.00
0.00	0.13	0.00
0.00	0.00	0.00
0.00	0.15	0.00
0.00	1.43	0.00
0.00	0.17	0.00
0.00	0.34	0.00

0.00	0.13	0.00
0.00	0.27	0.00
0.00	0.15	0.00
-0.01	0.69	0.01
-0.01	0.19	0.01
-0.01	0.12	0.01
-0.01	0.11	0.01
-0.01	0.11	0.01
-0.01	0.10	0.01
-0.01	0.09	0.01
-0.01	0.13	0.01
-0.01	0.04	0.01
-0.01	0.19	0.01
-0.01	0.11	0.01
-0.01	0.57	0.01
-0.01	0.21	0.01
-0.02	0.07	0.02
-0.02	0.94	0.02
-0.02	0.34	0.02
-0.02	0.11	0.02
-0.02	0.10	0.02
-0.02	0.07	0.02
-0.02	0.15	0.02
-0.02	0.15	0.02
-0.02	0.10	0.02
-0.02	0.03	0.02
-0.02	0.41	0.02
-0.02	0.45	0.02
-0.02	0.07	0.02
-0.02	0.13	0.02
-0.02	0.15	0.02
-0.02	0.07	0.02
-0.02	0.25	0.02
-0.02	0.55	0.02
-0.03	0.26	0.03
-0.03	0.40	0.03
-0.03	0.27	0.03
-0.03	0.08	0.03
-0.03	0.21	0.03
-0.03	0.23	0.03
-0.03	0.63	0.03
-0.03	0.11	0.03
-0.03	0.15	0.03
-0.03	0.16	0.03
-0.03	0.28	0.03
-0.03	0.16	0.03
-0.03	0.35	0.03
-0.03	0.08	0.03
-0.03	0.34	0.03
-0.03	0.21	0.03
-0.03	0.18	0.03
-0.03	0.07	0.03

-0.03	0.19	0.03
-0.03	0.88	0.03
-0.03	0.05	0.03
-0.04	0.20	0.04
-0.04	0.16	0.04
-0.04	0.16	0.04
-0.04	0.07	0.04
-0.04	0.48	0.04
-0.04	0.35	0.04
-0.04	0.11	0.04
-0.04	0.11	0.04
-0.04	0.22	0.04
-0.04	0.28	0.04
-0.04	0.14	0.04
-0.04	0.18	0.04
-0.04	0.17	0.04
-0.05	0.11	0.05
-0.05	0.07	0.05
-0.05	0.18	0.05
-0.05	0.15	0.05
-0.05	0.21	0.05
-0.05	0.28	0.05
-0.05	0.08	0.05
-0.05	0.22	0.05
-0.05	0.25	0.05
-0.05	0.07	0.05
-0.05	0.27	0.05
-0.06	0.17	0.06
-0.06	0.34	0.06
-0.06	0.92	0.06
-0.06	0.57	0.06
-0.06	0.19	0.06
-0.06	0.09	0.06
-0.06	0.11	0.06
-0.06	0.09	0.06
-0.06	0.06	0.06
-0.07	0.18	0.07
-0.07	0.10	0.07
-0.07	0.12	0.07
-0.07	0.18	0.07
-0.07	0.40	0.07
-0.07	0.16	0.07
-0.07	0.16	0.07
-0.07	0.30	0.07
-0.07	0.78	0.07
-0.07	0.17	0.07
-0.07	0.72	0.07
-0.07	0.08	0.07
-0.07	0.40	0.07
-0.07	0.06	0.07
-0.08	0.18	0.08
-0.08	0.16	0.08

-0.08	0.22	0.08
-0.08	0.19	0.08
-0.08	0.17	0.08
-0.08	0.85	0.08
-0.08	0.28	0.08
-0.08	0.14	0.08
-0.08	0.12	0.08
-0.08	0.23	0.08
-0.08	0.25	0.08
-0.08	0.25	0.08
-0.08	0.10	0.08
-0.08	0.13	0.08
-0.08	0.10	0.08
-0.08	0.21	0.08
-0.08	0.15	0.08
-0.08	0.11	0.08
-0.09	0.20	0.09
-0.09	0.71	0.09
-0.09	0.30	0.09
-0.09	0.16	0.09
-0.09	0.11	0.09
-0.09	0.13	0.09
-0.09	0.14	0.09
-0.09	0.16	0.09
-0.09	0.19	0.09
-0.09	0.14	0.09
-0.10	0.08	0.10
-0.10	0.10	0.10
-0.10	0.23	0.10
-0.10	0.11	0.10
-0.10	0.21	0.10
-0.10	0.07	0.10
-0.10	0.10	0.10
-0.10	0.23	0.10
-0.10	0.14	0.10
-0.10	0.17	0.10
-0.10	0.19	0.10
-0.10	0.17	0.10
-0.10	0.43	0.10
-0.10	0.60	0.10
-0.11	0.09	0.11
-0.11	0.47	0.11
-0.11	0.17	0.11
-0.11	0.11	0.11
-0.11	0.09	0.11
-0.11	0.86	0.11
-0.11	0.11	0.11
-0.11	0.12	0.11
-0.11	0.28	0.11
-0.11	0.68	0.11
-0.11	0.31	0.11
-0.12	0.17	0.12

-0.12	0.46	0.12
-0.12	0.23	0.12
-0.12	0.09	0.12
-0.12	0.22	0.12
-0.13	0.13	0.13
-0.13	0.25	0.13
-0.13	0.25	0.13
-0.13	0.11	0.13
-0.13	0.29	0.13
-0.13	0.23	0.13
-0.13	0.15	0.13
-0.13	0.15	0.13
-0.14	0.14	0.14
-0.14	0.14	0.14
-0.14	0.15	0.14
-0.14	0.17	0.14
-0.14	0.13	0.14
-0.14	0.23	0.14
-0.14	0.13	0.14
-0.14	0.19	0.14
-0.14	0.21	0.14
-0.14	0.50	0.14
-0.14	0.38	0.14
-0.15	0.27	0.15
-0.15	0.12	0.15
-0.15	0.27	0.15
-0.15	0.24	0.15
-0.15	0.13	0.15
-0.15	0.15	0.15
-0.16	0.24	0.16
-0.16	0.64	0.16
-0.16	0.13	0.16
-0.16	0.15	0.16
-0.16	0.17	0.16
-0.16	0.32	0.16
-0.16	0.16	0.16
-0.16	0.14	0.16
-0.17	0.29	0.17
-0.17	0.12	0.17
-0.17	0.20	0.17
-0.17	0.43	0.17
-0.17	0.20	0.17
-0.17	0.29	0.17
-0.17	0.35	0.17
-0.17	0.39	0.17
-0.18	0.14	0.18
-0.18	0.17	0.18
-0.18	0.62	0.18
-0.19	0.13	0.19
-0.19	0.33	0.19
-0.19	0.12	0.19
-0.19	0.36	0.19

-0.19	0.27	0.19
-0.19	0.27	0.19
-0.19	0.18	0.19
-0.19	0.51	0.19
-0.20	0.11	0.20
-0.20	0.14	0.20
-0.20	0.67	0.20
-0.20	0.12	0.20
-0.20	0.16	0.20
-0.20	0.25	0.20
-0.20	0.75	0.20
-0.20	0.22	0.20
-0.20	0.42	0.20
-0.20	0.19	0.20
-0.20	1.01	0.20
-0.20	0.32	0.20
-0.20	0.95	0.20
-0.20	0.34	0.20
-0.20	0.24	0.20
-0.21	0.82	0.21
-0.21	0.42	0.21
-0.21	0.33	0.21
-0.21	0.20	0.21
-0.21	0.16	0.21
-0.21	0.24	0.21
-0.21	0.26	0.21
-0.21	0.40	0.21
-0.21	0.15	0.21
-0.22	0.17	0.22
-0.22	0.22	0.22
-0.22	0.13	0.22
-0.22	0.73	0.22
-0.22	0.21	0.22
-0.22	0.56	0.22
-0.23	0.38	0.23
-0.23	0.26	0.23
-0.23	0.35	0.23
-0.23	0.21	0.23
-0.23	0.28	0.23
-0.23	0.50	0.23
-0.23	0.58	0.23
-0.24	0.82	0.24
-0.24	0.16	0.24
-0.24	0.20	0.24
-0.24	0.24	0.24
-0.24	0.27	0.24
-0.25	0.79	0.25
-0.25	0.18	0.25
-0.25	0.17	0.25
-0.25	0.27	0.25
-0.25	0.29	0.25
-0.25	0.25	0.25

-0.25	0.25	0.25
-0.26	0.55	0.26
-0.26	0.17	0.26
-0.26	0.27	0.26
-0.27	0.26	0.27
-0.27	0.31	0.27
-0.27	0.18	0.27
-0.27	0.72	0.27
-0.28	0.22	0.28
-0.28	0.61	0.28
-0.28	0.25	0.28
-0.28	0.33	0.28
-0.28	0.31	0.28
-0.28	0.28	0.28
-0.28	0.54	0.28
-0.29	0.34	0.29
-0.29	0.19	0.29
-0.29	0.28	0.29
-0.30	0.46	0.30
-0.30	0.34	0.30
-0.30	0.29	0.30
-0.30	0.19	0.30
-0.30	0.35	0.30
-0.30	0.87	0.30
-0.30	0.26	0.30
-0.31	0.64	0.31
-0.31	0.31	0.31
-0.31	0.28	0.31
-0.31	0.34	0.31
-0.31	1.34	0.31
-0.31	0.56	0.31
-0.31	0.32	0.31
-0.32	0.25	0.32
-0.32	0.29	0.32
-0.32	0.52	0.32
-0.33	0.22	0.33
-0.33	0.82	0.33
-0.33	0.60	0.33
-0.34	0.68	0.34
-0.34	1.97	0.34
-0.34	0.29	0.34
-0.34	0.26	0.34
-0.34	0.28	0.34
-0.34	0.37	0.34
-0.35	0.34	0.35
-0.36	0.28	0.36
-0.36	0.28	0.36
-0.36	0.30	0.36
-0.36	1.02	0.36
-0.37	0.34	0.37
-0.38	0.21	0.38
-0.38	0.50	0.38

-0.38	0.62	0.38
-0.39	0.58	0.39
-0.39	0.25	0.39
-0.39	0.47	0.39
-0.39	0.32	0.39
-0.39	0.39	0.39
-0.39	0.29	0.39
-0.40	0.94	0.40
-0.40	0.34	0.40
-0.40	1.12	0.40
-0.41	0.27	0.41
-0.42	0.40	0.42
-0.43	0.41	0.43
-0.43	0.56	0.43
-0.43	0.33	0.43
-0.43	0.43	0.43
-0.44	1.20	0.44
-0.44	0.32	0.44
-0.44	0.64	0.44
-0.44	0.34	0.44
-0.44	0.37	0.44
-0.45	0.41	0.45
-0.45	0.48	0.45
-0.45	0.36	0.45
-0.45	0.39	0.45
-0.45	0.48	0.45
-0.45	0.34	0.45
-0.46	0.36	0.46
-0.46	0.46	0.46
-0.47	0.68	0.47
-0.47	0.59	0.47
-0.48	0.57	0.48
-0.48	0.54	0.48
-0.48	0.32	0.48
-0.49	1.13	0.49
-0.49	0.75	0.49
-0.49	0.28	0.49
-0.50	0.33	0.50
-0.50	0.38	0.50
-0.50	0.52	0.50
-0.50	0.46	0.50
-0.50	0.63	0.50
-0.51	0.40	0.51
-0.51	1.09	0.51
-0.51	0.49	0.51
-0.51	0.37	0.51
-0.52	0.51	0.52
-0.52	0.34	0.52
-0.52	0.65	0.52
-0.52	0.45	0.52
-0.52	0.40	0.52
-0.52	0.44	0.52

-0.52	1.89	0.52
-0.52	0.32	0.52
-0.53	0.43	0.53
-0.53	0.44	0.53
-0.53	0.48	0.53
-0.53	0.55	0.53
-0.53	0.57	0.53
-0.53	0.50	0.53
-0.54	0.54	0.54
-0.54	0.78	0.54
-0.56	0.73	0.56
-0.56	0.43	0.56
-0.56	0.63	0.56
-0.57	0.58	0.57
-0.57	0.44	0.57
-0.57	1.07	0.57
-0.58	1.18	0.58
-0.58	0.52	0.58
-0.58	0.54	0.58
-0.58	0.60	0.58
-0.59	1.12	0.59
-0.59	1.08	0.59
-0.59	0.44	0.59
-0.59	0.59	0.59
-0.60	0.60	0.60
-0.60	1.18	0.60
-0.60	0.68	0.60
-0.60	0.41	0.60
-0.60	0.52	0.60
-0.60	0.71	0.60
-0.62	0.41	0.62
-0.62	0.65	0.62
-0.63	0.57	0.63
-0.63	0.46	0.63
-0.63	0.59	0.63
-0.63	0.59	0.63
-0.64	0.61	0.64
-0.65	1.03	0.65
-0.65	0.47	0.65
-0.66	0.58	0.66
-0.67	0.42	0.67
-0.67	0.78	0.67
-0.67	1.61	0.67
-0.68	0.57	0.68
-0.68	1.14	0.68
-0.68	0.49	0.68
-0.68	1.37	0.68
-0.69	0.62	0.69
-0.70	0.94	0.70
-0.71	0.54	0.71
-0.72	0.54	0.72
-0.72	1.31	0.72

-0.72		0.90	0.72
-0.72		0.86	0.72
-0.73		0.57	0.73
-0.73		0.96	0.73
-0.74		0.46	0.74
-0.75		1.46	0.75
-0.75		0.61	0.75
-0.75		1.08	0.75
-0.75		1.85	0.75
-0.76		1.98	0.76
-0.76		0.54	0.76
-0.77		0.42	0.77
-0.78		0.47	0.78
-0.78		0.75	0.78
-0.78		0.77	0.78
-0.79		0.68	0.79
-0.80		0.51	0.80
-0.80		0.62	0.80
-0.80		0.65	0.80
-0.80		0.54	0.80
-0.81		0.79	0.81
-0.81		0.64	0.81
-0.81		0.69	0.81
-0.82		1.22	0.82
-0.83		1.03	0.83
-0.83		0.62	0.83
-0.83		0.67	0.83
-0.84		0.72	0.84
-0.85		2.10	0.85
-0.85		1.28	0.85
-0.85		1.04	0.85
-0.87		0.76	0.87
-0.87		0.71	0.87
-0.87		1.77	0.87
-0.89		0.58	0.89
-0.89		1.47	0.89
-0.90		0.66	0.90
-0.92		0.81	0.92
-0.92		0.75	0.92
-0.92	ribosome	0.67	0.92
-0.93		1.30	0.93
-0.95		1.38	0.95
-0.96		0.70	0.96
-0.97	ribosome	0.85	0.97
-0.97		0.85	0.97
-0.98		1.03	0.98
-0.98		0.90	0.98
-0.99		0.82	0.99
-0.99		1.66	0.99
-0.99		1.19	0.99
-0.99		0.85	0.99
-1.01		1.06	1.01

-1.01		0.66	1.01
-1.01	ribosome	0.86	1.01
-1.02		1.11	1.02
-1.03		0.72	1.03
-1.03		1.66	1.03
-1.04		0.78	1.04
-1.04		0.85	1.04
-1.05		1.47	1.05
-1.05		0.96	1.05
-1.07		0.88	1.07
-1.08		0.95	1.08
-1.08		0.94	1.08
-1.09		2.00	1.09
-1.11		1.62	1.11
-1.12		0.88	1.12
-1.15	ribosome	1.41	1.15
-1.16	ribosome	0.73	1.16
-1.16	ribosome	0.96	1.16
-1.17		0.89	1.17
-1.18		1.36	1.18
-1.19		1.33	1.19
-1.20		1.41	1.20
-1.20		1.06	1.20
-1.20		1.14	1.20
-1.20		0.87	1.20
-1.21		0.84	1.21
-1.22		1.95	1.22
-1.23		1.23	1.23
-1.23		1.65	1.23
-1.24		1.10	1.24
-1.26		1.66	1.26
-1.26		2.04	1.26
-1.26		0.85	1.26
-1.26		0.99	1.26
-1.27		1.45	1.27
-1.27		1.17	1.27
-1.27		1.23	1.27
-1.27		1.15	1.27
-1.27		0.89	1.27
-1.27		1.10	1.27
-1.28		0.93	1.28
-1.30		1.16	1.30
-1.31		1.38	1.31
-1.31	ribosome	1.15	1.31
-1.33		1.36	1.33
-1.33		1.01	1.33
-1.34		1.52	1.34
-1.35		1.02	1.35
-1.37		1.22	1.37
-1.37		1.44	1.37
-1.40		1.43	1.40
-1.40		1.60	1.40

-1.40		1.20	1.40
-1.42		1.30	1.42
-1.45	ribosome	0.96	1.45
-1.47		1.70	1.47
-1.48		1.21	1.48
-1.48		1.28	1.48
-1.49		1.35	1.49
-1.49		1.10	1.49
-1.49	ribosome	1.14	1.49
-1.49		1.35	1.49
-1.50		1.33	1.50
-1.50		1.22	1.50
-1.52		1.87	1.52
-1.52		1.23	1.52
-1.53		2.03	1.53
-1.54		2.08	1.54
-1.56		2.15	1.56
-1.59		0.96	1.59
-1.60		1.37	1.60
-1.61		2.40	1.61
-1.61		1.02	1.61
-1.62		1.83	1.62
-1.63		1.37	1.63
-1.64		2.05	1.64
-1.66		1.42	1.66
-1.66		1.82	1.66
-1.67		1.46	1.67
-1.70		2.03	1.70
-1.72		1.26	1.72
-1.73		1.18	1.73
-1.74		1.61	1.74
-1.74		1.76	1.74
-1.76	ribosome	1.42	1.76
-1.77		1.08	1.77
-1.77		1.42	1.77
-1.78		2.51	1.78
-1.81		1.93	1.81
-1.83		1.54	1.83
-1.84		1.94	1.84
-1.85		1.70	1.85
-1.85		1.69	1.85
-1.87		1.91	1.87
-1.89		2.33	1.89
-1.95		1.82	1.95
-1.95		1.26	1.95
-1.98		1.40	1.98
-2.01		1.66	2.01
-2.01		1.89	2.01
-2.03		2.11	2.03
-2.04		1.90	2.04
-2.05		1.21	2.05
-2.09		2.39	2.09

-2.09	1.48	2.09
-2.11	1.80	2.11
-2.14	1.50	2.14
-2.15	2.41	2.15
-2.16	3.25	2.16
-2.19	2.44	2.19
-2.21	2.15	2.21
-2.22	1.63	2.22
-2.24	2.52	2.24
-2.27	1.57	2.27
-2.28	1.95	2.28
-2.29	2.43	2.29
-2.29	1.99	2.29
-2.29	1.69	2.29
-2.32	2.40	2.32
-2.32	3.10	2.32
-2.37	2.68	2.37
-2.40	1.80	2.40
-2.41	2.10	2.41
-2.41	2.07	2.41
-2.41	1.83	2.41
-2.46	2.11	2.46
-2.50	1.76	2.50
-2.51	2.55	2.51
-2.51	1.95	2.51
-2.53	2.17	2.53
-2.60	2.14	2.60
-2.63	2.40	2.63
-2.64	2.25	2.64
-2.66	2.06	2.66
-2.67	2.51	2.67
-2.68	1.83	2.68
-2.69	1.93	2.69
-2.69	2.35	2.69
-2.71	1.96	2.71
-2.73	2.09	2.73
-2.74	2.26	2.74
-2.76	2.54	2.76
-2.77	2.72	2.77
-2.87	2.45	2.87
-2.90	2.57	2.90
-3.02	2.35	3.02
-3.08	1.95	3.08
-3.13	2.07	3.13
-3.18	2.82	3.18
-3.19	2.80	3.19
-3.20	3.13	3.20
-3.22	2.46	3.22
-3.36	2.31	3.36
-3.39	2.67	3.39
-3.40	3.06	3.40
-3.42	2.37	3.42

-3.47		2.18	3.47
-3.52		2.35	3.52
-3.55		2.42	3.55
-3.58		2.46	3.58
-3.60		3.27	3.60
-3.66		2.15	3.66
-3.66		2.44	3.66
-3.67		3.13	3.67
-3.68		2.40	3.68
-3.68		2.61	3.68
-3.76		2.69	3.76
-3.78		3.27	3.78
-3.78		3.03	3.78
-3.79		3.23	3.79
-3.84		2.66	3.84
-3.95		2.82	3.95
-3.97		2.76	3.97
-4.00		3.07	4.00
-4.08		3.08	4.08
-4.09		3.75	4.09
-4.17		3.37	4.17
-4.24		3.05	4.24
-4.39		3.16	4.39
-4.48		3.34	4.48
-4.50		3.02	4.50
-4.59		3.11	4.59
-4.61	ECM remodelling, tumour suppressor	3.13	4.61
-4.74		2.48	4.74
-4.84		3.14	4.84
-4.95		2.63	4.95
-5.44		3.28	5.44
-5.50	prostaglandin reductase 2	3.57	5.50

Figure S1

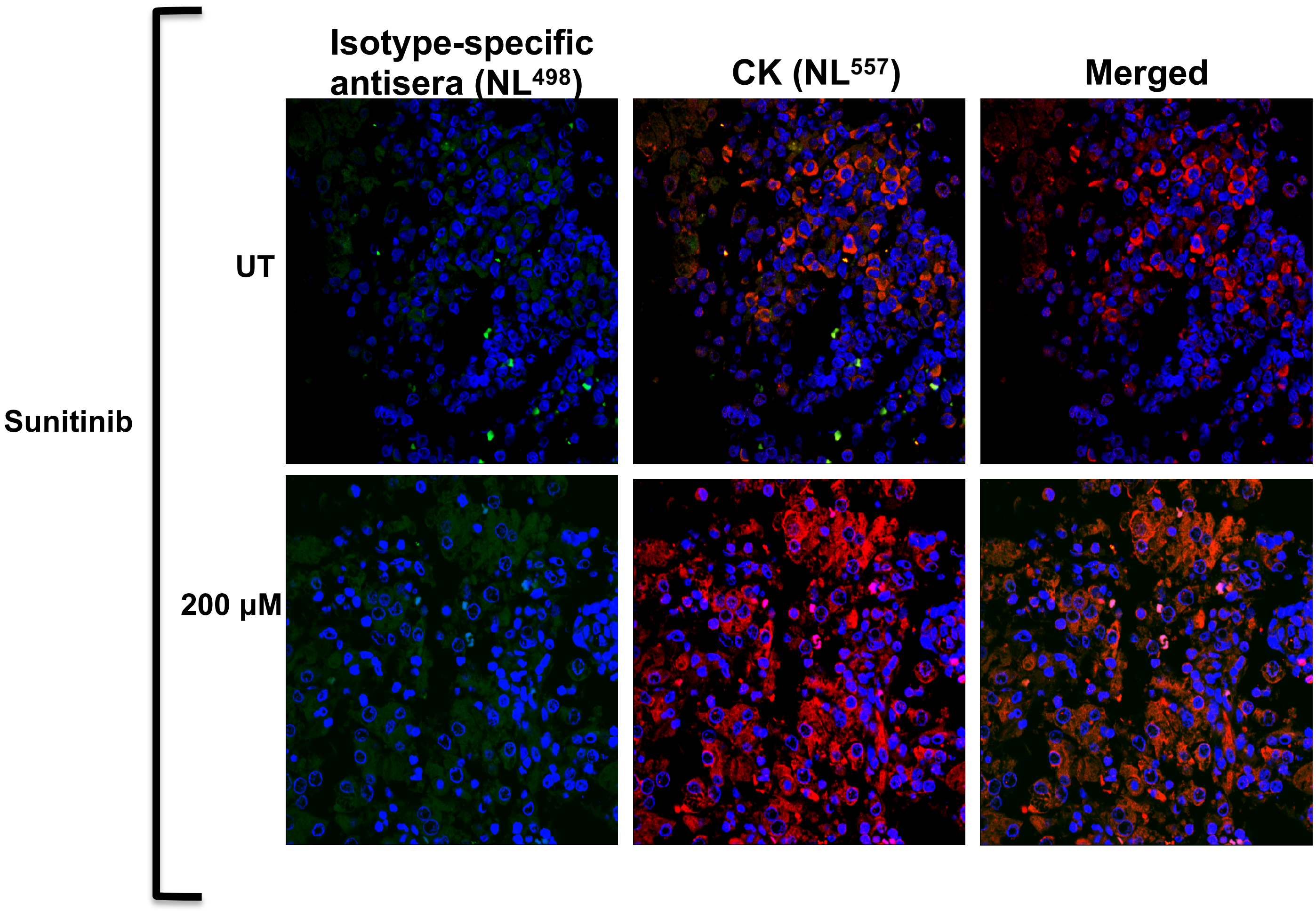
Isotype-specific antisera (NL498)
CK (NL557)
Merged
Sunitinib
UT
200 μM

Figure S2

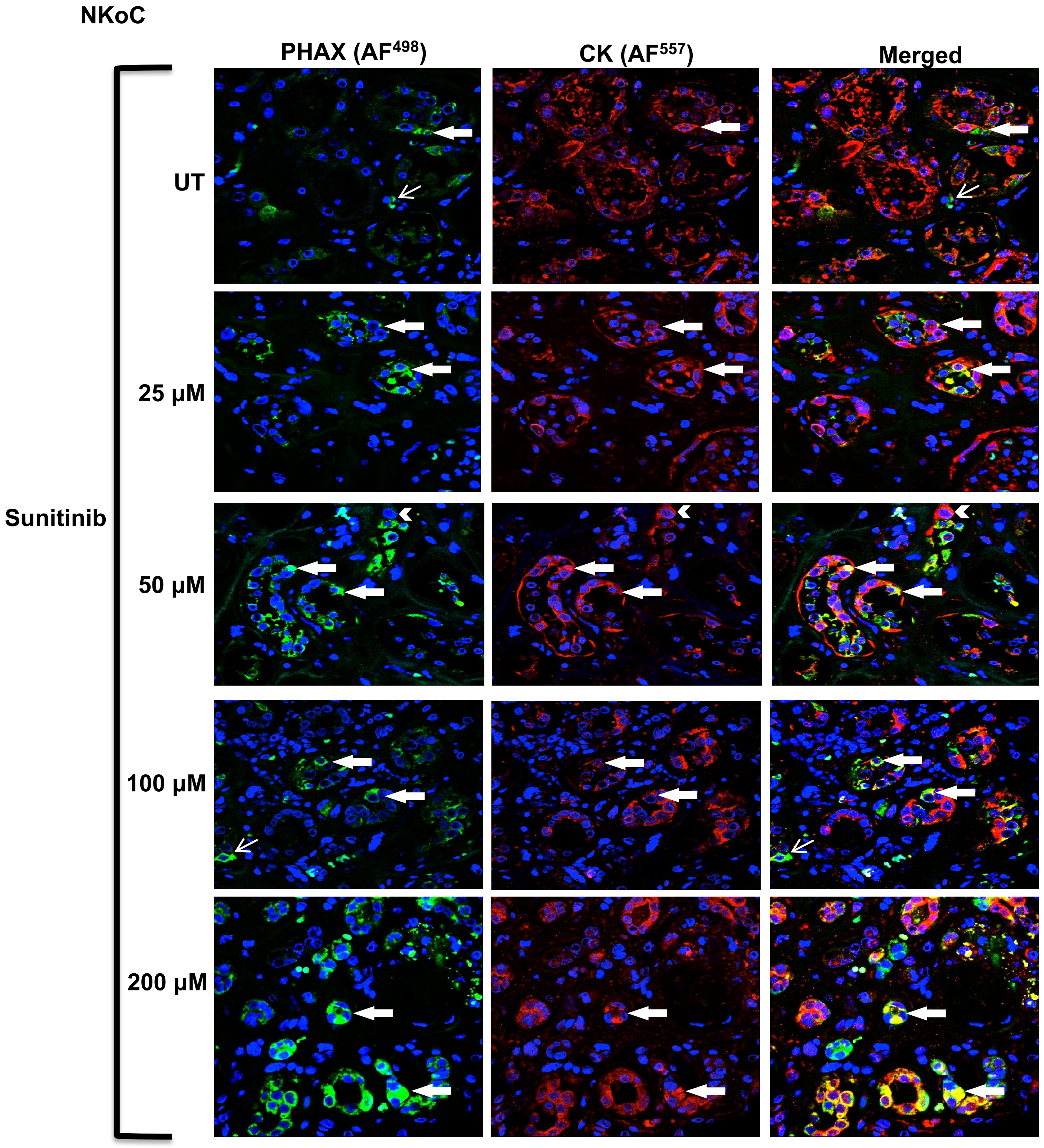
NKoC
PHAX (AF498)
CK (AF557)
Merged
UT
25 µM
50 µM
100 µM
200 µM
Sunitinib

Figure S3

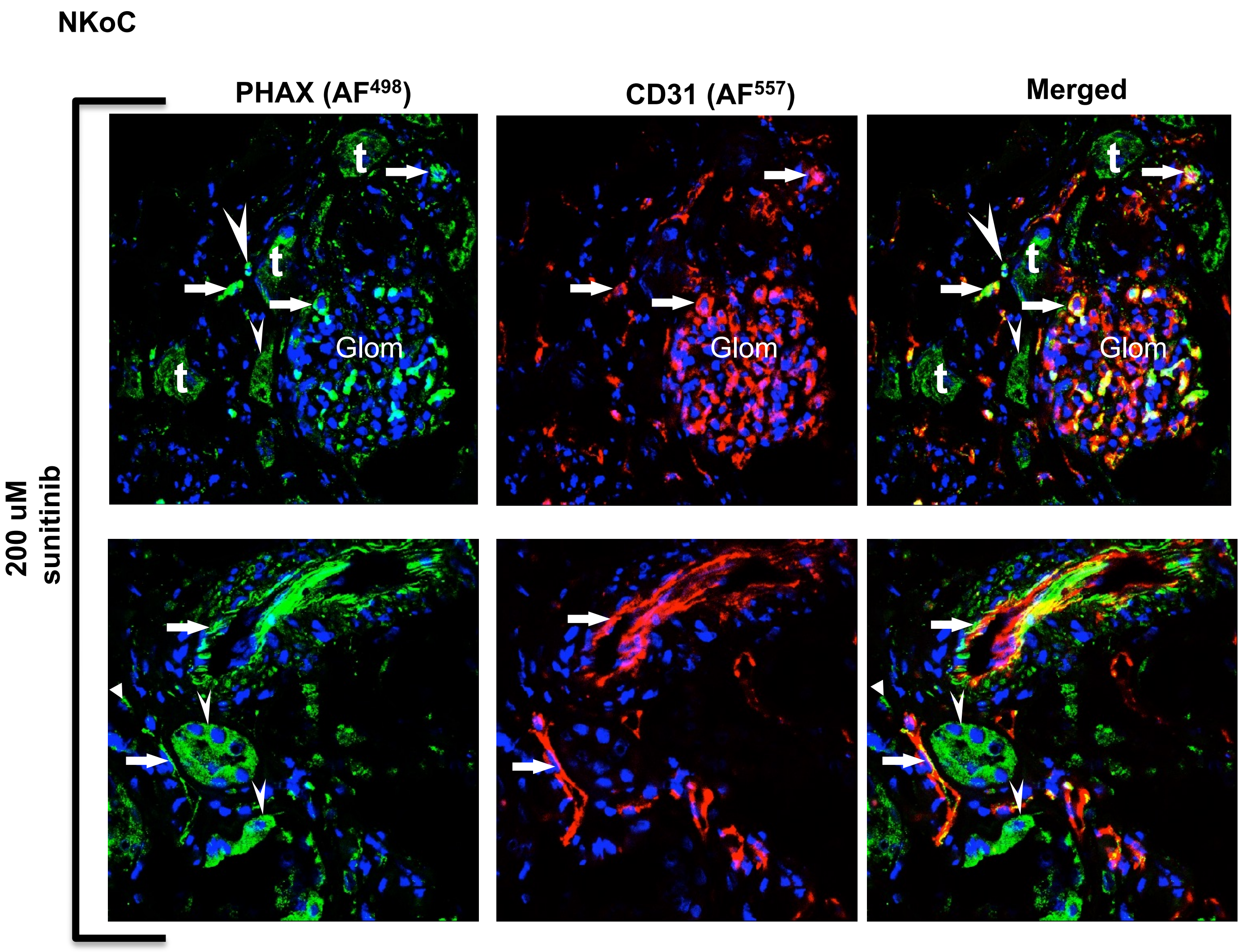

NKoC
PHAX (AF498)
CD31 (AF557)
Merged
200 uM sunitinib
t
t
t
Glom
Glom
t
t
t
Glom

Figure S4

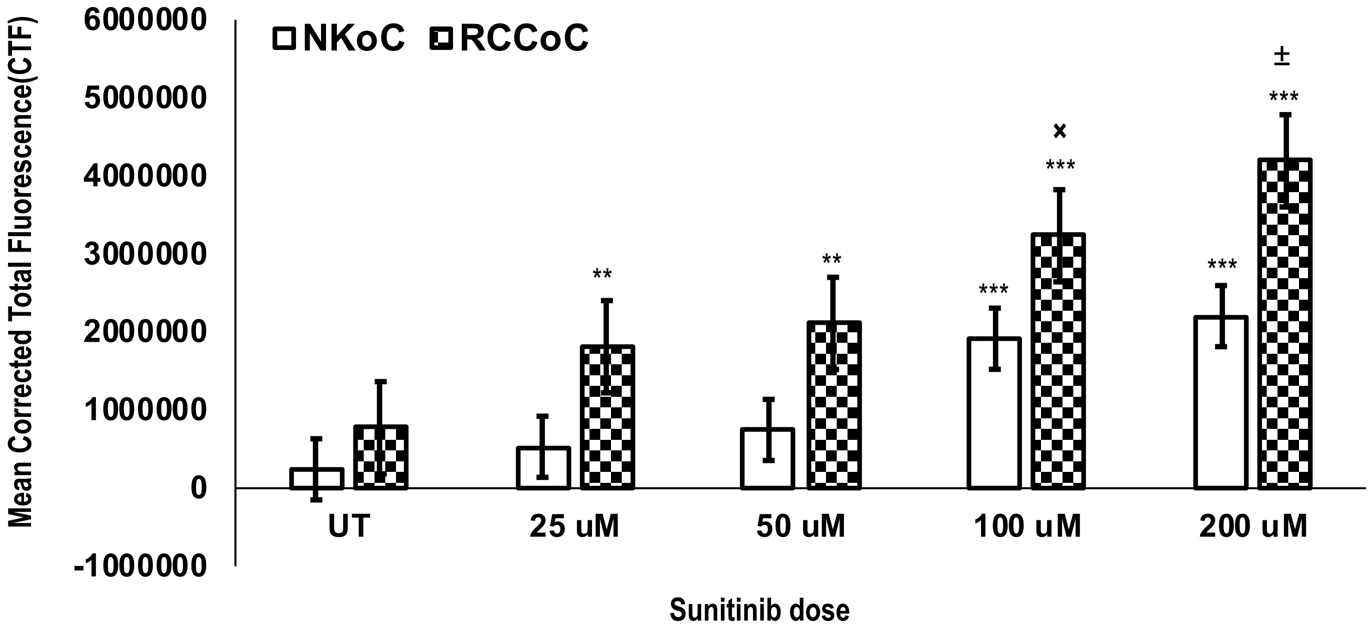

NKoC
RCCoC
Mean Corrected Total Fluorescence(CTF)
6000000
5000000
4000000
3000000
2000000
1000000
0
-1000000
UT
25 uM
50 uM
100 uM
200 uM
Sunitinib dose
**
**
***
***
×
***
***
±
